# Comparative analysis of chloroplast genomes in *Vasconcellea pubescens* A.DC. and *Carica papaya* L.

**DOI:** 10.1038/s41598-020-72769-y

**Published:** 2020-09-25

**Authors:** Zhicong Lin, Ping Zhou, Xinyi Ma, Youjin Deng, Zhenyang Liao, Ruoyu Li, Ray Ming

**Affiliations:** 1 College of Agriculture, Center for Genomics and Biotechnology, Fujian Provincial Key Laboratory of Haixia Applied Plant Systems Biology, Fujian Agriculture and Forestry University, Fuzhou, 350002 Fujian China; 2 College of Life Sciences, Fujian Agriculture and Forestry University, Fuzhou, 350002 Fujian China; 3 Fruit Research Institute, Fujian Academy of Agricultural Sciences, Fuzhou, 350013 Fujian China; 4 Department of Plant Biology, University of Illinois at Urbana-Champaign, Urbana, IL 61801 USA

**Keywords:** Genetic variation, Agricultural genetics, Genome

## Abstract

The chloroplast genome is an integral part of plant genomes in a species along with nuclear and mitochondrial genomes, contributing to adaptation, diversification, and evolution of plant lineages. In the family Caricaceae, only the *Carica papaya* chloroplast genome and its nuclear and mitochondrial genomes were sequenced, and no chloroplast genome-wide comparison across genera was conducted. Here, we sequenced and assembled the chloroplast genome of *Vasconcellea pubescens* A.DC. using Oxford Nanopore Technology. The size of the genome is 158,712 bp, smaller than 160,100 bp of the *C. papaya* chloroplast genome. And two structural haplotypes, LSC_IRa_SSCrc_IRb and LSC_IRa_SSC_IRb, were identified in both *V. pubescens* and *C. papaya* chloroplast genomes. The insertion-deletion mutations may play an important role in *Ycf1* gene evolution in family Caricaceae. *Ycf2* is the only one gene positively selected in the *V. pubescens* chloroplast genome. In the *C. papaya* chloroplast genome, there are 46 RNA editing loci with an average RNA editing efficiency of 63%. These findings will improve our understanding of the genomes of these two crops in the family Caricaceae and will contribute to crop improvement.

## Introduction

The family Caricaceae includes six genera and thirty-five species^1–3^. Among them, *Vasconcellea pubescens* A.DC, also named highland or mountain papaya, is a tropical crop native to the Ecuadorian Andes, distributed at high altitudes (above 1500 m). It was introduced to Chile about 60 years ago, and gradually became an important cash crop in central Chile^4,5^. *V. pubescens* has many economic values, and is commonly used to produce canned fruit, juice, jam and sweets^5^.

Chloroplasts are essential organelles in plants, which evolved from a cyanobacterium via endosymbiosis, specialized in photosynthesis. They are active metabolic centers, contributing to fatty acid and amino acid synthesis pathways^6,7^. The size of chloroplast genomes is small, ranging from 120 to 220 kb^8^. It consists of four regions, two inverted repeats (IRs), a large single-copy region (LSC) and a small single-copy region (SSC). LSC and SSC are separated by the IR regions. The structure of chloroplast genomes can be circular or linear, and the four regions can arrange differently^7,9^. There are 110–130 genes distributed in a chloroplast genome, which are involved in photosynthesis, transcription and translation processes^10^. The gene number and gene composition are conserved in chloroplast genomes of most plants, making them suitable for evolutionary analysis, Whereas variable regions of a genome provide sequence resources for developing molecular markers^10–13^.

RNA editing is a post-transcriptional modification of RNA, distinct from other modifications such as 5 prime capping and RNA splicing^14^. RNA editing events have been reported in many plant chloroplast genomes, such as maize, Spirodela, rice, and tobacco^14–17^. In plants, most RNA editing events detected in chloroplast mRNAs show cytidine to uridine conversion or sometimes an adenosine to inosine transition^15^. RNA editing in chloroplast mRNAs is more likely a rescue of mutation event than producing a new protein^15,16^. Members of PPR protein family appear to be involved, and a *cis*-element is required for editing site recognition^19–23^. RNA editing sites tend to favor the first and second bases of a codon, since most detected editing sites are the first two bases^15,23^.

*Ycf1* is a gene with unknown function in chloroplast genomes. At structural level, it is usually predicted to contain six to eight trans-membrane domains at the N terminus and a variable hydrophilic C-terminal structural domain^25^. Moreover, due to high variability of *Ycf1* gene sequences, it can be used as a promising plasmid DNA fragment for barcode development^11,12^. The function of YCF1 is still unclear, though there are some controversial reports that YCF1 included in the translocon at the inner envelope membrane of chloroplasts (TIC) system^24,26–31^.

Genomic researches on chloroplasts have been growing exponentially. Thanks to rapid development of sequencing technologies, the cost of sequencing is decreasing, and more chloroplast genomes will be deciphered and published. Long sequence reads facilitate assembly of chloroplast genomes. Here, we assembled and annotated the mountain papaya chloroplast genome using Oxford Nanopore Technology (ONT) reads, compared it with the *C. papaya* chloroplast genome, explored RNA editing profile of *C. papaya* chloroplast, and analyzed the evolution of the *Ycf1* gene in the Caricaceae family.

## Materials and methods

### Sample collection and sequencing

Young leaves of *V.* *pubescens* were collected from a greenhouse in Fujian Agriculture and Forestry University, Fuzhou, China, and some of them were sent to Biomarker Technologies Corporation (Beijing) for DNA extraction, library construction, and Oxford Nanopore technology sequencing. The others were used to extract DNA in our lab using CTAB protocol and then sent to Biomarker Technologies Corporation (Beijing) Co., Ltd for library construction and next generation sequencing.

### Chloroplast genome assembly and annotation

All sequence data from Nanopore sequencing was first corrected by Canu-1.7^32^ and the reads were mapped to *C. papaya* chloroplast genome (GenBank: EU431223.1), which was downloaded from NCBI, using BLASR software^33^ with the parameters minMatch 15, minAlnLength 5000. All mapped reads were assembled via Smartdenovo (https://github.com/ruanjue/smartdenovo) to generate the first version of *V. pubescens* chloroplast genome. NGS data was then blasted to the first version genome and the reads mapped were used for chloroplast genome assembly using SPAdes3.13.1^34^, to generate the second version of the *V. pubescens* chloroplast genome. The two versions were compared and mutually corrected. The unclear parts of the genome were corrected by Sanger sequencing to generate the final version of the *V. pubescens* chloroplast genome. Gene annotation was performed using Geseq^35^ and CPGAVAS2^36^, and annotation results were manual corrected. The revised annotation gbf file was uploaded to OGDRAW^37^ to draw the chloroplast genome map.

### Chloroplast genome analysis

The *C. papaya* and *V. pubescens* chloroplast genomes were aligned using the MAFFT7.0 software^38^ and the result was uploaded to DnaSP6.0^39^ for DNA polymorphism analysis. GC content and codon usage bias analysis were performed in the GALAXY^40^ platform. We used mVISTA^41^ to compare chloroplast genomes of *C. papaya* and *V. pubescens*. MISA v1.0^42^ (misa.ini parmeter: 1–10 2–5 3–4 4–3 5–3 6–3) and REPuter^43^ (minimal repeat size, 18 bp) were used to analyze simple sequence repeats (SSR) and repetitive sequences. PREP-cp^44^ was used to predict RNA editing sites of protein coding genes. Single nucleotide polymorphisms and indels were detected using DnaSP6.0 and a python script (https://www.biostars.org/p/119214/). Non-synonymous and synonymous rates of substitution analysis were done using ParaAT^45^ and KaKs_Calculator^46^. Haplotype detection was done using Cp-hap^9^ with both *C. papaya* and *V. pubescens* Nanopore reads (> 30 kb). The haplotype structure was confirmed using PCR and Sanger Sequencing. All related figures were drawn using R 3.4.0, IBS^47^, DNAMAN 6.0 software (Lynnon Biosoft) or OFFICE 2010.

### *Ycf1* gene analysis

*Ycf1* gene sequences from *V. pubescens* and *C. papaya* were aligned, and identical sequences were selected to design primers to amplify *Ycf1* genes using leaf DNA from *Vasconcellea monoica*, *Jacaratia spinosa*, *Jarilla caudata*, *Jarilla heterophella*, and *Jarilla chocola*. The phylogenetic tree of six *Ycf1* genes was constructed by MEGA7.0^48^ and MA model. Arabidopsis *Ycf1* gene was selected as an out-group for analysis. DNA polymorphism and Ka/Ks ratio of *Ycf1* genes were calculated, and YCF1 protein sequences were input to calculate dN/dS value using MEGA7.0 and easycodeML^49^. The trans-membrane region was predicted using TMHMM Server v. 2.0^50^. The conserved motifs of *Ycf1* genes were searched using MEME suit^51^.

### Variant calling for RNA editing site

Four replications of RNA-seq data (unpublished) from *C. papaya* leaves were used for variant calling. The paired-end reads were mapped to the *C. papaya* chloroplast genome using HISAT2^52^ and were sorted and converted into a bam file using samtools^50,51^. Variant calling with quality (> or = 20) and depth (> 10) were conducted using samtools (mpileup and bcftools). Variants in at least two replications were selected as final RNA editing candidate sites. Using DP4 value from final variant calling VCF file, we calculated RNA editing efficiency by edited reads divided by total mapped reads.

## Result

### Global comparison of *V. pubescens* and* C. papaya* chloroplast genomes

**Figure 1:**
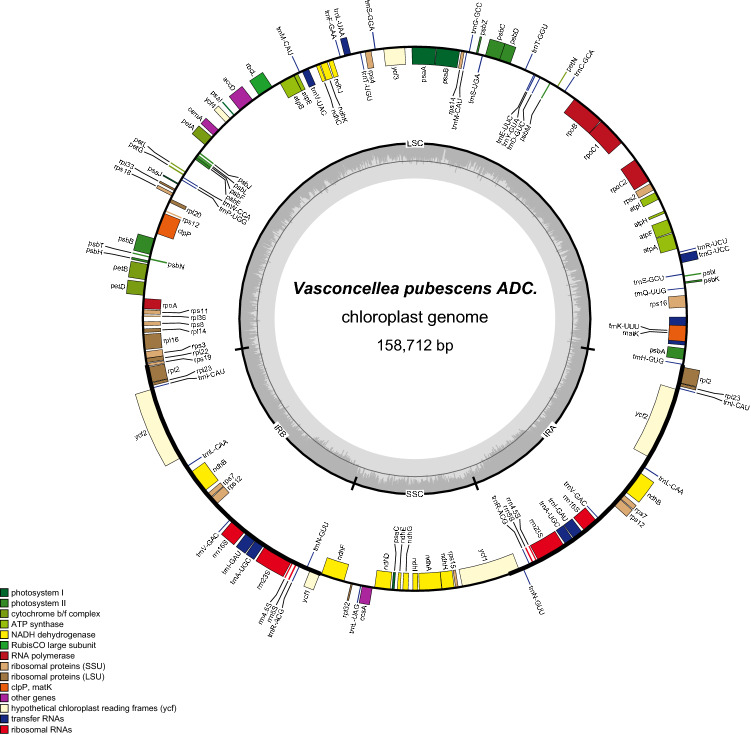
Gene map of the *Vasconcellea pubescens*. Thick lines indicate the extent of the inverted repeat regions (IRa and IRb), which separate the genome into small (SSC) and large (LSC) single copy regions. Genes drawn inside the circle are transcribed clockwise, and those outside are transcribed counter clockwise. Different colors represent different functional groups of Genes.

**Figure 2:**
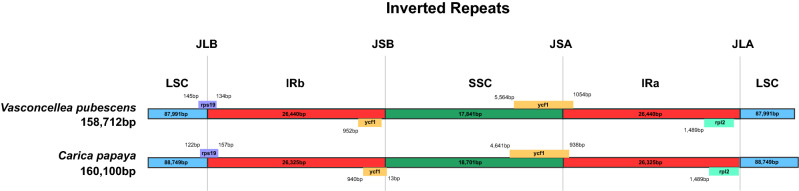
Comparison of the borders of LSC, SSC, and IR regions of chloroplast genomes of *Vasconcellea pubescens* and *Carica papaya*.

There were 124,170 ONT reads mapped to the *C. papaya* chloroplast genome, representing 10.3% of the total corrected ONT reads (1,195,269). These reads were assembled using Smartdenovo returned 2.4 M consensus unitigs, and the N50 is 118,474 bp. After self-blast, two unitigs were selected to assemble a closed circle chloroplast genome. We also did polishing and manually comparing with the chloroplast genome assembled with Illumina NGS data. Finally, we assembled the final version of the *V. pubescens* chloroplast genome. The size of *V. pubescens* chloroplast genome is 158,712 bp, 1388 bp smaller than the 160,100 bp in *C. papaya* chloroplast genome (deposited in GenBank: MT062856). The chloroplast genome of *V. pubescens* contains 131 genes, the same number as that of *C. papaya*, including 82 protein-coding genes, 2 pseudo genes (Ycf1* and infA*), 8 rRNA and 37 tRNA genes (Fig. 1, Table 1). In the *V. pubescens* chloroplast genome, the sizes of the LSC region and SSC region are 87,991 bp and 17,841 bp, respectively, which are separated by two IR regions. The IR region of *V. pubescens* is 26,440 bp, larger than that of *C. papaya*’s 26,325 bp. The *rps19* genes in both *V. pubescens* and *C. papaya* crossed the LSC and IRb boundaries (Fig. 2). In the *V. pubescens* chloroplast genome, 15 protein-coding genes have introns, of which 11 genes have one intron and four genes have two introns, the same as those in *C. papaya* (S2.3). The GC content is the same at 37% in both chloroplast genomes.The untranslated region (UTR) had the highest GC content, whereas the lowest is in the intergenic region. The GC content of exons and introns was somewhere in between (Fig S1).

**Table 1:** Comparison of gene number, gene type and different regions size of *Vasconcellea pubescens* and *Carica papaya* from the whole-genome wide.

	*V. pubescens*	*C. papaya*
Total gene number	131	131
Protein coding gene	84	84
Pseudo gene	2 (Ycf1*, infA*)	2 (Ycf1*, infA*)
rRNA	8	8
tRNA	37	37
IRa	17	17
IRb	18 (rps19 spans the IRb/LSC boundary)	18 (rps19 spans the IRb/LSC boundary)
LSC	82	83
SSC	14	13
Total cpDNA size (bp)	158,712	160,100
IR size (bp)	26,440	26,325
LSC size (bp)	87,991	88,749
SSC size (bp)	17,841	18,701

### Structural variation and DNA polymorphism in *V. pubescens* and *C. papaya*

We compared *V. pubescens* and *C. papaya* chloroplast genome sequence similarity using mVISTA with *C. papaya* chloroplast as the reference. There is a high degree of synteny between the two chloroplast genomes, but sequences of genes, UTR, and CNS were highly variable (Fig S2). Three low consistency regions with less than 50% identity were found, two of which located in the LSC region (49,469–49,840 bp, 53,979–54,533 bp), while the third located in the SSC region (118,717–119,363 bp). In order to elucidate sequence nucleotide divergence between *V. pubescens* and *C. papaya*, we used DnaSP6.0 to analyze sequence nucleotide variability with a 600 bp sliding-window and a 200 bp step size. The highest value was observed in genes *psbZ*, *trnG-GCC*, between *trnH-GUG* and *psbA*, *trnS-GCU* and *trnG-UCC*, and gene *rps16* (S2.11). We also found that average DNA sequence variation in SSC region is higher than that in LSC. Lower divergences are in IR regions (S2.4). We also investigated single nucleotide polymorphisms (SNPs) and indels between the two genomes with 2,321 SNPs and 387 indels found (S2.5).

### SSR and repetitive sequences

**Figure 3:**
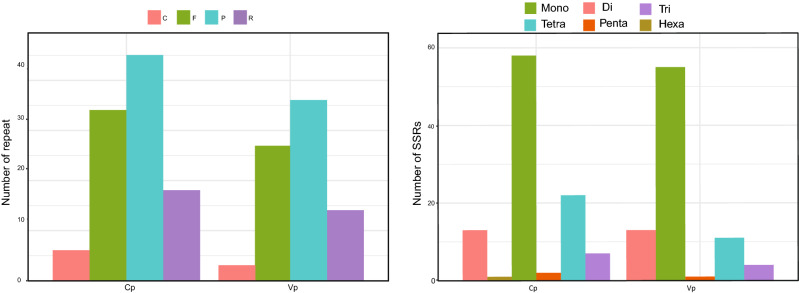
Repeats and SSRs number comparison of *Vasconcellea pubescens* (Vp) and *Carica papaya* (Cp) chloroplast genome.

Six types of SSRs were founded in the *C. papaya* chloroplast genome, including: single nucleotide, dinucleotide, trinucleotide, tetranucleotide, pentanucleotide, and hexanucleotide. Five types of SSRs were found in the *V. pubescens* chloroplast genome, including single nucleotides, dinucleotides, trinucleotides, tetranucleotides, and pentanucleotides. *C. papaya* had 103 SSRs compared to 84 in *V. pubescens. C. papaya* had a higher number of SSRs in each type except for the dinucleotide type. Repeated sequence information is important for phylogenetic analysis, which plays an important role in rearrangements of the genome^54^. 103 repetitive sequences were found in *C. papaya*, while 86 were found in *V. pubescens*. Similar to SSRs, all types of repetitive sequences in *V. pubescens* are fewer than those in *C. papaya*, which has 45 palindromic repeats, 34 direct repeats, 18 inverted repeats, and 6 complementary repeats, whereas *V. pubescens* has 36 palindromic repeats, 27 direct repeats, 14 inverted repeats, and 3 complementary repeats (Fig. 3).

### RNA editing

In seed plants, RNA editing in the chloroplast converts cytidine to uridine (C to U RNA editing), some of which can affect protein function^54,55^. RNA editing in chloroplast was reported to contribute to chloroplast-to-nucleus signaling^56,57^. We predicted RNA editing sites in the *V. pubescens* and *C. papaya* chloroplast genomes. 52 RNA editing sites were detected in both *V. pubescens* and *C. papaya*, and coding genes of the subunits of the NAD(P)H dehydrogenase complex appear to undergo extensive RNA editing (S2.6). *ndhB* (12/12 loci) had the highest number of RNA edits, followed by *ndhD* (8/8 loci), *ndhA* (4/4 loci), *rpoC2* (4/3 loci), *ndhF* (3/3 loci) and *ndhG* (3/3 loci). In *V. pubescens*, 56% of the editing sites occurred on the serine codon, and 54% of the editing events changed the target codon to the leucine codon. In *C. papaya*, 50% of the editing sites occurred on the serine codon and 50% of the editing sites changed to the leucine codon. For locations of editing sites, 80% of the RNA editing events in *V. pubescens* occurred at the second nucleotide of the codon, 20% at the first nucleotide, and no change was detected at the third nucleotide. The type and pattern of RNA editing in *C. papaya* were similar to those in *V. pubescens*. The start codon of *ndhD* was predicted to be edited from ACG to ATG, back into the normal start codon. Most RNA editing sites changed amino acids from polar to nonpolar, resulting in an increased protein hydrophobicity.

**Figure 4:**
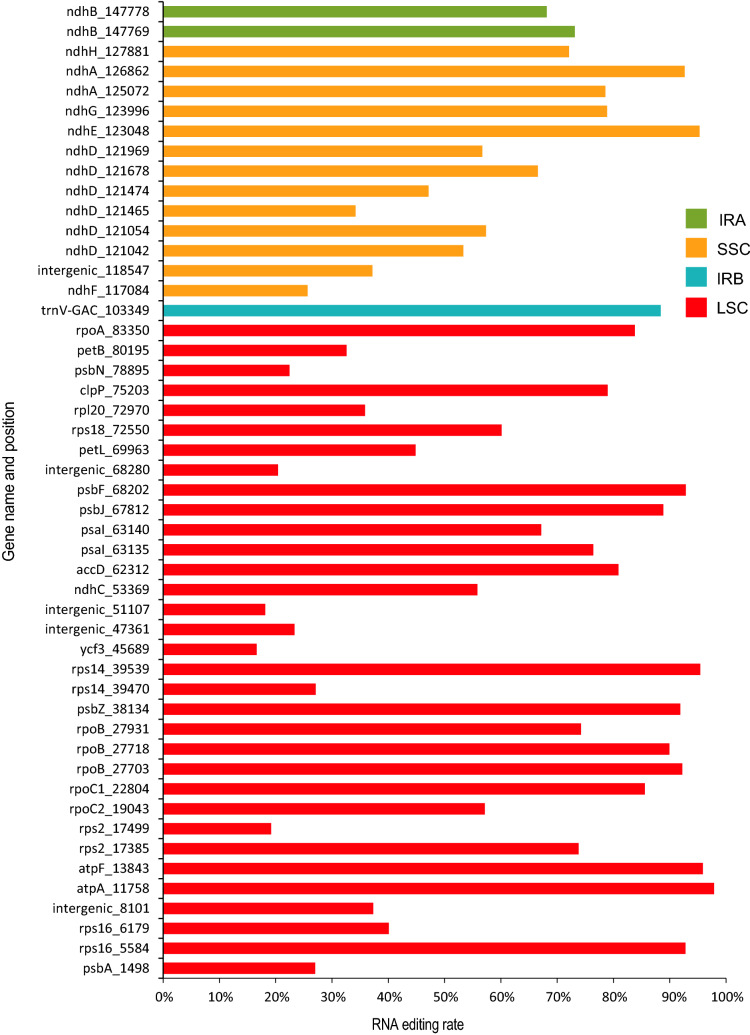
RNA editing efficiency plot of *Carica papaya* chloroplast RNA*.* Different colors respect for different Region of chloroplast genome, red for LSC, green for IRa, yellow for SSC and blue for IRb region. X axis represent RNA efficiency, and the number after the gene name in the Y axis represent the editing site.

We used RNA-seq data to explore RNA editing events in *C. papaya* chloroplast genome and detected 46 RNA editing sites in 30 genes and 3 intergenic regions. All of them are C-to-U conversion. Of all editing loci, 15 (32%) located in the NDH components coding genes. The ribosomal protein-coding genes have 8 sites. Among them, the *ndhD* gene has the most frequent RNA editing sites (6 sites) and the *rpoB* has 3 sties. We detected only 2 loci in the *ndhB* gene, though this gene was predicted to be the one with the highest editing frequency by PREP. We calculated RNA editing efficiency based on the DP4 value in the VCF file, which is variable, ranging from 15 to 98%, with an average editing efficiency of 63%. RNA editing efficiency is also different between the same gene editing sites. For example, RNA editing efficiency of the *ndhD* ranges from 34 to 66% (Fig. 4). We selected three sites for verification based on either they showed low quality during the filter step in some replications or they are not detected in some replications, and all of them were verified by Sanger sequencing results, which showed a duplication peak or different sequencing results from different transformants in the editing sites (Fig. S4–S6).

### Codon usage bias

**Figure 5:**
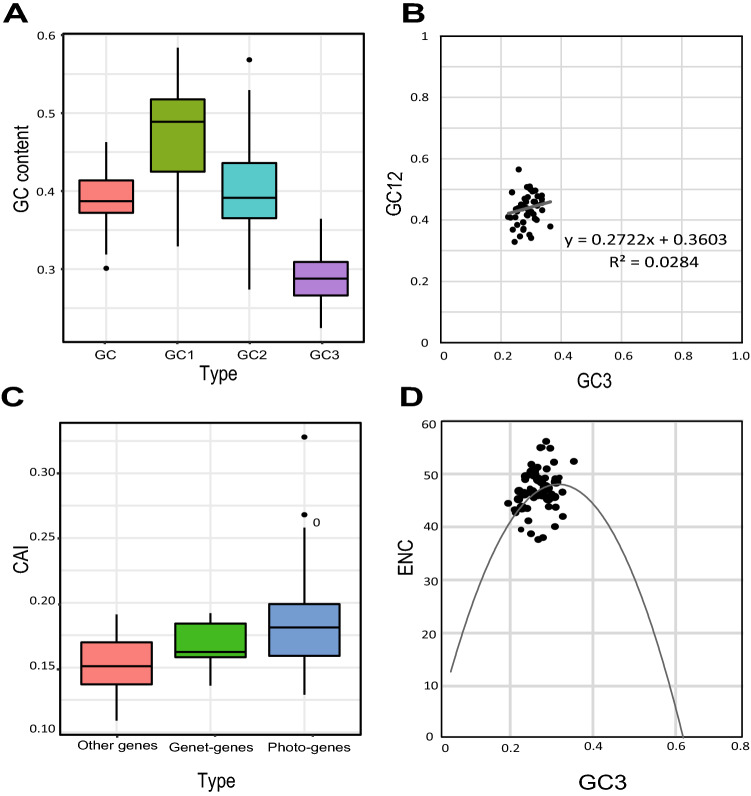
*Vasconcellea pubescens* chloroplast genome codon usage pattern related plot. (**A**) GC content of different codon sites. (**B**) Neutrality plot (GC12 against GC3). (**C**) The codon adaptation index (CAI) value of different function gene sets. (**D**) Relationship between GC3 and effective number of codons (ENC) (ENC-plot).

Codon usage bias (CUB) means different frequencies among synonymous codons. Analyzing CUB can help us to understand molecular evolution, environmental adaptation, and genomic features^59^. The chloroplast genome CUB is highly conserved and varies among different genes^60^. We explored the codon usage pattern of the chloroplast genome of *V. pubescens* and *C. papaya* using those protein-coding genes that have more than 300 nucleotides^61^. We first calculated relative synonymous codon usage (RSCU) value for each gene and marked codons with priority usage (RSCU > 1) with an asterisk (S2.7). We then investigated the base composition of each codon at each position in *V. pubescens*. The mean GC content was 47.4% in the first position, 40.2% in the second position, 28.7% in the third position, and 38.7% for all codons (Fig. 5A). The correlation between GC12 and GC3 was not significant (R^2^ = 0.0284), indicating that selective pressure does not affect the CUB in *V. pubescens* chloroplast genome (Fig. 5B). The codon adaptation index (CAI) is a measure of directional synonymous codon usage bias proposed by Paul et. al in 1987^60^. We investigated the CAI of each gene in *V. pubescens* chloroplast genome by dividing the genes into three types: photosynthesis-related genes (photo-genes), genetic system-related genes (Genet-genes), and other genes. The CAI of the photo-genes was higher than that of other genes (Fig. 5C), and the CAI of genetic genes was the lowest^58,61^. The effective number of codons (ENC) values are used to quantify how far codon usage of a gene departs from equal usage of synonymous codons^62^. The ENCs of *V. pubescens* chloroplast genes ranged from 38.36 to 56.88, which implies that CUB of genes is different and relatively weak (Fig. 5D). The relationship between base composition and ENC was investigated using ENC plots. Most genes were off the standard curve, indicating that no base composition was affected CUB.

### Structural haplotypes

**Figure 6:**
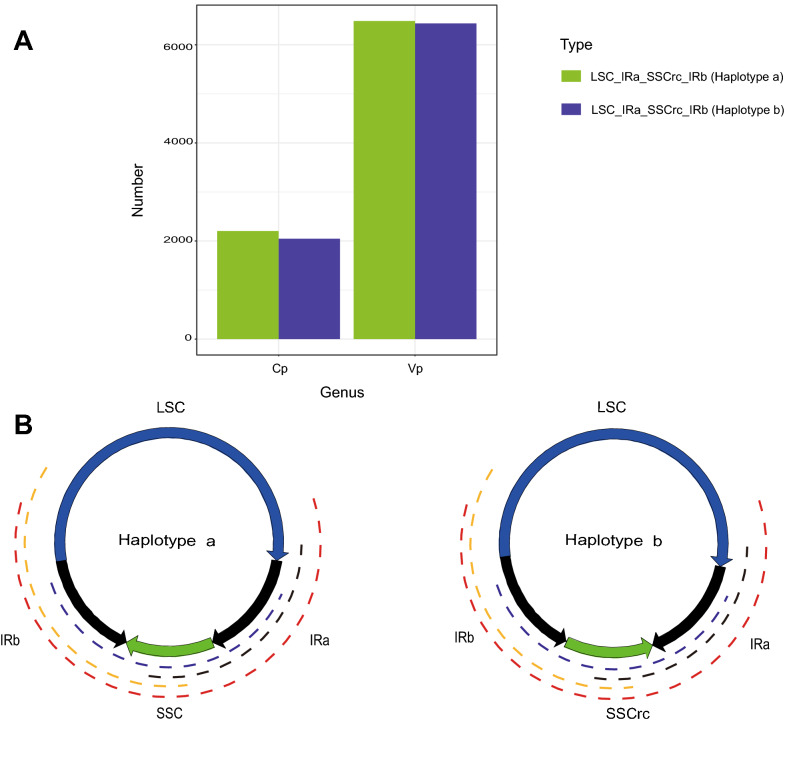
Stucture haplotypes plot. The number of Nanopore reads that supported the two structure haplotypes of *Carica papaya* and *Vasconcellea pubescens* chloroplast genome, respectively (**A**). Two different structure haplotypes of the chloroplast genome detected in this study, the dashed line with different colors illustrated Nanopore reads with different lengths which covered different regions of the two haplotypes chloroplast molecular (**B**).

We detected two haplotypes of the chloroplast genome from both *C. papaya* and *V. pubescens,* and the directions of the haplotypes are LSC_IRa_SSCrc_IRb and LSC_IRa_SSC_IRb (“rc” means reverse and complementary) (Fig. 6B). In *V. pubescens*, the ONT reads covering the LSC_IRa_SSCrc_IRb type were 6435 and the reads covering the LSC_IRa_SSC_IRb type were 6485. Meanwhile, in *C. papaya*, the numbers were 2047 and 2203, respectively (Fig. 6A). We further designed primers to verify the existence of the two types of chloroplast genome structures, PCR products of expected size were obtained. Sanger sequencing results were also confirmed that the PCR products either covering the end part of IR or the start part of SSC region or covering the end part of IR and start part of SSCrc region (S3). Using combination of the start part and the end part of the LSC region sequence (2371 bp) as a query to blast all the *V. pubescens* ONT reads, we detected one ONT read (we named it “ONT1”, 44,186 bp) that covered the end and start part of LSC sequence (S2.12). We then extracted and used the ONT1 sequence as a query to blast the *V. pubescens* chloroplast genome (S2.12), it does cover the start part of the LSC (from site 1 to site 16,440) and the end part of the LSC (from site 59,366 to site 87,980), and the LSC region start from 1 to 87,991. Based on these results, we conclude that there was a closed circle LSC form of chloroplast structure exist in the cell. However, the amount of this atypical type chloroplast DNA may be very small, since we only got one ONT read that covered the combined fragment. The other 505 blast hits represented ONT reads that covered only one of the two separate parts of the combined fragment or covered both but the ONT read represents the normal LSC fragment.

### Non-synonymous and synonymous rate of substitution

The evolutionary rates of all 78 protein coding genes were analyzed according to non-synonymous and synonymous rates of substitution (Ka and Ks). We generated 61 Ka/Ks ratio values (S2.8). The mean Ka/Ks ratio was 0.198, and most of the ratios were lower than 0.5, indicating purifying selection. The Ka/Ks of *Ycf2* gene, however, was greater than 1, indicating positive selection.

### *Ycf1* gene

The function of YCF1 remains unknown. We compared *Ycf1* gene sequences of *V. pubescens* and *C. papaya*, and designed primers to amplify *Ycf1* orthologous gene of several other Caricaceae species. Finally, we got all seven *Ycf1* gene sequences from four genera in family Caricaceae, and the four genera are *Jarilla, Carica, Vasconcella,* and *Jacaratia* (S2.9). Multiple alignment results showed that 5 prime (1–605 bp) and 3 prime regions (about 100 bp) of all *Ycf1* genes were nearly identical, except variable 3 prime regions. Using the TMHMM website to predict trans-membrane helices, we identified six trans-membrane helices in all YCF1 proteins, as previously reported (Fig. S7)^29^. We also found a motif RLEDLACMNRYW through MEME in 3 prime regions (about 100 bp), which is consistent with previously reported conserved motif in the carboxy terminus of YCF1 in Ste-YCF1^25^. We further investigated the indels (insertion-deletion mutations) and SNP distribution profile among all seven *Ycf1* genes, and all four genera’s *Ycf1* genes had their genus-specific indels and such situation was not found in the SNPs. Nine indels were detected and although the *Carica* genus has only one species, it contained five of the nine indels, including 4 insertions and 1 deletion (Table 2, S4). The genera *Vasconcella* and *Jacaratia* each had one genus-specific insertion, and the genus *Jarilla* had two genus-specific insertions. We then added Arabidopsis *Ycf1* gene sequence for indel analysis, and all insertions and deletions were genus-specific, occured after the divergence of Brassicaceae and Caricaceae (S5). We also selected eight *Ycf1* genes from three different genera in family Lauraceae, and alignment results did not show similar results (S6).

**Table 2:** Indels distribution of *Ycf1* genes from different genera in Caricaceae.

Genus name	Insertion	Deletion
*Carica*	4	1
*Vasconcella*	1	0
*Jacaratia*	1	0
*Jarilla*	2	0

The evolution of *Ycf1* gene was analyzed by calculating synonymous (Ks) and nonsynonymous (Ka) values of *Ycf1* sequences from seven species in four genera. The results showed that Ka/Ks < 1, indicating that the *Ycf1* was under purifying selection. We then examined site-specific evolution of the *Ycf1* gene, and only one locus (311 K 0.961*) was positively selected according to the M8 model. The phylogenetic tree of *Ycf1* genes shows that *Jacaratia spinosa* was close to the *Vasconcellea* genus (*V. pubescens* and *V. monoica*), while *C. papaya* was close to *Jarilla* genus (*Jarilla heterophella, Jarilla chocola and Jarilla caudata*) (Fig. S8).

The photosystem biogenesis regulator 1 (PBR1) strongly regulates the expression of the chloroplast gene *Ycf1* by binding to the 5′UTR of the chloroplast gene *Ycf1* mRNA in Arabidopsis. We also compared the 5′UTR of *V. pubescens*, *C. papaya*, and *A. thaliana Ycf1* genes. All 5′UTR sequences have the same size (73 bp), with only one difference detected in the position 43 where *V. pubescens* and *C. papaya* have C base while *A. thaliana* has T base.

## Discussion

The chloroplast genome of highland papaya *V. pubescens* was sequenced and assembled using Nanopore long reads combined with Illumina short reads. This long read sequences greatly facilitated the assembly of the chloroplast genome, and also revealed structural variations of the chloroplast genome. Our assembly results showed that two unitigs were sufficient to produce the whole circular chloroplast genome when assembled using the Smartdenovo soft. Meanwhile, we also tested the newly published NECAT2 software, which produced only one contig that covered almost the whole *V. pubescens* chloroplast genome (99% similar to the final version *V. pubescens* chloroplast genome, after polishing with the NGS data). All results showed advantage of long reads. Two haplotypes, LSC_IRa_SSCrc_IRb and LSC_IRa_SSC_IRb, were detected in both *V. pubescens* and *C. papaya*. Though different versions of the chloroplast genome structure of some plants have been reported recently^9^, there is no previous report in the Caricaceae family. The change of the chloroplast structures was assumed to be the result of flipped recombination^9,64,65^. We also found one over 40 kb ONT read that covered the beginning segment and ending segment of the LSC region. The amount of circular type LSC maybe very small, since there is only one ONT read among the 506 ONT reads matching the complete query sequence in the same direction. Chloroplast structure plasticity was reported in higher plants and chloroplast DNA without IRs was also found^8^. Our results further confirmed the structure plasticity of the chloroplast.

The chloroplast genome of *V. pubescens* was smaller than that of *C. papaya,* but its IR regions was longer than *C. papaya*. Unequal recombination and replication slippage can result in expansion and contraction of the IR regions, leading to variations in chloroplast genome size^59,63,64^. Five highly variable regions were identified in the *V. pubescens* and *C. papaya* chloroplast genomes, which are *psbZ*, *trnG-GCC*, *trnH-GUG* and *psbA*, *trnS-GCU* and *trnG-UCC*, and *rps16*. These variable sequences between the two chloroplast genomes can be used to develop molecular markers that have been widely used for new species identification in other plant lineages^53,59,65–67^.

RNA editing is an important tool for gene expression regulation, and most RNA editing events in chloroplast genomes are converting C to U^15,54,55^. Members of the pentatricopeptide repeat (PPR) protein family play an important role in RNA editing in chloroplast genomes, which has been reported in Arabidopsis, rice, and maize^14,68–72^. Prediction of RNA editing loci in chloroplasts helps us to understand chloroplast regulatory mechanisms and design strategies for chloroplast genome engineering. With PREP prediction, we detected 52 RNA editing loci in 18 protein-coding genes in *V. pubescens* and *C. papaya*. We used RNA-seq data from *C. papaya* leaves for variant calling, and identified 46 RNA editing loci located in 30 genes and 3 intergenic regions of the chloroplast genome. Some of these loci were verified by Sanger sequencing. RNA editing frequently occurs in gene-encoding components of the NDH complex, particularly *ndhD* gene. This is similar to the *Ginkgo biloba* chloroplast genome, supporting the hypothesis that the highest RNA editing events occur in the NDH complex genes in seed plants^17,73,74^. RNA editing efficiency were also calculated which showed differences across all genes with an average efficiency of 63%. RNA editing efficiency in *Spirodela polyrhiza* averaged 76%, higher than that in *C. papaya*^17^. The changes in RNA editing efficiency on genes or at different loci of the same gene imply complex RNA editing regulatory mechanisms, which need to be explored. RNA editing could cause protein structure change and regulate the photosynthesis process^18,73^. Therefore, the detailed analysis of RNA editing loci in the chloroplast genome is quite necessary.

*Ycf1* genes were amplified from seven species in four genera of the Caricaceae family. The N terminal (605 bp) and C terminal (100 bp) of the *Ycf1* genes are nearly identical, which enables us to amplify *Ycf1* from different species, and also implies the evolutionary conservation of both regions of *Ycf1* genes in Caricaceae family, whereas the other regions of the *Ycf1* genes showed very high variation, making them good candidates for barcode development. All seven *Ycf1* genes from different genera had genus-specific indels. These results implied that indels may play an important role in genus-specific evolution of *Ycf1* genes in Caricaceae. Six trans-membrane helices were found as previously reported^3^. Analysis of full length *Ycf1* genes showed that the *Ycf1* genes were under purification selection, whereas site-specific evolution analysis results showed that position 311 K was under positive selection with a *p* < 0.05. This suggests that a specific locus of the gene might evolve independently under a specific driving force, or the 311 K locus might be an important boundary for YCF1.

## Supplementary information

Supplementary Information 1.

Supplementary Information 2.

Supplementary Information 3.

Supplementary Information 4.

Supplementary Information 5.

Supplementary Information 6.

## Data Availability

Raw data of high-throughput sequencing and Oxford Nanopore sequencing were deposited in NCBI SRA. (SRA accession: PRJNA605960, https://www.ncbi.nlm.nih.gov/sra/PRJNA605960).
