# Supplementary material for: Comparative analysis of chloroplast genomes in Vasconcellea pubescens A.DC. and Carica papaya L
Source: Sci Rep. 2020 Sep 25;10:15799. doi: 10.1038/s41598-020-72769-y (PMC7519098; doi:10.1038/s41598-020-72769-y)
Supplement: Supplementary file 1 — Supplementary Information 1. [file 41598_2020_72769_MOESM1_ESM.pdf]

Comparative analysis of chloroplast genomes in *Vasconcellea pubescens* A.DC. and *Carica papaya* L.

Zhicong Lin[1] , Ping Zhou[3], Xinyi Ma[2], Youjin Deng[2], Zhenyang Liao[2],

Ruoyu Li[2] and Ray Ming[4,1] *

[1]College of Agriculture, Center for Genomics and Biotechnology, Fujian Provincial Key Laboratory of Haixia Applied Plant Systems Biology, Fujian Agriculture and Forestry University, Fuzhou, Fujian 350002, China.

[2]College of Life Sciences, Fujian Agriculture and Forestry University, Fuzhou 350002, Fujian, China.

[3]Fruit Research Institute，Fujian Academy of Agricultural Sciences，Fuzhou 350013，Fujian, China

[4]Department of Plant Biology, University of Illinois at Urbana-Champaign, Urbana, IL 61801, USA.

*rayming@illinois.edu

Supplementary Figures

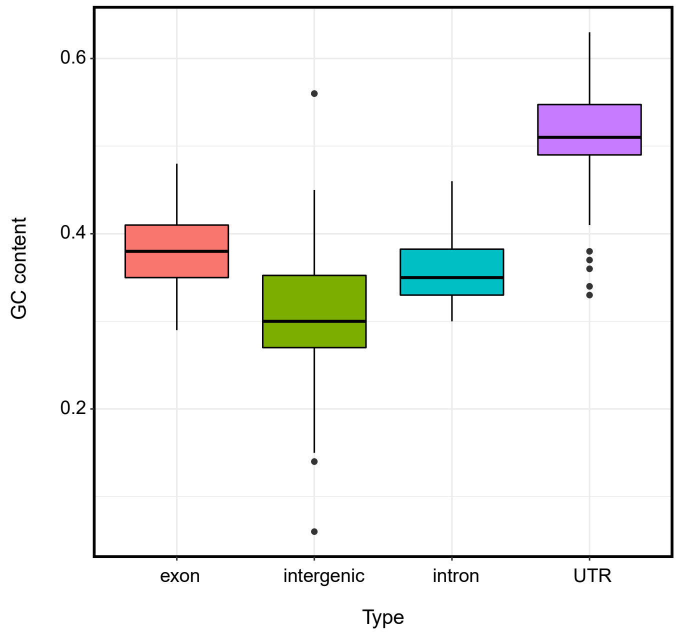

Fig. S1 GC content of *V. pubscens* chloroplast in region exon, intron, UTR and intergenic.

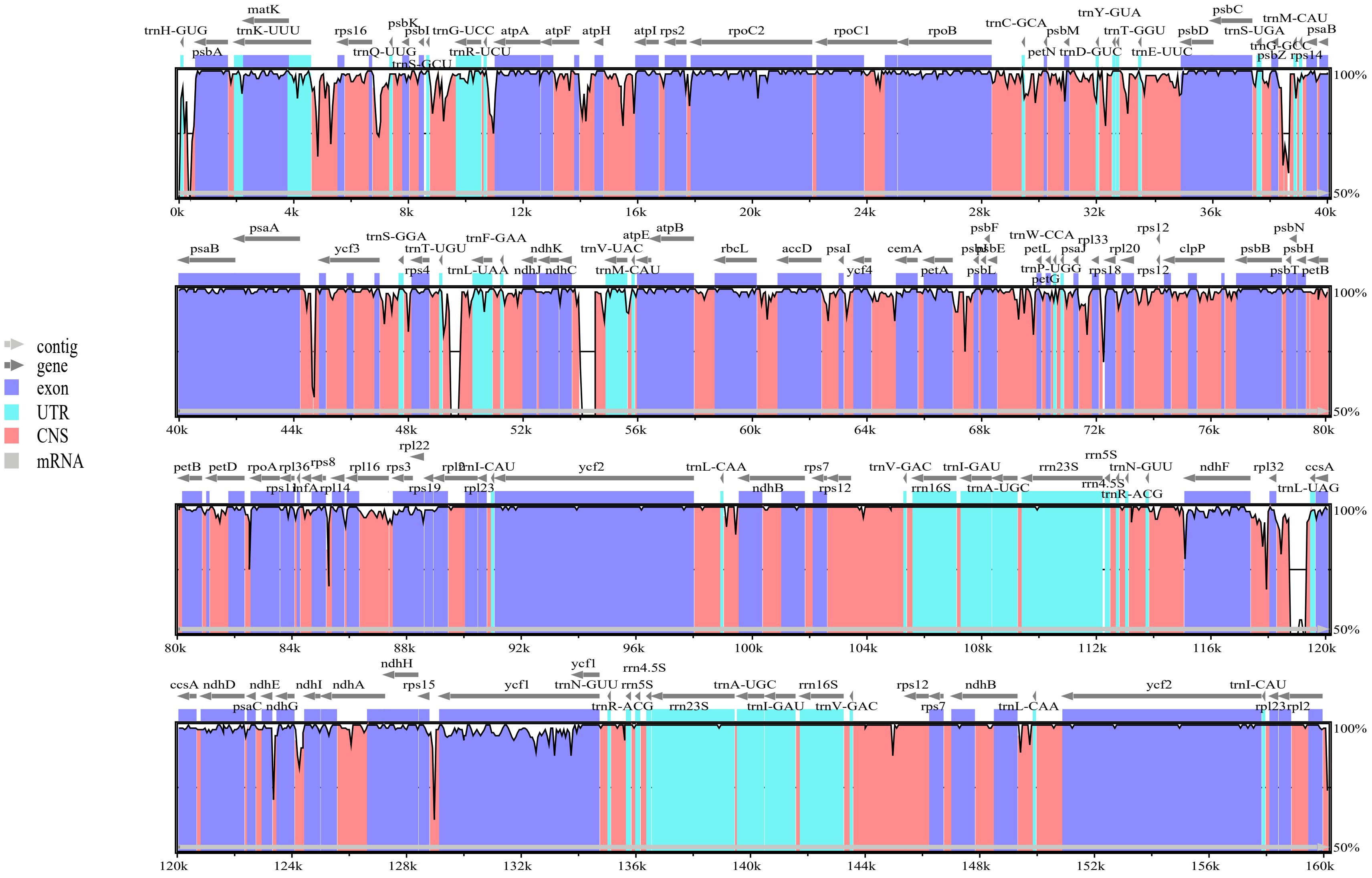

Fig. S2 Visual alignment of *V. pubescens* and *C. papaya* chloroplast genome, *C. papaya* chloroplast genome sequence as a reference.

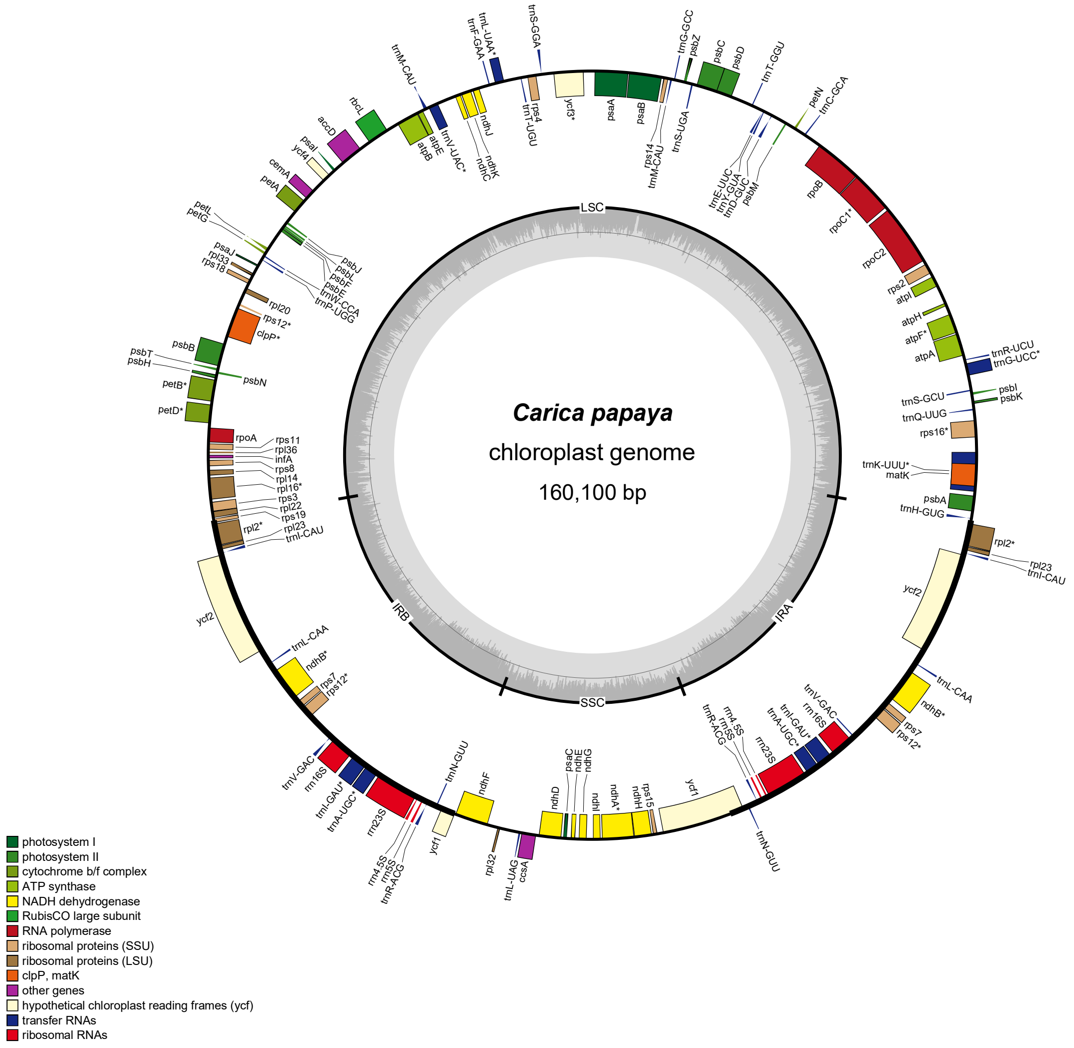

Fig. S3 *C. papaya* chloroplast gene map

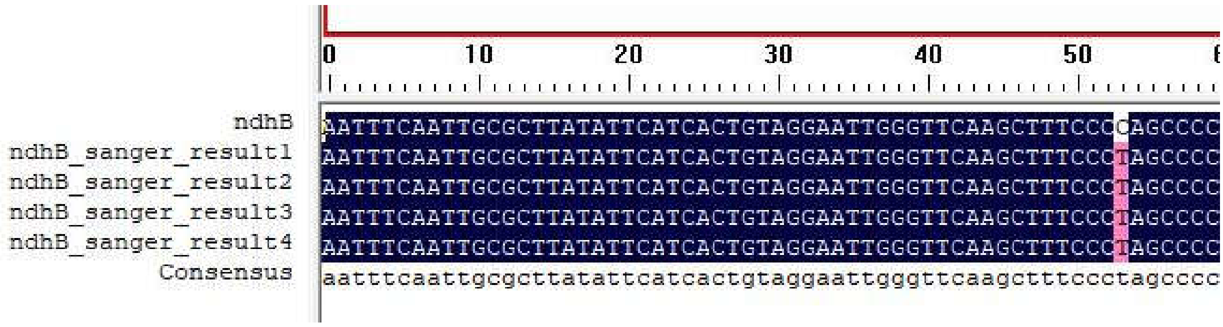

Fig. S4 *ndhB*_editing site (Pos 147,769) sanger sequencing result (*C. papaya*)

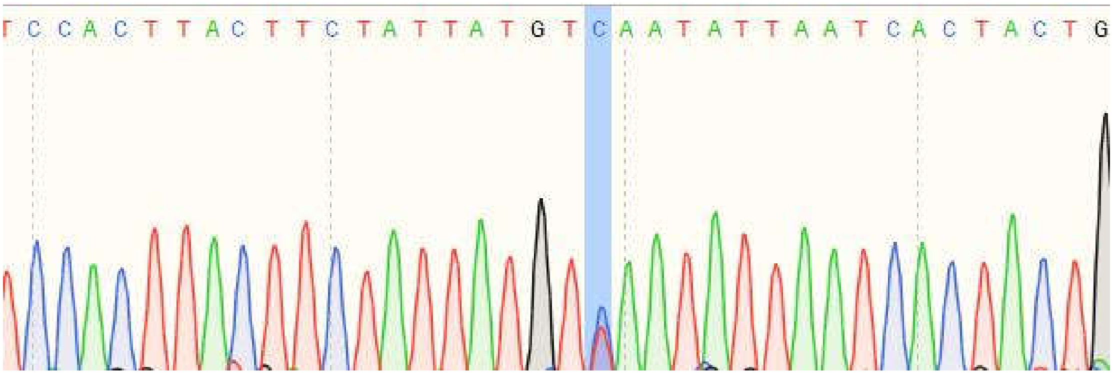

Fig. S5 *ndhF*_editing site (Pos 117,084) sanger sequencing result (*C. papaya*)

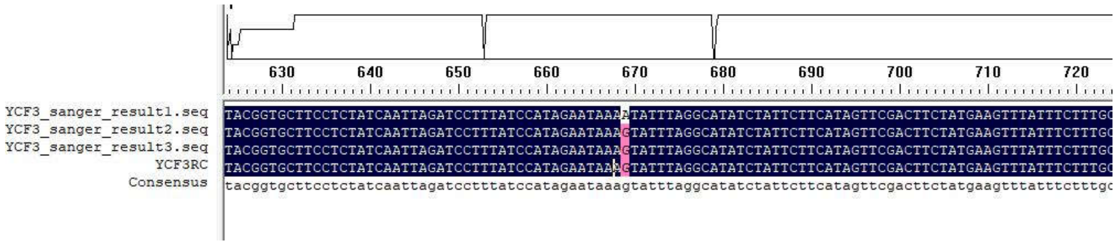

Fig. S6 *Ycf3*_editing site (Pos 45,689) sanger sequencing result (*C. papaya*)

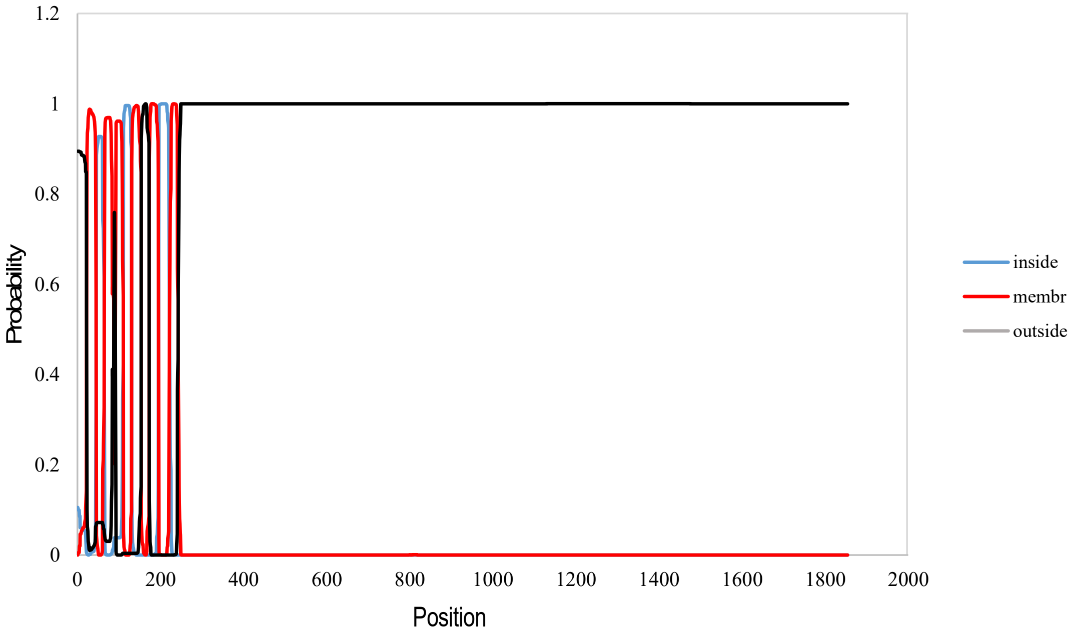

Fig. S7 *V. pubescens* TMHMM prediction result

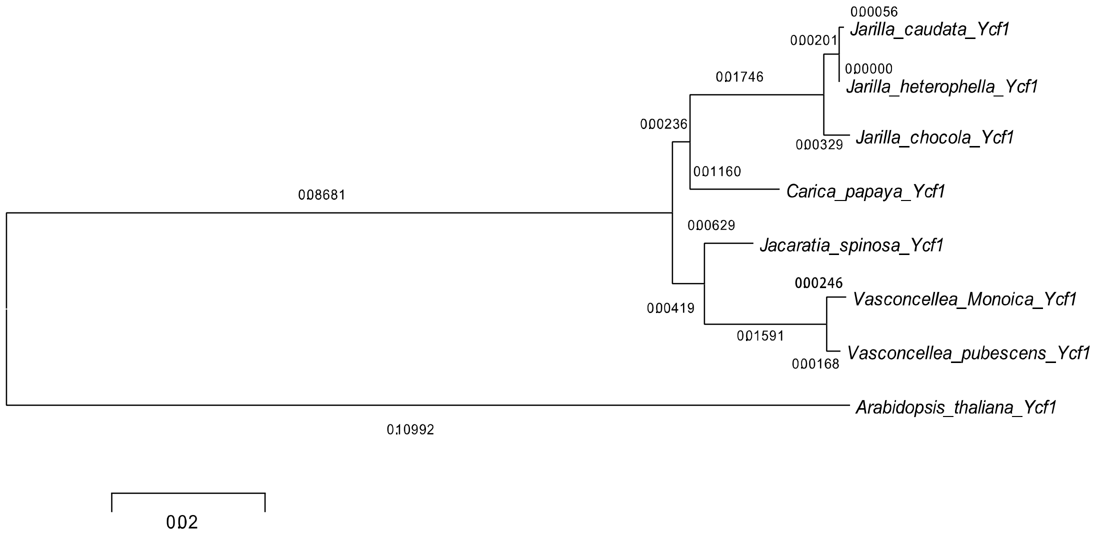

Fig. S8 Phylogenetic tree of seven species of Caricaceae family using *Ycf1* gene nucleotide sequences, maximum likehood method.
