# Supplementary material for: Comparative analysis of chloroplast genomes in Vasconcellea pubescens A.DC. and Carica papaya L
Source: Sci Rep. 2020 Sep 25;10:15799. doi: 10.1038/s41598-020-72769-y (PMC7519098; doi:10.1038/s41598-020-72769-y)

Comparative analysis of chloroplast genomes in *Vasconcellea pubescens* A.DC. and *Carica papaya* L.

Zhicong Lin[1] , Ping Zhou[3], Xinyi Ma[2], Youjin Deng[2], Zhenyang Liao[2],

Ruoyu Li[2] and Ray Ming[4,1] *

[1]College of Agriculture, Center for Genomics and Biotechnology, Fujian Provincial Key Laboratory of Haixia Applied Plant Systems Biology, Fujian Agriculture and Forestry University, Fuzhou, Fujian 350002, China.

[2]College of Life Sciences, Fujian Agriculture and Forestry University, Fuzhou 350002, Fujian, China.

[3]Fruit Research Institute，Fujian Academy of Agricultural Sciences，Fuzhou 350013，Fujian, China

[4]Department of Plant Biology, University of Illinois at Urbana-Champaign, Urbana, IL 61801, USA.

*rayming@illinois.edu

Structure haplotype verified results

Note: Yellow part means IRa region (end part), green part means SSC or SSCrc region (start part). The sequences marked with red and underline were primers used for amplified the target region. The black part in the chromatogram represents the IRa part in the junction sequence of IRa and SSC region.

1）*Carica papaya* two structure haplotypes Sanger sequencing results

LSC+IRa+SSC+IRb:

>IRa+SSC

GTCAACATTTATATGTTTCGATGCAACAACAAGATGTTATTTGTAACAAGTAGTTTTGTTGGTTGGTTAATTGG
TCACATTTTATTCATGAAAT**GGGTTGGATTGGTATTAGTC**TGGATACAGCAAAATAATTCTATTAGGTCTAATG
TACTTATTCGATCTAATAAGTATAAGTACCTTGTGTCAGAATTGAGAAATTCTATGGCTCGAATCTTTAGTATTC
TCTTATTTATTACCTGTGTCTACTATTTAGGCAGAATACCGTCACCCATTTTTACTAAGAAACTAAAAGGAACCT
CGGAAACGGAGGAAAGGGGGGGGACTAAACAGGACCAAGAGGTATCCACCGAAGAAGATCCTTTTCCTT
CTCTTTTTTCGGAAGAAAGGGAGGATCCGGACAAAATCGATGAAACGGAAGAGATCCGAGTGAATGGAAA
GGAAAAAAAAAATAAGGATGATGAATTTAAATTTAAAATAAAATTTAATTAAAATTAATAAAATTAAAGTGAA
AGATTCTTCTTAGATTAGTAGATTAGAAGAAATTCAATAAATTTAAAAAGTAGTAAATAAATAAAAAAATTAAT
ACATAGAACAAATACAATAATAGATAAGAAGAGATGCGACTTCCCCCGACATATTTTATGCCTTCTCCTATAAA
GAAACTCGTAATACCTACCCCATTTGTAATTCCATCAATTACTCGTCTATCAAAAAAATGAGTTAGTTCCGCTAA
TCCTCTTATACCCCCAGTTAAAGATATTGTATAAAAAGTAT**CTATGTAACCACGATTATACGACC**AATCATATATA
AAATTGATTATTTTGTCCCACAGAATTCTCTTAGGACCCCTTTTTGCAAATGAATTAAGTAAGTTTAAATTCTG

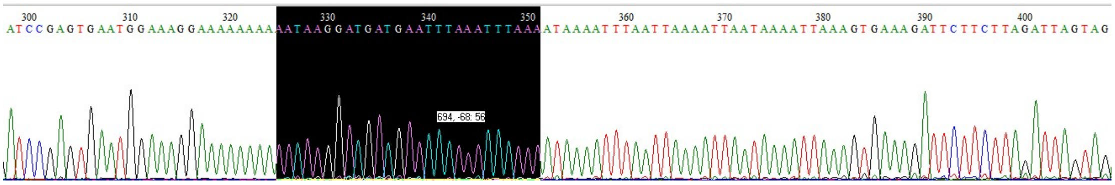

LSC+IRa+SSCrc+IRb:

>IRa+SSCrc

ATGTGTATTCCTGAATAATCTCATTTTTCAATTATTCAACCATTTCATTTTACCAAGTTCAATGTTAGCCAGATTA
GTCAACATTTATATGTTTCGATGCAACAACAAGATGTTATTTGTAACAAGTAGTTTTGTTGGTTGGTTAATTGG
TCACATTTTATTCATGAAAT**GGGTTGGATTGGTATTAGTC**TGGATACAGCAAAATAATTCTATTAGGTCTAATG
TACTTATTCGATCTAATAAGTATAAGTACCTTGTGTCAGAATTGAGAAATTCTATGGCTCGAATCTTTAGTATTC
TCTTATTTATTACCTGTGTCTACTATTTAGGCAGAATACCGTCACCCATTTTTACTAAGAAACTAAAAGGAACCT
CGGAAACGGAGGAAAGGGGGGGGACTAAACAGGACCAAGAGGTATCCACCGAAGAAGATCCTTTTCCTT
CTCTTTTTTCGGAAGAAAGGGAGGATCCGGACAAAATCGATGAAACGGAAGAGATCCGAGTGAATGGAAA
GGAAAAAAAAAATAAGGATGATGAATTTAAATTTAAAGAGATATCCTCTAACTTTTATAACTTTAAATATAAAA
ATAGGCTAGGTTATGAAAATCTAGATGAGAATCAAGAAAATTCCAATTTAGAAATATTAAAAAGAAAAGAAG
ATAAATATTTATTATGGTTTGAAAAACCTTTTGTGACTCTTCTTTTTGATTATAAA**CGGTGGAATCGGCCATTTC**
GATATCTAAAAAATGATCGATTTGAAAATGCTGTAAGAAATCAAATGTCACAATATTTTTTTATACATGTCAAA

GTGATGGAAAAGAAAGAATATCTTTTACGTATCCACCCAGTTTGGCAACTTTTTTGGAAATGATACAACAAA

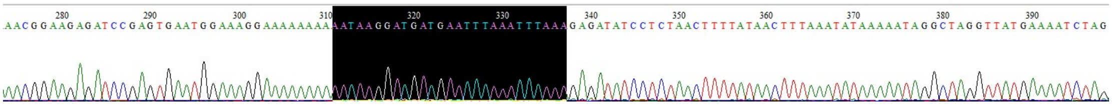

2）*Vasconcellea pubescens* two structure haplotypes verified result

LSC+IRa+SSC+IRb:

>IRa+SSC

GGCAGAATACCGTCACCCATTTTTACTAAGAAAATAAAA**GGAATCTCAGAAACGGAGG**AAAGTGGGGGGA
CTAAACAGGACCAAGAGGTATCCACCGAAGAAGATCCTTTTCCTTCTCTTTTTTCGGAAGAAAGGGAGGAT
CCGGACAAAATCGATGAAACGGAAGAGATCCGAGTAAATGGAAAGGAAAAAAAAAAATAAAGATGATGAAT
TTCTCTTTACAGAGACATCCTCTAACTTAAAATATAAAAATAGGCTAGTTTATGAAAATCTAGATGAGAATCAA
GAAAATTCCAATTTCGAAATATTAAAAAGAAAAGAAGATAAATATTTTTTAAAATTAAAATTAAAGATTCTTCT
TAGATCTTAGATTAGACGAAATTCAATAAATTTGAAAAGTAGTAAATTAATAAAAAAATTAATACATAGAACAA
ATACAATAATAAATAAGAAGAGATGCGACTTCCCCCGACATATTTTATGCCTTCCCCTATAAAGAAACTCGTAA
TACCTACCCCATTTGTAATTCCATCAATTACTCGTCTATCAAAAAAAT**GAGTTAGTTCTGCTAATCCTC**TTATAC
CCCCAGTTAAAGATATTCTATAAAAAGTATCTATGTAACCACGATTATACGACCAAGCATATATCAAATTTATT

The black part was reverse complementary sequence of IRa part.

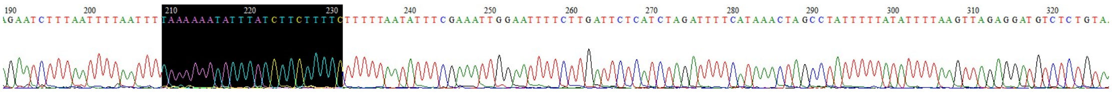

LSC+IRa+SSCrc+IRb:

>IRa+SSCrc

GGCAGAATACCGTCACCCATTTTTACTAAGAAAATAAAA**GGAATCTCAGAAACGGAGG**AAAGTGGGGGGA
CTAAACAGGACCAAGAGGTATCCACCGAAGAAGATCCTTTTCCTTCTCTTTTTTCGGAAGAAAGGGAGGAT
CCGGACAAAATCGATGAAACGGAAGAGATCCGAGTAAATGGAAAGGAAAAAAAAAAATAAAGATGATGAAT
TTCTCTTTACAGAGACATCCTCTAACTTAAAATATAAAAATAGGCTAGTTTATGAAAATCTAGATGAGAATCAA
GAAAATTCCAATTTCGAAATATTAAAAAGAAAAGAAGATAAATATTTTTTATGGTTTGAAAAACCGTTTGTGA
CTCTTCTTTTTGATTATAAACGGTGGAATCGGCCATTTCGATATATAAAAAATGATCAGTTTGAAAATGCTATAA
GAAATAAAATGTCACAATATTTTTTTTATACATGTCAAAGTGATGGAAAAGAACGAATATCTTTTA**CGTATCCA
CCCAGTTTGGCAAC**TTTTTTGGAACTGATACAAAAAAA

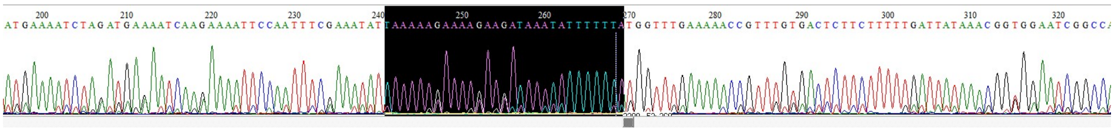

Supplement: Supplementary file 3 — Supplementary Information 3. [file 41598_2020_72769_MOESM3_ESM.pdf]
