# Supplementary material for: Comparative analysis of chloroplast genomes in Vasconcellea pubescens A.DC. and Carica papaya L
Source: Sci Rep. 2020 Sep 25;10:15799. doi: 10.1038/s41598-020-72769-y (PMC7519098; doi:10.1038/s41598-020-72769-y)
Supplement: Supplementary file 4 — Supplementary Information 4. [file 41598_2020_72769_MOESM4_ESM.pdf]

Comparative analysis of chloroplast genomes in *Vasconcellea pubescens* A.DC. and *Carica papaya* L.

Zhicong Lin[1] , Ping Zhou[3], Xinyi Ma[2], Youjin Deng[2], Zhenyang Liao[2],

Ruoyu Li[2] and Ray Ming[4,1 *]

[1]College of Agriculture, Center for Genomics and Biotechnology, Fujian Provincial Key Laboratory of Haixia Applied Plant Systems Biology, Fujian Agriculture and Forestry University, Fuzhou, Fujian 350002, China.

[2]College of Life Sciences, Fujian Agriculture and Forestry University, Fuzhou 350002, Fujian, China.

[3]Fruit Research Institute，Fujian Academy of Agricultural Sciences，Fuzhou 350013，Fujian, China

[4]Department of Plant Biology, University of Illinois at Urbana-Champaign, Urbana, IL 61801, USA.

[*]rayming@illinois.edu

Indels and SNPs profile of *Ycf1* gene in four genera of family Caricaceae

Note: Indels specific to each genus were marked with different colors

	....|....|	....|....|	....|....|	....|....|	....|....|	....|....|
	5	15	25	35	45	55
Carica_papaya_Ycf1	ATGATTTTTA	AATCTTTTAT	ACTAGGTAAT	CTAGTATCCT	TATGCATGAA	GATAATCAAT
Vasconcellea_Monoica_Ycf1	ATGATTTTTA	AATCTTTTAT	ACTAGGTAAT	CTAGTATCCT	TATGCATGAA	GATAATCAAT
Jacaratia_spinosa_Ycf1	ATGATTTTTA	AATCTTTTAT	ACTAGGTAAT	CTAGTATCCT	TATGCATGAA	GATAATCAAT
Vasconcellea_pubescens_Ycf1	ATGATTTTTA	AATCTTTTAT	ACTAGGTAAT	CTAGTATCCT	TATGCATGAA	GATAATCAAT
Jarilla_caudata_Ycf1	ATGATTTTTA	AATCTTTTAT	ACTAGGTAAT	CTAGTATCCT	TATGCATGAA	GATAATCAAT
Jarilla_chocola_Ycf1	ATGATTTTTA	AATCTTTTAT	ACTAGGTAAT	CTAGTATCCT	TATGCATGAA	GATAATCAAT
Jarilla_heterophella_Ycf1	ATGATTTTTA	AATCTTTTAT	ACTAGGTAAT	CTAGTATCCT	TATGCATGAA	GATAATCAAT

	....|....|	....|....|	....|....|	....|....|	....|....|	....|....|
	65	75	85	95	105	115
Carica_papaya_Ycf1	TCGGTCGTTG	TGGTCGGACT	CTATTATGGA	TTTCTGACCA	CATTCTCCGT	AGGGCCCTCT
Vasconcellea_Monoica_Ycf1	TCGGTCGTTG	TGGTCGGACT	CTATTATGGA	TTTCTGACCA	CATTCTCCAT	AGGGCCCTCT
Jacaratia_spinosa_Ycf1	TCGGTCGTTG	TGGTCGGACT	CTATTATGGA	TTTCTGACCA	CATTCTCCAT	AGGGCCCTCT
Vasconcellea_pubescens_Ycf1	TCGGTCGTTG	TGGTCGGACT	CTATTATGGA	TTTCTGACCA	CATTCTCCAT	AGGGCCCTCT
Jarilla_caudata_Ycf1	TCGGTCGTTG	TGGTCGGACT	CTATTATGGA	TTTCTGACCA	CATTCTCCAT	AGGGCCCTCT
Jarilla_chocola_Ycf1	TCGGTCGTTG	TGGTCGGACT	CTATTATGGA	TTTCTGACCA	CATTCTCCAT	AGGGCCCTCT
Jarilla_heterophella_Ycf1	TCGGTCGTTG	TGGTCGGACT	CTATTATGGA	TTTCTGACCA	CATTCTCCAT	AGGGCCCTCT

	....|....|	....|....|	....|....|	....|....|	....|....|	....|....|
	125	135	145	155	165	175
Carica_papaya_Ycf1	TATCTCTTTC	TTCTCCGAGC	TCGGGTTATG	GAAGAAGGAG	AAGAAGGAAC	CGAGAAGAAG
Vasconcellea_Monoica_Ycf1	TATCTCTTTC	TTCTCCGAGC	TCGGGTTATG	GAAGAAGGAG	AAGAAGGAAC	CGAGAAGAAG
Jacaratia_spinosa_Ycf1	TATCTCTTTC	TTCTCCGAGC	TCGGGTTATG	GAAGAAGGAG	AAGAAGGAAC	CGAGAAGAAG
Vasconcellea_pubescens_Ycf1	TATCTCTTTC	TTCTCCGAGC	TCGGGTTATG	GAAGAAGGAG	AAGAAGGAAC	CGAGAAGAAG
Jarilla_caudata_Ycf1	TATCTCTTTC	TTCTCCGAGC	TCGGGTTATG	GAAGAAGGAG	AAGAAGGAAC	CGAGAAGAAG
Jarilla_chocola_Ycf1	TATCTCTTTC	TTCTCCGAGC	TCGGGTTATG	GAAGAAGGAG	AAGAAGGAAC	CGAGAAGAAG
Jarilla_heterophella_Ycf1	TATCTCTTTC	TTCTCCGAGC	TCGGGTTATG	GAAGAAGGAG	AAGAAGGAAC	CGAGAAGAAG

	....|....|	....|....|	....|....|	....|....|	....|....|	....|....|
	185	195	205	215	225	235
Carica_papaya_Ycf1	GTATCAGCAA	CAACTGGTTT	TATTGCGGGA	CAGCTCATGA	TGTTCATATC	GATCTATTAT
Vasconcellea_Monoica_Ycf1	GTATCAGCAA	CAACTGGTTT	TATTGCGGGA	CAGCTCATGA	TGTTCATATC	GATCTATTAT
Jacaratia_spinosa_Ycf1	GTATCAGCAA	CAACTGGTTT	TATTGCGGGA	CAGCTCATGA	TGTTCATATC	GATCTATTAT
Vasconcellea_pubescens_Ycf1	GTATCAGCAA	CAACTGGTTT	TATTGCGGGA	CAGCTCATGA	TGTTCATATC	GATCTATTAT
Jarilla_caudata_Ycf1	GTATCAGCAA	CAACTGGTTT	TATTGCGGGA	CAGCTCATGA	TGTTCATATC	GATCTATTAT
Jarilla_chocola_Ycf1	GTATCAGCAA	CAACTGGTTT	TATTGCGGGA	CAGCTCATGA	TGTTCATATC	GATCTATTAT
Jarilla_heterophella_Ycf1	GTATCAGCAA	CAACTGGTTT	TATTGCGGGA	CAGCTCATGA	TGTTCATATC	GATCTATTAT

	....|....| ....|....| ....|....| ....|....| ....|....| ....|....|
	245 255 265 275 285 295
Carica_papaya_Ycf1	GCGCCTCTGC ATTTAGCATT GGGTAGACCT CATACAATAA CTGTCCTAGC TCTACCGTAT
Vasconcellea_Monoica_Ycf1	GCGCCTCTGC ATTTAGCATT GGGTAGACCT CATACAATAA CTGTCCTAGC TCTACCGTAT
Jacaratia_spinosa_Ycf1	GCGCCTCTGC ATTTAGCATT GGGTAGACCT CATACAATAA CTGTCCTAGC TCTACCGTAT
Vasconcellea_pubescens_Ycf1	GCGCCTCTGC ATTTAGCATT GGGTAGACCT CATACAATAA CTGTCCTAGC TCTACCGTAT
Jarilla_caudata_Ycf1	GCGCCTCTGC ATTTAGCATT GGGTAGACCT CATACAATAA CTGTCCTAGC TCTACCGTAT
Jarilla_chocola_Ycf1	GCGCCTCTGC ATTTAGCATT GGGTAGACCT CATACAATAA CTGTCCTAGC TCTACCGTAT
Jarilla_heterophella_Ycf1	GCGCCTCTGC ATTTAGCATT GGGTAGACCT CATACAATAA CTGTCCTAGC TCTACCGTAT
	....|....| ....|....| ....|....| ....|....| ....|....| ....|....|
	305 315 325 335 345 355
Carica_papaya_Ycf1	CTTTTGTTTC ATTTCTTCTG GAACAATCAC AAACACTTTT TTGATTATGG ATCTACTACC
Vasconcellea_Monoica_Ycf1	CTTTTGTTTC ATTTCTTCTG GAACAATCAC AAACACTTTT TTGATTATGG ATCTACTACC
Jacaratia_spinosa_Ycf1	CTTTTGTTTC ATTTCTTCTG GAACAATCAC AAACACTTTT TTGATTATGG ATCTACTACC
Vasconcellea_pubescens_Ycf1	CTTTTGTTTC ATTTCTTCTG GAACAATCAC AAACACTTTT TTGATTATGG ATCTACTACC
Jarilla_caudata_Ycf1	CTTTTGTTTC ATTTCTTCTG GAACAATCAC AAACACTTTT TTGATTATGG ATCTACTACC
Jarilla_chocola_Ycf1	CTTTTGTTTC ATTTCTTCTG GAACAATCAC AAACACTTTT TTGATTATGG ATCTACTACC
Jarilla_heterophella_Ycf1	CTTTTGTTTC ATTTCTTCTG GAACAATCAC AAACACTTTT TTGATTATGG ATCTACTACC
	....|....| ....|....| ....|....| ....|....| ....|....| ....|....|
	365 375 385 395 405 415
Carica_papaya_Ycf1	AGAAATTCAA TGCGTAATCT TCGCATTCAA TGTGTATTCC TGAATAATCT CATTTTTCAA
Vasconcellea_Monoica_Ycf1	AGAAATTCAA TGCGTAATCT TCGCATTCAA TGTGTATTCC TGAATAATCT CATTTTTCAA
Jacaratia_spinosa_Ycf1	AGAAATTCAA TGCGTAATCT TCGCATTCAA TGTGTATTCC TGAATAATCT CATTTTTCAA
Vasconcellea_pubescens_Ycf1	AGAAATTCAA TGCGTAATCT TCGCATTCAA TGTGTATTCC TGAATAATCT CATTTTTCAA
Jarilla_caudata_Ycf1	AGAAATTCAA TGCGTAATCT TCGCATTCAA TGTGTATTCC TGAATAATCT CATTTTTCAA
Jarilla_chocola_Ycf1	AGAAATTCAA TGCGTAATCT TCGCATTCAA TGTGTATTCC TGAATAATCT CATTTTTCAA
Jarilla_heterophella_Ycf1	AGAAATTCAA TGCGTAATCT TCGCATTCAA TGTGTATTCC TGAATAATCT CATTTTTCAA
	....|....| ....|....| ....|....| ....|....| ....|....| ....|....|
	425 435 445 455 465 475
Carica_papaya_Ycf1	TTATTCAACC ATTTCATTTT ACCAAGTTCA ATGTTAGCCA GATTAGTCAA CATTTATATG
Vasconcellea_Monoica_Ycf1	TTATTCAACC ATTTCATTTT ACCAAGTTCA ATGTTAGCCA GATTAGTCAA CATTTATATG
Jacaratia_spinosa_Ycf1	TTATTCAACC ATTTCATTTT ACCAAGTTCA ATGTTAGCCA GATTAGTCAA CATTTATATG
Vasconcellea_pubescens_Ycf1	TTATTCAACC ATTTCATTTT ACCAAGTTCA ATGTTAGCCA GATTAGTCAA CATTTATATG
Jarilla_caudata_Ycf1	TTATTCAACC ATTTCATTTT ACCAAGTTCA ATGTTAGCCA GATTAGTCAA CATTTATATG
Jarilla_chocola_Ycf1	TTATTCAACC ATTTCATTTT ACCAAGTTCA ATGTTAGCCA GATTAGTCAA CATTTATATG
Jarilla_heterophella_Ycf1	TTATTCAACC ATTTCATTTT ACCAAGTTCA ATGTTAGCCA GATTAGTCAA CATTTATATG
	....|....| ....|....| ....|....| ....|....| ....|....| ....|....|
	485 495 505 515 525 535
Carica_papaya_Ycf1	TTTCGATGCA ACAACAAGAT GTTATTTGTA ACAAGTAGTT TTGTTGGTTG GTTAATTGGT
Vasconcellea_Monoica_Ycf1	TTTCGATGCA ACAACAAGAT GTTATTTGTA ACAAGTAGTT TTGTTGGTTG GTTAATTGGT

Jacaratia_spinosa_Ycf1 TTTCGATGCA ACAACAAGAT GTTATTTGTA ACAAGTAGTT TTGTTGGTTG GTTAATTGGT

Vasconcellea_pubescens_Ycf1 TTTCGATGCA ACAACAAGAT GTTATTTGTA ACAAGTAGTT TTGTTGGTTG GTTAATTGGT

Jarilla_caudata_Ycf1 TTTCGATGCA ACAACAAGAT GTTATTTGTA ACAAGTAGTT TTGTTGGTTG GTTAATTGGT

Jarilla_chocola_Ycf1 TTTCGATGCA ACAACAAGAT GTTATTTGTA ACAAGTAGTT TTGTTGGTTG GTTAATTGGT

Jarilla_heterophella_Ycf1 TTTCGATGCA ACAACAAGAT GTTATTTGTA ACAAGTAGTT TTGTTGGTTG GTTAATTGGT

....|....| ....|....| ....|....| ....|....| ....|....| ....|....|

545 555 565 575 585 595

Carica_papaya_Ycf1 CACATTTTAT TCATGAAATG GGTTGGATTG GTATTAGTCT GGATACAGCA AAATAATTCT

Vasconcellea_Monoica_Ycf1 CACATTTTAT TCATGAAATG GGTTGGATTG GTATTAGTCT GGATACAGCA AAATAATTCT

Jacaratia_spinosa_Ycf1 CACATTTTAT TCATGAAATG GGTTGGATTG GTATTAGTCT GGATACAGCA AAATAATTCT

Vasconcellea_pubescens_Ycf1 CACATTTTAT TCATGAAATG GGTTGGATTG GTATTAGTCT GGATACAGCA AAATAATTCT

Jarilla_caudata_Ycf1 CACATTTTAT TCATGAAATG GGTTGGATTG GTATTAGTCT GGATACAGCA AAATAATTCT

Jarilla_chocola_Ycf1 CACATTTTAT TCATGAAATG GGTTGGATTG GTATTAGTCT GGATACAGCA AAATAATTCT

Jarilla_heterophella_Ycf1 CACATTTTAT TCATGAAATG GGTTGGATTG GTATTAGTCT GGATACAGCA AAATAATTCT

....|....| ....|....| ....|....| ....|....| ....|....| ....|....|

605 615 625 635 645 655

Carica_papaya_Ycf1 ATTAGGTCTA ATGTACTTAT TCGATCTAAT AAGTATAAGT ACCTTGTGTC AGAATTGAGA

Vasconcellea_Monoica_Ycf1 ATTAGGTCTA ATGTACTTAT TCGATCTAAT AAGTATAAAT ACCTTGTGTC AGAATTGAGA

Jacaratia_spinosa_Ycf1 ATTAGGTCTA ATGTACTTAT TCGATCTAAT AAGTATAAGT ACCTTGTGTC AGAATTGAGA

Vasconcellea_pubescens_Ycf1 ATTAGGTCTA ATGTACTTAT TCGATCTAAT AAGTATAAAT ACCTTGTGTC AGAATTGAGA

Jarilla_caudata_Ycf1 ATTAGGTCTA ATGTACTTAT TCGATCTAAT AAGTATAAGT ACCTTGTGTC AGAATTGAGA

Jarilla_chocola_Ycf1 ATTAGGTCTA ATGTACTTAT TCGATCTAAT AAGTATAAGT ACCTTGTGTC AGAATTGAGA

Jarilla_heterophella_Ycf1 ATTAGGTCTA ATGTACTTAT TCGATCTAAT AAGTATAAGT ACCTTGTGTC AGAATTGAGA

....|....| ....|....| ....|....| ....|....| ....|....| ....|....|

665 675 685 695 705 715

Carica_papaya_Ycf1 AATTCTATGG CTCGAATCTT TAGTATTCTC TTATTTATTA CCTGTGTCTA CTATTTAGGC

Vasconcellea_Monoica_Ycf1 AATTCTATGG CTCGAATCTT TAGTATTCTC TTATTTATTA CCTGTGTCTA CTATTTAGGC

Jacaratia_spinosa_Ycf1 AATTCTATGG CTCGAATCTT TAGTATTCTC TTATTTATTA CCTGTGTCTA CTATTTAGGC

Vasconcellea_pubescens_Ycf1 AATTCTATGG CTCGAATCTT TAGTATTCTC TTATTTATTA CCTGTGTCTA CTATTTAGGC

Jarilla_caudata_Ycf1 AATTCTATGG CTCGAATCTT TAGTATTCTC TTATTTATTA CCTGTGTCTA CTATTTAGGC

Jarilla_chocola_Ycf1 AATTCTATGG CTCGAATCTT TAGTATTCTC TTATTTATTA CCTGTGTCTA CTATTTAGGC

Jarilla_heterophella_Ycf1 AATTCTATGG CTCGAATCTT TAGTATTCTC TTATTTATTA CCTGTGTCTA CTATTTAGGC

....|....| ....|....| ....|....| ....|....| ....|....| ....|....|

725 735 745 755 765 775

Carica_papaya_Ycf1 AGAATACCGT CACCCATTTT TACTAAGAAA CTAAAAGGAA CCTCGGAAAC GGAGGAAAGG

Vasconcellea_Monoica_Ycf1 AGAATACCGT CACCCATTTT GACTAAGAAA ATAAAAGGAA TCTCAGAAAC GGAGGAAAGT

Jacaratia_spinosa_Ycf1 AGAATACCGT CACCCATTTT TACTAAGAAA ATAAAAGGAA TCTCAGAAAC GGAGGAAAGT

Vasconcellea_pubescens_Ycf1 AGAATACCGT CACCCATTTT TACTAAGAAA ATAAAAGGAA TCTCAGAAAC GGAGGAAAGT

Jarilla_caudata_Ycf1 AGAATACCGT CACCCATTTT TACTAAGAAA CTAAAAGGAA CCTCAGAAAC GGAGGAAAGG

Jarilla_chocola_Ycf1 AGAATACCGT CACCCATTTT TACTAAGAAA CTAAAAGGAA CCTCAGAAAT GGAGGAAAGG

Jarilla_heterophella_Ycf1 AGAATACCGT CACCCATTTT TACTAAGAAA CTAAAAGGAA CCTCAGAAAC GGAGGAAAGG

....|....| ....|....| ....|....| ....|....| ....|....| ....|....|
785 795 805 815 825 835

Carica_papaya_Ycf1 GGGGGGACTA AACAGGACCA AGAGGTATCC ACCGAAGAAG ATCCTTTTCC TTCTCTTTTT
Vasconcellea_Monoica_Ycf1 GGGGGGACTA AACAGGACCA AGAGGTATCC ACCGAAGAAG ATCCTTTTCC TTCTCTTTTT
Jacaratia_spinosa_Ycf1 GGGGGGACTA AACAGGACCA AGAGGTATCC ACCGAAGAAG ATCCTTTTCC TTCTCTTTTT
Vasconcellea_pubescens_Ycf1 GGGGGGACTA AACAGGACCA AGAGGTATCC ACCGAAGAAG ATCCTTTTCC TTCTCTTTTT
Jarilla_caudata_Ycf1 GGGGGGACTA AACAGGACCA AGAGGTATCC ACCGAAGAAG ATCCTTTTCC TTCTCTTTTT
Jarilla_chocola_Ycf1 GGGGGGACTA AACAGGACCA AGAGGTATCC ACCGAAGAAG ATCCTTTTCC TTCTCTTTTT
Jarilla_heterophella_Ycf1 GGGGGGACTA AACAGGACCA AGAGGTATCC ACCGAAGAAG ATCCTTTTCC TTCTCTTTTT

....|....| ....|....| ....|....| ....|....| ....|....| ....|....|
845 855 865 875 885 895

Carica_papaya_Ycf1 TCGGAAGAAA GGGAGGATCC GGACAAAATC GATGAAACGG AAGAGATCCG AGTGAATGGA
Vasconcellea_Monoica_Ycf1 TCGGAAGAAA GGGAGGATCC GGACAAAATC GATGAAACGG AAGAGATCCG AGTAAATGGA
Jacaratia_spinosa_Ycf1 TCGGAAGAAA GGGAGGATCC GGACAAAATC GATGAAACGG AAGAGATCCG AGTGAATGGA
Vasconcellea_pubescens_Ycf1 TCGGAAGAAA GGGAGGATCC GGACAAAATC GATGAAACGG AAGAGATCCG AGTAAATGGA
Jarilla_caudata_Ycf1 TCGGAAGAAA GGGAGGATCC GGACAAAATC GATGAAACGG AAGAGATCCG AGTGAATGGA
Jarilla_chocola_Ycf1 TCGGAAGAAA GGGAGGATCC GGACAAAATC GATGAAACGG AAGAGATCCG AGTGAATGGA
Jarilla_heterophella_Ycf1 TCGGAAGAAA GGGAGGATCC GGACAAAATC GATGAAACGG AAGAGATCCG AGTGAATGGA

....|....| ....|....| ....|....| ....|....| ....|....| ....|....|
905 915 925 935 945 955

Carica_papaya_Ycf1 AAGGAAAAAA AAAATAAGGA TGATGAATTT AAATTTAAAG AGATATCCTC TAACTTTTAT
Vasconcellea_Monoica_Ycf1 AAGGAAAAAA AAAATAAAGA TGATGAATTT CTCTTTACAG AGACATCCTC TAACTTA---
Jacaratia_spinosa_Ycf1 AAGGAAAAAA AAAATAAAGA TGATGAATTT ATCTTTAAAG AGACATCTTC TAACGTT---
Vasconcellea_pubescens_Ycf1 AAGGAAAAAA AAAATAAAGA TGATGAATTT CTCTTTACAG AGACATCCTC TAACTTA---
Jarilla_caudata_Ycf1 AAGGAAAATC AAAATAAGGA TGATGAATTT TTCTTTAAAG AGACATCCTC TAACTTT---
Jarilla_chocola_Ycf1 AAGGAAAATA AAAATAAGGA TGATGAATTT TTATTTAAAG AGACATCCTC TAACTTT---
Jarilla_heterophella_Ycf1 AAGGAAAATC AAAATAAGGA TGATGAATTT TTCTTTAAAG AGACATCCTC TAACTTT---

....|....| ....|....| ....|....| ....|....| ....|....| ....|....|
965 975 985 995 1005 1015

Carica_papaya_Ycf1 AACTTTAAAT ATAAAAATAG GCTAGGTTAT GAAAATCTAG ATGAGAATCA AGAAAATTCC
Vasconcellea_Monoica_Ycf1 ------AAAT ATAAAAATAG GCTAGTTTAT GAAAATCTAG ATGAGAATCA AGAAAATTCC
Jacaratia_spinosa_Ycf1 ------AAAT ATAAAAATAG GCTAGTTTAT GAAAATCTAG ATGAGAATCA AGAAAATTCC
Vasconcellea_pubescens_Ycf1 ------AAAT ATAAAAATAG GCTAGTTTAT GAAAATCTAG ATGAGAATCA AGAAAATTCC
Jarilla_caudata_Ycf1 ------AAAT ATAAAAAAAG GCTAGTTTCT GAAAATCTAG ATGAGAATCA AGAAAATTCC
Jarilla_chocola_Ycf1 ------AAAT ATAAAAAAAG GCTAGTTTCT GAAAATCTAG ATGAGAATCA AGAAAATTCC
Jarilla_heterophella_Ycf1 ------AAAT ATAAAAAAAG GCTAGTTTCT GAAAATCTAG ATGAGAATCA AGAAAATTCC

....|....| ....|....| ....|....| ....|....| ....|....| ....|....|
1025 1035 1045 1055 1065 1075

Carica_papaya_Ycf1 AATTTAGAAA TATTAAAAAG AAAAGAAGAT AAATATTTAT TATGGTTTGA AAAACCTTTT
Vasconcellea_Monoica_Ycf1 AATTTCGAAA TATTAAAAAG AAAAGAAGAT AAATATTTAT TATGGTTTGA AAAACCGTTT
Jacaratia_spinosa_Ycf1 AATTTAGAAA TATTAAAAAG AAAAGAAGAT AAATATTTAT TATGGTTTGA AAAACCTTTT
Vasconcellea_pubescens_Ycf1 AATTTCGAAA TATTAAAAAG AAAAGAAGAT AAATATTTTT TATGGTTTGA AAAACCGTTT
Jarilla_caudata_Ycf1 AATTTAGAAA TATTAAAAAA CAAAGAAGAT AAATATTTAT TATGGTTTGA AAAACCTTTT
Jarilla_chocola_Ycf1 AATTTAGAAA TATTAAAAAA CAAAGAAGAT AAATATTTAT TATGGTTTGA AAAACCTTTT
Jarilla_heterophella_Ycf1 AATTTAGAAA TATTAAAAAA CAAAGAAGAT AAATATTTAT TATGGTTTGA AAAACCTTTT

....|....| ....|....| ....|....| ....|....| ....|....| ....|....|
1085 1095 1105 1115 1125 1135

Carica_papaya_Ycf1 GTGACTCTTC TTTTTGATTA TAAACGGTGG AATCGGCCAT TTCGATATCT AAAAAATGAT
Vasconcellea_Monoica_Ycf1 GTGACTCTTC TTTTTGATTA TAAACGGTGG AATCGGCCAT TTCGATATAT AAAAAATGAT
Jacaratia_spinosa_Ycf1 GTGACTCTTC TTTTTGATTA TAAACGGTGG AATCGGCCAT TTCGATATAT AAAAAATGAT
Vasconcellea_pubescens_Ycf1 GTGACTCTTC TTTTTGATTA TAAACGGTGG AATCGGCCAT TTCGATATAT AAAAAATGAT
Jarilla_caudata_Ycf1 GTTACTCTTC TTTTTGATTA TAAACGATGG AATCGGCCAT TTCGATATAT AAAAAATGAT
Jarilla_chocola_Ycf1 GTTACTCTTC TTTTTGATTA TAAACGATGG AATCGGCCAT TTCGATATAT AAAAAATGAT
Jarilla_heterophella_Ycf1 GTTACTCTTC TTTTTGATTA TAAACGATGG AATCGGCCAT TTCGATATAT AAAAAATGAT

....|....| ....|....| ....|....| ....|....| ....|....| ....|....|
1145 1155 1165 1175 1185 1195

Carica_papaya_Ycf1 CGATTTGAAA ATGCTGTAAG AAATCAAATG TCACAATATT TTTTTTATAC ATGTCAAAGT
Vasconcellea_Monoica_Ycf1 CAGTTTGAAA ATGCTATAAG AAATAAAATG TCACAATATT TTTTTTATAC ATGTCAAAGT
Jacaratia_spinosa_Ycf1 CGGTTTGAAA ATGCTATAAG AAATAAAATG TCACAATATT TTTTTTATAC ATGTCAAAGT
Vasconcellea_pubescens_Ycf1 CAGTTTGAAA ATGCTATAAG AAATAAAATG TCACAATATT TTTTTTATAC ATGTCAAAGT
Jarilla_caudata_Ycf1 CAATTTGAAA ATGCTGTAAG AAATAAAATG TCACAATATT TTTTTTATAC ATGTCAAAGT
Jarilla_chocola_Ycf1 CAATTTGAAA ATGCTGTAAG AAATAAAATG TCACAATATT TTTTTTATAC ATGTCAAAGT
Jarilla_heterophella_Ycf1 CAATTTGAAA ATGCTGTAAG AAATAAAATG TCACAATATT TTTTTTATAC ATGTCAAAGT

....|....| ....|....| ....|....| ....|....| ....|....| ....|....|
1205 1215 1225 1235 1245 1255

Carica_papaya_Ycf1 GATGGAAAAG AAAGAATATC TTTTACGTAT CCACCCAGTT TGGCAACTTT TTTGGAAATG
Vasconcellea_Monoica_Ycf1 GATGGAAAAG AACAAATATC TTTTACGTAT CCACCCAGTT TGGCAACTTT TTTGGAACTG
Jacaratia_spinosa_Ycf1 GATGGAAAAG AAAGAATATC TTTTACGTAT CCACCCAGTT TGGCAACTTT TTTGGAAATG
Vasconcellea_pubescens_Ycf1 GATGGAAAAG AACGAATATC TTTTACGTAT CCACCCAGTT TGGCAACTTT TTTGGAACTG
Jarilla_caudata_Ycf1 GATGGAAAAG AAAGAATATC TTTTACGTAT CCACCCAGTT TGGCAACTTT TTTGGAAATG
Jarilla_chocola_Ycf1 GATGGAAAAG AAAGAATATC TTTTACGTAT CCACCCAGTT TGGCGACTTT TTTGGAAATG
Jarilla_heterophella_Ycf1 GATGGAAAAG AAAGAATATC TTTTACGTAT CCACCCAGTT TGGCAACTTT TTTGGAAATG

....|....| ....|....| ....|....| ....|....| ....|....| ....|....|
1265 1275 1285 1295 1305 1315

Carica_papaya_Ycf1 ATACAACAAA AGATGCCTTT GTTCACACAA GAAAAATTAT CCTCTAATGA ATTCTATAAT
Vasconcellea_Monoica_Ycf1 ATACAAAAAA AGATGCCGTT GTGCACACAA GACAAATTAT CCTCTAATGA ATTCTATAAT
Jacaratia_spinosa_Ycf1 ATACAAAAAA CGATGCCTTT GTTCACACAA GAAAAATTAT CTTCTAATGA ATTCTATAAT
Vasconcellea_pubescens_Ycf1 ATACAAAAAA AGATGCCGTT GTGCACACAA GACAAATTAT CCTCTAATGA ATTCTATAAT

```
Jarilla_caudata_Ycf1          ATACAAAAAA AGATGCCCTT GTTCACCCAA GAAAAATTAT CCTCTAATGA ATTCTATAAT
Jarilla_chocola_Ycf1          ATACAAAAAA AGATGCCCTT GTTCACCCAA GAAAAATTAT CCTCTAATGA ATTCTATAAT
Jarilla_heterophella_Ycf1     ATACAAAAAA AGATGCCCTT GTTCACCCAA GAAAAATTAT CCTCTAATGA ATTCTATAAT

                              ....|....| ....|....| ....|....| ....|....| ....|....| ....|....|
                                 1325       1335       1345       1355       1365       1375
Carica_papaya_Ycf1            CATTGGATTC TTACCAATGA GAAAAAAGAG AGCAAGTTAA AGAACGAATT TCTAAATAGA
Vasconcellea_Monoica_Ycf1     CATTGGATTC TTACCAATGA GAAAAAAGAG AGCAAGTTAA AGAACGAATT TATAAATAGA
Jacaratia_spinosa_Ycf1        CATTGGATTC TTACCAATGA GAAAAAAGAG AGCAAGTTAA AGAACGAATT TCTAAATAGA
Vasconcellea_pubescens_Ycf1   CATTGGATTC TTACCAATGA GAAAAAAGAG AGCAAGTTAA AGAACGAATT TATAAATAGA
Jarilla_caudata_Ycf1          CATTGGATTC TTACCAATGA GAAAAAAGAG AGTAAGTTAA CGAACGAATT TTTAAATAGA
Jarilla_chocola_Ycf1          CATTGGATTC TTACCAATGA GAAAAAAGAG AGTAAGTTAA CGAACGAATT TTTAAATAGA
Jarilla_heterophella_Ycf1     CATTGGATTC TTACCAATGA GAAAAAAGAG AGTAAGTTAA CGAACGAATT TTTAAATAGA

                              ....|....| ....|....| ....|....| ....|....| ....|....| ....|....|
                                 1385       1395       1405       1415       1425       1435
Carica_papaya_Ycf1            ATTAAAGCTT TAGATAAAGG ATTTCTTGCT CTGAATATAC TCGAAAAAAG GACTCGATTT
Vasconcellea_Monoica_Ycf1     ATTGAAACTT TAGATAAAGG AGTTCTTGCT CTGAATATAT TCGCAAAAAG GACTCGATTT
Jacaratia_spinosa_Ycf1        ATTGAAGCCT TAGATAAAGG ATTTCTTGCT CTGAATATAC TCGAAAAAAG GACTCGCTTT
Vasconcellea_pubescens_Ycf1   ATTGAAACTT TAGATAAAGG AGTTCTTGCT CTGAATATAT TCGCAAAAAG GACTCGATTT
Jarilla_caudata_Ycf1          ATTGAAGCTT TAGATAAAGG ATTTCTTGCT CTGAATATAC TCGAAAAAAG GACGCGATTT
Jarilla_chocola_Ycf1          ATTGAAGCTT TAGATAAAGG ATTTCTTGCT CTGAATATAC TCGAAAAAAG GACGCGATTT
Jarilla_heterophella_Ycf1     ATTGAAGCTT TAGATAAAGG ATTTCTTGCT CTGAATATAC TCGAAAAAAG GACGCGATTT

                              ....|....| ....|....| ....|....| ....|....| ....|....| ....|....|
                                 1445       1455       1465       1475       1485       1495
Carica_papaya_Ycf1            TGTAATAATG AGACTAAAAA AGAATATTTG CCTAAAATTT ATGATCCGTT ATTATATGGA
Vasconcellea_Monoica_Ycf1     TGTAATAATG AGACTAAAAA AGAATATTTG CCTAAAATTT ATGATCCGTT CTTATATGGA
Jacaratia_spinosa_Ycf1        TGTAATAATG AGACTAAAAA AGAATATTTG CCTAAAAGTT ATGATCCGTT CTTATATGGA
Vasconcellea_pubescens_Ycf1   TGTAATAATG AGACTAAAAA AGAATATTTG CCTAAAATTT ATGATCCGTT CTTATATGGA
Jarilla_caudata_Ycf1          TGTAATAATG AGACTAAAAA AGAATATTTG CCTAAAATTT ATGATCCGTT ATTATATGGA
Jarilla_chocola_Ycf1          TGTAATAATG AGACTAAAAA AGAATATTTG CCTAAAATTT ATGATCCGTT ATTATATGGA
Jarilla_heterophella_Ycf1     TGTAATAATG AGACTAAAAA AGAATATTTG CCTAAAATTT ATGATCCGTT ATTATATGGA

                              ....|....| ....|....| ....|....| ....|....| ....|....| ....|....|
                                 1505       1515       1525       1535       1545       1555
Carica_papaya_Ycf1            TCCTATCGTG GAAGAATCAT AAATTTTCTT TCACTACC-- -------TGA AACTTATATA
Vasconcellea_Monoica_Ycf1     TCCTATCGTG GAAGAATCAA AAATTTTCTT TCACTACCAA ACGTAACCGA AACTTATATA
Jacaratia_spinosa_Ycf1        TCCTATCGTG GAAGAATCAA AAATTTTCTT TCACTACCAA ACGTAACTGA AACTTATATA
Vasconcellea_pubescens_Ycf1   TCCTATCGTG GAAGAATCAA AAATTTTCTT TCACTACCAA ACGTAACCGA AACTTATATA
Jarilla_caudata_Ycf1          TCCTATCGTG GAAGAATCAT AAATTTTCTT TCACTACCAA ACGTAACTGA AACTTATATA
Jarilla_chocola_Ycf1          TCCTATCGTG GAAGAATCAT AAATTTTCTT TCACTACCAA ACGTAACTGA AACTTATATA
Jarilla_heterophella_Ycf1     TCCTATCGTG GAAGAATCAT AAATTTTCTT TCACTACCAA ACGTAACTGA AACTTATATA
```

	....|....|	....|....|	....|....|	....|....|	....|....|	....|....|
	1565	1575	1585	1595	1605	1615
Carica_papaya_Ycf1	AAAAATAACA	TAGGAACGGT	TTTGATAAAT	AAGATTCATA	GTATACTTCT	TAATACTGAT
Vasconcellea_Monoica_Ycf1	AAAAATAACA	TAGGAACGGT	TCTGATAAAT	AAGATTCATA	GTCTACTTCT	TAATACTGAT
Jacaratia_spinosa_Ycf1	AAAAATAACA	TAGGAACGGT	TCTGATAAAT	AAGATTCATA	GTATACTTCT	TAATACTGAT
Vasconcellea_pubescens_Ycf1	AAAAATAACA	TAGGAACGGT	TCTGATAAAT	AAGATTCATA	GTCTACTTCT	TAATACTGAT
Jarilla_caudata_Ycf1	AAAAAAAACA	TAGGAACGGT	TTTGATAAAT	AAGATTCATA	GTATACTTCT	TAATACTGAT
Jarilla_chocola_Ycf1	AAAAAAAACA	TAGGAACGGT	TTTGATAAAT	AAAATTCATA	GGATACTTCT	TAATACTGAT
Jarilla_heterophella_Ycf1	AAAAAAAACA	TAGGAACGGT	TTTGATAAAT	AAGATTCATA	GTATACTTCT	TAATACTGAT

	....|....|	....|....|	....|....|	....|....|	....|....|	....|....|
	1625	1635	1645	1655	1665	1675
Carica_papaya_Ycf1	TATCACGAAT	TTGAACAGAC	AATAGAAATA	GATACACTTA	ATAGAAAATC	ATTATCAATA
Vasconcellea_Monoica_Ycf1	TATCACGAAT	TTGAACAGAC	AATAGAAATA	GATACACTTA	ATAGAAAATC	ATTATCAATA
Jacaratia_spinosa_Ycf1	TATCACGAAT	TTGAACAGAC	AATAGAAATA	GATACACTTA	ATAGAAAATC	ATTATCAATA
Vasconcellea_pubescens_Ycf1	TATCACGAAT	TTGAACAGAC	AATAGAAATA	GATACACTTA	ATAGAAAATC	ATTATCAATA
Jarilla_caudata_Ycf1	TATCACGAAT	TTGAACAGAC	AATAGAAATA	GATACACTTA	ATAGAAAATC	ATTATCAATA
Jarilla_chocola_Ycf1	TATCACGAAT	TTGAACAGAC	AATAGAAATA	GATACACTTA	ATAGAAAATC	ATTATCAATA
Jarilla_heterophella_Ycf1	TATCACGAAT	TTGAACAGAC	AATAGAAATA	GATACACTTA	ATAGAAAATC	ATTATCAATA

	....|....|	....|....|	....|....|	....|....|	....|....|	....|....|
	1685	1695	1705	1715	1725	1735
Carica_papaya_Ycf1	GAAAAAAGAC	TTTCTTTATT	TCCA------	GAACACCAAC	AAGAGCAAGT	TGATTCAAAA
Vasconcellea_Monoica_Ycf1	GAAAAAAGAC	TTTCTTTATT	TCCATTTCCA	GAACACCAAA	AAGAGCAAGT	TGATTCAAAA
Jacaratia_spinosa_Ycf1	GAAAAAAGAC	TTTATTTATT	TCCA------	GAACACCAAA	AAGAGAAAGT	TGATTCAAAA
Vasconcellea_pubescens_Ycf1	GAAAAAAGAC	TTTCTTTATT	TCCATTTCCA	GAACACCAAA	AAGAGCAAGT	TGATTCAAAA
Jarilla_caudata_Ycf1	GAAAAAAGAC	TTTCTTTATT	TCCA------	GAACACCAAC	AAGAGCAAGT	TGATTCAAAA
Jarilla_chocola_Ycf1	GAAAAAAGAC	TTTCTTTATT	TCCA------	GAACACCAAC	AAGAGCAAGT	TGATTCAAAA
Jarilla_heterophella_Ycf1	GAAAAAAGAC	TTTCTTTATT	TCCA------	GAACACCAAC	AAGAGCAAGT	TGATTCAAAA

	....|....|	....|....|	....|....|	....|....|	....|....|	....|....|
	1745	1755	1765	1775	1785	1795
Carica_papaya_Ycf1	AATCGAAAAA	ATAAAA-TTC	AATTTTTATT	CTTTTTATTC	GATGTGGTTC	TAACTAATCC
Vasconcellea_Monoica_Ycf1	AATCGAAAAA	AAAAAA-TTC	AATTTTTATT	C---------	GATGCGGTTC	TAACTGATCC
Jacaratia_spinosa_Ycf1	AATCGAAAAA	AAAAAA-TTC	CATTTTTATT	T---------	GATACGGTTC	TAACTGATCC
Vasconcellea_pubescens_Ycf1	AATCGAAAAA	AAAAAA-TTC	AATTTTTATT	C---------	GATGCGGCTC	TAACTGATCC
Jarilla_caudata_Ycf1	AATCGAAAAA	AAAAAAATTC	AATTTTTATT	T---------	GATGCGGTTC	TAACTGATCC
Jarilla_chocola_Ycf1	AATCGAAAAA	AAAAAA-TTC	AATTTTTATT	T---------	GATGCGGTTC	TAACTGATCC
Jarilla_heterophella_Ycf1	AATCGAAAAA	AAAAAA-TTC	AATTTTTATT	T---------	GATGCGGTTC	TAACTGATCC

	....|....|	....|....|	....|....|	....|....|	....|....|	....|....|
	1805	1815	1825	1835	1845	1855
Carica_papaya_Ycf1	CAATGATCAA	ACGAATTCTG	TTGGAATAAA	AGAAATCAGT	AAAAAAGTTC	CTCGTTGGTC
Vasconcellea_Monoica_Ycf1	CAATGATCAA	ACGAATTCTG	TTGGAATAAA	AGAAATCAGT	AAAAAAGTTC	CTTGTTGGTC

Jacaratia_spinosa_Ycf1 CAATGATCTA ACAAATTCTG TTGGAATAAA AGAAATCAGT AAAAAAGTTC CTCGTTGGTC

Vasconcellea_pubescens_Ycf1 CAATGATCAA ACGAATTCTG TTGGAATAAA AGAAATCAGT AAAAAAGTTC CTCGTTGGTC

Jarilla_caudata_Ycf1 CAATGATCAA ACGAATTCTG TTGGAATAAA AGAAATCAGT AAAAAAGTTC CTCGTTGGTC

Jarilla_chocola_Ycf1 CAATGATCAA ACGAATTCTG TTGGAATAAA AGAAATCAGT AAAAAAGTTC CTCGTTGGTC

Jarilla_heterophella_Ycf1 CAATGATCAA ACGAATTCTG TTGGAATAAA AGAAATCAGT AAAAAAGTTC CTCGTTGGTC

....|....| ....|....| ....|....| ....|....| ....|....| ....|....|

1865 1875 1885 1895 1905 1915

Carica_papaya_Ycf1 ATACAAATTA ATCGACGATT TAGAACAACT GGAGGGGGAA AATGAAGAAA ACGTGACAGC

Vasconcellea_Monoica_Ycf1 ATACAAATTG ATCGACGATT TAGAACAACA GGAGGGGGAA AATGAAGAAA GCGTGACCGC

Jacaratia_spinosa_Ycf1 ATACAAATTA ATCGACGATT TAGAACAACA GGAGGGGGAA AATGAAGAAA GCGTGACAGC

Vasconcellea_pubescens_Ycf1 ATACAAATTG ATCGACGATT TAGAACAACA GGAGGGGGAA AATGAAGAAA GCGTGACCGC

Jarilla_caudata_Ycf1 ATACAAATTA ATCGACGATT TAGAACTACT GGAGGGGGAA AATGAAGAAA ACATGACAGG

Jarilla_chocola_Ycf1 ATACAAATTA ATCGACGATT TAGAACTACT GGAGGGGGAA AATGAAGAAA ACATGACAGG

Jarilla_heterophella_Ycf1 ATACAAATTA ATCGACGATT TAGAACTACT GGAGGGGGAA AATGAAGAAA ACATGACAGG

....|....| ....|....| ....|....| ....|....| ....|....| ....|....|

1925 1935 1945 1955 1965 1975

Carica_papaya_Ycf1 GGATCCTGAA ATTCGTTCAA GAAAAGCCAA ACATGTAGTG ATTTTTACTG ATAACCAACA

Vasconcellea_Monoica_Ycf1 CGATCCTGAA ATTCGTTCAA GAAAAGCCAA ACATGTAGTG ATTTTTACTG ATAACCAACA

Jacaratia_spinosa_Ycf1 CGATCCTGAA ATTCGTTCAA GAAAAGCCAA ACGTGTAGTG ATTTTTACTG ATAACCAACA

Vasconcellea_pubescens_Ycf1 CGATCCTGAA ATTCGTTCAA GAAAAGCCAA ACATGTAGTG ATTTTTACTG ATAACCAACA

Jarilla_caudata_Ycf1 GGATCATGAA ATTCGTTCAA GAAAAGCCAA ACGTGTAGTG ATTTTTACTG AGAACCAAGA

Jarilla_chocola_Ycf1 GGATCATGAA ATTCGTTCAA GAAAAGCCAA ACGTGTAGTG ATTTTTACTG AGAACCAAGA

Jarilla_heterophella_Ycf1 GGATCATGAA ATTCGTTCAA GAAAAGCCAA ACGTGTAGTG ATTTTTACTG AGAACCAAGA

....|....| ....|....| ....|....| ....|....| ....|....| ....|....|

1985 1995 2005 2015 2025 2035

Carica_papaya_Ycf1 GAATAACGAT ATTTATACTA ACATCAAAGA TACTAATAAT TCTGATGAAA GAGACGAAGT

Vasconcellea_Monoica_Ycf1 GAATAACGAT ATTTATACTA ATATAAAAGA TATTAATAAT TCTGATCAAA GAGACGAAGT

Jacaratia_spinosa_Ycf1 GAATAACGAT ATTTATACTA ATATCAAAGA TACTAATAAT TCTGATCAAA GAGACGAAGT

Vasconcellea_pubescens_Ycf1 GAATAACGAT ATTTATACTA ATATAAAAGA TATTAATAAT TCTGATCAAA GAGACGAAGT

Jarilla_caudata_Ycf1 GAATAACGAT ATTTATAATA GTCTCCAAGA TTTTACTAAT TCTGATCAAA GAGACGAAGT

Jarilla_chocola_Ycf1 GAATAACGAT ATTTATAATA GTCTCCAAGA TTCTACTAAT TCGGATCAAA GAGACGAAGT

Jarilla_heterophella_Ycf1 GAATAACGAT ATTTATAATA GTCTCCAAGA TTTTACTAAT TCTGATCAAA GAGACGAAGT

....|....| ....|....| ....|....| ....|....| ....|....| ....|....|

2045 2055 2065 2075 2085 2095

Carica_papaya_Ycf1 AGCTTTGATA CGTTATTCAC AACAATCAGA TTTTCGTCGA GACATAATCA AAGGATCCAT

Vasconcellea_Monoica_Ycf1 GGCTTTGATA CGTTATTCAC AACAATCAGA TTTTCGTCGA GACATAATCA AAGGCTCCAT

Jacaratia_spinosa_Ycf1 GGCTTTGATA CGTTATTCAC AACAATCAGA TTTTCGTCGA GACATAATCA AAGGCTCCAT

Vasconcellea_pubescens_Ycf1 GGCTTTGATA CGTTATTCAC AACAATCAGA TTTTCGTCGA GACATAATCA AAGGCTCCAT

Jarilla_caudata_Ycf1 GGCTTTGATA CGTTATTCAC AACAATCAGA TTTTCGTCGA GACATAATCA AAGGCTCCAT

Jarilla_chocola_Ycf1 GGCTTTGATA CGTTATTCAC AACAATCAGA TTTTCGTCGA GACATAATCA AAGGCTCCAT

Jarilla_heterophella_Ycf1 GGCTTTGATA CGTTATTCAC AACAATCAGA TTTTCGTCGA GACATAATCA AAGGCTCCAT

....|....| ....|....| ....|....| ....|....| ....|....| ....|....|
2105 2115 2125 2135 2145 2155

Carica_papaya_Ycf1 GCGTCCTCAA AGACGTAAAA CAGTTACTTG GGAACTGTTT CAAGCAAATG CGCATTCCCC
Vasconcellea_Monoica_Ycf1 GCGTGCTCAA AGACGTAAAA CAGTTCCTTG GGAACTGTTT CAAGCAAATG TGCATTCGCC
Jacaratia_spinosa_Ycf1 GCGTGCTCAA AGACGTAAAA CAGTTACTTG GGAACTGTTT CAAGCAAATG TGCATTCCCC
Vasconcellea_pubescens_Ycf1 GCGTGCTCAA AGACGTAAAA CAGTTCCTTG GGAACTGTTT CAAGCAAATG TGCATTCGCC
Jarilla_caudata_Ycf1 GCGTGCTCAA AGACGTAAAA CAGTTCCTTG GGAACTATTT CAATCAAATG TGCATTCCCC
Jarilla_chocola_Ycf1 GCGTGCTCAA AGACGTAAAA CAGTTCCTTG GGAACTATTT CAATCAAATG TGCATTCCCC
Jarilla_heterophella_Ycf1 GCGTGCTCAA AGACGTAAAA CAGTTCCTTG GGAACTATTT CAATCAAATG TGCATTCCCC

....|....| ....|....| ....|....| ....|....| ....|....| ....|....|
2165 2175 2185 2195 2205 2215

Carica_papaya_Ycf1 TCTTTTTTTG GACAGAATAG ACAAACCCCT TTTTTTTTTT TCTGCATTTG ATATTTCTGA
Vasconcellea_Monoica_Ycf1 TCTTTTTTTG GACAGAATAG ACAAACCCCT TTTTTCTT-- ----CATTTG ATATTTCTGA
Jacaratia_spinosa_Ycf1 TCTTTTTTTG GACAGAATAG ACAAACCCCT TTTTTCTT-- ----CATTTG ATATTTCTGA
Vasconcellea_pubescens_Ycf1 TCTTTTTTTG GACAGAATAG ACAAACCCCT TTTTTCTT-- ----CATTTG ATATTTCTGA
Jarilla_caudata_Ycf1 TCTTTTTTTG GACAGAATAG ACAAACCTCT TTTTTCTT-- ----TATTTG ATATTTCTGA
Jarilla_chocola_Ycf1 TCTTTTTTTG GACAGAATAG ACAAACCTCT TTTTTCTT-- ----TATTTG ATATTTCTGA
Jarilla_heterophella_Ycf1 TCTTTTTTTG GACAGAATAG ACAAACCTCT TTTTTCTT-- ----TATTTG ATATTTCTGA

....|....| ....|....| ....|....| ....|....| ....|....| ....|....|
2225 2235 2245 2255 2265 2275

Carica_papaya_Ycf1 ACTGATGAAA CGCATTTTTA GAAATAGGAT ATGGATACGG ACAAACCAGG AAAAAAAAAA
Vasconcellea_Monoica_Ycf1 ACTGATGAAA CTCATTTTTA GAAATAGGAT ATGGG----- -CAAACAAGG AAAAAAAAAT
Jacaratia_spinosa_Ycf1 ACTGATGAAA CTCATTTTTA GAAATAGGAT ATGGG----- -CAAACAAGG AAAAAAAAAT
Vasconcellea_pubescens_Ycf1 ACTGATGAAA CTCATTTTTA GAAATAGGAT ATGGG----- -CAAACAAGG AAAAAAAAAT
Jarilla_caudata_Ycf1 ACTGATGAAA CTTATTTTTA CAAATAGGAT ATGGA----- -CAAACAAGG AAAAAAAAAA
Jarilla_chocola_Ycf1 ACTGATGAAA CTTATTTTTA CAAATAGGAT ATGGA----- -CAAACAAGG AAAAAAAAAA
Jarilla_heterophella_Ycf1 ACTGATGAAA CTTATTTTTA CAAATAGGAT ATGGA----- -CAAACAAGG AAAAAAAAAA

....|....| ....|....| ....|....| ....|....| ....|....| ....|....|
2285 2295 2305 2315 2325 2335

Carica_papaya_Ycf1 TTCTGATTAT ACAGAAGAAA AGACAAAAAC AATTGAGAAA AAAGAAGACG ACAAAAGAGA
Vasconcellea_Monoica_Ycf1 TTCTGATTAT ACGGAAGAAA AGACAAAAAC AATTGCGAAA ACAAAAGAAG ACAAAAGAGA
Jacaratia_spinosa_Ycf1 TTCTGATTAT ACAGAAGAAA AGACAAAAAC AATTGAGAAA AAAAAAGAAG ACAAAAGAGA
Vasconcellea_pubescens_Ycf1 TTCTGATTAT ACGGAAGAAA AGACAAAAAC AATTGCGAAA ACAAAAGAAG ACAAAAGAGA
Jarilla_caudata_Ycf1 TTCTGATTAT ACAGAAGAAA AGACAAAAAC AATTGAGAAA AAAGAAGAAG ATAAAAGAAA
Jarilla_chocola_Ycf1 TTCTGATTAT ACAGAAGAAA AGACAAAAAC AATTGAGAAA AAAGAAGAAG ATAAAAGAAA
Jarilla_heterophella_Ycf1 TTCTGATTAT ACAGAAGAAA AGACAAAAAC AATTGAGAAA AAAGAAGAAG ATAAAAGAAA

....|....| ....|....| ....|....| ....|....| ....|....| ....|....|
2345 2355 2365 2375 2385 2395

Carica_papaya_Ycf1 ACAATACAAA AGAGAAGAAA AAACACGAAT AGAAATAGCG GAAGCCTGGG ATAGTCTTTT
Vasconcellea_Monoica_Ycf1 AAAATACAAA AGAGAAGAAA AAACACGGAT AGAAATAGCG GAAGCCTGGG ATAGTCTTTT
Jacaratia_spinosa_Ycf1 AAAATACAAA AGAGAAGAAA AAACACGGAT AGAAATAGCG GAAGCCTGGG ATAGTCTTTT
Vasconcellea_pubescens_Ycf1 AAAATACAAA AGAGAAGAAA AAACACGGAT AGAAATAGCG GAAGCCTGGG ATAGTCTTTT
Jarilla_caudata_Ycf1 AAAATACAAA AGAGAGGAAA AAACACGGAT AGAAATAGCA GAAACCTGGG ATAGTCTTTT
Jarilla_chocola_Ycf1 AAAATACAAA AGAGAGGAAA AAACACGGAT AGAAATAGCA GAAACCTGGG ATAGTCTTTT
Jarilla_heterophella_Ycf1 AAAATACAAA AGAGAGGAAA AAACACGGAT AGAAATAGCA GAAACCTGGG ATAGTCTTTT

....|....| ....|....| ....|....| ....|....| ....|....| ....|....|
2405 2415 2425 2435 2445 2455

Carica_papaya_Ycf1 ATTTGCTCAA GTTCTAAGAG GTTTCGTCTT AGTAACCCAA TCGATTCTTA GAAAATATAT
Vasconcellea_Monoica_Ycf1 ATTTGCTCAA GTTATAAGGG GTTTGGTCTT AGTAACCCAC TCGATTATTA GAAAATATAT
Jacaratia_spinosa_Ycf1 ATTTGTTCAA GTTATAAGGG GTTTGGTCTT AGTAACCCAA TCGATTCTTA GAAAATATAT
Vasconcellea_pubescens_Ycf1 ATTTGCTCAA GTTATAAGGG GTTTGGTCTT AGTAACCCAC TCGATTATTA GAAAATATAT
Jarilla_caudata_Ycf1 ATTTGCTCAA GTTCTAAGAG GTTTCGTCTT AGTAACCCAC TCGATTCTTA GAAAATATAT
Jarilla_chocola_Ycf1 ATTTGCTCAA GTTCTAAGAG GTTTCGTCTT AGTAACCCAC TCGATTCTTA GAAAATATAT
Jarilla_heterophella_Ycf1 ATTTGCTCAA GTTCTAAGAG GTTTCGTCTT AGTAACCCAC TCGATTCTTA GAAAATATAT

....|....| ....|....| ....|....| ....|....| ....|....| ....|....|
2465 2475 2485 2495 2505 2515

Carica_papaya_Ycf1 TATATTACCT TCCTTGATAA TAGTTAAAAA TATCGCCCGT CTGCTATTAT TTCAATTTTC
Vasconcellea_Monoica_Ycf1 TATATTACCT TCCTTGATAA TAGTTAAAAA TATAGCCCGT ATGCTATTAT TTCAATTTCC
Jacaratia_spinosa_Ycf1 TATATTACCT TCCTTGATAA TAGTTAAAAA TATCGCCCGT ATGCTATTAT TTCAATTTCC
Vasconcellea_pubescens_Ycf1 TATATTACCT TCCTTGATAA TAGTTAAAAA TATAGCCCGT ATGCTATTAT TTCAATTTCC
Jarilla_caudata_Ycf1 TATATTACCG TCCTTGATAA TAGTTAAAAA TATCGCCCGT ATCCTATTAT TTCAAATTCC
Jarilla_chocola_Ycf1 TATATTACCG TCCTTGATAA TAGTTAAAAA TATCGCCCGT ATCCTATTGT TTCAAATTCC
Jarilla_heterophella_Ycf1 TATATTACCG TCCTTGATAA TAGTTAAAAA TATCGCCCGT ATCCTATTAT TTCAAATTCC

....|....| ....|....| ....|....| ....|....| ....|....| ....|....|
2525 2535 2545 2555 2565 2575

Carica_papaya_Ycf1 CGAATGGTCT GAGGATTTAA AGGATTGGAA TCGAGAAATG CATATTAAAT GTACCTATAA
Vasconcellea_Monoica_Ycf1 CGAATGGTCT GAGGATTTAA AGGATTGGAA TCGAGAAATG CATATTAAAT GCACCTATAA
Jacaratia_spinosa_Ycf1 CGAATGGTCT GAGGATTTAA AGGATTGGAA TCGAGAAATG CATATTAAAT GCACCTATAA
Vasconcellea_pubescens_Ycf1 CGAATGGTCT GAGGATTTAA AGGATTGGAA TCGAGAAATG CATATTAAAT GCACCTATAA
Jarilla_caudata_Ycf1 CGAATGGTCT GAGGATTTAA AGGATTGGAA TCGAGAAATG CATATTAAAT GCACCTATAA
Jarilla_chocola_Ycf1 CGAATGGTCT GAGGATTTAA AGGATTGGAA TCGAGAAATG CATATTAAAT GCACCTATAA
Jarilla_heterophella_Ycf1 CGAATGGTCT GAGGATTTAA AGGATTGGAA TCGAGAAATG CATATTAAAT GCACCTATAA

....|....| ....|....| ....|....| ....|....| ....|....| ....|....|
2585 2595 2605 2615 2625 2635

Carica_papaya_Ycf1 TGGTGTTCAA TTATCAGAAA CAGAATTTCC AAAAAACTGG TTAACAGACG GTATTCAGAT
Vasconcellea_Monoica_Ycf1 TGGAGTTCAA TTATCAGAAA CAGAATTTCC AAAAAACTGG TTAACAGACG GTATTCAGAT
Jacaratia_spinosa_Ycf1 TGGTGTTCAA TTATCCGAAA CAGAATTTCC AAAAAACTGG TTAACAGACG GTATTCAGAT
Vasconcellea_pubescens_Ycf1 TGGAGTTCAA TTATCAGAAA CAGAATTTCC AAAAAACTGG TTAACAGACG GTATTCAGAT

Jarilla_caudata_Ycf1 TGGTGTTCAA TTATCAGAAA CAGAATTTCC AAAAAACTGG TTAACAGACG GTATTCAGAT
Jarilla_chocola_Ycf1 TGGTGTTCAA TTATCAGAAA CAGAATTTCC AAAAAACTGG TTAACGGACG GTATTCAGAT
Jarilla_heterophella_Ycf1 TGGTGTTCAA TTATCAGAAA CAGAATTTCC AAAAAACTGG TTAACAGACG GTATTCAGAT

....|....| ....|....| ....|....| ....|....| ....|....| ....|....|
2645 2655 2665 2675 2685 2695

Carica_papaya_Ycf1 AAAAATACTA TTCCCTTTTT GCCTTAAACC TTGGCACAGA TCGAAATTAC GATCCCCTCA
Vasconcellea_Monoica_Ycf1 AAAAATACTA TTCCCTTTTT GCCTTAAACC TTGGCACAGA TCTAAATTAC GATCCCCTCA
Jacaratia_spinosa_Ycf1 AAAAATACTA TTCCCTTTTT GCCTTAAACC TTGGCACAGA TCTAAATTAC GATCCCCTCA
Vasconcellea_pubescens_Ycf1 AAAAATACTA TTCCCTTTTT GCCTTAAACC TTGGCACAGA TCTAAATTAC GATCCCCTCA
Jarilla_caudata_Ycf1 GAAAATACTA TTCCCTTTTT GCCTTAAACC TTGGCACAGA TCGAAATTAC GATCCCCTCA
Jarilla_chocola_Ycf1 AAAAATACTA TTCCCTTTTT GCCTTAAACC TTGGCACAGA TCGAAATTAC GATCCCCTCA
Jarilla_heterophella_Ycf1 AAAAATACTA TTCCCTTTTT GCCTTAAACC TTGGCACAGA TCGAAATTAC GATCCCCTCA

....|....| ....|....| ....|....| ....|....| ....|....| ....|....|
2705 2715 2725 2735 2745 2755

Carica_papaya_Ycf1 TAAAGATCCA ATCAAAAAGA GGAAGGGGGA AAAAAATGAT TTTTGTTTTT TAACAGTTTG
Vasconcellea_Monoica_Ycf1 TAAAGATCCA ATCAAAAAAA GGAAGGGAGA AAAAAATGAT TTTTGTTTTT TAACAGTTTG
Jacaratia_spinosa_Ycf1 TAAAGATCCA ATCAAAAAGA GGAAGGGGGA AAAAAATGAT TTTTGTTTTT TAACAGTTTG
Vasconcellea_pubescens_Ycf1 TAAAGATCCA ATCAAAAAAA GGAAGGGAGA AAAAAATGAT TTTTGTTTTT TAACAGTTTG
Jarilla_caudata_Ycf1 TAAAGATCCA ATCAAAAAGG GGAGGGGGGA AAAAAATGAT TTTTGTTTTT TAACAGTTTG
Jarilla_chocola_Ycf1 TAAAGATCCA ATCAAAAAGG GGAGGGGGGA AAAAAATGAT TTTTGTTTTT TAACAGTTTG
Jarilla_heterophella_Ycf1 TAAAGATCCA ATCAAAAAGG GGAGGGGGGA AAAAAATGAT TTTTGTTTTT TAACAGTTTG

....|....| ....|....| ....|....| ....|....| ....|....| ....|....|
2765 2775 2785 2795 2805 2815

Carica_papaya_Ycf1 GGGGATGGAA ACTGAATTGC CTTTTGGTTC TCCCCGAAAA CGGCCTTCAT TTTTTGAACC
Vasconcellea_Monoica_Ycf1 GGGGATGGAA ACTGAATTTC CTTTTGGTTC TCCCCGAAAA CGGCCTTCAT TTTTTGAACC
Jacaratia_spinosa_Ycf1 GGGGATGGAA ACTGAATTTC CTTTTGGTTC GCCCCGAAAA CGGCCTTCAT TTTTTGAACC
Vasconcellea_pubescens_Ycf1 GGGGATGGAA ACTGAATTTC CTTTTGGTTC TCCCCGAAAA CGGCCTTCAT TTTTTGAACC
Jarilla_caudata_Ycf1 GGGGATGGAA ACTGAATTGC CTTTTGGTTC TCCCCGAAAA CGGCCGTCAT TTTTTGAACC
Jarilla_chocola_Ycf1 GGGGATGGAA ACTGAATTGC CTTTTGGTTC TCCCCGAAAA CGGCCGTCAT TTTTTGAACC
Jarilla_heterophella_Ycf1 GGGGATGGAA ACTGAATTGC CTTTTGGTTC TCCCCGAAAA CGGCCGTCAT TTTTTGAACC

....|....| ....|....| ....|....| ....|....| ....|....| ....|....|
2825 2835 2845 2855 2865 2875

Carica_papaya_Ycf1 TCTTTTTAAA GAATTGAAAA AAAAAATTAG AAAATTGAAA ATTAAGTATT TTCTGGGTTT
Vasconcellea_Monoica_Ycf1 TATTTTTAAA GAATTGAAAA AAAAAATTAT AAAATTGAAA ATTAAGTATT TTCTGGGTTT
Jacaratia_spinosa_Ycf1 TATTTTTAAA GAATTGAAAA AAAAAATTAG AAAATTGAAA ATTAAGTATT TTCTGGGTTT
Vasconcellea_pubescens_Ycf1 TATTTTTAAA GAATTGAAAA AAAAAATTAT AAAATTGAAA ATTAAGTATT TTCTGGGTTT
Jarilla_caudata_Ycf1 TATTTTTAAA GAATTGAAAA AAAAAATTAG AAAATTGAAA ATTAAGGATT TTCTGGGTTT
Jarilla_chocola_Ycf1 TATTTTTAAA GAATTGAAAA AAAAAATTAG AAAATTGAAA ATTAAGGATT TTCTGGGTTT
Jarilla_heterophella_Ycf1 TATTTTTAAA GAATTGAAAA AAAAAATTAG AAAATTGAAA ATTAAGGATT TTCTGGGTTT

	....|....| ....|....| ....|....| ....|....| ....|....| ....|....|
	2885 2895 2905 2915 2925 2935
Carica_papaya_Ycf1	AAGGGTTTTA AAAGAAAGAG CAAAAATATT TGTAAAAGTC TCAAAAGAAA CAAAAAAACA
Vasconcellea_Monoica_Ycf1	ACGGGTTTTA AAAGAAAGAG CAAAAATTTT TCTAAAAGTC TCAAAAGAAA CAAAAAAACA
Jacaratia_spinosa_Ycf1	AAGGGTTTTA AAAGAAAGAG CAAAAATTTT TCTAAAAGTC TCAAAAGAAA CAAAAAAACA
Vasconcellea_pubescens_Ycf1	ACGGGTTTTA AAAGAAAGAG CAAAAATTTT TCTAAAAGTC TCAAAAGAAA CAAAAAAACA
Jarilla_caudata_Ycf1	AAGGATTTTA AAAGAAAGAG CAAAAATTTT TCTAAAAGTC TCAAAAGAAC CAAAAAAACA
Jarilla_chocola_Ycf1	AAGGGTTTTA AAAGAAAGAG CAAAAATTTT TCTAAAAGTC TCAAAAGAAC CAAAAAAACA
Jarilla_heterophella_Ycf1	AAGGGTTTTA AAAGAAAGAG CAAAAATTTT TCTAAAAGTC TCAAAAGAAC CAAAAAAACA

	....|....| ....|....| ....|....| ....|....| ....|....| ....|....|
	2945 2955 2965 2975 2985 2995
Carica_papaya_Ycf1	GGTCAGTACA AACCTTCTAT TTGTAAAAGG AATAATAAAA GAATTTTCAA AACGAAACCG
Vasconcellea_Monoica_Ycf1	AGTGATTACA AACCTTCTAT TTGTAAAAGG AATACTAAAA GAATTTTCAA AAATAAACCT
Jacaratia_spinosa_Ycf1	GGTCATTACA AACCTTCTAT TTGTCAAAGG AATAATAAAA GAATTTTCAA AAATCAACCC
Vasconcellea_pubescens_Ycf1	AGTCATTACA AACCTTCTAT TTGTAAAAGG AATACTAAAA GAATTTTCAA AAATAAACCT
Jarilla_caudata_Ycf1	GGTCAGTACA AACCTTCTAT TTGTAAAAGA AATAATCAAA GAATTTTCAA AAATAAACCG
Jarilla_chocola_Ycf1	GGTCAGTACA AACCCTCTAT TTGTAAAAGA AATAATCAAA GAATTTTCAA AAATAAACCG
Jarilla_heterophella_Ycf1	GGTCAGTACA AACCTTCTAT TTGTAAAAGA AATAATCAAA GAATTTTCAA AAATAAACCG

	....|....| ....|....| ....|....| ....|....| ....|....| ....|....|
	3005 3015 3025 3035 3045 3055
Carica_papaya_Ycf1	AAGTCCATTA TTTGGATTGA AAGAAATATC TGAATTGAGT GAAACGAAAA AAGATTCAAG
Vasconcellea_Monoica_Ycf1	AATTCCATTA TTTGGATTGA GAGAAATATC TGAATTGAGT GAAACGAAAA AAGATTCACG
Jacaratia_spinosa_Ycf1	AATTCCATTA TTTGTATTGA GAGAAATATC TGAATTGAGT GAAATGAAAA AAGATTCAAG
Vasconcellea_pubescens_Ycf1	AATTCCATTA TTTGGATTGA GAGAAATATC TGAATTGAGT GAAACGAAAA AAGATTCACG
Jarilla_caudata_Ycf1	AATTCCATTA TTTGGATTGA GAGAAATATC TGAATTGAGT GAAACGAAAA AAGATTCAAG
Jarilla_chocola_Ycf1	AATTCCATTA TTTGGATTGA GAGAAATATC TGAATTGAGT GAAACGAAAA AAGATTCAAG
Jarilla_heterophella_Ycf1	AATTCCATTA TTTGGATTGA GAGAAATATC TGAATTGAGT GAAACGAAAA AAGATTCAAG

	....|....| ....|....| ....|....| ....|....| ....|....| ....|....|
	3065 3075 3085 3095 3105 3115
Carica_papaya_Ycf1	AATTAGTAAT CAGATGATTC CCGAATCGTC TGTTCAAATT CGATCTATGG AGTGGACAAA
Vasconcellea_Monoica_Ycf1	AATGAGTAAT CAGATGATTC ATGAATTGTC TGTTCAAATT CGATCTATGG AGTGGAAAAA
Jacaratia_spinosa_Ycf1	AATTAGTAAT CAGATGATTC ATGAATCGTC TGTTCAAATT CGATCTATGG AGTGGACAAA
Vasconcellea_pubescens_Ycf1	AATGAGTAAT CAGATGATTC ATGAATCGTC TGTTCAAATT CGATCTATGG AGTGGAAAAA
Jarilla_caudata_Ycf1	AATTAGTAAT CAGATGATTC ATGAATCGTC TGTTCAAATT CGATCTATGG AGTGGACAAA
Jarilla_chocola_Ycf1	AATTAGTAAT CAGATGATTC ATGAATCATC TGTTCAAATT CGATCTATGG AGTGGACAAA
Jarilla_heterophella_Ycf1	AATTAGTAAT CAGATGATTC ATGAATCGTC TGTTCAAATT CGATCTATGG AGTGGACAAA

	....|....| ....|....| ....|....| ....|....| ....|....| ....|....|
	3125 3135 3145 3155 3165 3175
Carica_papaya_Ycf1	TTATTCCCTG ACAGAAAACA AAATAAAAGA TTTGACTGAT AGAGCAAGTA CAATCAGAAA
Vasconcellea_Monoica_Ycf1	TTATTCCCTG ACGGAAAACA AAATAAAAGA TTTGACTGAT AGAGCAAGCA CAATCAGAAA

Jacaratia_spinosa_Ycf1 TTATTCCCTG ACAGAAAACA AAATAAAAGA TTTGACTGAT AGAGCAAGCA CAATCAGAAA

Vasconcellea_pubescens_Ycf1 TTATTCCCTG ACGGAAAACA AAATAAAAGA TTTGACTGAT AGAGCAAGCA CAATCAGAAA

Jarilla_caudata_Ycf1 TTATTCCCTG ACAGAAAACA AAATAAAAGA TTTGACTGAT AGAGCAAGCA CAATCAGAAA

Jarilla_chocola_Ycf1 TTATTCCCTG ACAGAAAACA AAATAAAAGA TTTGACTGAT AGAGCAAGCA CAATCAGAAA

Jarilla_heterophella_Ycf1 TTATTCCCTG ACAGAAAACA AAATAAAAGA TTTGACTGAT AGAGCAAGCA CAATCAGAAA

....|....| ....|....| ....|....| ....|....| ....|....| ....|....|

3185 3195 3205 3215 3225 3235

Carica_papaya_Ycf1 TCAAATAGAA CAAATTACAA AAGAGAAGAA AAACGGATTT ATAACCCAAA CTCAAGAAAT

Vasconcellea_Monoica_Ycf1 TCAAATAGAA CAAATTACAA AAGAGAAGAA AAATGGATTT ATAACCCAAA CTCAAGAAAT

Jacaratia_spinosa_Ycf1 TCAAATAGAA CAAATTACAA AAGAGAAGCA AAACGGATTT ATAACCCAAA CTCAAGAAAT

Vasconcellea_pubescens_Ycf1 TCAAATAGAA CAAATTACAA AAGAGAAGAA AAACGGATTT ATAACCCAAA CTCAAGAAAT

Jarilla_caudata_Ycf1 TCAAATAGAA CAAATTACAA AAGAGAAGAA AAACGGATTT GTAACCCAAA CTCAAGAAAT

Jarilla_chocola_Ycf1 TCAAATAGAA CAAATTACAA AAGAGAAGAA AAACGGATTT GTAACCCAAA CTCAAGAAAT

Jarilla_heterophella_Ycf1 TCAAATAGAA CAAATTACAA AAGAGAAGAA AAACGGATTT GTAACCCAAA CTCAAGAAAT

....|....| ....|....| ....|....| ....|....| ....|....| ....|....|

3245 3255 3265 3275 3285 3295

Carica_papaya_Ycf1 AAATATTAGT TCTAACAAAC CAAGTTATCA TGCTAAAATA TTAGCATCAT CAAAAAATAT

Vasconcellea_Monoica_Ycf1 AAATATGAGT TCTAACAAAC CAAGTTATGA TGCTAAAATA TTAGCATCAT CAAAAAATAT

Jacaratia_spinosa_Ycf1 AAATATTAGT TCTAACAAAC CAAGTTATCA TGCTAAACTA TTAGCATCAT CAAAAAATAT

Vasconcellea_pubescens_Ycf1 AAATATTAGT TCTAACAAAC CAAGTTATCA TGCTAAAATA TTAGCATCAT CAAAAAATAT

Jarilla_caudata_Ycf1 AAATATTAGT TCTAACAAAC CAAGTTATCA TGCTAAAATA TTAGCATCAT CAAAAAATAT

Jarilla_chocola_Ycf1 AAATATTAGT TCTAACAAAC CAAGTTATCA TGCTAAAATA TTAGCATCAT CAAAAAATAT

Jarilla_heterophella_Ycf1 AAATATTAGT TCTAACAAAC CAAGTTATCA TGCTAAAATA TTAGCATCAT CAAAAAATAT

....|....| ....|....| ....|....| ....|....| ....|....| ....|....|

3305 3315 3325 3335 3345 3355

Carica_papaya_Ycf1 TTGGCAAATA TTAAAAAGAA AAAATGCTCG ATTAATTCGT AAATCATATT ATTTTATAAA

Vasconcellea_Monoica_Ycf1 TTGGCAAATA TTAAAAAGAA AAAATACTCG ATTAATTCGT AAATCATATT ATTTTATAAA

Jacaratia_spinosa_Ycf1 TTGGCAAATA TTAAAAAGAA AAAATGCTCG ATTAATTCGT AAATCATATT ATTTTATAAA

Vasconcellea_pubescens_Ycf1 TTGGCAAATA TTAAAAAGAA AAAATGCTCG ATTAATTCGT AAATCATATT ATTTTATAAA

Jarilla_caudata_Ycf1 TTGGCAAATA TTAAAAAGAA AAAATGCTCG ATTAATTCGT AAATCATATT ATTTTATAAA

Jarilla_chocola_Ycf1 TTGGCAAATA TTAAAAAGAA AAAATGCTCG ATTAATTCGT AAATCATATT ATTTTATAAA

Jarilla_heterophella_Ycf1 TTGGCAAATA TTAAAAAGAA AAAATGCTCG ATTAATTCGT AAATCATATT ATTTTATAAA

....|....| ....|....| ....|....| ....|....| ....|....| ....|....|

3365 3375 3385 3395 3405 3415

Carica_papaya_Ycf1 ATTTTTCATT GAAAGGATAT ATATAGATAT TTTTCTATGT ATCGTTAATA TTCCAAGGAT

Vasconcellea_Monoica_Ycf1 ATTTTTCAGT GAAAGGATAT ATATAGATAT TTTTCTATGT ATCGTTAATA TTCCAAGGAT

Jacaratia_spinosa_Ycf1 ATTTTTCATT GAAAGGATAT ATATAGATAT TTTTCTATGT ATCGTTAATA TTCCAAGGAT

Vasconcellea_pubescens_Ycf1 ATTTTTCAGT GAAAGGATAT ATATAGATAT TTTTCTATGT ATCGTTAATA TTCCAAGGAT

Jarilla_caudata_Ycf1 ATTTTTCATT GAAAGGATAT ATATAGATAT TTTTCTATGT ATCGTTAATA TTCTGAGGAT

Jarilla_chocola_Ycf1 ATTTTTCATT GAAAGGATAT ATAGAGATAT TTTTCTATGT ATCGTTAATA TTCCGAGGAT

Jarilla_heterophella_Ycf1 ATTTTTCATT GAAAGGATAT ATATAGATAT TTTTCTATGT ATCGTTAATA TTCTGAGGAT

....|....| ....|....| ....|....| ....|....| ....|....| ....|....|

3425 3435 3445 3455 3465 3475

Carica_papaya_Ycf1 GAATACACAA CTTTTTCTTG AATCAACAAA AAAAAATTTT GATAAATACA TTTACAATAA

Vasconcellea_Monoica_Ycf1 GAATACACAA CTTTTTGTTG AATCAACAAA AAAAATTATT GATAAATATA TTTACACTAA

Jacaratia_spinosa_Ycf1 GAATACACAA CTTTTTCTTG AATCAACAAA AAAAATTATT GATAAATACA TTTACAATAC

Vasconcellea_pubescens_Ycf1 GAATACACAA CTTTTTGTTG AATCAACAAA AAAAATTATT GATAAATATA TTTACACTAA

Jarilla_caudata_Ycf1 GAATACACAA CTTTTTTTTG AATCAACAAA CAAAATTATT GATAAATACA TTTACAATAA

Jarilla_chocola_Ycf1 GAATACACAA CTTTTTTTTG AATCAACAAA CAAAATTATT GATAAATACA TTTACAATAA

Jarilla_heterophella_Ycf1 GAATACACAA CTTTTTTTTG AATCAACAAA CAAAATTATT GATAAATACA TTTACAATAA

....|....| ....|....| ....|....| ....|....| ....|....| ....|....|

3485 3495 3505 3515 3525 3535

Carica_papaya_Ycf1 TGAAGTAAAT CAAGAAAGAA TTAATAAAAC AAA------T AAAAATACCA GTCAATTTAT

Vasconcellea_Monoica_Ycf1 TGAAGTAAAT CAAGAAAGAA TTAATAAAAC AAA------T AAAAATACCA TTCAATTTAT

Jacaratia_spinosa_Ycf1 TGAAATAAAT CAAGAAAGAA TTAATAAAAC AAA------T AAAAATACCA TTCAATTTAT

Vasconcellea_pubescens_Ycf1 TGAAGTAAAT CAAGAAAGAA TTAATAAAAC AAA------T AAAAATACCA TTCAATTTAT

Jarilla_caudata_Ycf1 TGAAGTAAAT CAAGAAAGAA TTAATAAAAC AAAAACAAAT AAAAATACCA TTCAATTTAT

Jarilla_chocola_Ycf1 TGAAGTAAAT CAAGAAAGAA TTAATAAAAC AAAAACAAAT AAAAATACCA TTCAATTTAT

Jarilla_heterophella_Ycf1 TGAAGTAAAT CAAGAAAGAA TTAATAAAAC AAAAACAAAT AAAAATACCA TTCAATTTAT

....|....| ....|....| ....|....| ....|....| ....|....| ....|....|

3545 3555 3565 3575 3585 3595

Carica_papaya_Ycf1 TTCGAATATA AAAAACTCAC TTTCTAATAC TAATATTAGA AATAAGAATT CAAAAAATTG

Vasconcellea_Monoica_Ycf1 TTGGAATATA AAAAATTCAC TTTCTAATAC TAATATTAGA AATAAGAATT CACAAAATTG

Jacaratia_spinosa_Ycf1 TTGGAATATA AAAAACTCAC TTTCTAATAC TAATATTAGA AATAAGAATT CACAAAATTG

Vasconcellea_pubescens_Ycf1 TTGGAATATC AAAAATTCAC TTTCTAATAC TAATATTAGA AATAAGAATT CACAAAATTG

Jarilla_caudata_Ycf1 TTCGAATATA AAAAATTCAC TTTCTAATAC TAATATTCGA AATAAGAATT CACAAAATTC

Jarilla_chocola_Ycf1 TTCGAATATA AAAAACTCAC TTTCTAATAC TAATATTCGA AATAAGAATT CACAAAATTC

Jarilla_heterophella_Ycf1 TTCGAATATA AAAAACTCAC TTTCTAATAC TAATATTCGA AATAAGAATT CACAAAATTC

....|....| ....|....| ....|....| ....|....| ....|....| ....|....|

3605 3615 3625 3635 3645 3655

Carica_papaya_Ycf1 TTGTAATCTA TCCTCCTTCT CACAAGCATA TGTATTTTAC AAATTATCAC AAATAAAAGT

Vasconcellea_Monoica_Ycf1 TTGTAATCTA TCCTCTTTCT CACAAGCAGA TGTATTTTAC AAATTATTAC AACTAAAAGT

Jacaratia_spinosa_Ycf1 GTGTAATCTA TCCTCCTTCT CACAAGCATA TGTATTTTAC AAATTATCAC AAATAAAAGT

Vasconcellea_pubescens_Ycf1 TTGTAATCTA TCCTCTTTCT CACAAGCATA TGTATTTTAC AAATTATTAC AACTAAAAGT

Jarilla_caudata_Ycf1 TTGTAAGCTA TCCTCCTTCT CACAAGCATA TGTATTTTTT AAATTATCAC AAATAAAAGT

Jarilla_chocola_Ycf1 TTGTAAGCTA TCCTCCTTCT CACAAGCATA TGTATTTTTT AAATTATCAC AAATAAAAGT

Jarilla_heterophella_Ycf1 TTGTAAGCTA TCCTCCTTCT CACAAGCATA TGTATTTTTT AAATTATCAC AAATAAAAGT

....|....| ....|....| ....|....| ....|....| ....|....| ....|....|

3665 3675 3685 3695 3705 3715

Carica_papaya_Ycf1 TATTAACTTG TATAAGTTAA GGTCTGTCCT TCAATATCAA TATAACGGAA CATCTTTTTT
Vasconcellea_Monoica_Ycf1 TATTAACTTG TATAAGTTAA GGTCTGTCCT TCAATATCAC TATAACGGAA CATCTTTTTT
Jacaratia_spinosa_Ycf1 TATTAACTTG TATAAGTTAA GGTCTGTCCT TCAATATCAC TATAACGGAA CATCTTTTTT
Vasconcellea_pubescens_Ycf1 TATTAACTTG TATAAGTTAA GATCTGTCCT TCAATATCAC TATAACGGAA CATCTTTTTT
Jarilla_caudata_Ycf1 TATTAACTTG GATAAGTTAA GGTCTGTCCT TCAATATCAA TATAACGGAA CATCGTTTTT
Jarilla_chocola_Ycf1 TATTAACTTG GATAAGTTAA GGTCTGTCCT TCAATATCAA TATAACGGAA CATCGTTTTT
Jarilla_heterophella_Ycf1 TATTAACTTG GATAAGTTAA GGTCTGTCCT TCAATATCAA TATAACGGAA CATCGTTTTT

....|....| ....|....| ....|....| ....|....| ....|....| ....|....|
3725 3735 3745 3755 3765 3775
Carica_papaya_Ycf1 TCTTAAGAAT GAAATAAAGG ATTCTTTTGA AGAACAAGGA ATATTTGATT ACCAATTAAG
Vasconcellea_Monoica_Ycf1 TCTTAAGAAT GAAATAAAGG ATTTTTTTGA AGAACAAGGA ATATTTGATT ACAAATTAAG
Jacaratia_spinosa_Ycf1 TCTTAAGAAT GAAATAAAGG GTTCTTTTGA AGAACAAGGA ATATTTGATT ACAAATTAAG
Vasconcellea_pubescens_Ycf1 TCTTAAGAAT GAAATAAAGG ATTTTTTTGA AGAACAAGGA ATATTTGATT ACAAATTAAG
Jarilla_caudata_Ycf1 TCTTAAGAAT GAAATAAAGG ATTCTTTTGA AGAACAAGGA ATATTTGATT ACAAATTAAG
Jarilla_chocola_Ycf1 TCTTAAGAAT GAAATAAAGG ATTCTTTTGA AGAACAAGGA ATATTTGATT ACAAATTAAG
Jarilla_heterophella_Ycf1 TCTTAAGAAT GAAATAAAGG ATTCTTTTGA AGAACAAGGA ATATTTGATT ACAAATTAAG

....|....| ....|....| ....|....| ....|....| ....|....| ....|....|
3785 3795 3805 3815 3825 3835
Carica_papaya_Ycf1 ACATAAACCC CCTTTTAATT ACGAAAAAAA TCTATGGAAA AACTGGTTAA GAAGTCATTA
Vasconcellea_Monoica_Ycf1 ACATAAACCC TTTTTTAATC ACGAAAAAAA TCTATGGAAA AACTGGTTAA GAAGTCATTA
Jacaratia_spinosa_Ycf1 ACATAAACCC CTTTTTAATC ACGAAAAAAA TCTATGGAAA AACTGGTTAA GAAGTCATTA
Vasconcellea_pubescens_Ycf1 ACATAAACCC TTTTTTAATC ACGAAAAAAA TCTATGGAAA AACTGGTTAA GAAGTCATTA
Jarilla_caudata_Ycf1 ACATAAACCC CCTTTTAATT ACGAAAAGAA TCTATGGAAA AACTGGTTAA GAAGTCATTA
Jarilla_chocola_Ycf1 ACATAAACCC CCTTTTAATT ACGAAAAGAA TCTATGGAAA AACTGGTTAA GAAGTCATTA
Jarilla_heterophella_Ycf1 ACATAAACCC CCTTTTAATT ACGAAAAGAA TCTATGGAAA AACTGGTTAA GAAGTCATTA

....|....| ....|....| ....|....| ....|....| ....|....| ....|....|
3845 3855 3865 3875 3885 3895
Carica_papaya_Ycf1 TCAATATGAT TTACCTCAGA TTAGATGGCC TAGATTAGTA CCAGAAAAAT GGCGAAATAG
Vasconcellea_Monoica_Ycf1 TCAATATGAT TTACCTCAGA TTAGATGGCC TAGATTAGTA CCAGAAAAAT GGCGAAATAG
Jacaratia_spinosa_Ycf1 TCAATATGAT TTACCTCAGA TTAGATGGCC TAGATTAGTC CCAGAAAAAT GGCGAAATAG
Vasconcellea_pubescens_Ycf1 TCAATATGAT TTACCTCAGA TTAGATGGCC TAGATTAGTA CCAGAAAAAT GGCGAAATAG
Jarilla_caudata_Ycf1 TCAATATGAT TTACCTCAGA TTAGATGGCC TAGATTAGTA CCAGAAAAAT GGCGAAATAG
Jarilla_chocola_Ycf1 TCAATATGAT TTACCTCAGA TTAGATGGCC TAGATTAGTA CCAGAAAAAT GGCGAAATAG
Jarilla_heterophella_Ycf1 TCAATATGAT TTACCTCAGA TTAGATGGCC TAGATTAGTA CCAGAAAAAT GGCGAAATAG

....|....| ....|....| ....|....| ....|....| ....|....| ....|....|
3905 3915 3925 3935 3945 3955
Carica_papaya_Ycf1 AGTCAAAGAA TACTGTAGAG TTCAAAATAA AGATTTAAAC AAATGGGATT CATATGAAAA
Vasconcellea_Monoica_Ycf1 AGTCAAAGAA TACTGTAGAG TTCAAAATCA AGATTTAAAC AAATGGGATT CATATGAAAA
Jacaratia_spinosa_Ycf1 AGTCAAAGAA TACTGTAGAG TTCAAAATAA AGATTTAAAC AAATGGGATT CATATGAAAA
Vasconcellea_pubescens_Ycf1 AGTCAAAGAA TACTGTAGAG TTCAAAATAA AGATTTAAAC AAATGGGATT CATATGAAAA

Jarilla_caudata_Ycf1	AGTCAAAGAA TACCGTAGAG TTCAAAATAA AGATTTAAAC AAATGGGATT CATATGAAAA
Jarilla_chocola_Ycf1	AGTCAAAGAA TACCATAGAG TTCAAAATAA AGATTTAAAG AAATGGGATT CATATGAAAA
Jarilla_heterophella_Ycf1	AGTCAAAGAA TACCGTAGAG TTCAAAATAA AGATTTAAAC AAATGGGATT CATATGAAAA
	....|....| ....|....| ....|....| ....|....| ....|....| ....|....|
	3965 3975 3985 3995 4005 4015
Carica_papaya_Ycf1	AGACAAATTA AGTCATTACG AAAGACAAAA CTCTTTTGAA GTGGACTCAT TGCTGAATCA
Vasconcellea_Monoica_Ycf1	AGACAAATTA ATTCATTACA AAAGACAAAA CTTTTTTGAA GTGGACTCAT TGCTGAATCA
Jacaratia_spinosa_Ycf1	AGACAAATTA ATTCATTACG AAAGACAAAA CTTTTTTGAA GTGGACTCAT TGCTGAATCA
Vasconcellea_pubescens_Ycf1	AGACAAATTA ATTCATTACA AAAGACAAAA CTTTTTTGAA GTGGACTCAT TGCTGAATCA
Jarilla_caudata_Ycf1	AGACAAATTA ATTCATTACG AAAAACAAAA CTTTTTTGAA GTGGACTCAT TGCTGAATCA
Jarilla_chocola_Ycf1	AGACAAATTA ATTCATTACG AAAGACAAAA CCTTTTTGAA GTGGACTCAT TGCTGAATCA
Jarilla_heterophella_Ycf1	AGACAAATTA ATTCATTACG AAAAACAAAA CTTTTTTGAA GTGGACTCAT TGCTGAATCA
	....|....| ....|....| ....|....| ....|....| ....|....| ....|....|
	4025 4035 4045 4055 4065 4075
Carica_papaya_Ycf1	AAAATCGAAT TTCAAAAAAA ATGATAGATA TGATCTTTTA TCATCTAAAT CTATTAATTC
Vasconcellea_Monoica_Ycf1	AAAATCTAAT TTCAAAAAAA ATTATAGATA TGATCTTTTA TCATCTAAAT CTATTAATTC
Jacaratia_spinosa_Ycf1	AAAATCGAAT TTAAAAAAAA ATTATAGATA TGATCTTTTA TCATCTAAAT CTATTAATTC
Vasconcellea_pubescens_Ycf1	AAAATCTAAT TTCAAAAAAA ATGATAGATA TGATCTTTTA TCATCTAAAT CTATTAATTC
Jarilla_caudata_Ycf1	AAAATCGAAT TTAAAAAAGA ATTATAGATA TGATCTTTTA TCATCTAAAT CTATTAATTC
Jarilla_chocola_Ycf1	AAAATCGAAT TTAAAAAAGA ATTATAGATA TGATCTTTTA TCATCTAAAT CTATTAATTC
Jarilla_heterophella_Ycf1	AAAATCGAAT TTAAAAAAGA ATTATAGATA TGATCTTTTA TCATCTAAAT CTATTAATTC
	....|....| ....|....| ....|....| ....|....| ....|....| ....|....|
	4085 4095 4105 4115 4125 4135
Carica_papaya_Ycf1	TAAAGACAAG ATTTTTTTTA ATTACAACAT GGATAAACAG AAATTTTTTG ATATCCTCGT
Vasconcellea_Monoica_Ycf1	TAAAGACACG GTTTTTTATA ATTACAACAT GGATAAACAG AAATTTTTTG ATATCCTCGT
Jacaratia_spinosa_Ycf1	GAAAGACAAG ATTTTTTATA ATTACAACAT GGATAAACAG AAATTTTTTG ATATCCTCGT
Vasconcellea_pubescens_Ycf1	TAAAGACACG GTTTTTGATA ATTACAACAT GGATAAACAG AAATTTTTTG ATATCCTCGT
Jarilla_caudata_Ycf1	TAAAGACAAG ATTTTGTATA ATTACAACAT GGATAAACAG AAATTCTTTG ATATCCTCGT
Jarilla_chocola_Ycf1	TAAAGACAAG ATTTTGTATA ATTACAACAT GGATAAACAG AAATTCTTTG ATACCCTCGT
Jarilla_heterophella_Ycf1	TAAAGACAAG ATTTTGTATA ATTACAACAT GGATAAACAG AAATTCTTTG ATATCCTCGT
	....|....| ....|....| ....|....| ....|....| ....|....| ....|....|
	4145 4155 4165 4175 4185 4195
Carica_papaya_Ycf1	AGGTATCCCT ATCCATAATT ATCTAGTCTC TAATTATTTC GGAGAAGATC ATATTTTTGA
Vasconcellea_Monoica_Ycf1	AGGTATTCCT AGCCATAATT ATCTAGTCTC TAATTATTTA GGAGAAGAGC ATATTTTTGA
Jacaratia_spinosa_Ycf1	AGGTATCCCT ATCCATAATT ATCTAGTCTC TAATTATTTA GGAGAAGATC ATATTTTTGA
Vasconcellea_pubescens_Ycf1	AGGTATTCCT AGCCATAATT ATCTAGTCTC TAATTATTTA GGAGAAGAGC ATATTTTTGA
Jarilla_caudata_Ycf1	AGGTATCCCT ATCCATAATT ATCTAGTCTC TAATTATTTA GGAGAAGATG ATATTTTTGA
Jarilla_chocola_Ycf1	AGGTATCCCT ATCCATAATT ATCTAGTCTC TAATTATTTA GGAGAAGATG ATATTTTTGA
Jarilla_heterophella_Ycf1	AGGTATCCCT ATCCATAATT ATCTAGTCTC TAATTATTTA GGAGAAGATG ATATTTTTGA

	....|....| ....|....| ....|....| ....|....| ....|....| ....|....|
	4205 4215 4225 4235 4245 4255
Carica_papaya_Ycf1	TAGCGAGAAA ATTCTGGATC GAAAATATTT AGATTGGAGA ATTCTCAACT TTTGTCTTAG
Vasconcellea_Monoica_Ycf1	TAGCGAGAAA ATTCTGTATC GAAAATATTT AGATTGGAGA ATTCTCAACT TTTGTCTTAG
Jacaratia_spinosa_Ycf1	TAGCGAGAAA ATTCTGGATC GAAAATATTT AGATTGGAGA ATTCTCAACT TTTGTCTTAG
Vasconcellea_pubescens_Ycf1	TAGCGAGAAA ATTCTGTATC GAAAATATTT AGATTGGAGA ATTCTCAACT TTTGTCTTAG
Jarilla_caudata_Ycf1	TAGCGAGAAA ATTATGGATC GAAAATATTT AGATTGGAGA ATTCTCAACT TTAGTCTTAG
Jarilla_chocola_Ycf1	TAGCGAGAAA ATTATGGATC GAAAATATTT AGATTGGAGA ATTCTCAACT TTAGTCTTAG
Jarilla_heterophella_Ycf1	TAGCGAGAAA ATTATGGATC GAAAATATTT AGATTGGAGA ATTCTCAACT TTAGTCTTAG

	....|....| ....|....| ....|....| ....|....| ....|....| ....|....|
	4265 4275 4285 4295 4305 4315
Carica_papaya_Ycf1	AAACAAGGTC GATATTGAGT CGTGGGTTGA TATCGATACT AAGAGTAATA AAAATATTAA
Vasconcellea_Monoica_Ycf1	AAACAAAGTC GATATTGAGT CTTGGGTTGA TATCGATACT AAGAGTAATA AAAATATTAA
Jacaratia_spinosa_Ycf1	AAACAAAGTC GATATTGAGT CTTGGGTTGA TATCGATACT AAGAGTAATA AAAATATTAA
Vasconcellea_pubescens_Ycf1	AAACAAAGTC GATATTGAGT CTTGGGTTGA TATCGATACT AAGAGTAATA AAAATATTAA
Jarilla_caudata_Ycf1	AAACAAGGTC GATATTGAGT CTTGGGTTGA TATCGATACT AAGAGTAATA CAAATATTAA
Jarilla_chocola_Ycf1	AAACAAGGTC GATATTGAGT CTTGGGTTGA TATCGATACT AAGAGTAATA CAAATATTAA
Jarilla_heterophella_Ycf1	AAACAAGGTC GATATTGAGT CTTGGGTTGA TATCGATACT AAGAGTAATA CAAATATTAA

	....|....| ....|....| ....|....| ....|....| ....|....| ....|....|
	4325 4335 4345 4355 4365 4375
Carica_papaya_Ycf1	GACTGGAGTT AATAATTATC AAATTATTGA TAAAATTAAT AAGAA----- ----GAAGGG
Vasconcellea_Monoica_Ycf1	GACTGGAGTT AATAATTATC AAATTCTTGA TAAAATTAAT AAGAA----- ----GAAGGG
Jacaratia_spinosa_Ycf1	GACTGGAGTT AATAATTATC AAATTATTGA TAAAATTAAT AAGAATAATA AGAAGAAGGG
Vasconcellea_pubescens_Ycf1	GACTGGAGTT AATAATTATA AAATTCTTGA TAAAATTAAT AAGAA----- ----GAAGGG
Jarilla_caudata_Ycf1	AACTGGAGTT AAGAATTATC AAATTTTTGA TAAAATTAAT AAGAG----- ----GAAGAG
Jarilla_chocola_Ycf1	AAATGGAGTT AAGAATTATC AAATTTTTGA TAAAATTAAT AAGAG----- ----GAAGAG
Jarilla_heterophella_Ycf1	AACTGGAGTT AAGAATTATC AAATTTTTGA TAAAATTAAT AAGAG----- ----GAAGAG

	....|....| ....|....| ....|....| ....|....| ....|....| ....|....|
	4385 4395 4405 4415 4425 4435
Carica_papaya_Ycf1	TCTTTTATAT TTCACAATTC ATCAAGATCA AGAAATCAAA CCTTTCAATC CAAAAAGTTT
Vasconcellea_Monoica_Ycf1	TCTTTTTTAT TTCACAATTC ATCAAGATCA AGAATTCAAA CCATCCAATC AAAAAAATTT
Jacaratia_spinosa_Ycf1	TCTTTTTTAT TTCACAATTC ATCAAGATCA AGAAATCAAA CCTTCCAATC CAAAAAATTT
Vasconcellea_pubescens_Ycf1	TCTTTTTTAT TTCACAATTC ATCAAGATCA AGAATTCAAA CCATCCAATC AAAAAAATTT
Jarilla_caudata_Ycf1	TCTTCTTTAT TTCACAATTC ATCAAGATCA AGAAATCAAA CCTTCCAATC AAAAAAGGAA
Jarilla_chocola_Ycf1	TCTTCTTTAT TTCACAATTC ATCAAGATCA AGAAATCAAA CCTTCCAATC CAAAAAGTTC
Jarilla_heterophella_Ycf1	TCTTCTTTAT TTCACAATTC ATCAAGATCA AGAAATCAAA CCTTCCAATC AAAAAAGGAA

	....|....| ....|....| ....|....| ....|....| ....|....| ....|....|
	4445 4455 4465 4475 4485 4495
Carica_papaya_Ycf1	CTTTTTTGAT TGGATGGGAA TGAATGAAGA AATACTAAGT CGTCTATCGA ATCTAGAACT
Vasconcellea_Monoica_Ycf1	ATTTTTTGAT TGGATGGGAA TGAATGAAGA AATACTAAGT TGTCTATCGA ATCTAGAATT

Jacaratia_spinosa_Ycf1 CTTTTTTGAT TGGATGGGAA TGAATGAAGA AATACTAAGT CGTCTATCGA ATCTAGAATT

Vasconcellea_pubescens_Ycf1 ATTTTTTGAT TGGATGGGAA TGAATGAAGA AATACTAAGT TGTCTATCGA ATCTAGAATT

Jarilla_caudata_Ycf1 CTTTTTGGAT TGGATGGGAA TGAATGAAGA AATACTAAGT CGTCTATCGA ATCTAGAACT

Jarilla_chocola_Ycf1 CTTTTTTGAT TGGATGGGAA TGAATGAAGA AATACTAAGT CGTCTATCGA ATCTAGAACT

Jarilla_heterophella_Ycf1 CTTTTTGGAT TGGATGGGAA TGAATGAAGA AATACTAAGT CGTCTATCGA ATCTAGAACT

....|....| ....|....| ....|....| ....|....| ....|....| ....|....|

4505 4515 4525 4535 4545 4555

Carica_papaya_Ycf1 TTGGTTCTTT CCCGAATTTG TCTTACTTTA TAATGCATAT AAGATTAAAC CGTGGATCAT

Vasconcellea_Monoica_Ycf1 TTGGTTCTTT CCCGAATTTG TCTTACTTTA TAATGCATAT AAGATTAAAC CGTGGATCAT

Jacaratia_spinosa_Ycf1 TTGGTTCTTT CCCGAATTTG TCTTACTTTA TAATGCATAT AAGATTAAAC CGTGGATCAT

Vasconcellea_pubescens_Ycf1 TTGGTTCTTT CCCGAATTTG TCTTACTTTA TAATGCATAT AAGATTAAAC CGTGGATCAT

Jarilla_caudata_Ycf1 TTGGTTCTTT CCCGAATTTG TCTTACTTTA TAATGCATAT AAGATTAAAC CGTGGATCAT

Jarilla_chocola_Ycf1 TTGGTTCTTT CCCGAATTTG TCTTACTTTA TAATGCATAT AAGATTAAAC CGTGGATCAT

Jarilla_heterophella_Ycf1 TTGGTTCTTT CCCGAATTTG TCTTACTTTA TAATGCATAT AAGATTAAAC CGTGGATCAT

....|....| ....|....| ....|....| ....|....| ....|....| ....|....|

4565 4575 4585 4595 4605 4615

Carica_papaya_Ycf1 ACCAAGCAAT TTACTTCTTT TTAATTTTAA TGAAAATGAA AACCTTAATA AAAGCATCAC

Vasconcellea_Monoica_Ycf1 ACCAAGCAAT TTACTTCTTT TTAATTTTAA TGAAAATGAA AACCTTAAGA AAAGCATTAC

Jacaratia_spinosa_Ycf1 ACCAAGCAAT TTATTTCTTT TGAATTTTAA TGAAAATGAA AACCTTAATA AAAGCATCAC

Vasconcellea_pubescens_Ycf1 ACCAAGCAAT TTACTTCTTT TTAATTTTAA TGAAAATGAA AACCTTAAGA AAAGCATTAC

Jarilla_caudata_Ycf1 ACCAAGCAAT TTACTTCTTT TTAATTTTAA TGAAAATGAA AACCTTAATA AAAGCATCAC

Jarilla_chocola_Ycf1 ACCAAGCAAT TTACTTCTTT TTAATTTTAA TGAAAATGAA AACCTTAATA AAAGCATCAC

Jarilla_heterophella_Ycf1 ACCAAGCAAT TTACTTCTTT TTAATTTTAA TGAAAATGAA AACCTTAATA AAAGCATCAC

....|....| ....|....| ....|....| ....|....| ....|....| ....|....|

4625 4635 4645 4655 4665 4675

Carica_papaya_Ycf1 GGGAAAGAAA AAGGGTTTTA TATCATCGAA TGAAAAACAA TTTCTTGGAT TTGGATTCGA

Vasconcellea_Monoica_Ycf1 TGGAACGAAA AAGGGTTTTA TAGCATCGAA TGAAAAACAA TTTCTTGGAT TTGGATTCGA

Jacaratia_spinosa_Ycf1 TGGAAAGAAA AAGGGTTTTA TATCATCGAA TGAAAAACAA TTTCTTGGAT TTGGATTCGA

Vasconcellea_pubescens_Ycf1 TGGAACGAAA AAGGGTTTTA TAGCATCGAA TGAAAAACAA TTTCTTGGAT TTGGATTCGA

Jarilla_caudata_Ycf1 TGGAAAGAAA AAGGATTTTA TATCATCGAA TGAAAAACAA TTTCTTGGAT TTGGATTAGA

Jarilla_chocola_Ycf1 TGGAAAGAAA AAGGATTTTA TATCATCGAA TGAAAAACAA TTTCCTGGAT TTGGATTAGA

Jarilla_heterophella_Ycf1 TGGAAAGAAA AAGGATTTTA TATCATCGAA TGAAAAACAA TTTCTTGGAT TTGGATTAGA

....|....| ....|....| ....|....| ....|....| ....|....| ....|....|

4685 4695 4705 4715 4725 4735

Carica_papaya_Ycf1 AAATCCAAAT CACGAAGAAA GAGACCCCGC GTACCAAGGG GAGCTTGGAC CAGATGAACA

Vasconcellea_Monoica_Ycf1 AAATCCAAAT CAAGAAGAAA GAGACCCCAC ATACCAAGGG GAGCTTGGAT CAGATGAACA

Jacaratia_spinosa_Ycf1 AAATCCAAAT CAAGAAGAAG GAGACCCCAC ATACCAAGGG GAGCTTGGAT CAGATGAACA

Vasconcellea_pubescens_Ycf1 AAATCCAAAT CAAGAAGAAA GAGACCCCAC ATACCAAGGG GAGCTTGGAT CAGATGAACA

Jarilla_caudata_Ycf1 AAATCCAAAG CAAGAAGAAA GAGACCCCGC ATATCAAGGG GAGCTTGGAT CAGATGAACA

Jarilla_chocola_Ycf1 AAATCCAAAT CAAGAAGAAA GAGACCCCGC ATATCAAGGG GAGCTTGGAT CAGATGAACA

Jarilla_heterophella_Ycf1 AAATCCAAAG CAAGAAGAAA GAGACCCCGC ATATCAAGGG GAGCTTGGAT CAGATGAACA

....|....| ....|....| ....|....| ....|....| ....|....| ....|....|

4745 4755 4765 4775 4785 4795

Carica_papaya_Ycf1 AAAACAAGGG AATCTTGGAT CAATTCTCTC AAACCAAGAA AAAAATATTG AAGAAGATTA

Vasconcellea_Monoica_Ycf1 AAAACAAGGA AATCTTGGAT CCATTCTCTC AAATCAAGAA ACAACTATTG AAGAAGATTA

Jacaratia_spinosa_Ycf1 AAAACAAGGA AATCTTGGAT CAATTCTCTC AAACCAAGAA AAAAATATTG AAGAAGATTA

Vasconcellea_pubescens_Ycf1 AAAACAAGGA AATCTTGGAT CCATTCTCTC AAATCAAGAA ACAACTATTG AAGAAGATTA

Jarilla_caudata_Ycf1 AAAACAAGGA AATCTTGGAT CAATTCTCTC AAACCAAGAA AAAAATATTG AAGAAGATTA

Jarilla_chocola_Ycf1 AAAACAAGGA AATCTTGGAT CAATTCTCTC AAACCAAGAA AAAAATATTG AAGAAGATTA

Jarilla_heterophella_Ycf1 AAAACAAGGA AATCTTGGAT CAATTCTCTC AAACCAAGAA AAAAATATTG AAGAAGATTA

....|....| ....|....| ....|....| ....|....| ....|....| ....|....|

4805 4815 4825 4835 4845 4855

Carica_papaya_Ycf1 TGCAGAATCA GACA------ ---------- ---------- ----TCAAAA AACGTAGAAA

Vasconcellea_Monoica_Ycf1 TGCAGAATCA GACA------ ---------- ---------- ----TAAAAA AACGTAGAAA

Jacaratia_spinosa_Ycf1 TGCAGAATCA GACA------ ---------- ---------- ----TAAAAA AACGTAGAAA

Vasconcellea_pubescens_Ycf1 TGCGGAATCA GACA------ ---------- ---------- ----TAAAAA AACGTAGAAA

Jarilla_caudata_Ycf1 TGCAGAATCA GACAATATTG AAGAAGATTA TGCAGAATCA GACATAAAAA AACGTAGAAA

Jarilla_chocola_Ycf1 TGCAGAATCA GACAATATTG AAGAAGATTA TGCAGAATCA GACATAAAAA AACGTAGAAA

Jarilla_heterophella_Ycf1 TGCAGAATCA GACAATATTG AAGAAGATTA TGCAGAATCA GACATAAAAA AACGTAGAAA

....|....| ....|....| ....|....| ....|....| ....|....| ....|....|

4865 4875 4885 4895 4905 4915

Carica_papaya_Ycf1 GAAAAAGCAA TACAAAAGCA ATACAGAAGC AGAACTTCAT TTCTTCCTAA AAAGATATTT

Vasconcellea_Monoica_Ycf1 GAAAAAGCAA TACAAAAGCA ATACCGAAGC AGAACTTCAT TTCTTCCTAA AAAGATATTT

Jacaratia_spinosa_Ycf1 GAAAAAGCAA TACAAAAGCA ATACCGAAGC AGAACTTCAT TTCTTCCTAA AAAGATATTT

Vasconcellea_pubescens_Ycf1 GAAAAAGCAA TACAAAAGCA ATACCGAAGC AGAACTTCAT TTCTTCCTAA AAAGATATTT

Jarilla_caudata_Ycf1 GAAAAACCAA TACAAAAACA ATACAGAAGC AGAACTTTAT TTCTTCCTAA AAAGATATTT

Jarilla_chocola_Ycf1 GAAAAACCAA TACAAAAACA ATACAGAAGC AGAACTTTAT TTCTTCCTAA AAAGATATTT

Jarilla_heterophella_Ycf1 GAAAAACCAA TACAAAAACA ATACAGAAGC AGAACTTTAT TTCTTCCTAA AAAGATATTT

....|....| ....|....| ....|....| ....|....| ....|....| ....|....|

4925 4935 4945 4955 4965 4975

Carica_papaya_Ycf1 GCGTTTTCAA TTGAGATGGA ATGATTCTTT AAATGAAAGA ATTATCAATA ATATCAAAGT

Vasconcellea_Monoica_Ycf1 GCGTTTTCAA TTGAGATGGA ATGATTCTTT AAATCACAGA ATTATCAATA ATATCAAAGT

Jacaratia_spinosa_Ycf1 GCGTTTTCAA TTGAGATGGA ATGATTCTTT AAATCAAAGA ATTATCAATA ATATCAAAGT

Vasconcellea_pubescens_Ycf1 GCGCTTTCAA TTGAGATGGA ATGATTCTTT AAATCACAGA ATTATCAATA ATATCAAAGT

Jarilla_caudata_Ycf1 GCGTTTTCAA TTGAGATGGA ATGATTCTTT AAATGAAAGA ATTATCAATA ATATCAAAGT

Jarilla_chocola_Ycf1 GCGTTTTCAA TTGAGATGGA ATGATTCTTT AAATGAAAGA ATTATCAATA ATATCAAAGT

Jarilla_heterophella_Ycf1 GCGTTTTCAA TTGAGATGGA ATGATTCTTT AAATGAAAGA ATTATCAATA ATATCAAAGT

....|....| ....|....| ....|....| ....|....| ....|....| ....|....|

4985 4995 5005 5015 5025 5035

Carica_papaya_Ycf1	ATATTGTCTC TTGCTTAGAC TGATAAATCC AAGAGAAATT ACTATATCTT CTATTCAAAG
Vasconcellea_Monoica_Ycf1	ATATTGTCTC TTGCTTAGAC TGATAAATCC AAGAGAAATT ACTATATCTT CTATTCAAAG
Jacaratia_spinosa_Ycf1	ATATTGTCTC TTGCTTAGAC TGATAAATCC AAGAGAAATT ACTATATCTT CTATTCAAAG
Vasconcellea_pubescens_Ycf1	ATATTGTCTC TTGCTTAGAC TGATAAATCC AAGAGAAATT ACTATATCTT CTATTCAAAG
Jarilla_caudata_Ycf1	ATATTGTCTC TTGCTTAGAC TGATAAATCC AAGAGAAATT GCTATATCTT CTATTCAAAG
Jarilla_chocola_Ycf1	ATATTGTCTC TTGCTTAGAC TGATAAATCC AAGAGAAATT GCTATATCTT CTATTCAAAG
Jarilla_heterophella_Ycf1	ATATTGTCTC TTGCTTAGAC TGATAAATCC AAGAGAAATT GCTATATCTT CTATTCAAAG
	....|....| ....|....| ....|....| ....|....| ....|....| ....|....|
	5045 5055 5065 5075 5085 5095
Carica_papaya_Ycf1	GGGAGAACTG AGTCTAGATA TCCTGATGAT TCAGAAGGAT TTAACTCTTC CAGAATTAAT
Vasconcellea_Monoica_Ycf1	GGGAGAAATG AGTCTAGATA TCCTAATGAG TCAGAAGGAT TTAACTCTTC CAGAATTAAT
Jacaratia_spinosa_Ycf1	GGGAGAAATG AGTCTAGATA TCCTAATGAT TCAGAAGGAT TTAACTCTTC CAGAATTAAT
Vasconcellea_pubescens_Ycf1	GGGAGAAATG AGTCTAGATA TCCTAATGAG TCAGAAGGAT TTAACTCTTC CAGAATTAAT
Jarilla_caudata_Ycf1	GGGAGAAATG AGTCTAGATA TCCTGATGAT TCAGAAGGAT TTAACTCTTC CAGAATTAAT
Jarilla_chocola_Ycf1	GGGAGAAATG AGTCTAGATA TCCTGATGAT TCAGAAGGAT TTAACTCTTC CAGAATTAAT
Jarilla_heterophella_Ycf1	GGGAGAAATG AGTCTAGATA TCCTGATGAT TCAGAAGGAT TTAACTCTTC CAGAATTAAT
	....|....| ....|....| ....|....| ....|....| ....|....| ....|....|
	5105 5115 5125 5135 5145 5155
Carica_papaya_Ycf1	GAAAAAGGGA ATATTGATTA TCGAACCAGT CCGTTTGTCT GTCAAAAACG ATGGACAATT
Vasconcellea_Monoica_Ycf1	GAAAAAGGGA ATATTGATTA TCGAACCAGT TCGTTTGTCT GTCAAAAACG ATGGACAATT
Jacaratia_spinosa_Ycf1	GAAAAAGGGA ATATTGATTA TCGAACCAGT TCGTTTGTCT GTCAAAAACG ATGGGCAATT
Vasconcellea_pubescens_Ycf1	GAAAAAGGGA ATATTGATTA TCGAACCAGT TCGTTTGTCT GTCAAAAACG ATGGACAATT
Jarilla_caudata_Ycf1	GAAAAAGGGA ATATTGATTC TCGAACCAGT TCGTTTATCT GTCAAAAACG ATGGACAATT
Jarilla_chocola_Ycf1	GAAAAAGGGA ATATTGATTC TCGAACCAGT TCGTTTATCT GTCAAAAACG ATGGACAATT
Jarilla_heterophella_Ycf1	GAAAAAGGGA ATATTGATTC TCGAACCAGT TCGTTTATCT GTCAAAAACG ATGGACAATT
	....|....| ....|....| ....|....| ....|....| ....|....| ....|....|
	5165 5175 5185 5195 5205 5215
Carica_papaya_Ycf1	TATTATATAT CAAACCATAG GTATTTCATT GGGTGATAAG AATAAGCCCC AAATAAAGCA
Vasconcellea_Monoica_Ycf1	TATTATATAT CAAACCATAA GTATTTCATT GGTTGATAAG AATAAGCACC AAATAAAGCA
Jacaratia_spinosa_Ycf1	TATTATATAT CAAACCATAG GTATTTCATT GGTTGATAAG AATAAGCACC AAATAAAGCA
Vasconcellea_pubescens_Ycf1	TATTATATAT CAAACCATAA GTATTTCATT GGTTGATAAG AATAAGCACC AAATAAAGCA
Jarilla_caudata_Ycf1	TATTATCTAT CAAACCATAG GTATTTCATT GGTTAATACG AATAAGCAAC AAATAAAGCA
Jarilla_chocola_Ycf1	TATTATCTAT CAAACCATAG GTATTTCATT GGTTGATACG AATAAGCAAC AAATAAAGCA
Jarilla_heterophella_Ycf1	TATTATCTAT CAAACCATAG GTATTTCATT GGTTAATACG AATAAGCAAC AAATAAAGCA
	....|....| ....|....| ....|....| ....|....| ....|....| ....|....|
	5225 5235 5245 5255 5265 5275
Carica_papaya_Ycf1	AAGATACCAA GAAAAAAACT ATGTTGATAA AAAGAATTTT GATGAATCCA TTGCAAGACA
Vasconcellea_Monoica_Ycf1	AAGATACCAA GAAAAAAACT ATGTTGATAA AAATCATTTT GATGAATCCA TTGCAAGATA
Jacaratia_spinosa_Ycf1	AAGATACCAA GAAAAAAACT ATGTTGATAA AAAGAATTTT GATGAATCCA TTGCAAGACA
Vasconcellea_pubescens_Ycf1	AAGATACCAA GAAAAAAACT ATGTTGATAA AAATCATTTT GATGAATCCA TTGCAAGACA

Jarilla_caudata_Ycf1 AAGATACCAA GAAAAAAACT ATGTTGATAA AAATCATTTT GATGAATCCA TTGCAAGACA
Jarilla_chocola_Ycf1 AAGATACCAA GAAAAAAACT ATGTTGATAA AAATCATTTT GATGAATCCA TTGCAAGACA
Jarilla_heterophella_Ycf1 AAGATACCAA GAAAAAAACT ATGTTGATAA AAATCATTTT GATGAATCCA TTGCAAGACA

....|....| ....|....| ....|....| ....|....| ....|....| ....|....|
5285 5295 5305 5315 5325 5335

Carica_papaya_Ycf1 TCAAAGAATG ACTGGAAATA GAGAAAAAAA TCATTATGAT TTCCTTGTCC CTGAAAATAT
Vasconcellea_Monoica_Ycf1 TCAAAGACTG ACTGGAAATA GAGAAAAAAA TCATTATGAT TTCCTTGTCC CGGAAAAGAT
Jacaratia_spinosa_Ycf1 TCAAAGAATG ACTGGAAATA GAGAAAAAAA TCATTATGAT TTCCTTGTCC CTGAAAATAT
Vasconcellea_pubescens_Ycf1 TCAAAGACTG ACTGGCAATA GAGAAAAAAA TCATTATGAT TTCCTTGTCC CGGAAAAGAT
Jarilla_caudata_Ycf1 TCAAAGAATG ACTGGAAATA GAGAAAAAAA TCATTATGAT TTCCTTGTCC CTGAAAATAT
Jarilla_chocola_Ycf1 TCAAAGAATG ACTGGAAATA GAGAAAAAAA TCATTATGAT TTCCTTGTCC CTGAAAATAT
Jarilla_heterophella_Ycf1 TCAAAGAATG ACTGGAAATA GAGAAAAAAA TCATTATGAT TTCCTTGTCC CTGAAAATAT

....|....| ....|....| ....|....| ....|....| ....|....| ....|....|
5345 5355 5365 5375 5385 5395

Carica_papaya_Ycf1 TTTATCCCCT AAATGTCGTA GAGAATTTCG AATTCTAATT TGTTTCAATT CAAAGAATAG
Vasconcellea_Monoica_Ycf1 TTTATCCCCG AAATGTCGTC GAGAATTTCG AATTCTAATT TGTTTCAACT CAAAGAATAG
Jacaratia_spinosa_Ycf1 TTTATCCCCT AAATGTCGTA GAGAATTTCG AATTCTAATT TGTTTCAATT CAAAGAATAG
Vasconcellea_pubescens_Ycf1 TTTATCCCCG AAATGTCGTC GAGAATTTCG AATTCTAATT TGTTTCAACT CAAAGAATAG
Jarilla_caudata_Ycf1 TTTATCCCCT AAATGTCGTA GAGAATTTAG AATTCTAATT TGTTTCAATT CACAGAATAG
Jarilla_chocola_Ycf1 TTTATCCCCT AAATGTCGTA GAGAATTTAG AATTCTAATT TGTTTCAATT CACAGAATAG
Jarilla_heterophella_Ycf1 TTTATCCCCT AAATGTCGTA GAGAATTTAG AATTCTAATT TGTTTCAATT CACAGAATAG

....|....| ....|....| ....|....| ....|....| ....|....| ....|....|
5405 5415 5425 5435 5445 5455

Carica_papaya_Ycf1 AAATGATATG CGTAGAAAGA CAAAATTTCA CAATAACATA AAAAACTGTG GTCAAGTTTT
Vasconcellea_Monoica_Ycf1 AAATGATATG TGTAGAAAGA CACAATTTCA CAATACCATA AAAAACGGTG GTCAAGTTTT
Jacaratia_spinosa_Ycf1 AAATGATATG CGTAGAAAGA CAAAATTTCA CAATACCATA AAAAACTGTG GTCAAGTTTT
Vasconcellea_pubescens_Ycf1 AAATGATATG CGTAGAAAGA CACAATTTCA CAATACCATA AAAAACGGTG GTCAAGTTTT
Jarilla_caudata_Ycf1 AAATGATATG TGTAGAAAGA CCAAATTTCA CAATAACATA AAAAACTGTG GTCAAGTTTT
Jarilla_chocola_Ycf1 AAATGATATG TGTAGAAAGA CCAAATTTCA CAATAACATA AAAAACTGTG GTCAAGTTTT
Jarilla_heterophella_Ycf1 AAATGATATG TGTAGAAAGA CCAAATTTCA CAATAACATA AAAAACTGTG GTCAAGTTTT

....|....| ....|....| ....|....| ....|....| ....|....| ....|....|
5465 5475 5485 5495 5505 5515

Carica_papaya_Ycf1 GAATACAACC AAAGATTTTG ATAGAAATAA AAATACCCTA ATTAAATCAA AGTTCTTTCT
Vasconcellea_Monoica_Ycf1 GAATACAAGC AAAGATTTTG ATAGAAATAA AAATAACCTA ATTAAATCAA AGTTCTTTCT
Jacaratia_spinosa_Ycf1 GAATACAAGC AAAGATTTTG ATAGAAATAA AAATAACCTA ATTAAATCAA AGTTCTTTCT
Vasconcellea_pubescens_Ycf1 GAATACAAGC AAAGATTTTG ATAGAAATAA AAATAACCTA ATTAAATCAA AGTTCTTTCT
Jarilla_caudata_Ycf1 GAATACAACC AAAGATTTTG ATAAAAATAA AAATAACCTA ATTAAATCAA AGCTCTTTCT
Jarilla_chocola_Ycf1 GAATACAACC AAAGATTTTG ATAAAAATAA AAATAACCTA ATTAAATCAA AGCTCTTTCT
Jarilla_heterophella_Ycf1 GAATACAACC AAAGATTTTG ATAAAAATAA AAATAACCTA ATTAAATCAA AGCTCTTTCT

	....|....| ....|....| ....|....| ....|....| ....|....| ....|....|
	5525 5535 5545 5555 5565 5575
Carica_papaya_Ycf1	TTGGCCCAAT TTTCGATTAG AAGATTTAGC TTGTATGAAT CGCTATTGGT TTAATACTAA
Vasconcellea_Monoica_Ycf1	TTGGCCCAAT TTTCGATTAG AAGATTTAGC TTGTATGAAT CGCTATTGGT TTAATACTAA
Jacaratia_spinosa_Ycf1	TTGGCCCAAT TTTCGATTAG AAGATTTAGC TTGTATGAAT CGCTATTGGT TTAATACTAA
Vasconcellea_pubescens_Ycf1	TTGGCCCAAT TTTCGATTAG AAGATTTAGC TTGTATGAAT CGCTATTGGT TTAATACTAA
Jarilla_caudata_Ycf1	TTGGCCCAAT TTTCGATTAG AAGATTTAGC TTGTATGAAT CGCTATTGGT TTAATACTAA
Jarilla_chocola_Ycf1	TTGGCCCAAT TTTCGATTAG AAGATTTAGC TTGTATGAAT CGCTATTGGT TTAATACTAA
Jarilla_heterophella_Ycf1	TTGGCCCAAT TTTCGATTAG AAGATTTAGC TTGTATGAAT CGCTATTGGT TTAATACTAA

	....|....| ....|....| ....|....| ....|....| ....|....| ....|....|
	5585 5595 5605 5615 5625 5635
Carica_papaya_Ycf1	TAATGGTAGT CGTTTCAGTA TGATAAGGAT ACATATGTAT CCGCGATTAA AAACTCCTTG
Vasconcellea_Monoica_Ycf1	TAATGGTAGT CGTTTCAGTA TGATAAGGAT ACATATGTAT CCGCGATTAA AAACTCCTTG
Jacaratia_spinosa_Ycf1	TAATGGTAGT CGTTTCAGTA TGATAAGGAT ACATATGTAT CCGCGATTAA AAACTCCTTG
Vasconcellea_pubescens_Ycf1	TAATGGTAGT CGTTTCAGTA TGATAAGGAT ACATATGTAT CCGCGATTAA AAACTCCTTG
Jarilla_caudata_Ycf1	TAATGGTAGT CGTTTCAGTA TGATAAGGAT ACATATGTAT CCGCGATTAA AAACTCCTTG
Jarilla_chocola_Ycf1	TAATGGTAGT CGTTTCAGTA TGATAAGGAT ACATATGTAT CCGCGATTAA AAACTCCTTG
Jarilla_heterophella_Ycf1	TAATGGTAGT CGTTTCAGTA TGATAAGGAT ACATATGTAT CCGCGATTAA AAACTCCTTG

	.
Carica_papaya_Ycf1	A
Vasconcellea_Monoica_Ycf1	A
Jacaratia_spinosa_Ycf1	A
Vasconcellea_pubescens_Ycf1	A
Jarilla_caudata_Ycf1	A
Jarilla_chocola_Ycf1	A
Jarilla_heterophella_Ycf1	A
