# Supplementary material for: Comparative analysis of chloroplast genomes in Vasconcellea pubescens A.DC. and Carica papaya L
Source: Sci Rep. 2020 Sep 25;10:15799. doi: 10.1038/s41598-020-72769-y (PMC7519098; doi:10.1038/s41598-020-72769-y)
Supplement: Supplementary file 5 — Supplementary Information 5. [file 41598_2020_72769_MOESM5_ESM.pdf]

Comparative analysis of chloroplast genomes in *Vasconcellea pubescens* A.DC. and *Carica papaya* L.

Zhicong Lin[1] , Ping Zhou[3], Xinyi Ma[2], Youjin Deng[2], Zhenyang Liao[2],

Ruoyu Li[2] and Ray Ming[4,1 *]

[1]College of Agriculture, Center for Genomics and Biotechnology, Fujian Provincial Key Laboratory of Haixia Applied Plant Systems Biology, Fujian Agriculture and Forestry University, Fuzhou, Fujian 350002, China.

[2]College of Life Sciences, Fujian Agriculture and Forestry University, Fuzhou 350002, Fujian, China.

[3]Fruit Research Institute，Fujian Academy of Agricultural Sciences，Fuzhou 350013，Fujian, China

[4]Department of Plant Biology, University of Illinois at Urbana-Champaign, Urbana, IL 61801, USA.

[*]rayming@illinois.edu

Indels and SNPs profile of *Ycf1* gene

	....|....|	....|....|	....|....|	....|....|	....|....|	....|....|
	5	15	25	35	45	55
Carica_papaya_Ycf1	ATGATTTTTA	AATCTTTTAT	ACTAGGTAAT	CTAGTATCCT	TATGCATGAA	GATAATCAAT
Vasconcellea_Monoica_Ycf1	ATGATTTTTA	AATCTTTTAT	ACTAGGTAAT	CTAGTATCCT	TATGCATGAA	GATAATCAAT
Jacaratia_spinosa_Ycf1	ATGATTTTTA	AATCTTTTAT	ACTAGGTAAT	CTAGTATCCT	TATGCATGAA	GATAATCAAT
Vasconcellea_pubescens_Ycf1	ATGATTTTTA	AATCTTTTAT	ACTAGGTAAT	CTAGTATCCT	TATGCATGAA	GATAATCAAT
Jarilla_caudata_Ycf1	ATGATTTTTA	AATCTTTTAT	ACTAGGTAAT	CTAGTATCCT	TATGCATGAA	GATAATCAAT
Jarilla_chocola_Ycf1	ATGATTTTTA	AATCTTTTAT	ACTAGGTAAT	CTAGTATCCT	TATGCATGAA	GATAATCAAT
Jarilla_heterophella_Ycf1	ATGATTTTTA	AATCTTTTAT	ACTAGGTAAT	CTAGTATCCT	TATGCATGAA	GATAATCAAT
Arabidopsis_thaliana_Ycf1	ATGGTTTTTC	AATCTTTTAT	ACTAGGTAAT	CTAGTATCCT	TATGCATGAA	GATAATAAAT

	....|....|	....|....|	....|....|	....|....|	....|....|	....|....|
	65	75	85	95	105	115
Carica_papaya_Ycf1	TCGGTCGTTG	TGGTCGGACT	CTATTATGGA	TTTCTGACCA	CATTCTCCGT	AGGGCCCTCT
Vasconcellea_Monoica_Ycf1	TCGGTCGTTG	TGGTCGGACT	CTATTATGGA	TTTCTGACCA	CATTCTCCAT	AGGGCCCTCT
Jacaratia_spinosa_Ycf1	TCGGTCGTTG	TGGTCGGACT	CTATTATGGA	TTTCTGACCA	CATTCTCCAT	AGGGCCCTCT
Vasconcellea_pubescens_Ycf1	TCGGTCGTTG	TGGTCGGACT	CTATTATGGA	TTTCTGACCA	CATTCTCCAT	AGGGCCCTCT
Jarilla_caudata_Ycf1	TCGGTCGTTG	TGGTCGGACT	CTATTATGGA	TTTCTGACCA	CATTCTCCAT	AGGGCCCTCT
Jarilla_chocola_Ycf1	TCGGTCGTTG	TGGTCGGACT	CTATTATGGA	TTTCTGACCA	CATTCTCCAT	AGGGCCCTCT
Jarilla_heterophella_Ycf1	TCGGTCGTTG	TGGTCGGACT	CTATTATGGA	TTTCTGACCA	CATTCTCCAT	AGGGCCCTCT
Arabidopsis_thaliana_Ycf1	TCGGTCGTTG	TGGTCGGACT	CTATTATGGA	TTTCTGACCA	CATTTTCCAT	AGGGCCCTCT

	....|....|	....|....|	....|....|	....|....|	....|....|	....|....|
	125	135	145	155	165	175
Carica_papaya_Ycf1	TATCTCTTTC	TTCTCCGAGC	TCGGGTTATG	GAAGAAGGAG	AAGAAGGAAC	CGAGAAGAAG
Vasconcellea_Monoica_Ycf1	TATCTCTTTC	TTCTCCGAGC	TCGGGTTATG	GAAGAAGGAG	AAGAAGGAAC	CGAGAAGAAG
Jacaratia_spinosa_Ycf1	TATCTCTTTC	TTCTCCGAGC	TCGGGTTATG	GAAGAAGGAG	AAGAAGGAAC	CGAGAAGAAG
Vasconcellea_pubescens_Ycf1	TATCTCTTTC	TTCTCCGAGC	TCGGGTTATG	GAAGAAGGAG	AAGAAGGAAC	CGAGAAGAAG
Jarilla_caudata_Ycf1	TATCTCTTTC	TTCTCCGAGC	TCGGGTTATG	GAAGAAGGAG	AAGAAGGAAC	CGAGAAGAAG
Jarilla_chocola_Ycf1	TATCTCTTTC	TTCTCCGAGC	TCGGGTTATG	GAAGAAGGAG	AAGAAGGAAC	CGAGAAGAAG
Jarilla_heterophella_Ycf1	TATCTCTTTC	TTCTCCGAGC	TCGGGTTATG	GAAGAAGGAG	AAGAAGGAAC	CGAGAAGAAG
Arabidopsis_thaliana_Ycf1	TATCTCTTCC	TTCTCCGAGC	TCGGGTTATG	GACGAAGGAG	AAGAAGGAAC	CGAGAAGAAA

	....|....|	....|....|	....|....|	....|....|	....|....|	....|....|
	185	195	205	215	225	235
Carica_papaya_Ycf1	GTATCAGCAA	CAACTGGTTT	TATTGCGGGA	CAGCTCATGA	TGTTCATATC	GATCTATTAT
Vasconcellea_Monoica_Ycf1	GTATCAGCAA	CAACTGGTTT	TATTGCGGGA	CAGCTCATGA	TGTTCATATC	GATCTATTAT
Jacaratia_spinosa_Ycf1	GTATCAGCAA	CAACTGGTTT	TATTGCGGGA	CAGCTCATGA	TGTTCATATC	GATCTATTAT
Vasconcellea_pubescens_Ycf1	GTATCAGCAA	CAACTGGTTT	TATTGCGGGA	CAGCTCATGA	TGTTCATATC	GATCTATTAT
Jarilla_caudata_Ycf1	GTATCAGCAA	CAACTGGTTT	TATTGCGGGA	CAGCTCATGA	TGTTCATATC	GATCTATTAT
Jarilla_chocola_Ycf1	GTATCAGCAA	CAACTGGTTT	TATTGCGGGA	CAGCTCATGA	TGTTCATATC	GATCTATTAT
Jarilla_heterophella_Ycf1	GTATCAGCAA	CAACTGGTTT	TATTGCGGGA	CAGCTCATGA	TGTTCATATC	GATCTATTAT

Arabidopsis_thaliana_Ycf1 GTATCAGCAA CAACAGGTTT TATTGCGGGA CAGCTCATGA TGTTCATATC GATCTATTAT

....|....| ....|....| ....|....| ....|....| ....|....| ....|....|
245 255 265 275 285 295

Carica_papaya_Ycf1 GCGCCTCTGC ATTTAGCATT GGGTAGACCT CATACAATAA CTGTCCTAGC TCTACCGTAT
Vasconcellea_Monoica_Ycf1 GCGCCTCTGC ATTTAGCATT GGGTAGACCT CATACAATAA CTGTCCTAGC TCTACCGTAT
Jacaratia_spinosa_Ycf1 GCGCCTCTGC ATTTAGCATT GGGTAGACCT CATACAATAA CTGTCCTAGC TCTACCGTAT
Vasconcellea_pubescens_Ycf1 GCGCCTCTGC ATTTAGCATT GGGTAGACCT CATACAATAA CTGTCCTAGC TCTACCGTAT
Jarilla_caudata_Ycf1 GCGCCTCTGC ATTTAGCATT GGGTAGACCT CATACAATAA CTGTCCTAGC TCTACCGTAT
Jarilla_chocola_Ycf1 GCGCCTCTGC ATTTAGCATT GGGTAGACCT CATACAATAA CTGTCCTAGC TCTACCGTAT
Jarilla_heterophella_Ycf1 GCGCCTCTGC ATTTAGCATT GGGTAGACCT CATACAATAA CTGTCCTAGC TCTACCGTAT
Arabidopsis_thaliana_Ycf1 GCGCCTCTGC ATTTAGCATT GGGTAGACCT CATACAATAA CTGTCCTAGC TCTACCGTAT

....|....| ....|....| ....|....| ....|....| ....|....| ....|....|
305 315 325 335 345 355

Carica_papaya_Ycf1 CTTTTGTTTC ATTTCTTCTG GAACAATCAC AAACACTTTT TTGATTATGG ATCTACTACC
Vasconcellea_Monoica_Ycf1 CTTTTGTTTC ATTTCTTCTG GAACAATCAC AAACACTTTT TTGATTATGG ATCTACTACC
Jacaratia_spinosa_Ycf1 CTTTTGTTTC ATTTCTTCTG GAACAATCAC AAACACTTTT TTGATTATGG ATCTACTACC
Vasconcellea_pubescens_Ycf1 CTTTTGTTTC ATTTCTTCTG GAACAATCAC AAACACTTTT TTGATTATGG ATCTACTACC
Jarilla_caudata_Ycf1 CTTTTGTTTC ATTTCTTCTG GAACAATCAC AAACACTTTT TTGATTATGG ATCTACTACC
Jarilla_chocola_Ycf1 CTTTTGTTTC ATTTCTTCTG GAACAATCAC AAACACTTTT TTGATTATGG ATCTACTACC
Jarilla_heterophella_Ycf1 CTTTTGTTTC ATTTCTTCTG GAACAATCAC AAACACTTTT TTGATTATGG ATCTACTACC
Arabidopsis_thaliana_Ycf1 CTTTTGTTTC ATTTCTTCTG GAACAATCAC AAACACTTTT TTGATTATGG ATCTACTACC

....|....| ....|....| ....|....| ....|....| ....|....| ....|....|
365 375 385 395 405 415

Carica_papaya_Ycf1 AGAAATTCAA TGCGTAATCT TCGCATTCAA TGTGTATTCC TGAATAATCT CATTTTTCAA
Vasconcellea_Monoica_Ycf1 AGAAATTCAA TGCGTAATCT TCGCATTCAA TGTGTATTCC TGAATAATCT CATTTTTCAA
Jacaratia_spinosa_Ycf1 AGAAATTCAA TGCGTAATCT TCGCATTCAA TGTGTATTCC TGAATAATCT CATTTTTCAA
Vasconcellea_pubescens_Ycf1 AGAAATTCAA TGCGTAATCT TCGCATTCAA TGTGTATTCC TGAATAATCT CATTTTTCAA
Jarilla_caudata_Ycf1 AGAAATTCAA TGCGTAATCT TCGCATTCAA TGTGTATTCC TGAATAATCT CATTTTTCAA
Jarilla_chocola_Ycf1 AGAAATTCAA TGCGTAATCT TCGCATTCAA TGTGTATTCC TGAATAATCT CATTTTTCAA
Jarilla_heterophella_Ycf1 AGAAATTCAA TGCGTAATCT TCGCATTCAA TGTGTATTCC TGAATAATCT CATTTTTCAA
Arabidopsis_thaliana_Ycf1 AGAAATGAAA TGCGTAATCT TCGCATTCAA TGTGTATTCC TGAATAATCT CATTTTTCAA

....|....| ....|....| ....|....| ....|....| ....|....| ....|....|
425 435 445 455 465 475

Carica_papaya_Ycf1 TTATTCAACC ATTTCATTTT ACCAAGTTCA ATGTTAGCCA GATTAGTCAA CATTTATATG
Vasconcellea_Monoica_Ycf1 TTATTCAACC ATTTCATTTT ACCAAGTTCA ATGTTAGCCA GATTAGTCAA CATTTATATG
Jacaratia_spinosa_Ycf1 TTATTCAACC ATTTCATTTT ACCAAGTTCA ATGTTAGCCA GATTAGTCAA CATTTATATG
Vasconcellea_pubescens_Ycf1 TTATTCAACC ATTTCATTTT ACCAAGTTCA ATGTTAGCCA GATTAGTCAA CATTTATATG
Jarilla_caudata_Ycf1 TTATTCAACC ATTTCATTTT ACCAAGTTCA ATGTTAGCCA GATTAGTCAA CATTTATATG
Jarilla_chocola_Ycf1 TTATTCAACC ATTTCATTTT ACCAAGTTCA ATGTTAGCCA GATTAGTCAA CATTTATATG
Jarilla_heterophella_Ycf1 TTATTCAACC ATTTCATTTT ACCAAGTTCA ATGTTAGCCA GATTAGTCAA CATTTATATG

Arabidopsis_thaliana_Ycf1 TTATTCAACC ATTTCATTTT ACCAAGTTCA ATGTTAGCCA GATTAGTCAA CATTTATATG

....|....| ....|....| ....|....| ....|....| ....|....| ....|....|

485 495 505 515 525 535

Carica_papaya_Ycf1 TTTCGATGCA ACAACAAGAT GTTATTTGTA ACAAGTAGTT TTGTTGGTTG GTTAATTGGT

Vasconcellea_Monoica_Ycf1 TTTCGATGCA ACAACAAGAT GTTATTTGTA ACAAGTAGTT TTGTTGGTTG GTTAATTGGT

Jacaratia_spinosa_Ycf1 TTTCGATGCA ACAACAAGAT GTTATTTGTA ACAAGTAGTT TTGTTGGTTG GTTAATTGGT

Vasconcellea_pubescens_Ycf1 TTTCGATGCA ACAACAAGAT GTTATTTGTA ACAAGTAGTT TTGTTGGTTG GTTAATTGGT

Jarilla_caudata_Ycf1 TTTCGATGCA ACAACAAGAT GTTATTTGTA ACAAGTAGTT TTGTTGGTTG GTTAATTGGT

Jarilla_chocola_Ycf1 TTTCGATGCA ACAACAAGAT GTTATTTGTA ACAAGTAGTT TTGTTGGTTG GTTAATTGGT

Jarilla_heterophella_Ycf1 TTTCGATGCA ACAACAAGAT GTTATTTGTA ACAAGTAGTT TTGTTGGTTG GTTAATTGGT

Arabidopsis_thaliana_Ycf1 TTTCGATGCA ACAACAAGAT GTTATTTGTA ACAAGTAGTT TTGTTGGTTG GTTAATTGGT

....|....| ....|....| ....|....| ....|....| ....|....| ....|....|

545 555 565 575 585 595

Carica_papaya_Ycf1 CACATTTTAT TCATGAAATG GGTTGGATTG GTATTAGTCT GGATACAGCA AAATAATTCT

Vasconcellea_Monoica_Ycf1 CACATTTTAT TCATGAAATG GGTTGGATTG GTATTAGTCT GGATACAGCA AAATAATTCT

Jacaratia_spinosa_Ycf1 CACATTTTAT TCATGAAATG GGTTGGATTG GTATTAGTCT GGATACAGCA AAATAATTCT

Vasconcellea_pubescens_Ycf1 CACATTTTAT TCATGAAATG GGTTGGATTG GTATTAGTCT GGATACAGCA AAATAATTCT

Jarilla_caudata_Ycf1 CACATTTTAT TCATGAAATG GGTTGGATTG GTATTAGTCT GGATACAGCA AAATAATTCT

Jarilla_chocola_Ycf1 CACATTTTAT TCATGAAATG GGTTGGATTG GTATTAGTCT GGATACAGCA AAATAATTCT

Jarilla_heterophella_Ycf1 CACATTTTAT TCATGAAATG GGTTGGATTG GTATTAGTCT GGATACAGCA AAATAATTCT

Arabidopsis_thaliana_Ycf1 CACATTTTAT TCATGAAATG GGTTGGATTG GTATTAGTCT GGATACAGCA AAATAATTCT

....|....| ....|....| ....|....| ....|....| ....|....| ....|....|

605 615 625 635 645 655

Carica_papaya_Ycf1 ATTAGGTCTA ATGTACTTAT TCGATCTAAT AAGTATAAGT ACCTTGTGTC AGAATTGAGA

Vasconcellea_Monoica_Ycf1 ATTAGGTCTA ATGTACTTAT TCGATCTAAT AAGTATAAAT ACCTTGTGTC AGAATTGAGA

Jacaratia_spinosa_Ycf1 ATTAGGTCTA ATGTACTTAT TCGATCTAAT AAGTATAAGT ACCTTGTGTC AGAATTGAGA

Vasconcellea_pubescens_Ycf1 ATTAGGTCTA ATGTACTTAT TCGATCTAAT AAGTATAAAT ACCTTGTGTC AGAATTGAGA

Jarilla_caudata_Ycf1 ATTAGGTCTA ATGTACTTAT TCGATCTAAT AAGTATAAGT ACCTTGTGTC AGAATTGAGA

Jarilla_chocola_Ycf1 ATTAGGTCTA ATGTACTTAT TCGATCTAAT AAGTATAAGT ACCTTGTGTC AGAATTGAGA

Jarilla_heterophella_Ycf1 ATTAGGTCTA ATGTACTTAT TCGATCTAAT AAGTATAAGT ACCTTGTGTC AGAATTGAGA

Arabidopsis_thaliana_Ycf1 ATTAGGTCTA ATGTAGTTAT TAGATCTAAT AAGTATAAGT TCCTTGTGTC AGAATTGAGA

....|....| ....|....| ....|....| ....|....| ....|....| ....|....|

665 675 685 695 705 715

Carica_papaya_Ycf1 AATTCTATGG CTCGAATCTT TAGTATTCTC TTATTTATTA CCTGTGTCTA CTATTTAGGC

Vasconcellea_Monoica_Ycf1 AATTCTATGG CTCGAATCTT TAGTATTCTC TTATTTATTA CCTGTGTCTA CTATTTAGGC

Jacaratia_spinosa_Ycf1 AATTCTATGG CTCGAATCTT TAGTATTCTC TTATTTATTA CCTGTGTCTA CTATTTAGGC

Vasconcellea_pubescens_Ycf1 AATTCTATGG CTCGAATCTT TAGTATTCTC TTATTTATTA CCTGTGTCTA CTATTTAGGC

Jarilla_caudata_Ycf1 AATTCTATGG CTCGAATCTT TAGTATTCTC TTATTTATTA CCTGTGTCTA CTATTTAGGC

Jarilla_chocola_Ycf1 AATTCTATGG CTCGAATCTT TAGTATTCTC TTATTTATTA CCTGTGTCTA CTATTTAGGC

Jarilla_heterophella_Ycf1 AATTCTATGG CTCGAATCTT TAGTATTCTC TTATTTATTA CCTGTGTCTA CTATTTAGGC

Arabidopsis_thaliana_Ycf1 AATTCTATGG CTCGAATCTT TAGTATTCTC TTATTTATTA CCTGTGTCTA CTATTTAGGC

....|....| ....|....| ....|....| ....|....| ....|....| ....|....|

725 735 745 755 765 775

Carica_papaya_Ycf1 AGAATACCGT CACCCATTTT TACTAAGAAA CTAAAAGGAA CCTCGGAAAC GGAGGAAAGG

Vasconcellea_Monoica_Ycf1 AGAATACCGT CACCCATTTT GACTAAGAAA ATAAAAGGAA TCTCAGAAAC GGAGGAAAGT

Jacaratia_spinosa_Ycf1 AGAATACCGT CACCCATTTT TACTAAGAAA ATAAAAGGAA TCTCAGAAAC GGAGGAAAGT

Vasconcellea_pubescens_Ycf1 AGAATACCGT CACCCATTTT TACTAAGAAA ATAAAAGGAA TCTCAGAAAC GGAGGAAAGT

Jarilla_caudata_Ycf1 AGAATACCGT CACCCATTTT TACTAAGAAA CTAAAAGGAA CCTCAGAAAC GGAGGAAAGG

Jarilla_chocola_Ycf1 AGAATACCGT CACCCATTTT TACTAAGAAA CTAAAAGGAA CCTCAGAAAT GGAGGAAAGG

Jarilla_heterophella_Ycf1 AGAATACCGT CACCCATTTT TACTAAGAAA CTAAAAGGAA CCTCAGAAAC GGAGGAAAGG

Arabidopsis_thaliana_Ycf1 AGAATACCAT CACCCATTTT TACTAAGAAA CTTAAAGGAA CCTCAGAAAC GG--------

....|....| ....|....| ....|....| ....|....| ....|....| ....|....|

785 795 805 815 825 835

Carica_papaya_Ycf1 GGGGGGACTA AACAGGACCA AGAGGTATCC ACCGAAGAAG ATCCTTTTCC TTCTCTTTTT

Vasconcellea_Monoica_Ycf1 GGGGGGACTA AACAGGACCA AGAGGTATCC ACCGAAGAAG ATCCTTTTCC TTCTCTTTTT

Jacaratia_spinosa_Ycf1 GGGGGGACTA AACAGGACCA AGAGGTATCC ACCGAAGAAG ATCCTTTTCC TTCTCTTTTT

Vasconcellea_pubescens_Ycf1 GGGGGGACTA AACAGGACCA AGAGGTATCC ACCGAAGAAG ATCCTTTTCC TTCTCTTTTT

Jarilla_caudata_Ycf1 GGGGGGACTA AACAGGACCA AGAGGTATCC ACCGAAGAAG ATCCTTTTCC TTCTCTTTTT

Jarilla_chocola_Ycf1 GGGGGGACTA AACAGGACCA AGAGGTATCC ACCGAAGAAG ATCCTTTTCC TTCTCTTTTT

Jarilla_heterophella_Ycf1 GGGGGGACTA AACAGGACCA AGAGGTATCC ACCGAAGAAG ATCCTTTTCC TTCTCTTTTT

Arabidopsis_thaliana_Ycf1 -GTGGGACTA AACAGGACCA AGAGGTATCC ACCGAAGAAG CTCCTTTTCC TTCTCTTTTT

....|....| ....|....| ....|....| ....|....| ....|....| ....|....|

845 855 865 875 885 895

Carica_papaya_Ycf1 TCGGAAGAAA GGGAGGATCC GGACAAAATC GATGAAACGG AAGAGATCCG AGTGAATGGA

Vasconcellea_Monoica_Ycf1 TCGGAAGAAA GGGAGGATCC GGACAAAATC GATGAAACGG AAGAGATCCG AGTAAATGGA

Jacaratia_spinosa_Ycf1 TCGGAAGAAA GGGAGGATCC GGACAAAATC GATGAAACGG AAGAGATCCG AGTGAATGGA

Vasconcellea_pubescens_Ycf1 TCGGAAGAAA GGGAGGATCC GGACAAAATC GATGAAACGG AAGAGATCCG AGTAAATGGA

Jarilla_caudata_Ycf1 TCGGAAGAAA GGGAGGATCC GGACAAAATC GATGAAACGG AAGAGATCCG AGTGAATGGA

Jarilla_chocola_Ycf1 TCGGAAGAAA GGGAGGATCC GGACAAAATC GATGAAACGG AAGAGATCCG AGTGAATGGA

Jarilla_heterophella_Ycf1 TCGGAAGAAA GGGAGGATCC GGACAAAATC GATGAAACGG AAGAGATCCG AGTGAATGGA

Arabidopsis_thaliana_Ycf1 TCGGAAGAAG GGGAAGATCT GGACAAAATC GATGAAATGG AAGAAATCCG AGTGAATGGA

....|....| ....|....| ....|....| ....|....| ....|....| ....|....|

905 915 925 935 945 955

Carica_papaya_Ycf1 AAGGAAAAAA AAAATAAGGA TGATGAATTT AAATTTAAAG AGATATCCTC TAACTTTTAT

Vasconcellea_Monoica_Ycf1 AAGGAAAAAA AAAATAAAGA TGATGAATTT CTCTTTACAG AGACATCCTC TAACTTA---

Jacaratia_spinosa_Ycf1 AAGGAAAAAA AAAATAAAGA TGATGAATTT ATCTTTAAAG AGACATCTTC TAACGTT---

Vasconcellea_pubescens_Ycf1 AAGGAAAAAA AAAATAAAGA TGATGAATTT CTCTTTACAG AGACATCCTC TAACTTA---

Jarilla_caudata_Ycf1 AAGGAAAATC AAAATAAGGA TGATGAATTT TTCTTTAAAG AGACATCCTC TAACTTT---

Jarilla_chocola_Ycf1 AAGGAAAATA AAAATAAGGA TGATGAATTT TTATTTAAAG AGACATCCTC TAACTTT---

Jarilla_heterophella_Ycf1 AAGGAAAATC AAAATAAGGA TGATGAATTT TTCTTTAAAG AGACATCCTC TAACTTT---

```
Arabidopsis_thaliana_Ycf1       AAAGACAAAA TTAATAAGGA TGATGAATTC CACGTTCGA- --ACATACTA TAAC------

                                ....|....| ....|....| ....|....| ....|....| ....|....| ....|....|
                                      965        975        985        995       1005       1015
Carica_papaya_Ycf1              AACTTTAAAT ATAAAAATAG GCTAGGTTAT GAAAATCTAG ATGAGAATCA AGAAAATTCC
Vasconcellea_Monoica_Ycf1       ------AAAT ATAAAAATAG GCTAGTTTAT GAAAATCTAG ATGAGAATCA AGAAAATTCC
Jacaratia_spinosa_Ycf1          ------AAAT ATAAAAATAG GCTAGTTTAT GAAAATCTAG ATGAGAATCA AGAAAATTCC
Vasconcellea_pubescens_Ycf1     ------AAAT ATAAAAATAG GCTAGTTTAT GAAAATCTAG ATGAGAATCA AGAAAATTCC
Jarilla_caudata_Ycf1            ------AAAT ATAAAAAAAG GCTAGTTTCT GAAAATCTAG ATGAGAATCA AGAAAATTCC
Jarilla_chocola_Ycf1            ------AAAT ATAAAAAAAG GCTAGTTTCT GAAAATCTAG ATGAGAATCA AGAAAATTCC
Jarilla_heterophella_Ycf1       ------AAAT ATAAAAAAAG GCTAGTTTCT GAAAATCTAG ATGAGAATCA AGAAAATTCC
Arabidopsis_thaliana_Ycf1       ---------T ATAAAACT-- ----GTTTCT GAAAATCTAT ATGGAAATAA AGAAAATTCG

                                ....|....| ....|....| ....|....| ....|....| ....|....| ....|....|
                                     1025       1035       1045       1055       1065       1075
Carica_papaya_Ycf1              AATTTAGAAA TATTAAAAA- --GAAAAGAA GATAAATATT TATTATGGTT TGAAAAACCT
Vasconcellea_Monoica_Ycf1       AATTTCGAAA TATTAAAAA- --GAAAAGAA GATAAATATT TATTATGGTT TGAAAAACCG
Jacaratia_spinosa_Ycf1          AATTTAGAAA TATTAAAAA- --GAAAAGAA GATAAATATT TATTATGGTT TGAAAAACCT
Vasconcellea_pubescens_Ycf1     AATTTCGAAA TATTAAAAA- --GAAAAGAA GATAAATATT TTTTATGGTT TGAAAAACCG
Jarilla_caudata_Ycf1            AATTTAGAAA TATTAAAAA- --ACAAAGAA GATAAATATT TATTATGGTT TGAAAAACCT
Jarilla_chocola_Ycf1            AATTTAGAAA TATTAAAAA- --ACAAAGAA GATAAATATT TATTATGGTT TGAAAAACCT
Jarilla_heterophella_Ycf1       AATTTAGAAA TATTAAAAA- --ACAAAGAA GATAAATATT TATTATGGTT TGAAAAACCT
Arabidopsis_thaliana_Ycf1       AATTTAGAAT TTTTCAAAAT AAAAAAAAAA GAGGATCATT TTTTATGGTT TGAAAAACCA

                                ....|....| ....|....| ....|....| ....|....| ....|....| ....|....|
                                     1085       1095       1105       1115       1125       1135
Carica_papaya_Ycf1              TTTGTGACTC TTCTTTTTGA TTATAAACGG TGGAATCGGC CATTTCGATA TCTAAAAAAT
Vasconcellea_Monoica_Ycf1       TTTGTGACTC TTCTTTTTGA TTATAAACGG TGGAATCGGC CATTTCGATA TATAAAAAAT
Jacaratia_spinosa_Ycf1          TTTGTGACTC TTCTTTTTGA TTATAAACGG TGGAATCGGC CATTTCGATA TATAAAAAAT
Vasconcellea_pubescens_Ycf1     TTTGTGACTC TTCTTTTTGA TTATAAACGG TGGAATCGGC CATTTCGATA TATAAAAAAT
Jarilla_caudata_Ycf1            TTTGTTACTC TTCTTTTTGA TTATAAACGA TGGAATCGGC CATTTCGATA TATAAAAAAT
Jarilla_chocola_Ycf1            TTTGTTACTC TTCTTTTTGA TTATAAACGA TGGAATCGGC CATTTCGATA TATAAAAAAT
Jarilla_heterophella_Ycf1       TTTGTTACTC TTCTTTTTGA TTATAAACGA TGGAATCGGC CATTTCGATA TATAAAAAAT
Arabidopsis_thaliana_Ycf1       TTTGTAACTC TCGTTTTCGA TTATAAAAGA TGGAATCGAC CAAACCGATA TATAAAAAAT

                                ....|....| ....|....| ....|....| ....|....| ....|....| ....|....|
                                     1145       1155       1165       1175       1185       1195
Carica_papaya_Ycf1              GATCGATTTG AAAATGCTGT AAGAAATCAA ATGTCACAAT ATTTTTTTTA TACATGTCAA
Vasconcellea_Monoica_Ycf1       GATCAGTTTG AAAATGCTAT AAGAAATAAA ATGTCACAAT ATTTTTTTTA TACATGTCAA
Jacaratia_spinosa_Ycf1          GATCGGTTTG AAAATGCTAT AAGAAATAAA ATGTCACAAT ATTTTTTTTA TACATGTCAA
Vasconcellea_pubescens_Ycf1     GATCAGTTTG AAAATGCTAT AAGAAATAAA ATGTCACAAT ATTTTTTTTA TACATGTCAA
Jarilla_caudata_Ycf1            GATCAATTTG AAAATGCTGT AAGAAATAAA ATGTCACAAT ATTTTTTTTA TACATGTCAA
Jarilla_chocola_Ycf1            GATCAATTTG AAAATGCTGT AAGAAATAAA ATGTCACAAT ATTTTTTTTA TACATGTCAA
Jarilla_heterophella_Ycf1       GATCAATTTG AAAATGCTGT AAGAAATAAA ATGTCACAAT ATTTTTTTTA TACATGTCAA
```

```
Arabidopsis_thaliana_Ycf1       GATAAAATTG AAAATATTGT AAGAAATGAA ATGTCACAAT ATTTTTTTTA TACATGCCAA

                                ....|....| ....|....| ....|....| ....|....| ....|....| ....|....|
                                    1205       1215       1225       1235       1245       1255
Carica_papaya_Ycf1              AGTGATGGAA AAGAAAGAAT ATCTTTTACG TATCCACCCA GTTTGGCAAC TTTTTTGGAA
Vasconcellea_Monoica_Ycf1       AGTGATGGAA AAGAACAAAT ATCTTTTACG TATCCACCCA GTTTGGCAAC TTTTTTGGAA
Jacaratia_spinosa_Ycf1          AGTGATGGAA AAGAAAGAAT ATCTTTTACG TATCCACCCA GTTTGGCAAC TTTTTTGGAA
Vasconcellea_pubescens_Ycf1     AGTGATGGAA AAGAACGAAT ATCTTTTACG TATCCACCCA GTTTGGCAAC TTTTTTGGAA
Jarilla_caudata_Ycf1            AGTGATGGAA AAGAAAGAAT ATCTTTTACG TATCCACCCA GTTTGGCAAC TTTTTTGGAA
Jarilla_chocola_Ycf1            AGTGATGGAA AAGAAAGAAT ATCTTTTACG TATCCACCCA GTTTGGCGAC TTTTTTGGAA
Jarilla_heterophella_Ycf1       AGTGATGGAA AAGAAAGAAT ATCTTTTACG TATCCACCCA GTTTGGCAAC TTTTTTGGAA
Arabidopsis_thaliana_Ycf1       AGTGATGGAA AAGAACGAAT ATCTTTTACA TATCCCCCCA ACCTTTCCAC CTTTTTTGAA

                                ....|....| ....|....| ....|....| ....|....| ....|....| ....|....|
                                    1265       1275       1285       1295       1305       1315
Carica_papaya_Ycf1              ATGATACAAC AAAAGATGCC TTTGTTCACA CAAGAAAAAT TATCCTCTAA TGAATTCTAT
Vasconcellea_Monoica_Ycf1       CTGATACAAA AAAAGATGCC GTTGTGCACA CAAGACAAAT TATCCTCTAA TGAATTCTAT
Jacaratia_spinosa_Ycf1          ATGATACAAA AAACGATGCC TTTGTTCACA CAAGAAAAAT TATCTTCTAA TGAATTCTAT
Vasconcellea_pubescens_Ycf1     CTGATACAAA AAAAGATGCC GTTGTGCACA CAAGACAAAT TATCCTCTAA TGAATTCTAT
Jarilla_caudata_Ycf1            ATGATACAAA AAAAGATGCC CTTGTTCACC CAAGAAAAAT TATCCTCTAA TGAATTCTAT
Jarilla_chocola_Ycf1            ATGATACAAA AAAAGATGCC CTTGTTCACC CAAGAAAAAT TATCCTCTAA TGAATTCTAT
Jarilla_heterophella_Ycf1       ATGATACAAA AAAAGATGCC CTTGTTCACC CAAGAAAAAT TATCCTCTAA TGAATTCTAT
Arabidopsis_thaliana_Ycf1       ATGATCCAAA AAAGGATACC TTCATTTACA AAAGAAAAAA AAACCTTTGA CCAAGTTTCT

                                ....|....| ....|....| ....|....| ....|....| ....|....| ....|....|
                                    1325       1335       1345       1355       1365       1375
Carica_papaya_Ycf1              AATCATTGGA TTCTTACCAA TGAGAAAAAA GAGAGCAAGT TAAAGAACGA ATTTCTAAAT
Vasconcellea_Monoica_Ycf1       AATCATTGGA TTCTTACCAA TGAGAAAAAA GAGAGCAAGT TAAAGAACGA ATTTATAAAT
Jacaratia_spinosa_Ycf1          AATCATTGGA TTCTTACCAA TGAGAAAAAA GAGAGCAAGT TAAAGAACGA ATTTCTAAAT
Vasconcellea_pubescens_Ycf1     AATCATTGGA TTCTTACCAA TGAGAAAAAA GAGAGCAAGT TAAAGAACGA ATTTATAAAT
Jarilla_caudata_Ycf1            AATCATTGGA TTCTTACCAA TGAGAAAAAA GAGAGTAAGT TAACGAACGA ATTTTTAAAT
Jarilla_chocola_Ycf1            AATCATTGGA TTCTTACCAA TGAGAAAAAA GAGAGTAAGT TAACGAACGA ATTTTTAAAT
Jarilla_heterophella_Ycf1       AATCATTGGA TTCTTACCAA TGAGAAAAAA GAGAGTAAGT TAACGAACGA ATTTTTAAAT
Arabidopsis_thaliana_Ycf1       ACTTATTGGA GTTTGATCCA TGAAGAAAAA AGGGAAAACT TAAAAAAAGA ATTTTTAAAT

                                ....|....| ....|....| ....|....| ....|....| ....|....| ....|....|
                                    1385       1395       1405       1415       1425       1435
Carica_papaya_Ycf1              AGAATTAAAG CTTTAGATAA AGGATTTCTT GCTCTGAATA TACTCGAAAA AAGGACTCGA
Vasconcellea_Monoica_Ycf1       AGAATTGAAA CTTTAGATAA AGGAGTTCTT GCTCTGAATA TATTCGCAAA AAGGACTCGA
Jacaratia_spinosa_Ycf1          AGAATTGAAG CCTTAGATAA AGGATTTCTT GCTCTGAATA TACTCGAAAA AAGGACTCGC
Vasconcellea_pubescens_Ycf1     AGAATTGAAA CTTTAGATAA AGGAGTTCTT GCTCTGAATA TATTCGCAAA AAGGACTCGA
Jarilla_caudata_Ycf1            AGAATTGAAG CTTTAGATAA AGGATTTCTT GCTCTGAATA TACTCGAAAA AAGGACGCGA
Jarilla_chocola_Ycf1            AGAATTGAAG CTTTAGATAA AGGATTTCTT GCTCTGAATA TACTCGAAAA AAGGACGCGA
Jarilla_heterophella_Ycf1       AGAATTGAAG CTTTAGATAA AGGATTTCTT GCTCTGAATA TACTCGAAAA AAGGACGCGA
```

Arabidopsis_thaliana_Ycf1 AGAATTGAAG CTTTAGATAA GGAATGGTCT GTTGAAAATA TACTGGAAAA AACGACTCGC

....|....| ....|....| ....|....| ....|....| ....|....| ....|....|

1445 1455 1465 1475 1485 1495

Carica_papaya_Ycf1 TTTTGTAATA ATGAGACTAA AAAAGAATAT TTGCCTAAAA TTTATGATCC GTTATTATAT

Vasconcellea_Monoica_Ycf1 TTTTGTAATA ATGAGACTAA AAAAGAATAT TTGCCTAAAA TTTATGATCC GTTCTTATAT

Jacaratia_spinosa_Ycf1 TTTTGTAATA ATGAGACTAA AAAAGAATAT TTGCCTAAAA GTTATGATCC GTTCTTATAT

Vasconcellea_pubescens_Ycf1 TTTTGTAATA ATGAGACTAA AAAAGAATAT TTGCCTAAAA TTTATGATCC GTTCTTATAT

Jarilla_caudata_Ycf1 TTTTGTAATA ATGAGACTAA AAAAGAATAT TTGCCTAAAA TTTATGATCC GTTATTATAT

Jarilla_chocola_Ycf1 TTTTGTAATA ATGAGACTAA AAAAGAATAT TTGCCTAAAA TTTATGATCC GTTATTATAT

Jarilla_heterophella_Ycf1 TTTTGTAATA ATGAGACTAA AAAAGAATAT TTGCCTAAAA TTTATGATCC GTTATTATAT

Arabidopsis_thaliana_Ycf1 TTTTGTTATA ACGAAGCTAA AAAAGAATAT TTACCTAAAA TTTATGATCC TTTTTTGCAT

....|....| ....|....| ....|....| ....|....| ....|....| ....|....|

1505 1515 1525 1535 1545 1555

Carica_papaya_Ycf1 GGATCCTATC GTGGAAGAAT CATAAATTTT CTTTCACTAC C--------- TGAAACTTAT

Vasconcellea_Monoica_Ycf1 GGATCCTATC GTGGAAGAAT CAAAAATTTT CTTTCACTAC CAAACGTAAC CGAAACTTAT

Jacaratia_spinosa_Ycf1 GGATCCTATC GTGGAAGAAT CAAAAATTTT CTTTCACTAC CAAACGTAAC TGAAACTTAT

Vasconcellea_pubescens_Ycf1 GGATCCTATC GTGGAAGAAT CAAAAATTTT CTTTCACTAC CAAACGTAAC CGAAACTTAT

Jarilla_caudata_Ycf1 GGATCCTATC GTGGAAGAAT CATAAATTTT CTTTCACTAC CAAACGTAAC TGAAACTTAT

Jarilla_chocola_Ycf1 GGATCCTATC GTGGAAGAAT CATAAATTTT CTTTCACTAC CAAACGTAAC TGAAACTTAT

Jarilla_heterophella_Ycf1 GGATCCTATC GTGGAAGAAT CATAAATTTT CTTTCACTAC CAAACGTAAC TGAAACTTAT

Arabidopsis_thaliana_Ycf1 GGAATCTCTC GGGGAAGAAT CAAAAAATTA CCTCCATTCC AAATCATAAC CGAAACCTAT

....|....| ....|....| ....|....| ....|....| ....|....| ....|....|

1565 1575 1585 1595 1605 1615

Carica_papaya_Ycf1 ATAAAAAATA ACATAGGAAC GGTTTTGATA AATAAGATTC ATAGTATACT TCTTAATACT

Vasconcellea_Monoica_Ycf1 ATAAAAAATA ACATAGGAAC GGTTCTGATA AATAAGATTC ATAGTCTACT TCTTAATACT

Jacaratia_spinosa_Ycf1 ATAAAAAATA ACATAGGAAC GGTTCTGATA AATAAGATTC ATAGTATACT TCTTAATACT

Vasconcellea_pubescens_Ycf1 ATAAAAAATA ACATAGGAAC GGTTCTGATA AATAAGATTC ATAGTCTACT TCTTAATACT

Jarilla_caudata_Ycf1 ATAAAAAAAA ACATAGGAAC GGTTTTGATA AATAAGATTC ATAGTATACT TCTTAATACT

Jarilla_chocola_Ycf1 ATAAAAAAAA ACATAGGAAC GGTTTTGATA AATAAAATTC ATAGGATACT TCTTAATACT

Jarilla_heterophella_Ycf1 ATAAAAAAAA ACATAGGAAC GGTTTTGATA AATAAGATTC ATAGTATACT TCTTAATACT

Arabidopsis_thaliana_Ycf1 AGAAAAAACA ATTTAGGAGG ATCCTGGATA AACAAGATTC ATGGTCTACT TCTGAAGATT

....|....| ....|....| ....|....| ....|....| ....|....| ....|....|

1625 1635 1645 1655 1665 1675

Carica_papaya_Ycf1 GATTATCACG AATTTGAACA GACAATAGAA ATAGATACAC TTAATAGAAA ATCATTATCA

Vasconcellea_Monoica_Ycf1 GATTATCACG AATTTGAACA GACAATAGAA ATAGATACAC TTAATAGAAA ATCATTATCA

Jacaratia_spinosa_Ycf1 GATTATCACG AATTTGAACA GACAATAGAA ATAGATACAC TTAATAGAAA ATCATTATCA

Vasconcellea_pubescens_Ycf1 GATTATCACG AATTTGAACA GACAATAGAA ATAGATACAC TTAATAGAAA ATCATTATCA

Jarilla_caudata_Ycf1 GATTATCACG AATTTGAACA GACAATAGAA ATAGATACAC TTAATAGAAA ATCATTATCA

Jarilla_chocola_Ycf1 GATTATCACG AATTTGAACA GACAATAGAA ATAGATACAC TTAATAGAAA ATCATTATCA

Jarilla_heterophella_Ycf1 GATTATCACG AATTTGAACA GACAATAGAA ATAGATACAC TTAATAGAAA ATCATTATCA

```
Arabidopsis_thaliana_Ycf1         AATTATAAAA AATTTGAGCA AACAATAGAA AAA------T TTAATAGAAA ATCTTTGTCA

                                   ....|....| ....|....| ....|....| ....|....| ....|....| ....|....|
                                      1685       1695       1705       1715       1725       1735
Carica_papaya_Ycf1                ATAGAAAAAA GACTTTCTTT ATTTCCA--- ---GAACACC AACAAGAGCA AGTTGATTCA
Vasconcellea_Monoica_Ycf1         ATAGAAAAAA GACTTTCTTT ATTTCCATTT CCAGAACACC AAAAAGAGCA AGTTGATTCA
Jacaratia_spinosa_Ycf1            ATAGAAAAAA GACTTTATTT ATTTCCA--- ---GAACACC AAAAAGAGAA AGTTGATTCA
Vasconcellea_pubescens_Ycf1       ATAGAAAAAA GACTTTCTTT ATTTCCATTT CCAGAACACC AAAAAGAGCA AGTTGATTCA
Jarilla_caudata_Ycf1              ATAGAAAAAA GACTTTCTTT ATTTCCA--- ---GAACACC AACAAGAGCA AGTTGATTCA
Jarilla_chocola_Ycf1              ATAGAAAAAA GACTTTCTTT ATTTCCA--- ---GAACACC AACAAGAGCA AGTTGATTCA
Jarilla_heterophella_Ycf1         ATAGAAAAAA GACTTTCTTT ATTTCCA--- ---GAACACC AACAAGAGCA AGTTGATTCA
Arabidopsis_thaliana_Ycf1         ATAGAGAAAA AACTTTCTTT TTTTTCA--- GAACCCCAAC AAGAAGAAAA AATTAATTCA

                                   ....|....| ....|....| ....|....| ....|....| ....|....| ....|....|
                                      1745       1755       1765       1775       1785       1795
Carica_papaya_Ycf1                AAAAATCGAA AAAATAAAAT TCAATTTTTA TTCTTTTTAT TCGATGTGGT TCTAACTAAT
Vasconcellea_Monoica_Ycf1         AAAAATCGAA AAAAAAAAAT TCAATTTTTA TTC------- --GATGCGGT TCTAACTGAT
Jacaratia_spinosa_Ycf1            AAAAATCGAA AAAAAAAAAT TCCATTTTTA TTT------- --GATACGGT TCTAACTGAT
Vasconcellea_pubescens_Ycf1       AAAAATCGAA AAAAAAAAAT TCAATTTTTA TTC------- --GATGCGGC TCTAACTGAT
Jarilla_caudata_Ycf1              AAAAATCGAA AAAAAAAAAT TCAATTTTTA TTT------- --GATGCGGT TCTAACTGAT
Jarilla_chocola_Ycf1              AAAAATCGAA AAAAAAAAAT TCAATTTTTA TTT------- --GATGCGGT TCTAACTGAT
Jarilla_heterophella_Ycf1         AAAAATCGAA AAAAAAAAAT TCAATTTTTA TTT------- --GATGCGGT TCTAACTGAT
Arabidopsis_thaliana_Ycf1         GAAGAAGAAA TCAAAACTTT CAAATTTTTA TTT------- --GATATTGT TAGAACTGAT

                                   ....|....| ....|....| ....|....| ....|....| ....|....| ....|....|
                                      1805       1815       1825       1835       1845       1855
Carica_papaya_Ycf1                CCCAATGATC AAACG----- ----AATTCT GTTGGAATAA AAGAAATCAG TAAAAAAGTT
Vasconcellea_Monoica_Ycf1         CCCAATGATC AAACG----- ----AATTCT GTTGGAATAA AAGAAATCAG TAAAAAAGTT
Jacaratia_spinosa_Ycf1            CCCAATGATC TAACA----- ----AATTCT GTTGGAATAA AAGAAATCAG TAAAAAAGTT
Vasconcellea_pubescens_Ycf1       CCCAATGATC AAACG----- ----AATTCT GTTGGAATAA AAGAAATCAG TAAAAAAGTT
Jarilla_caudata_Ycf1              CCCAATGATC AAACG----- ----AATTCT GTTGGAATAA AAGAAATCAG TAAAAAAGTT
Jarilla_chocola_Ycf1              CCCAATGATC AAACG----- ----AATTCT GTTGGAATAA AAGAAATCAG TAAAAAAGTT
Jarilla_heterophella_Ycf1         CCCAATGATC AAACG----- ----AATTCT GTTGGAATAA AAGAAATCAG TAAAAAAGTT
Arabidopsis_thaliana_Ycf1         AGCAACGATC AAACGCTTAT AAAAAATTTT ATGGATTTCC CTGAAATCAA TAAAAAAGTT

                                   ....|....| ....|....| ....|....| ....|....| ....|....| ....|....|
                                      1865       1875       1885       1895       1905       1915
Carica_papaya_Ycf1                CCTCGTTGGT CATACAAATT AATCGACGAT TTAGAACAAC TGGAGGGGGA AAATGAAGAA
Vasconcellea_Monoica_Ycf1         CCTTGTTGGT CATACAAATT GATCGACGAT TTAGAACAAC AGGAGGGGGA AAATGAAGAA
Jacaratia_spinosa_Ycf1            CCTCGTTGGT CATACAAATT AATCGACGAT TTAGAACAAC AGGAGGGGGA AAATGAAGAA
Vasconcellea_pubescens_Ycf1       CCTCGTTGGT CATACAAATT GATCGACGAT TTAGAACAAC AGGAGGGGGA AAATGAAGAA
Jarilla_caudata_Ycf1              CCTCGTTGGT CATACAAATT AATCGACGAT TTAGAACTAC TGGAGGGGGA AAATGAAGAA
Jarilla_chocola_Ycf1              CCTCGTTGGT CATACAAATT AATCGACGAT TTAGAACTAC TGGAGGGGGA AAATGAAGAA
Jarilla_heterophella_Ycf1         CCTCGTTGGT CATACAAATT AATCGACGAT TTAGAACTAC TGGAGGGGGA AAATGAAGAA
```

```
Arabidopsis_thaliana_Ycf1        CCTCGATGGT CATACAAATT AATAAGTGAG TTGGAAGAAT TGGAAGGCGA AAATGAAGAA

                                 ....|....| ....|....| ....|....| ....|....| ....|....| ....|....|
                                     1925       1935       1945       1955       1965       1975
Carica_papaya_Ycf1               AACGTGACAG CGGATCCTGA AATTCGTTCA AGAAAAGCCA AACATGTAGT GATTTTTACT
Vasconcellea_Monoica_Ycf1        AGCGTGACCG CCGATCCTGA AATTCGTTCA AGAAAAGCCA AACATGTAGT GATTTTTACT
Jacaratia_spinosa_Ycf1           AGCGTGACAG CCGATCCTGA AATTCGTTCA AGAAAAGCCA AACGTGTAGT GATTTTTACT
Vasconcellea_pubescens_Ycf1      AGCGTGACCG CCGATCCTGA AATTCGTTCA AGAAAAGCCA AACATGTAGT GATTTTTACT
Jarilla_caudata_Ycf1             AACATGACAG GGGATCATGA AATTCGTTCA AGAAAAGCCA AACGTGTAGT GATTTTTACT
Jarilla_chocola_Ycf1             AACATGACAG GGGATCATGA AATTCGTTCA AGAAAAGCCA AACGTGTAGT GATTTTTACT
Jarilla_heterophella_Ycf1        AACATGACAG GGGATCATGA AATTCGTTCA AGAAAAGCCA AACGTGTAGT GATTTTTACT
Arabidopsis_thaliana_Ycf1        AATGTACCAA TGGAGCCTGG AATTCGTTCA AGAAAAGCAA AACGTGTAGT GGTTTTTACT

                                 ....|....| ....|....| ....|....| ....|....| ....|....| ....|....|
                                     1985       1995       2005       2015       2025       2035
Carica_papaya_Ycf1               GATAACCAAC AGAATAACGA TATTTATACT AACATCAAAG ATACTAATAA TTCTGATGAA
Vasconcellea_Monoica_Ycf1        GATAACCAAC AGAATAACGA TATTTATACT AATATAAAAG ATATTAATAA TTCTGATCAA
Jacaratia_spinosa_Ycf1           GATAACCAAC AGAATAACGA TATTTATACT AATATCAAAG ATACTAATAA TTCTGATCAA
Vasconcellea_pubescens_Ycf1      GATAACCAAC AGAATAACGA TATTTATACT AATATAAAAG ATATTAATAA TTCTGATCAA
Jarilla_caudata_Ycf1             GAGAACCAAG AGAATAACGA TATTTATAAT AGTCTCCAAG ATTTTACTAA TTCTGATCAA
Jarilla_chocola_Ycf1             GAGAACCAAG AGAATAACGA TATTTATAAT AGTCTCCAAG ATTCTACTAA TTCGGATCAA
Jarilla_heterophella_Ycf1        GAGAACCAAG AGAATAACGA TATTTATAAT AGTCTCCAAG ATTTTACTAA TTCTGATCAA
Arabidopsis_thaliana_Ycf1        GATAAAGAGC CACATGGCGA GATTTATACT AATCTCAAAG ATAATCAAAA TTCTGATCAA

                                 ....|....| ....|....| ....|....| ....|....| ....|....| ....|....|
                                     2045       2055       2065       2075       2085       2095
Carica_papaya_Ycf1               AGAGACGAAG TAGCTTTGAT ACGTTATTCA CAACAATCAG ATTTTCGTCG AGACATAATC
Vasconcellea_Monoica_Ycf1        AGAGACGAAG TGGCTTTGAT ACGTTATTCA CAACAATCAG ATTTTCGTCG AGACATAATC
Jacaratia_spinosa_Ycf1           AGAGACGAAG TGGCTTTGAT ACGTTATTCA CAACAATCAG ATTTTCGTCG AGACATAATC
Vasconcellea_pubescens_Ycf1      AGAGACGAAG TGGCTTTGAT ACGTTATTCA CAACAATCAG ATTTTCGTCG AGACATAATC
Jarilla_caudata_Ycf1             AGAGACGAAG TGGCTTTGAT ACGTTATTCA CAACAATCAG ATTTTCGTCG AGACATAATC
Jarilla_chocola_Ycf1             AGAGACGAAG TGGCTTTGAT ACGTTATTCA CAACAATCAG ATTTTCGTCG AGACATAATC
Jarilla_heterophella_Ycf1        AGAGACGAAG TGGCTTTGAT ACGTTATTCA CAACAATCAG ATTTTCGTCG AGACATAATC
Arabidopsis_thaliana_Ycf1        AACGATGAAA TGGCTTTGAT CCGTTATTCG CAACAATCTG ATTTTCGTCG AGAGATAATT

                                 ....|....| ....|....| ....|....| ....|....| ....|....| ....|....|
                                     2105       2115       2125       2135       2145       2155
Carica_papaya_Ycf1               AAAGGATCCA TGCGTCCTCA AAGACGTAAA ACAGTTACTT GGGAACTGTT TCAAGCAAAT
Vasconcellea_Monoica_Ycf1        AAAGGCTCCA TGCGTGCTCA AAGACGTAAA ACAGTTCCTT GGGAACTGTT TCAAGCAAAT
Jacaratia_spinosa_Ycf1           AAAGGCTCCA TGCGTGCTCA AAGACGTAAA ACAGTTACTT GGGAACTGTT TCAAGCAAAT
Vasconcellea_pubescens_Ycf1      AAAGGCTCCA TGCGTGCTCA AAGACGTAAA ACAGTTCCTT GGGAACTGTT TCAAGCAAAT
Jarilla_caudata_Ycf1             AAAGGCTCCA TGCGTGCTCA AAGACGTAAA ACAGTTCCTT GGGAACTATT TCAATCAAAT
Jarilla_chocola_Ycf1             AAAGGCTCCA TGCGTGCTCA AAGACGTAAA ACAGTTCCTT GGGAACTATT TCAATCAAAT
Jarilla_heterophella_Ycf1        AAAGGCTCCA TGCGTGCTCA AAGACGTAAA ACAGTTCCTT GGGAACTATT TCAATCAAAT
```

Arabidopsis_thaliana_Ycf1 AAAGGATCCA TGCGTTCCCA AAGGCGTAAA ACTGTTATTT GGGAATTTTT TCAAGCAAAA

....|....| ....|....| ....|....| ....|....| ....|....| ....|....|

2165 2175 2185 2195 2205 2215

Carica_papaya_Ycf1 GCGCATTCCC CTCTTTTTTT GGACAGAATA GACAAACCCC TTTTTTTTTT TTCTGCATTT

Vasconcellea_Monoica_Ycf1 GTGCATTCGC CTCTTTTTTT GGACAGAATA GACAAACCCC TTTTTTCTT- -----CATTT

Jacaratia_spinosa_Ycf1 GTGCATTCCC CTCTTTTTTT GGACAGAATA GACAAACCCC TTTTTTCTT- -----CATTT

Vasconcellea_pubescens_Ycf1 GTGCATTCGC CTCTTTTTTT GGACAGAATA GACAAACCCC TTTTTTCTT- -----CATTT

Jarilla_caudata_Ycf1 GTGCATTCCC CTCTTTTTTT GGACAGAATA GACAAACCTC TTTTTTCTT- -----TATTT

Jarilla_chocola_Ycf1 GTGCATTCCC CTCTTTTTTT GGACAGAATA GACAAACCTC TTTTTTCTT- -----TATTT

Jarilla_heterophella_Ycf1 GTGCATTCCC CTCTTTTTTT GGACAGAATA GACAAACCTC TTTTTTCTT- -----TATTT

Arabidopsis_thaliana_Ycf1 GTACATTCCC CCCTTTTTTT TGATAGAATA GATAAACTTT TTTTTTTTT- -----CGTTT

....|....| ....|....| ....|....| ....|....| ....|....| ....|....|

2225 2235 2245 2255 2265 2275

Carica_papaya_Ycf1 GATATTTCTG AACTGATGAA ACGCATTTTT AGAAATAGGA TATGGATACG GACAAACCAG

Vasconcellea_Monoica_Ycf1 GATATTTCTG AACTGATGAA ACTCATTTTT AGAAATAGGA TATGGG---- --CAAACAAG

Jacaratia_spinosa_Ycf1 GATATTTCTG AACTGATGAA ACTCATTTTT AGAAATAGGA TATGGG---- --CAAACAAG

Vasconcellea_pubescens_Ycf1 GATATTTCTG AACTGATGAA ACTCATTTTT AGAAATAGGA TATGGG---- --CAAACAAG

Jarilla_caudata_Ycf1 GATATTTCTG AACTGATGAA ACTTATTTTT ACAAATAGGA TATGGA---- --CAAACAAG

Jarilla_chocola_Ycf1 GATATTTCTG AACTGATGAA ACTTATTTTT ACAAATAGGA TATGGA---- --CAAACAAG

Jarilla_heterophella_Ycf1 GATATTTCTG AACTGATGAA ACTTATTTTT ACAAATAGGA TATGGA---- --CAAACAAG

Arabidopsis_thaliana_Ycf1 GATATATGGG GGCTAAAAAA AAAAATTATT AAAAATTTCA TTTGGA---- ----------

....|....| ....|....| ....|....| ....|....| ....|....| ....|....|

2285 2295 2305 2315 2325 2335

Carica_papaya_Ycf1 GAAAAAAAAA ATTCTGATTA TACAGAAGAA AAGACAAAAA CAATTGAGAA AAAAGAAGAC

Vasconcellea_Monoica_Ycf1 GAAAAAAAAA TTTCTGATTA TACGGAAGAA AAGACAAAAA CAATTGCGAA AACAAAAGAA

Jacaratia_spinosa_Ycf1 GAAAAAAAAA TTTCTGATTA TACAGAAGAA AAGACAAAAA CAATTGAGAA AAAAAAAGAA

Vasconcellea_pubescens_Ycf1 GAAAAAAAAA TTTCTGATTA TACGGAAGAA AAGACAAAAA CAATTGCGAA AACAAAAGAA

Jarilla_caudata_Ycf1 GAAAAAAAAA ATTCTGATTA TACAGAAGAA AAGACAAAAA CAATTGAGAA AAAAGAAGAA

Jarilla_chocola_Ycf1 GAAAAAAAAA ATTCTGATTA TACAGAAGAA AAGACAAAAA CAATTGAGAA AAAAGAAGAA

Jarilla_heterophella_Ycf1 GAAAAAAAAA ATTCTGATTA TACAGAAGAA AAGACAAAAA CAATTGAGAA AAAAGAAGAA

Arabidopsis_thaliana_Ycf1 -AAAAAAAAA ---------- ---------- ---------- ---TTGATAA AAAAGAAGA-

....|....| ....|....| ....|....| ....|....| ....|....| ....|....|

2345 2355 2365 2375 2385 2395

Carica_papaya_Ycf1 GACAAAAGAG AACAATACAA AAGAGAAGAA AAAACACGAA TAGAAATAGC GGAAGCCTGG

Vasconcellea_Monoica_Ycf1 GACAAAAGAG AAAAATACAA AAGAGAAGAA AAAACACGGA TAGAAATAGC GGAAGCCTGG

Jacaratia_spinosa_Ycf1 GACAAAAGAG AAAAATACAA AAGAGAAGAA AAAACACGGA TAGAAATAGC GGAAGCCTGG

Vasconcellea_pubescens_Ycf1 GACAAAAGAG AAAAATACAA AAGAGAAGAA AAAACACGGA TAGAAATAGC GGAAGCCTGG

Jarilla_caudata_Ycf1 GATAAAAGAA AAAAATACAA AAGAGAGGAA AAAACACGGA TAGAAATAGC AGAAACCTGG

Jarilla_chocola_Ycf1 GATAAAAGAA AAAAATACAA AAGAGAGGAA AAAACACGGA TAGAAATAGC AGAAACCTGG

Jarilla_heterophella_Ycf1 GATAAAAGAA AAAAATACAA AAGAGAGGAA AAAACACGGA TAGAAATAGC AGAAACCTGG

Arabidopsis_thaliana_Ycf1 --------AG AACAATCAAA AAGAGAAGAA ACAAGACGGA TAGAAATTGC AGAAACTTGG

....|....| ....|....| ....|....| ....|....| ....|....| ....|....|

2405 2415 2425 2435 2445 2455

Carica_papaya_Ycf1 GATAGTCTTT TATTTGCTCA AGTTCTAAGA GGTTTCGTCT TAGTAACCCA ATCGATTCTT

Vasconcellea_Monoica_Ycf1 GATAGTCTTT TATTTGCTCA AGTTATAAGG GGTTTGGTCT TAGTAACCCA CTCGATTATT

Jacaratia_spinosa_Ycf1 GATAGTCTTT TATTTGTTCA AGTTATAAGG GGTTTGGTCT TAGTAACCCA ATCGATTCTT

Vasconcellea_pubescens_Ycf1 GATAGTCTTT TATTTGCTCA AGTTATAAGG GGTTTGGTCT TAGTAACCCA CTCGATTATT

Jarilla_caudata_Ycf1 GATAGTCTTT TATTTGCTCA AGTTCTAAGA GGTTTCGTCT TAGTAACCCA CTCGATTCTT

Jarilla_chocola_Ycf1 GATAGTCTTT TATTTGCTCA AGTTCTAAGA GGTTTCGTCT TAGTAACCCA CTCGATTCTT

Jarilla_heterophella_Ycf1 GATAGTCTTT TATTTGCTCA AGTTCTAAGA GGTTTCGTCT TAGTAACCCA CTCGATTCTT

Arabidopsis_thaliana_Ycf1 GATAGCTTCC TATTTGCTCA AATAATAAGA GGTTCTCTCT TAGTAACTCA ATCAATTCTT

....|....| ....|....| ....|....| ....|....| ....|....| ....|....|

2465 2475 2485 2495 2505 2515

Carica_papaya_Ycf1 AGAAAATATA TTATATTACC TTCCTTGATA ATAGTTAAAA ATATCGCCCG TCTGCTATTA

Vasconcellea_Monoica_Ycf1 AGAAAATATA TTATATTACC TTCCTTGATA ATAGTTAAAA ATATAGCCCG TATGCTATTA

Jacaratia_spinosa_Ycf1 AGAAAATATA TTATATTACC TTCCTTGATA ATAGTTAAAA ATATCGCCCG TATGCTATTA

Vasconcellea_pubescens_Ycf1 AGAAAATATA TTATATTACC TTCCTTGATA ATAGTTAAAA ATATAGCCCG TATGCTATTA

Jarilla_caudata_Ycf1 AGAAAATATA TTATATTACC GTCCTTGATA ATAGTTAAAA ATATCGCCCG TATCCTATTA

Jarilla_chocola_Ycf1 AGAAAATATA TTATATTACC GTCCTTGATA ATAGTTAAAA ATATCGCCCG TATCCTATTG

Jarilla_heterophella_Ycf1 AGAAAATATA TTATATTACC GTCCTTGATA ATAGTTAAAA ATATCGCCCG TATCCTATTA

Arabidopsis_thaliana_Ycf1 AGAAAATATA TTATATTACC TTTATTGATA ATAATTAAAA ACAGTGTCCG TATGTTATTA

....|....| ....|....| ....|....| ....|....| ....|....| ....|....|

2525 2535 2545 2555 2565 2575

Carica_papaya_Ycf1 TTTCAATTTT CCGAATGGTC TGAGGATTTA AAGGATTGGA ATCGAGAAAT GCATATTAAA

Vasconcellea_Monoica_Ycf1 TTTCAATTTC CCGAATGGTC TGAGGATTTA AAGGATTGGA ATCGAGAAAT GCATATTAAA

Jacaratia_spinosa_Ycf1 TTTCAATTTC CCGAATGGTC TGAGGATTTA AAGGATTGGA ATCGAGAAAT GCATATTAAA

Vasconcellea_pubescens_Ycf1 TTTCAATTTC CCGAATGGTC TGAGGATTTA AAGGATTGGA ATCGAGAAAT GCATATTAAA

Jarilla_caudata_Ycf1 TTTCAAATTC CCGAATGGTC TGAGGATTTA AAGGATTGGA ATCGAGAAAT GCATATTAAA

Jarilla_chocola_Ycf1 TTTCAAATTC CCGAATGGTC TGAGGATTTA AAGGATTGGA ATCGAGAAAT GCATATTAAA

Jarilla_heterophella_Ycf1 TTTCAAATTC CCGAATGGTC TGAGGATTTA AAGGATTGGA ATCGAGAAAT GCATATTAAA

Arabidopsis_thaliana_Ycf1 TTTCAATTTC CCGAGTGGTC CCAGGATTTA AAGGATTGGA AACGTGAAAT GCATGTTAAA

....|....| ....|....| ....|....| ....|....| ....|....| ....|....|

2585 2595 2605 2615 2625 2635

Carica_papaya_Ycf1 TGTACCTATA ATGGTGTTCA ATTATCAGAA ACAGAATTTC CAAAAAACTG GTTAACAGAC

Vasconcellea_Monoica_Ycf1 TGCACCTATA ATGGAGTTCA ATTATCAGAA ACAGAATTTC CAAAAAACTG GTTAACAGAC

Jacaratia_spinosa_Ycf1 TGCACCTATA ATGGTGTTCA ATTATCCGAA ACAGAATTTC CAAAAAACTG GTTAACAGAC

Vasconcellea_pubescens_Ycf1 TGCACCTATA ATGGAGTTCA ATTATCAGAA ACAGAATTTC CAAAAAACTG GTTAACAGAC

Jarilla_caudata_Ycf1 TGCACCTATA ATGGTGTTCA ATTATCAGAA ACAGAATTTC CAAAAAACTG GTTAACAGAC

Jarilla_chocola_Ycf1 TGCACCTATA ATGGTGTTCA ATTATCAGAA ACAGAATTTC CAAAAAACTG GTTAACGGAC

Jarilla_heterophella_Ycf1 TGCACCTATA ATGGTGTTCA ATTATCAGAA ACAGAATTTC CAAAAAACTG GTTAACAGAC

Arabidopsis_thaliana_Ycf1 TGTACTTATA ATGGGGTTCA ACTATCCGAA ACAGAATTTC CAAGAAACTG GTTAACGGAT

....|....| ....|....| ....|....| ....|....| ....|....| ....|....|

2645 2655 2665 2675 2685 2695

Carica_papaya_Ycf1 GGTATTCAGA TAAAAATACT ATTCCCTTTT TGCCTTAAAC CTTGGCACAG ATCGAAATTA

Vasconcellea_Monoica_Ycf1 GGTATTCAGA TAAAAATACT ATTCCCTTTT TGCCTTAAAC CTTGGCACAG ATCTAAATTA

Jacaratia_spinosa_Ycf1 GGTATTCAGA TAAAAATACT ATTCCCTTTT TGCCTTAAAC CTTGGCACAG ATCTAAATTA

Vasconcellea_pubescens_Ycf1 GGTATTCAGA TAAAAATACT ATTCCCTTTT TGCCTTAAAC CTTGGCACAG ATCTAAATTA

Jarilla_caudata_Ycf1 GGTATTCAGA TGAAAATACT ATTCCCTTTT TGCCTTAAAC CTTGGCACAG ATCGAAATTA

Jarilla_chocola_Ycf1 GGTATTCAGA TAAAAATACT ATTCCCTTTT TGCCTTAAAC CTTGGCACAG ATCGAAATTA

Jarilla_heterophella_Ycf1 GGTATTCAGA TAAAAATACT ATTCCCTTTT TGCCTTAAAC CTTGGCACAG ATCGAAATTA

Arabidopsis_thaliana_Ycf1 GGTATTCAGA TAAAAATCCT ATTTCCTTTT TATCTTAAAC CTTGGCATAA ATCTAAATTT

....|....| ....|....| ....|....| ....|....| ....|....| ....|....|

2705 2715 2725 2735 2745 2755

Carica_papaya_Ycf1 CGATCCCCTC ATAAAGATCC AATCAAAAAG AGGAAGG--- ---GGGAAAA AAATGATTTT

Vasconcellea_Monoica_Ycf1 CGATCCCCTC ATAAAGATCC AATCAAAAAA AGGAAGG--- ---GAGAAAA AAATGATTTT

Jacaratia_spinosa_Ycf1 CGATCCCCTC ATAAAGATCC AATCAAAAAG AGGAAGG--- ---GGGAAAA AAATGATTTT

Vasconcellea_pubescens_Ycf1 CGATCCCCTC ATAAAGATCC AATCAAAAAA AGGAAGG--- ---GAGAAAA AAATGATTTT

Jarilla_caudata_Ycf1 CGATCCCCTC ATAAAGATCC AATCAAAAAG GGGAGGG--- ---GGGAAAA AAATGATTTT

Jarilla_chocola_Ycf1 CGATCCCCTC ATAAAGATCC AATCAAAAAG GGGAGGG--- ---GGGAAAA AAATGATTTT

Jarilla_heterophella_Ycf1 CGATCCCCTC ATAAAGATCC AATCAAAAAG GGGAGGG--- ---GGGAAAA AAATGATTTT

Arabidopsis_thaliana_Ycf1 CAAGCATCTC AGAAGGCTCG ACTAAAAAAA ACAAAAGACA AAGGAGAAAA AAATGATTTT

....|....| ....|....| ....|....| ....|....| ....|....| ....|....|

2765 2775 2785 2795 2805 2815

Carica_papaya_Ycf1 TGTTTTTTAA CAGTTTGGGG GATGGAAACT GAATTGCCTT TTGGTTCTCC CCGAAAACGG

Vasconcellea_Monoica_Ycf1 TGTTTTTTAA CAGTTTGGGG GATGGAAACT GAATTTCCTT TTGGTTCTCC CCGAAAACGG

Jacaratia_spinosa_Ycf1 TGTTTTTTAA CAGTTTGGGG GATGGAAACT GAATTTCCTT TTGGTTCGCC CCGAAAACGG

Vasconcellea_pubescens_Ycf1 TGTTTTTTAA CAGTTTGGGG GATGGAAACT GAATTTCCTT TTGGTTCTCC CCGAAAACGG

Jarilla_caudata_Ycf1 TGTTTTTTAA CAGTTTGGGG GATGGAAACT GAATTGCCTT TTGGTTCTCC CCGAAAACGG

Jarilla_chocola_Ycf1 TGTTTTTTAA CAGTTTGGGG GATGGAAACT GAATTGCCTT TTGGTTCTCC CCGAAAACGG

Jarilla_heterophella_Ycf1 TGTTTTTTAA CAGTTTGGGG GATGGAAACT GAATTGCCTT TTGGTTCTCC CCGAAAACGG

Arabidopsis_thaliana_Ycf1 TGTTTCTTAA CAGTTTGGGG AATGGAAACC GAACTACCGT TTGGTTCTGC CCAAAGAAAG

....|....| ....|....| ....|....| ....|....| ....|....| ....|....|

2825 2835 2845 2855 2865 2875

Carica_papaya_Ycf1 CCTTCATTTT TTGAACCTCT TTTTAAAGAA TTGAAAAAAA AAATTAGAAA ATTGAAAATT

Vasconcellea_Monoica_Ycf1 CCTTCATTTT TTGAACCTAT TTTTAAAGAA TTGAAAAAAA AAATTATAAA ATTGAAAATT

Jacaratia_spinosa_Ycf1 CCTTCATTTT TTGAACCTAT TTTTAAAGAA TTGAAAAAAA AAATTAGAAA ATTGAAAATT

Vasconcellea_pubescens_Ycf1 CCTTCATTTT TTGAACCTAT TTTTAAAGAA TTGAAAAAAA AAATTATAAA ATTGAAAATT

Jarilla_caudata_Ycf1 CCGTCATTTT TTGAACCTAT TTTTAAAGAA TTGAAAAAAA AAATTAGAAA ATTGAAAATT

Jarilla_chocola_Ycf1 CCGTCATTTT TTGAACCTAT TTTTAAAGAA TTGAAAAAAA AAATTAGAAA ATTGAAAATT

Jarilla_heterophella_Ycf1 CCGTCATTTT TTGAACCTAT TTTTAAAGAA TTGAAAAAAA AAATTAGAAA ATTGAAAATT

Arabidopsis_thaliana_Ycf1 CCTTCTTTTT TTGAACCTAT TTCTAAAGAA TTAAAAAAAA GAATCAAAAA ATTAAAAAAA

....|....| ....|....| ....|....| ....|....| ....|....| ....|....|

2885 2895 2905 2915 2925 2935

Carica_papaya_Ycf1 AAGTATTTTC TGGGTTTAAG GGTTTTAAAA GAAAGAGCAA AAATATTTGT AAAAGTCTCA

Vasconcellea_Monoica_Ycf1 AAGTATTTTC TGGGTTTACG GGTTTTAAAA GAAAGAGCAA AAATTTTTCT AAAAGTCTCA

Jacaratia_spinosa_Ycf1 AAGTATTTTC TGGGTTTAAG GGTTTTAAAA GAAAGAGCAA AAATTTTTCT AAAAGTCTCA

Vasconcellea_pubescens_Ycf1 AAGTATTTTC TGGGTTTACG GGTTTTAAAA GAAAGAGCAA AAATTTTTCT AAAAGTCTCA

Jarilla_caudata_Ycf1 AAGGATTTTC TGGGTTTAAG GATTTTAAAA GAAAGAGCAA AAATTTTTCT AAAAGTCTCA

Jarilla_chocola_Ycf1 AAGGATTTTC TGGGTTTAAG GGTTTTAAAA GAAAGAGCAA AAATTTTTCT AAAAGTCTCA

Jarilla_heterophella_Ycf1 AAGGATTTTC TGGGTTTAAG GGTTTTAAAA GAAAGAGCAA AAATTTTTCT AAAAGTCTCA

Arabidopsis_thaliana_Ycf1 AAGTCTTTTG TGGTTTTAAA GATTTTCAAA GAAAGAGCAC CCATTTTCCT AAAAGTCGCA

....|....| ....|....| ....|....| ....|....| ....|....| ....|....|

2945 2955 2965 2975 2985 2995

Carica_papaya_Ycf1 AAAGAAACAA AAAAACAGGT CAGTACAAAC CTTCTATTTG TAAAAGGAAT AATAAAAGAA

Vasconcellea_Monoica_Ycf1 AAAGAAACAA AAAAACAAGT GATTACAAAC CTTCTATTTG TAAAAGGAAT ACTAAAAGAA

Jacaratia_spinosa_Ycf1 AAAGAAACAA AAAAACAGGT CATTACAAAC CTTCTATTTG TCAAAGGAAT AATAAAAGAA

Vasconcellea_pubescens_Ycf1 AAAGAAACAA AAAAACAAGT CATTACAAAC CTTCTATTTG TAAAAGGAAT ACTAAAAGAA

Jarilla_caudata_Ycf1 AAAGAACCAA AAAAACAGGT CAGTACAAAC CTTCTATTTG TAAAAGAAAT AATCAAAGAA

Jarilla_chocola_Ycf1 AAAGAACCAA AAAAACAGGT CAGTACAAAC CCTCTATTTG TAAAAGAAAT AATCAAAGAA

Jarilla_heterophella_Ycf1 AAAGAACCAA AAAAACAGGT CAGTACAAAC CTTCTATTTG TAAAAGAAAT AATCAAAGAA

Arabidopsis_thaliana_Ycf1 AAAGAAACTA AAAACTGGAT TCTCAAAAAC TTTATTTTTA TAAAAGGAAT ----------

....|....| ....|....| ....|....| ....|....| ....|....| ....|....|

3005 3015 3025 3035 3045 3055

Carica_papaya_Ycf1 TTTTCAAAAC GAAACCGAAG TCCATTATTT GGATTGAAAG AAATATCTGA ATTGAGTGAA

Vasconcellea_Monoica_Ycf1 TTTTCAAAAA TAAACCTAAT TCCATTATTT GGATTGAGAG AAATATCTGA ATTGAGTGAA

Jacaratia_spinosa_Ycf1 TTTTCAAAAA TCAACCCAAT TCCATTATTT GTATTGAGAG AAATATCTGA ATTGAGTGAA

Vasconcellea_pubescens_Ycf1 TTTTCAAAAA TAAACCTAAT TCCATTATTT GGATTGAGAG AAATATCTGA ATTGAGTGAA

Jarilla_caudata_Ycf1 TTTTCAAAAA TAAACCGAAT TCCATTATTT GGATTGAGAG AAATATCTGA ATTGAGTGAA

Jarilla_chocola_Ycf1 TTTTCAAAAA TAAACCGAAT TCCATTATTT GGATTGAGAG AAATATCTGA ATTGAGTGAA

Jarilla_heterophella_Ycf1 TTTTCAAAAA TAAACCGAAT TCCATTATTT GGATTGAGAG AAATATCTGA ATTGAGTGAA

Arabidopsis_thaliana_Ycf1 --TTCAAAAC GAAATCTAAT TCCATTATTT GGCCCGAGAG AAATATATGA ATTAAATGAA

....|....| ....|....| ....|....| ....|....| ....|....| ....|....|

3065 3075 3085 3095 3105 3115

Carica_papaya_Ycf1 ACGAAAAAAG ATTCAAGAAT TAGTAATCAG ATGATTCCCG AATCGTCTGT TCAAATTCGA

Vasconcellea_Monoica_Ycf1 ACGAAAAAAG ATTCACGAAT GAGTAATCAG ATGATTCATG AATTGTCTGT TCAAATTCGA

Jacaratia_spinosa_Ycf1 ATGAAAAAAG ATTCAAGAAT TAGTAATCAG ATGATTCATG AATCGTCTGT TCAAATTCGA

Vasconcellea_pubescens_Ycf1 ACGAAAAAAG ATTCACGAAT GAGTAATCAG ATGATTCATG AATCGTCTGT TCAAATTCGA

Jarilla_caudata_Ycf1 ACGAAAAAAG ATTCAAGAAT TAGTAATCAG ATGATTCATG AATCGTCTGT TCAAATTCGA

Jarilla_chocola_Ycf1 ACGAAAAAAG ATTCAAGAAT TAGTAATCAG ATGATTCATG AATCATCTGT TCAAATTCGA

Jarilla_heterophella_Ycf1 ACGAAAAAAG ATTCAAGAAT TAGTAATCAG ATGATTCATG AATCGTCTGT TCAAATTCGA

Arabidopsis_thaliana_Ycf1 CCTAAAAAAG ATTCAATAAT AAGTAATCAG ATGATTCATG AACTATCTGT TCAAAATAAA

....|....| ....|....| ....|....| ....|....| ....|....| ....|....|

3125 3135 3145 3155 3165 3175

Carica_papaya_Ycf1 TCTATGGAGT GGACAAATTA TTCCCTGACA GAAAACAAAA TAAAAGATTT GACTGATAGA

Vasconcellea_Monoica_Ycf1 TCTATGGAGT GGAAAAATTA TTCCCTGACG GAAAACAAAA TAAAAGATTT GACTGATAGA

Jacaratia_spinosa_Ycf1 TCTATGGAGT GGACAAATTA TTCCCTGACA GAAAACAAAA TAAAAGATTT GACTGATAGA

Vasconcellea_pubescens_Ycf1 TCTATGGAGT GGAAAAATTA TTCCCTGACG GAAAACAAAA TAAAAGATTT GACTGATAGA

Jarilla_caudata_Ycf1 TCTATGGAGT GGACAAATTA TTCCCTGACA GAAAACAAAA TAAAAGATTT GACTGATAGA

Jarilla_chocola_Ycf1 TCTATGGAGT GGACAAATTA TTCCCTGACA GAAAACAAAA TAAAAGATTT GACTGATAGA

Jarilla_heterophella_Ycf1 TCTATGGAGT GGACAAATTA TTCCCTGACA GAAAACAAAA TAAAAGATTT GACTGATAGA

Arabidopsis_thaliana_Ycf1 TCCTTGGAGT GGACAAATTC TTCACTTAGC GAAAAAAAAA TCAAAAATTT GATTGATAGA

....|....| ....|....| ....|....| ....|....| ....|....| ....|....|

3185 3195 3205 3215 3225 3235

Carica_papaya_Ycf1 GCAAGTACAA TCAGAAATCA AATAGAACAA ATTACAAAAG AGAAGAAAAA CGGATTTATA

Vasconcellea_Monoica_Ycf1 GCAAGCACAA TCAGAAATCA AATAGAACAA ATTACAAAAG AGAAGAAAAA TGGATTTATA

Jacaratia_spinosa_Ycf1 GCAAGCACAA TCAGAAATCA AATAGAACAA ATTACAAAAG AGAAGCAAAA CGGATTTATA

Vasconcellea_pubescens_Ycf1 GCAAGCACAA TCAGAAATCA AATAGAACAA ATTACAAAAG AGAAGAAAAA CGGATTTATA

Jarilla_caudata_Ycf1 GCAAGCACAA TCAGAAATCA AATAGAACAA ATTACAAAAG AGAAGAAAAA CGGATTTGTA

Jarilla_chocola_Ycf1 GCAAGCACAA TCAGAAATCA AATAGAACAA ATTACAAAAG AGAAGAAAAA CGGATTTGTA

Jarilla_heterophella_Ycf1 GCAAGCACAA TCAGAAATCA AATAGAACAA ATTACAAAAG AGAAGAAAAA CGGATTTGTA

Arabidopsis_thaliana_Ycf1 AAAAAGACAA TCAGAAATCA AATAGAAGAA ATTTCAAAAG AAAAACAAAA CT--------

....|....| ....|....| ....|....| ....|....| ....|....| ....|....|

3245 3255 3265 3275 3285 3295

Carica_papaya_Ycf1 ACCCAAACTC AAGAAATAAA TATTAGTTCT AACAAACCAA GTTATCATGC TAAAATATTA

Vasconcellea_Monoica_Ycf1 ACCCAAACTC AAGAAATAAA TATGAGTTCT AACAAACCAA GTTATGATGC TAAAATATTA

Jacaratia_spinosa_Ycf1 ACCCAAACTC AAGAAATAAA TATTAGTTCT AACAAACCAA GTTATCATGC TAAACTATTA

Vasconcellea_pubescens_Ycf1 ACCCAAACTC AAGAAATAAA TATTAGTTCT AACAAACCAA GTTATCATGC TAAAATATTA

Jarilla_caudata_Ycf1 ACCCAAACTC AAGAAATAAA TATTAGTTCT AACAAACCAA GTTATCATGC TAAAATATTA

Jarilla_chocola_Ycf1 ACCCAAACTC AAGAAATAAA TATTAGTTCT AACAAACCAA GTTATCATGC TAAAATATTA

Jarilla_heterophella_Ycf1 ACCCAAACTC AAGAAATAAA TATTAGTTCT AACAAACCAA GTTATCATGC TAAAATATTA

Arabidopsis_thaliana_Ycf1 ---------- ------TAAC TAATAGTTGT ACCAAACTGC GTTATGATTC TAAAATAATT

....|....| ....|....| ....|....| ....|....| ....|....| ....|....|

3305 3315 3325 3335 3345 3355

Carica_papaya_Ycf1 GCATCATCAA AAAATATTTG GCAAATATTA AAAAGAAAAA ATGCTCGATT AATTCGTAAA

Vasconcellea_Monoica_Ycf1 GCATCATCAA AAAATATTTG GCAAATATTA AAAAGAAAAA ATACTCGATT AATTCGTAAA

Jacaratia_spinosa_Ycf1 GCATCATCAA AAAATATTTG GCAAATATTA AAAAGAAAAA ATGCTCGATT AATTCGTAAA

Vasconcellea_pubescens_Ycf1 GCATCATCAA AAAATATTTG GCAAATATTA AAAAGAAAAA ATGCTCGATT AATTCGTAAA

Jarilla_caudata_Ycf1 GCATCATCAA AAAATATTTG GCAAATATTA AAAAGAAAAA ATGCTCGATT AATTCGTAAA

Jarilla_chocola_Ycf1 GCATCATCAA AAAATATTTG GCAAATATTA AAAAGAAAAA ATGCTCGATT AATTCGTAAA

Jarilla_heterophella_Ycf1 GCATCATCAA AAAATATTTG GCAAATATTA AAAAGAAAAA ATGCTCGATT AATTCGTAAA

Arabidopsis_thaliana_Ycf1 GAGTCATCAA AAAAAATTTG GCAGACATTC AAAAGAAAAA ATACCCGATT AATTCGTAAA

....|....| ....|....| ....|....| ....|....| ....|....| ....|....|
3365 3375 3385 3395 3405 3415

Carica_papaya_Ycf1 TCATATTATT TTATAAAATT TTTCATTGAA AGGATATATA TAGATATTTT TCTATGTATC
Vasconcellea_Monoica_Ycf1 TCATATTATT TTATAAAATT TTTCAGTGAA AGGATATATA TAGATATTTT TCTATGTATC
Jacaratia_spinosa_Ycf1 TCATATTATT TTATAAAATT TTTCATTGAA AGGATATATA TAGATATTTT TCTATGTATC
Vasconcellea_pubescens_Ycf1 TCATATTATT TTATAAAATT TTTCAGTGAA AGGATATATA TAGATATTTT TCTATGTATC
Jarilla_caudata_Ycf1 TCATATTATT TTATAAAATT TTTCATTGAA AGGATATATA TAGATATTTT TCTATGTATC
Jarilla_chocola_Ycf1 TCATATTATT TTATAAAATT TTTCATTGAA AGGATATATA GAGATATTTT TCTATGTATC
Jarilla_heterophella_Ycf1 TCATATTATT TTATAAAATT TTTCATTGAA AGGATATATA TAGATATTTT TCTATGTATC
Arabidopsis_thaliana_Ycf1 TCCATTTTTT TTTTTAAATT TTGCATCGAA CAAATGTCTA TAGCTATTTT TCTAGGTATC

....|....| ....|....| ....|....| ....|....| ....|....| ....|....|
3425 3435 3445 3455 3465 3475

Carica_papaya_Ycf1 GTTAATATTC CAAGGATGAA TACACAACTT TTTCTTGAAT CAACAAAAAA AAATTTTGAT
Vasconcellea_Monoica_Ycf1 GTTAATATTC CAAGGATGAA TACACAACTT TTTGTTGAAT CAACAAAAAA AATTATTGAT
Jacaratia_spinosa_Ycf1 GTTAATATTC CAAGGATGAA TACACAACTT TTTCTTGAAT CAACAAAAAA AATTATTGAT
Vasconcellea_pubescens_Ycf1 GTTAATATTC CAAGGATGAA TACACAACTT TTTGTTGAAT CAACAAAAAA AATTATTGAT
Jarilla_caudata_Ycf1 GTTAATATTC TGAGGATGAA TACACAACTT TTTTTTGAAT CAACAAACAA AATTATTGAT
Jarilla_chocola_Ycf1 GTTAATATTC CGAGGATGAA TACACAACTT TTTTTTGAAT CAACAAACAA AATTATTGAT
Jarilla_heterophella_Ycf1 GTTAATATTC TGAGGATGAA TACACAACTT TTTTTTGAAT CAACAAACAA AATTATTGAT
Arabidopsis_thaliana_Ycf1 ATTAATATTC CAAGAATTAC TACACAACTT TTTTTTGAAT CAACAAAAAA AATTCTTGAT

....|....| ....|....| ....|....| ....|....| ....|....| ....|....|
3485 3495 3505 3515 3525 3535

Carica_papaya_Ycf1 AAATACATTT ACAATAATGA AGTAAATCAA GAAAGAATTA ATAAAACAAA ------TAAA
Vasconcellea_Monoica_Ycf1 AAATATATTT ACACTAATGA AGTAAATCAA GAAAGAATTA ATAAAACAAA ------TAAA
Jacaratia_spinosa_Ycf1 AAATACATTT ACAATACTGA AATAAATCAA GAAAGAATTA ATAAAACAAA ------TAAA
Vasconcellea_pubescens_Ycf1 AAATATATTT ACACTAATGA AGTAAATCAA GAAAGAATTA ATAAAACAAA ------TAAA
Jarilla_caudata_Ycf1 AAATACATTT ACAATAATGA AGTAAATCAA GAAAGAATTA ATAAAACAAA AACAAATAAA
Jarilla_chocola_Ycf1 AAATACATTT ACAATAATGA AGTAAATCAA GAAAGAATTA ATAAAACAAA AACAAATAAA
Jarilla_heterophella_Ycf1 AAATACATTT ACAATAATGA AGTAAATCAA GAAAGAATTA ATAAAACAAA AACAAATAAA
Arabidopsis_thaliana_Ycf1 AAATATATTT ACAAGAATGA AGAAAATGGA GAAAAAA--- ---AAA---- --------AA

....|....| ....|....| ....|....| ....|....| ....|....| ....|....|
3545 3555 3565 3575 3585 3595

Carica_papaya_Ycf1 AATACCAGTC AATTTATTTC GAATATAAAA AACTCACTTT CTAATACTAA TATTAGAAAT
Vasconcellea_Monoica_Ycf1 AATACCATTC AATTTATTTG GAATATAAAA AATTCACTTT CTAATACTAA TATTAGAAAT
Jacaratia_spinosa_Ycf1 AATACCATTC AATTTATTTG GAATATAAAA AACTCACTTT CTAATACTAA TATTAGAAAT
Vasconcellea_pubescens_Ycf1 AATACCATTC AATTTATTTG GAATATCAAA AATTCACTTT CTAATACTAA TATTAGAAAT
Jarilla_caudata_Ycf1 AATACCATTC AATTTATTTC GAATATAAAA AATTCACTTT CTAATACTAA TATTCGAAAT
Jarilla_chocola_Ycf1 AATACCATTC AATTTATTTC GAATATAAAA AACTCACTTT CTAATACTAA TATTCGAAAT
Jarilla_heterophella_Ycf1 AATACCATTC AATTTATTTC GAATATAAAA AACTCACTTT CTAATACTAA TATTCGAAAT

Arabidopsis_thaliana_Ycf1 AATACTCTTT ATTTTATTTC GACTATAAAA AATTTAATAT CCAATAAAAA ----AAAAAT

....|....| ....|....| ....|....| ....|....| ....|....| ....|....|
3605 3615 3625 3635 3645 3655

Carica_papaya_Ycf1 AAGAATTCAA AAAATTGTTG TAATCTATCC TCCTTCTCAC AAGCATATGT ATTTTACAAA
Vasconcellea_Monoica_Ycf1 AAGAATTCAC AAAATTGTTG TAATCTATCC TCTTTCTCAC AAGCAGATGT ATTTTACAAA
Jacaratia_spinosa_Ycf1 AAGAATTCAC AAAATTGGTG TAATCTATCC TCCTTCTCAC AAGCATATGT ATTTTACAAA
Vasconcellea_pubescens_Ycf1 AAGAATTCAC AAAATTGTTG TAATCTATCC TCTTTCTCAC AAGCATATGT ATTTTACAAA
Jarilla_caudata_Ycf1 AAGAATTCAC AAAATTCTTG TAAGCTATCC TCCTTCTCAC AAGCATATGT ATTTTTTAAA
Jarilla_chocola_Ycf1 AAGAATTCAC AAAATTCTTG TAAGCTATCC TCCTTCTCAC AAGCATATGT ATTTTTTAAA
Jarilla_heterophella_Ycf1 AAGAATTCAC AAAATTCTTG TAAGCTATCC TCCTTCTCAC AAGCATATGT ATTTTTTAAA
Arabidopsis_thaliana_Ycf1 GAG------- -------TTA TGACCTATGC TCTTTATCAC AAGCATATGT ATTTTACAAA

....|....| ....|....| ....|....| ....|....| ....|....| ....|....|
3665 3675 3685 3695 3705 3715

Carica_papaya_Ycf1 TTATCACAAA TAAAAGTTAT TAACTTGTAT AAGTTAAGGT CTGTCCTTCA ATATCAATAT
Vasconcellea_Monoica_Ycf1 TTATTACAAC TAAAAGTTAT TAACTTGTAT AAGTTAAGGT CTGTCCTTCA ATATCACTAT
Jacaratia_spinosa_Ycf1 TTATCACAAA TAAAAGTTAT TAACTTGTAT AAGTTAAGGT CTGTCCTTCA ATATCACTAT
Vasconcellea_pubescens_Ycf1 TTATTACAAC TAAAAGTTAT TAACTTGTAT AAGTTAAGAT CTGTCCTTCA ATATCACTAT
Jarilla_caudata_Ycf1 TTATCACAAA TAAAAGTTAT TAACTTGGAT AAGTTAAGGT CTGTCCTTCA ATATCAATAT
Jarilla_chocola_Ycf1 TTATCACAAA TAAAAGTTAT TAACTTGGAT AAGTTAAGGT CTGTCCTTCA ATATCAATAT
Jarilla_heterophella_Ycf1 TTATCACAAA TAAAAGTTAT TAACTTGGAT AAGTTAAGGT CTGTCCTTCA ATATCAATAT
Arabidopsis_thaliana_Ycf1 TTATCACAAA TTAAAGTTAG TAACTTTTGT AAATTAAAGG CTGTTCTTGA ATATAACATA

....|....| ....|....| ....|....| ....|....| ....|....| ....|....|
3725 3735 3745 3755 3765 3775

Carica_papaya_Ycf1 AACGGAACAT CTTTTTTTCT TAAGAATGAA ATAAAGGATT CTTTTGAAGA ACAAGGAATA
Vasconcellea_Monoica_Ycf1 AACGGAACAT CTTTTTTTCT TAAGAATGAA ATAAAGGATT TTTTTGAAGA ACAAGGAATA
Jacaratia_spinosa_Ycf1 AACGGAACAT CTTTTTTTCT TAAGAATGAA ATAAAGGGTT CTTTTGAAGA ACAAGGAATA
Vasconcellea_pubescens_Ycf1 AACGGAACAT CTTTTTTTCT TAAGAATGAA ATAAAGGATT TTTTTGAAGA ACAAGGAATA
Jarilla_caudata_Ycf1 AACGGAACAT CGTTTTTTCT TAAGAATGAA ATAAAGGATT CTTTTGAAGA ACAAGGAATA
Jarilla_chocola_Ycf1 AACGGAACAT CGTTTTTTCT TAAGAATGAA ATAAAGGATT CTTTTGAAGA ACAAGGAATA
Jarilla_heterophella_Ycf1 AACGGAACAT CGTTTTTTCT TAAGAATGAA ATAAAGGATT CTTTTGAAGA ACAAGGAATA
Arabidopsis_thaliana_Ycf1 TGCATAACAT CCTTTTTTGT TAAGAATAAA ATAAAGGTTT TTTTTCAAGA ACACGGAATC

....|....| ....|....| ....|....| ....|....| ....|....| ....|....|
3785 3795 3805 3815 3825 3835

Carica_papaya_Ycf1 TTTGATTACC AATTAAGACA TAAACCCCCT TTTAATTACG AAAAAAATCT ATGGAAAAAC
Vasconcellea_Monoica_Ycf1 TTTGATTACA AATTAAGACA TAAACCCTTT TTTAATCACG AAAAAAATCT ATGGAAAAAC
Jacaratia_spinosa_Ycf1 TTTGATTACA AATTAAGACA TAAACCCCTT TTTAATCACG AAAAAAATCT ATGGAAAAAC
Vasconcellea_pubescens_Ycf1 TTTGATTACA AATTAAGACA TAAACCCTTT TTTAATCACG AAAAAAATCT ATGGAAAAAC
Jarilla_caudata_Ycf1 TTTGATTACA AATTAAGACA TAAACCCCCT TTTAATTACG AAAAGAATCT ATGGAAAAAC
Jarilla_chocola_Ycf1 TTTGATTACA AATTAAGACA TAAACCCCCT TTTAATTACG AAAAGAATCT ATGGAAAAAC
Jarilla_heterophella_Ycf1 TTTGATTACA AATTAAGACA TAAACCCCCT TTTAATTACG AAAAGAATCT ATGGAAAAAC

Arabidopsis_thaliana_Ycf1 TTTCATTATG AATTGAAAAA TAAAACCTTT TTGAATTCCG AAGTAAATCA ATGGAAAAAC

....|....| ....|....| ....|....| ....|....| ....|....| ....|....|
3845 3855 3865 3875 3885 3895

Carica_papaya_Ycf1 TGGTTAAGAA GTCATTATCA ATATGATTTA CCTCAGATTA GATGGCCTAG ATTAGTACCA
Vasconcellea_Monoica_Ycf1 TGGTTAAGAA GTCATTATCA ATATGATTTA CCTCAGATTA GATGGCCTAG ATTAGTACCA
Jacaratia_spinosa_Ycf1 TGGTTAAGAA GTCATTATCA ATATGATTTA CCTCAGATTA GATGGCCTAG ATTAGTCCCA
Vasconcellea_pubescens_Ycf1 TGGTTAAGAA GTCATTATCA ATATGATTTA CCTCAGATTA GATGGCCTAG ATTAGTACCA
Jarilla_caudata_Ycf1 TGGTTAAGAA GTCATTATCA ATATGATTTA CCTCAGATTA GATGGCCTAG ATTAGTACCA
Jarilla_chocola_Ycf1 TGGTTAAGAA GTCATTATCA ATATGATTTA CCTCAGATTA GATGGCCTAG ATTAGTACCA
Jarilla_heterophella_Ycf1 TGGTTAAGAA GTCATTATCA ATATGATTTA CCTCAGATTA GATGGCCTAG ATTAGTACCA
Arabidopsis_thaliana_Ycf1 TGGTTACGAA GTCAGTATCA ATACAATTTA CCCCAGATTT CATGGGCTAG ATTAGTAACC

....|....| ....|....| ....|....| ....|....| ....|....| ....|....|
3905 3915 3925 3935 3945 3955

Carica_papaya_Ycf1 GAAAAATGGC GAAATAGAGT CAAAGAATAC TGTAGAGTTC AAAATAAAGA TTTAAACAAA
Vasconcellea_Monoica_Ycf1 GAAAAATGGC GAAATAGAGT CAAAGAATAC TGTAGAGTTC AAAATCAAGA TTTAAACAAA
Jacaratia_spinosa_Ycf1 GAAAAATGGC GAAATAGAGT CAAAGAATAC TGTAGAGTTC AAAATAAAGA TTTAAACAAA
Vasconcellea_pubescens_Ycf1 GAAAAATGGC GAAATAGAGT CAAAGAATAC TGTAGAGTTC AAAATAAAGA TTTAAACAAA
Jarilla_caudata_Ycf1 GAAAAATGGC GAAATAGAGT CAAAGAATAC CGTAGAGTTC AAAATAAAGA TTTAAACAAA
Jarilla_chocola_Ycf1 GAAAAATGGC GAAATAGAGT CAAAGAATAC CATAGAGTTC AAAATAAAGA TTTAAAGAAA
Jarilla_heterophella_Ycf1 GAAAAATGGC GAAATAGAGT CAAAGAATAC CGTAGAGTTC AAAATAAAGA TTTAAACAAA
Arabidopsis_thaliana_Ycf1 CAAAATTGGA AAAATAAAAT AAATAAAGAT TCTCTAGTTC TAAACCCAAG TTTAACCAAA

....|....| ....|....| ....|....| ....|....| ....|....| ....|....|
3965 3975 3985 3995 4005 4015

Carica_papaya_Ycf1 TGGGATTCAT ATGAAAAAGA CAAATTAAGT CATTACGAAA GACAAAACTC TTTTGAAGTG
Vasconcellea_Monoica_Ycf1 TGGGATTCAT ATGAAAAAGA CAAATTAATT CATTACAAAA GACAAAACTT TTTTGAAGTG
Jacaratia_spinosa_Ycf1 TGGGATTCAT ATGAAAAAGA CAAATTAATT CATTACGAAA GACAAAACTT TTTTGAAGTG
Vasconcellea_pubescens_Ycf1 TGGGATTCAT ATGAAAAAGA CAAATTAATT CATTACAAAA GACAAAACTT TTTTGAAGTG
Jarilla_caudata_Ycf1 TGGGATTCAT ATGAAAAAGA CAAATTAATT CATTACGAAA AACAAAACTT TTTTGAAGTG
Jarilla_chocola_Ycf1 TGGGATTCAT ATGAAAAAGA CAAATTAATT CATTACGAAA GACAAAACCT TTTTGAAGTG
Jarilla_heterophella_Ycf1 TGGGATTCAT ATGAAAAAGA CAAATTAATT CATTACGAAA AACAAAACTT TTTTGAAGTG
Arabidopsis_thaliana_Ycf1 GAGGATTCAT ATGAAAAAAA GAAATTTGAT AATTATAAAA AACAAAAATT TTTTGAGGCC

....|....| ....|....| ....|....| ....|....| ....|....| ....|....|
4025 4035 4045 4055 4065 4075

Carica_papaya_Ycf1 GACTCATTGC TGAATCAAAA ATCGAATTTC AAAAAAAATG ATAGATATGA TCTTTTATCA
Vasconcellea_Monoica_Ycf1 GACTCATTGC TGAATCAAAA ATCTAATTTC AAAAAAAATT ATAGATATGA TCTTTTATCA
Jacaratia_spinosa_Ycf1 GACTCATTGC TGAATCAAAA ATCGAATTTA AAAAAAAATT ATAGATATGA TCTTTTATCA
Vasconcellea_pubescens_Ycf1 GACTCATTGC TGAATCAAAA ATCTAATTTC AAAAAAAATG ATAGATATGA TCTTTTATCA
Jarilla_caudata_Ycf1 GACTCATTGC TGAATCAAAA ATCGAATTTA AAAAAGAATT ATAGATATGA TCTTTTATCA
Jarilla_chocola_Ycf1 GACTCATTGC TGAATCAAAA ATCGAATTTA AAAAAGAATT ATAGATATGA TCTTTTATCA
Jarilla_heterophella_Ycf1 GACTCATTGC TGAATCAAAA ATCGAATTTA AAAAAGAATT ATAGATATGA TCTTTTATCA

Arabidopsis_thaliana_Ycf1 GACTCATTAT TAAATCCAAA ACATAATGTA AAAAAAGATT CTATATATAA TCTTTTTTGC

....|....| ....|....| ....|....| ....|....| ....|....| ....|....|
4085 4095 4105 4115 4125 4135

Carica_papaya_Ycf1 TCTAAATCTA TTAATTCTAA AGACAAGATT TTTTTTAATT ACAACATGGA TAAACAGAAA
Vasconcellea_Monoica_Ycf1 TCTAAATCTA TTAATTCTAA AGACACGGTT TTTTATAATT ACAACATGGA TAAACAGAAA
Jacaratia_spinosa_Ycf1 TCTAAATCTA TTAATTCGAA AGACAAGATT TTTTATAATT ACAACATGGA TAAACAGAAA
Vasconcellea_pubescens_Ycf1 TCTAAATCTA TTAATTCTAA AGACACGGTT TTTGATAATT ACAACATGGA TAAACAGAAA
Jarilla_caudata_Ycf1 TCTAAATCTA TTAATTCTAA AGACAAGATT TTGTATAATT ACAACATGGA TAAACAGAAA
Jarilla_chocola_Ycf1 TCTAAATCTA TTAATTCTAA AGACAAGATT TTGTATAATT ACAACATGGA TAAACAGAAA
Jarilla_heterophella_Ycf1 TCTAAATCTA TTAATTCTAA AGACAAGATT TTGTATAATT ACAACATGGA TAAACAGAAA
Arabidopsis_thaliana_Ycf1 TATAAATCTA TTCATTCTAC AGA------- ---------- ---------- ------AAAA

....|....| ....|....| ....|....| ....|....| ....|....| ....|....|
4145 4155 4165 4175 4185 4195

Carica_papaya_Ycf1 TTTTTTGATA TCCTCGTAGG TATCCCTATC CATAATTATC TAGTCTCTAA TTATTTCGGA
Vasconcellea_Monoica_Ycf1 TTTTTTGATA TCCTCGTAGG TATTCCTAGC CATAATTATC TAGTCTCTAA TTATTTAGGA
Jacaratia_spinosa_Ycf1 TTTTTTGATA TCCTCGTAGG TATCCCTATC CATAATTATC TAGTCTCTAA TTATTTAGGA
Vasconcellea_pubescens_Ycf1 TTTTTTGATA TCCTCGTAGG TATTCCTAGC CATAATTATC TAGTCTCTAA TTATTTAGGA
Jarilla_caudata_Ycf1 TTCTTTGATA TCCTCGTAGG TATCCCTATC CATAATTATC TAGTCTCTAA TTATTTAGGA
Jarilla_chocola_Ycf1 TTCTTTGATA CCCTCGTAGG TATCCCTATC CATAATTATC TAGTCTCTAA TTATTTAGGA
Jarilla_heterophella_Ycf1 TTCTTTGATA TCCTCGTAGG TATCCCTATC CATAATTATC TAGTCTCTAA TTATTTAGGA
Arabidopsis_thaliana_Ycf1 AATTTTGACA TGTCTATAGG CATTGCCCTA GATAATTGTT TAGTCTCTTC TTTTCTAGAA

....|....| ....|....| ....|....| ....|....| ....|....| ....|....|
4205 4215 4225 4235 4245 4255

Carica_papaya_Ycf1 GAAGATCATA TTTTTGATAG CGAGAAAATT CTGGATCGAA AATATTTAGA TTGGAGAATT
Vasconcellea_Monoica_Ycf1 GAAGAGCATA TTTTTGATAG CGAGAAAATT CTGTATCGAA AATATTTAGA TTGGAGAATT
Jacaratia_spinosa_Ycf1 GAAGATCATA TTTTTGATAG CGAGAAAATT CTGGATCGAA AATATTTAGA TTGGAGAATT
Vasconcellea_pubescens_Ycf1 GAAGAGCATA TTTTTGATAG CGAGAAAATT CTGTATCGAA AATATTTAGA TTGGAGAATT
Jarilla_caudata_Ycf1 GAAGATGATA TTTTTGATAG CGAGAAAATT ATGGATCGAA AATATTTAGA TTGGAGAATT
Jarilla_chocola_Ycf1 GAAGATGATA TTTTTGATAG CGAGAAAATT ATGGATCGAA AATATTTAGA TTGGAGAATT
Jarilla_heterophella_Ycf1 GAAGATGATA TTTTTGATAG CGAGAAAATT ATGGATCGAA AATATTTAGA TTGGAGAATT
Arabidopsis_thaliana_Ycf1 AAATATAATA TTCGGGGTAT GGGGGAAATT CGGCATAGAA AATATTTGGA TTGGAGAATT

....|....| ....|....| ....|....| ....|....| ....|....| ....|....|
4265 4275 4285 4295 4305 4315

Carica_papaya_Ycf1 CTCAACTTTT GTCTTAGAAA CAAGGTCGAT ATTGAGTCGT GGGTTGATAT CGATACTAAG
Vasconcellea_Monoica_Ycf1 CTCAACTTTT GTCTTAGAAA CAAAGTCGAT ATTGAGTCTT GGGTTGATAT CGATACTAAG
Jacaratia_spinosa_Ycf1 CTCAACTTTT GTCTTAGAAA CAAAGTCGAT ATTGAGTCTT GGGTTGATAT CGATACTAAG
Vasconcellea_pubescens_Ycf1 CTCAACTTTT GTCTTAGAAA CAAAGTCGAT ATTGAGTCTT GGGTTGATAT CGATACTAAG
Jarilla_caudata_Ycf1 CTCAACTTTA GTCTTAGAAA CAAGGTCGAT ATTGAGTCTT GGGTTGATAT CGATACTAAG
Jarilla_chocola_Ycf1 CTCAACTTTA GTCTTAGAAA CAAGGTCGAT ATTGAGTCTT GGGTTGATAT CGATACTAAG
Jarilla_heterophella_Ycf1 CTCAACTTTA GTCTTAGAAA CAAGGTCGAT ATTGAGTCTT GGGTTGATAT CGATACTAAG

```
Arabidopsis_thaliana_Ycf1       CTTAACTTTT GGTTTACAAA AAAAGTAACT ATTGAGCCTT GGGTTGATAC TAAGAGTAAA

                                ....|....| ....|....| ....|....| ....|....| ....|....| ....|....|
                                    4325       4335       4345       4355       4365       4375
Carica_papaya_Ycf1              AGTAATAAAA ATATTAAGAC TGGAGTTAAT AATTATCAAA TTATTGATAA AATTAATAAG
Vasconcellea_Monoica_Ycf1       AGTAATAAAA ATATTAAGAC TGGAGTTAAT AATTATCAAA TTCTTGATAA AATTAATAAG
Jacaratia_spinosa_Ycf1          AGTAATAAAA ATATTAAGAC TGGAGTTAAT AATTATCAAA TTATTGATAA AATTAATAAG
Vasconcellea_pubescens_Ycf1     AGTAATAAAA ATATTAAGAC TGGAGTTAAT AATTATAAAA TTCTTGATAA AATTAATAAG
Jarilla_caudata_Ycf1            AGTAATACAA ATATTAAAAC TGGAGTTAAG AATTATCAAA TTTTTGATAA AATTAATAAG
Jarilla_chocola_Ycf1            AGTAATACAA ATATTAAAAA TGGAGTTAAG AATTATCAAA TTTTTGATAA AATTAATAAG
Jarilla_heterophella_Ycf1       AGTAATACAA ATATTAAAAC TGGAGTTAAG AATTATCAAA TTTTTGATAA AATTAATAAG
Arabidopsis_thaliana_Ycf1       A---AAAAAT ATATTAATAC TAAAGTTCAG AATTATCAAA AAATTGATAA AATAACGCAG

                                ....|....| ....|....| ....|....| ....|....| ....|....| ....|....|
                                    4385       4395       4405       4415       4425       4435
Carica_papaya_Ycf1              AA-------- -GAAGGGTCT TTTATATTTC ACAATTCATC AAGATCAAGA AATCAAACCT
Vasconcellea_Monoica_Ycf1       AA-------- -GAAGGGTCT TTTTTATTTC ACAATTCATC AAGATCAAGA ATTCAAACCA
Jacaratia_spinosa_Ycf1          AATAATAAGA AGAAGGGTCT TTTTTATTTC ACAATTCATC AAGATCAAGA AATCAAACCT
Vasconcellea_pubescens_Ycf1     AA-------- -GAAGGGTCT TTTTTATTTC ACAATTCATC AAGATCAAGA ATTCAAACCA
Jarilla_caudata_Ycf1            AG-------- -GAAGAGTCT TCTTTATTTC ACAATTCATC AAGATCAAGA AATCAAACCT
Jarilla_chocola_Ycf1            AG-------- -GAAGAGTCT TCTTTATTTC ACAATTCATC AAGATCAAGA AATCAAACCT
Jarilla_heterophella_Ycf1       AG-------- -GAAGAGTCT TCTTTATTTC ACAATTCATC AAGATCAAGA AATCAAACCT
Arabidopsis_thaliana_Ycf1       AC-------- ----GGATCT TG-------- ---------- ---------- ----------

                                ....|....| ....|....| ....|....| ....|....| ....|....| ....|....|
                                    4445       4455       4465       4475       4485       4495
Carica_papaya_Ycf1              TTCAATCCAA AAAGTTTCTT TTTTGATTGG ATGGGAATGA ATGAAGAAAT ACTAAGTC--
Vasconcellea_Monoica_Ycf1       TCCAATCAAA AAAATTTATT TTTTGATTGG ATGGGAATGA ATGAAGAAAT ACTAAGTT--
Jacaratia_spinosa_Ycf1          TCCAATCCAA AAAATTTCTT TTTTGATTGG ATGGGAATGA ATGAAGAAAT ACTAAGTC--
Vasconcellea_pubescens_Ycf1     TCCAATCAAA AAAATTTATT TTTTGATTGG ATGGGAATGA ATGAAGAAAT ACTAAGTT--
Jarilla_caudata_Ycf1            TCCAATCAAA AAAGGAACTT TTTGGATTGG ATGGGAATGA ATGAAGAAAT ACTAAGTC--
Jarilla_chocola_Ycf1            TCCAATCCAA AAAGTTCCTT TTTTGATTGG ATGGGAATGA ATGAAGAAAT ACTAAGTC--
Jarilla_heterophella_Ycf1       TCCAATCAAA AAAGGAACTT TTTGGATTGG ATGGGAATGA ATGAAGAAAT ACTAAGTC--
Arabidopsis_thaliana_Ycf1       -CTAATAAAA AAAGAAACTT TTTTGATTGG ATGGGAATGA ATGAAGAAAT ACTAAATCAG

                                ....|....| ....|....| ....|....| ....|....| ....|....| ....|....|
                                    4505       4515       4525       4535       4545       4555
Carica_papaya_Ycf1              -GTCTATCGA ATCTAGAACT TTGGTTCTTT CCCGAATTTG TCTTACTTTA TAATGCATAT
Vasconcellea_Monoica_Ycf1       -GTCTATCGA ATCTAGAATT TTGGTTCTTT CCCGAATTTG TCTTACTTTA TAATGCATAT
Jacaratia_spinosa_Ycf1          -GTCTATCGA ATCTAGAATT TTGGTTCTTT CCCGAATTTG TCTTACTTTA TAATGCATAT
Vasconcellea_pubescens_Ycf1     -GTCTATCGA ATCTAGAATT TTGGTTCTTT CCCGAATTTG TCTTACTTTA TAATGCATAT
Jarilla_caudata_Ycf1            -GTCTATCGA ATCTAGAACT TTGGTTCTTT CCCGAATTTG TCTTACTTTA TAATGCATAT
Jarilla_chocola_Ycf1            -GTCTATCGA ATCTAGAACT TTGGTTCTTT CCCGAATTTG TCTTACTTTA TAATGCATAT
Jarilla_heterophella_Ycf1       -GTCTATCGA ATCTAGAACT TTGGTTCTTT CCCGAATTTG TCTTACTTTA TAATGCATAT
```

```
Arabidopsis_thaliana_Ycf1        CGTATAACAA ATTTTGAATT TTTTTTCTTT CCGGAATTTT TCTTATTTTC TAGTACATAT

                                 ....|....| ....|....| ....|....| ....|....| ....|....| ....|....|
                                      4565       4575       4585       4595       4605       4615
Carica_papaya_Ycf1               AAGATTAAAC CGTGGATCAT ACCAAGCAAT TTACTTCTTT TTAATTTTAA TGAAAATGAA
Vasconcellea_Monoica_Ycf1        AAGATTAAAC CGTGGATCAT ACCAAGCAAT TTACTTCTTT TTAATTTTAA TGAAAATGAA
Jacaratia_spinosa_Ycf1           AAGATTAAAC CGTGGATCAT ACCAAGCAAT TTATTTCTTT TGAATTTTAA TGAAAATGAA
Vasconcellea_pubescens_Ycf1      AAGATTAAAC CGTGGATCAT ACCAAGCAAT TTACTTCTTT TTAATTTTAA TGAAAATGAA
Jarilla_caudata_Ycf1             AAGATTAAAC CGTGGATCAT ACCAAGCAAT TTACTTCTTT TTAATTTTAA TGAAAATGAA
Jarilla_chocola_Ycf1             AAGATTAAAC CGTGGATCAT ACCAAGCAAT TTACTTCTTT TTAATTTTAA TGAAAATGAA
Jarilla_heterophella_Ycf1        AAGATTAAAC CGTGGATCAT ACCAAGCAAT TTACTTCTTT TTAATTTTAA TGAAAATGAA
Arabidopsis_thaliana_Ycf1        AAAATGAAAC CATGGGTCAT ACCAATCAAA TTACTTCTTT TAAATTTTAA TGAAAACATA

                                 ....|....| ....|....| ....|....| ....|....| ....|....| ....|....|
                                      4625       4635       4645       4655       4665       4675
Carica_papaya_Ycf1               AACCTTAATA AAAGCATCAC GGGAAAGAAA AAGGGTTTTA TATCATCGAA TGAAAAACAA
Vasconcellea_Monoica_Ycf1        AACCTTAAGA AAAGCATTAC TGGAACGAAA AAGGGTTTTA TAGCATCGAA TGAAAAACAA
Jacaratia_spinosa_Ycf1           AACCTTAATA AAAGCATCAC TGGAAAGAAA AAGGGTTTTA TATCATCGAA TGAAAAACAA
Vasconcellea_pubescens_Ycf1      AACCTTAAGA AAAGCATTAC TGGAACGAAA AAGGGTTTTA TAGCATCGAA TGAAAAACAA
Jarilla_caudata_Ycf1             AACCTTAATA AAAGCATCAC TGGAAAGAAA AAGGATTTTA TATCATCGAA TGAAAAACAA
Jarilla_chocola_Ycf1             AACCTTAATA AAAGCATCAC TGGAAAGAAA AAGGATTTTA TATCATCGAA TGAAAAACAA
Jarilla_heterophella_Ycf1        AACCTTAATA AAAGCATCAC TGGAAAGAAA AAGGATTTTA TATCATCGAA TGAAAAACAA
Arabidopsis_thaliana_Ycf1        AATGTTAATA AAAAGATCAT TCGAAAGAAA AAAGGTTTTA TACCATCAAA TGAAAAAGAA

                                 ....|....| ....|....| ....|....| ....|....| ....|....| ....|....|
                                      4685       4695       4705       4715       4725       4735
Carica_papaya_Ycf1               TTTCTTGGAT TTGGATTCGA AAATCCAAAT CACGAAGAAA GAGACCCCGC GTACCAAGGG
Vasconcellea_Monoica_Ycf1        TTTCTTGGAT TTGGATTCGA AAATCCAAAT CAAGAAGAAA GAGACCCCAC ATACCAAGGG
Jacaratia_spinosa_Ycf1           TTTCTTGGAT TTGGATTCGA AAATCCAAAT CAAGAAGAAG GAGACCCCAC ATACCAAGGG
Vasconcellea_pubescens_Ycf1      TTTCTTGGAT TTGGATTCGA AAATCCAAAT CAAGAAGAAA GAGACCCCAC ATACCAAGGG
Jarilla_caudata_Ycf1             TTTCTTGGAT TTGGATTAGA AAATCCAAAG CAAGAAGAAA GAGACCCCGC ATATCAAGGG
Jarilla_chocola_Ycf1             TTTCCTGGAT TTGGATTAGA AAATCCAAAT CAAGAAGAAA GAGACCCCGC ATATCAAGGG
Jarilla_heterophella_Ycf1        TTTCTTGGAT TTGGATTAGA AAATCCAAAG CAAGAAGAAA GAGACCCCGC ATATCAAGGG
Arabidopsis_thaliana_Ycf1        TCCCTTCGAT TTT------A TAATCTGAAT AAAGAAGAAA AAGAATCGGC CGGTCAAGTA

                                 ....|....| ....|....| ....|....| ....|....| ....|....| ....|....|
                                      4745       4755       4765       4775       4785       4795
Carica_papaya_Ycf1               GAGCTTGGAC CAGATGAACA AAAACAAGGG AATCTTGGAT CAATTCTCTC AAACCAAGAA
Vasconcellea_Monoica_Ycf1        GAGCTTGGAT CAGATGAACA AAAACAAGGA AATCTTGGAT CCATTCTCTC AAATCAAGAA
Jacaratia_spinosa_Ycf1           GAGCTTGGAT CAGATGAACA AAAACAAGGA AATCTTGGAT CAATTCTCTC AAACCAAGAA
Vasconcellea_pubescens_Ycf1      GAGCTTGGAT CAGATGAACA AAAACAAGGA AATCTTGGAT CCATTCTCTC AAATCAAGAA
Jarilla_caudata_Ycf1             GAGCTTGGAT CAGATGAACA AAAACAAGGA AATCTTGGAT CAATTCTCTC AAACCAAGAA
Jarilla_chocola_Ycf1             GAGCTTGGAT CAGATGAACA AAAACAAGGA AATCTTGGAT CAATTCTCTC AAACCAAGAA
Jarilla_heterophella_Ycf1        GAGCTTGGAT CAGATGAACA AAAACAAGGA AATCTTGGAT CAATTCTCTC AAACCAAGAA
```

Arabidopsis_thaliana_Ycf1	GAACTTGAAT CAGATAAAGA AACAAAAAGA AATCCCGAAG CAGCTCGATT AAACCAAGAA

	....|....| ....|....| ....|....| ....|....| ....|....| ....|....|
	4805 4815 4825 4835 4845 4855
Carica_papaya_Ycf1	AAAAATATTG AAGAAGATTA TGCAGAATCA GACA------ ---------- ----------
Vasconcellea_Monoica_Ycf1	ACAACTATTG AAGAAGATTA TGCAGAATCA GACA------ ---------- ----------
Jacaratia_spinosa_Ycf1	AAAAATATTG AAGAAGATTA TGCAGAATCA GACA------ ---------- ----------
Vasconcellea_pubescens_Ycf1	ACAACTATTG AAGAAGATTA TGCGGAATCA GACA------ ---------- ----------
Jarilla_caudata_Ycf1	AAAAATATTG AAGAAGATTA TGCAGAATCA GACAATATTG AAGAAGATTA TGCAGAATCA
Jarilla_chocola_Ycf1	AAAAATATTG AAGAAGATTA TGCAGAATCA GACAATATTG AAGAAGATTA TGCAGAATCA
Jarilla_heterophella_Ycf1	AAAAATATTG AAGAAGATTA TGCAGAATCA GACAATATTG AAGAAGATTA TGCAGAATCA
Arabidopsis_thaliana_Ycf1	AAAAATATTG AAGAAAATTT TGCAGAATCA ACAA------ ---------- ----------

	....|....| ....|....| ....|....| ....|....| ....|....| ....|....|
	4865 4875 4885 4895 4905 4915
Carica_papaya_Ycf1	----TCAAAA AACGTAGAAA GAAAAAGCAA TACAAAAGCA ATACAGAAGC AGAACTTCAT
Vasconcellea_Monoica_Ycf1	----TAAAAA AACGTAGAAA GAAAAAGCAA TACAAAAGCA ATACCGAAGC AGAACTTCAT
Jacaratia_spinosa_Ycf1	----TAAAAA AACGTAGAAA GAAAAAGCAA TACAAAAGCA ATACCGAAGC AGAACTTCAT
Vasconcellea_pubescens_Ycf1	----TAAAAA AACGTAGAAA GAAAAAGCAA TACAAAAGCA ATACCGAAGC AGAACTTCAT
Jarilla_caudata_Ycf1	GACATAAAAA AACGTAGAAA GAAAAACCAA TACAAAAACA ATACAGAAGC AGAACTTTAT
Jarilla_chocola_Ycf1	GACATAAAAA AACGTAGAAA GAAAAACCAA TACAAAAACA ATACAGAAGC AGAACTTTAT
Jarilla_heterophella_Ycf1	GACATAAAAA AACGTAGAAA GAAAAACCAA TACAAAAACA ATACAGAAGC AGAACTTTAT
Arabidopsis_thaliana_Ycf1	----TAAAAA AACGTAAAAA TAAAAAACAA TACAAAAGTA ATACAGAAGC GGAACTTGAT

	....|....| ....|....| ....|....| ....|....| ....|....| ....|....|
	4925 4935 4945 4955 4965 4975
Carica_papaya_Ycf1	TTCTTCCTAA AAAGATATTT GCGTTTTCAA TTGAGATGGA ATGATTCTTT AAATGAAAGA
Vasconcellea_Monoica_Ycf1	TTCTTCCTAA AAAGATATTT GCGTTTTCAA TTGAGATGGA ATGATTCTTT AAATCACAGA
Jacaratia_spinosa_Ycf1	TTCTTCCTAA AAAGATATTT GCGTTTTCAA TTGAGATGGA ATGATTCTTT AAATCAAAGA
Vasconcellea_pubescens_Ycf1	TTCTTCCTAA AAAGATATTT GCGCTTTCAA TTGAGATGGA ATGATTCTTT AAATCACAGA
Jarilla_caudata_Ycf1	TTCTTCCTAA AAAGATATTT GCGTTTTCAA TTGAGATGGA ATGATTCTTT AAATGAAAGA
Jarilla_chocola_Ycf1	TTCTTCCTAA AAAGATATTT GCGTTTTCAA TTGAGATGGA ATGATTCTTT AAATGAAAGA
Jarilla_heterophella_Ycf1	TTCTTCCTAA AAAGATATTT GCGTTTTCAA TTGAGATGGA ATGATTCTTT AAATGAAAGA
Arabidopsis_thaliana_Ycf1	TTATTTCTGA CAAGATATTC GCGTTTTCAA TTGCGATGGA ATTGTTTTTT TAATCAAAAA

	....|....| ....|....| ....|....| ....|....| ....|....| ....|....|
	4985 4995 5005 5015 5025 5035
Carica_papaya_Ycf1	ATTATCAATA ATATCAAAGT ATATTGTCTC TTGCTTAGAC TGATAAATCC AAGAGAAATT
Vasconcellea_Monoica_Ycf1	ATTATCAATA ATATCAAAGT ATATTGTCTC TTGCTTAGAC TGATAAATCC AAGAGAAATT
Jacaratia_spinosa_Ycf1	ATTATCAATA ATATCAAAGT ATATTGTCTC TTGCTTAGAC TGATAAATCC AAGAGAAATT
Vasconcellea_pubescens_Ycf1	ATTATCAATA ATATCAAAGT ATATTGTCTC TTGCTTAGAC TGATAAATCC AAGAGAAATT
Jarilla_caudata_Ycf1	ATTATCAATA ATATCAAAGT ATATTGTCTC TTGCTTAGAC TGATAAATCC AAGAGAAATT
Jarilla_chocola_Ycf1	ATTATCAATA ATATCAAAGT ATATTGTCTC TTGCTTAGAC TGATAAATCC AAGAGAAATT
Jarilla_heterophella_Ycf1	ATTATCAATA ATATCAAAGT ATATTGTCTC TTGCTTAGAC TGATAAATCC AAGAGAAATT

Arabidopsis_thaliana_Ycf1 ATTCTCAATA ATGTAAAAGT ATACTGTCTC TTGGTTAGAC TAAACAATCC AAACGAAATA

....|....| ....|....| ....|....| ....|....| ....|....| ....|....|

5045 5055 5065 5075 5085 5095

Carica_papaya_Ycf1 ACTATATCTT CTATTCAAAG GGGAGAACTG AGTCTAGATA TCCTGATGAT TCAGAAGGAT

Vasconcellea_Monoica_Ycf1 ACTATATCTT CTATTCAAAG GGGAGAAATG AGTCTAGATA TCCTAATGAG TCAGAAGGAT

Jacaratia_spinosa_Ycf1 ACTATATCTT CTATTCAAAG GGGAGAAATG AGTCTAGATA TCCTAATGAT TCAGAAGGAT

Vasconcellea_pubescens_Ycf1 ACTATATCTT CTATTCAAAG GGGAGAAATG AGTCTAGATA TCCTAATGAG TCAGAAGGAT

Jarilla_caudata_Ycf1 GCTATATCTT CTATTCAAAG GGGAGAAATG AGTCTAGATA TCCTGATGAT TCAGAAGGAT

Jarilla_chocola_Ycf1 GCTATATCTT CTATTCAAAG GGGAGAAATG AGTCTAGATA TCCTGATGAT TCAGAAGGAT

Jarilla_heterophella_Ycf1 GCTATATCTT CTATTCAAAG GGGAGAAATG AGTCTAGATA TCCTGATGAT TCAGAAGGAT

Arabidopsis_thaliana_Ycf1 GCGGTATCTT CTATTGAAAG AGGAGAGATG AGCCTAGACA TTCTAATGAT TGAGAAAAAT

....|....| ....|....| ....|....| ....|....| ....|....| ....|....|

5105 5115 5125 5135 5145 5155

Carica_papaya_Ycf1 TTAACTCTTC CAGAATTAAT GAAAAAGGGA ATATTGATTA TCGAACCAGT CCGTTTGTCT

Vasconcellea_Monoica_Ycf1 TTAACTCTTC CAGAATTAAT GAAAAAGGGA ATATTGATTA TCGAACCAGT TCGTTTGTCT

Jacaratia_spinosa_Ycf1 TTAACTCTTC CAGAATTAAT GAAAAAGGGA ATATTGATTA TCGAACCAGT TCGTTTGTCT

Vasconcellea_pubescens_Ycf1 TTAACTCTTC CAGAATTAAT GAAAAAGGGA ATATTGATTA TCGAACCAGT TCGTTTGTCT

Jarilla_caudata_Ycf1 TTAACTCTTC CAGAATTAAT GAAAAAGGGA ATATTGATTC TCGAACCAGT TCGTTTATCT

Jarilla_chocola_Ycf1 TTAACTCTTC CAGAATTAAT GAAAAAGGGA ATATTGATTC TCGAACCAGT TCGTTTATCT

Jarilla_heterophella_Ycf1 TTAACTCTTC CAGAATTAAT GAAAAAGGGA ATATTGATTC TCGAACCAGT TCGTTTATCT

Arabidopsis_thaliana_Ycf1 TTCACTTTTG CAAAATTAAT GAAAAAAGGA ATATTGATTA TTGAACCTGT GCGTTTGTCT

....|....| ....|....| ....|....| ....|....| ....|....| ....|....|

5165 5175 5185 5195 5205 5215

Carica_papaya_Ycf1 GTCAAAAACG ATGGACAATT TATTATATAT CAAACCATAG GTATTTCATT GGGTGATAAG

Vasconcellea_Monoica_Ycf1 GTCAAAAACG ATGGACAATT TATTATATAT CAAACCATAA GTATTTCATT GGTTGATAAG

Jacaratia_spinosa_Ycf1 GTCAAAAACG ATGGGCAATT TATTATATAT CAAACCATAG GTATTTCATT GGTTGATAAG

Vasconcellea_pubescens_Ycf1 GTCAAAAACG ATGGACAATT TATTATATAT CAAACCATAA GTATTTCATT GGTTGATAAG

Jarilla_caudata_Ycf1 GTCAAAAACG ATGGACAATT TATTATCTAT CAAACCATAG GTATTTCATT GGTTAATACG

Jarilla_chocola_Ycf1 GTCAAAAACG ATGGACAATT TATTATCTAT CAAACCATAG GTATTTCATT GGTTGATACG

Jarilla_heterophella_Ycf1 GTCAAAAACG ATGGACAATT TATTATCTAT CAAACCATAG GTATTTCATT GGTTAATACG

Arabidopsis_thaliana_Ycf1 GTACAAAACG ATGGACAACT TATTATATAT AGAACCATAG GTATTTCATT GGTTCATAAA

....|....| ....|....| ....|....| ....|....| ....|....| ....|....|

5225 5235 5245 5255 5265 5275

Carica_papaya_Ycf1 AATAAGCCCC AAATAAAGCA AAGATACCAA GAAAAAAACT ATGTTGATAA AAAGAATTTT

Vasconcellea_Monoica_Ycf1 AATAAGCACC AAATAAAGCA AAGATACCAA GAAAAAAACT ATGTTGATAA AAATCATTTT

Jacaratia_spinosa_Ycf1 AATAAGCACC AAATAAAGCA AAGATACCAA GAAAAAAACT ATGTTGATAA AAAGAATTTT

Vasconcellea_pubescens_Ycf1 AATAAGCACC AAATAAAGCA AAGATACCAA GAAAAAAACT ATGTTGATAA AAATCATTTT

Jarilla_caudata_Ycf1 AATAAGCAAC AAATAAAGCA AAGATACCAA GAAAAAAACT ATGTTGATAA AAATCATTTT

Jarilla_chocola_Ycf1 AATAAGCAAC AAATAAAGCA AAGATACCAA GAAAAAAACT ATGTTGATAA AAATCATTTT

Jarilla_heterophella_Ycf1 AATAAGCAAC AAATAAAGCA AAGATACCAA GAAAAAAACT ATGTTGATAA AAATCATTTT

Arabidopsis_thaliana_Ycf1 AATAAACACA AAATAAGTAA AAGATACAAA AAAAAAAGCT ATATTAATAA AAAATTTTTT

....|....| ....|....| ....|....| ....|....| ....|....| ....|....|

5285 5295 5305 5315 5325 5335

Carica_papaya_Ycf1 GATGAATCCA TTGCAAGACA TCAAAGAATG ACTGGAAATA GAGAAAAAAA TCATTATGAT

Vasconcellea_Monoica_Ycf1 GATGAATCCA TTGCAAGATA TCAAAGACTG ACTGGAAATA GAGAAAAAAA TCATTATGAT

Jacaratia_spinosa_Ycf1 GATGAATCCA TTGCAAGACA TCAAAGAATG ACTGGAAATA GAGAAAAAAA TCATTATGAT

Vasconcellea_pubescens_Ycf1 GATGAATCCA TTGCAAGACA TCAAAGACTG ACTGGCAATA GAGAAAAAAA TCATTATGAT

Jarilla_caudata_Ycf1 GATGAATCCA TTGCAAGACA TCAAAGAATG ACTGGAAATA GAGAAAAAAA TCATTATGAT

Jarilla_chocola_Ycf1 GATGAATCCA TTGCAAGACA TCAAAGAATG ACTGGAAATA GAGAAAAAAA TCATTATGAT

Jarilla_heterophella_Ycf1 GATGAATCCA TTGCAAGACA TCAAAGAATG ACTGGAAATA GAGAAAAAAA TCATTATGAT

Arabidopsis_thaliana_Ycf1 GAAAAATCCA TTACAAAATA TCAAAACAAA ACTGTAAATA AAAAAAAAAA TAATTATGAT

....|....| ....|....| ....|....| ....|....| ....|....| ....|....|

5345 5355 5365 5375 5385 5395

Carica_papaya_Ycf1 TTCCTTGTCC CTGAAAATAT TTTATCCCCT AAATGTCGTA GAGAATTTCG AATTCTAATT

Vasconcellea_Monoica_Ycf1 TTCCTTGTCC CGGAAAAGAT TTTATCCCCG AAATGTCGTC GAGAATTTCG AATTCTAATT

Jacaratia_spinosa_Ycf1 TTCCTTGTCC CTGAAAATAT TTTATCCCCT AAATGTCGTA GAGAATTTCG AATTCTAATT

Vasconcellea_pubescens_Ycf1 TTCCTTGTCC CGGAAAAGAT TTTATCCCCG AAATGTCGTC GAGAATTTCG AATTCTAATT

Jarilla_caudata_Ycf1 TTCCTTGTCC CTGAAAATAT TTTATCCCCT AAATGTCGTA GAGAATTTAG AATTCTAATT

Jarilla_chocola_Ycf1 TTCCTTGTCC CTGAAAATAT TTTATCCCCT AAATGTCGTA GAGAATTTAG AATTCTAATT

Jarilla_heterophella_Ycf1 TTCCTTGTCC CTGAAAATAT TTTATCCCCT AAATGTCGTA GAGAATTTAG AATTCTAATT

Arabidopsis_thaliana_Ycf1 TTCTTTGTCC CTGAAAAAAT TCTATCCCCT AAACGACGTA GAGAATTTCG AATTCTAATT

....|....| ....|....| ....|....| ....|....| ....|....| ....|....|

5405 5415 5425 5435 5445 5455

Carica_papaya_Ycf1 TGTTTCAATT CAAAGAATAG AAATGATATG CGTAGAAAGA CAAAATTTCA CAATAACATA

Vasconcellea_Monoica_Ycf1 TGTTTCAACT CAAAGAATAG AAATGATATG TGTAGAAAGA CACAATTTCA CAATACCATA

Jacaratia_spinosa_Ycf1 TGTTTCAATT CAAAGAATAG AAATGATATG CGTAGAAAGA CAAAATTTCA CAATACCATA

Vasconcellea_pubescens_Ycf1 TGTTTCAACT CAAAGAATAG AAATGATATG CGTAGAAAGA CACAATTTCA CAATACCATA

Jarilla_caudata_Ycf1 TGTTTCAATT CACAGAATAG AAATGATATG TGTAGAAAGA CCAAATTTCA CAATAACATA

Jarilla_chocola_Ycf1 TGTTTCAATT CACAGAATAG AAATGATATG TGTAGAAAGA CCAAATTTCA CAATAACATA

Jarilla_heterophella_Ycf1 TGTTTCAATT CACAGAATAG AAATGATATG TGTAGAAAGA CCAAATTTCA CAATAACATA

Arabidopsis_thaliana_Ycf1 TGTTTCAACT TAAAAAAAAA AAATGCTAGG GATACAAATT CAAGATTTGA TAAGAATATT

....|....| ....|....| ....|....| ....|....| ....|....| ....|....|

5465 5475 5485 5495 5505 5515

Carica_papaya_Ycf1 AAAAACTGTG GTCAA-GTTT TGAATACAAC CAAAGATTTT GAT------A GAAATAAAAA

Vasconcellea_Monoica_Ycf1 AAAAACGGTG GTCAA-GTTT TGAATACAAG CAAAGATTTT GAT------A GAAATAAAAA

Jacaratia_spinosa_Ycf1 AAAAACTGTG GTCAA-GTTT TGAATACAAG CAAAGATTTT GAT------A GAAATAAAAA

Vasconcellea_pubescens_Ycf1 AAAAACGGTG GTCAA-GTTT TGAATACAAG CAAAGATTTT GAT------A GAAATAAAAA

Jarilla_caudata_Ycf1 AAAAACTGTG GTCAA-GTTT TGAATACAAC CAAAGATTTT GAT------A AAAATAAAAA

Jarilla_chocola_Ycf1 AAAAACTGTG GTCAA-GTTT TGAATACAAC CAAAGATTTT GAT------A AAAATAAAAA

Jarilla_heterophella_Ycf1 AAAAACTGTG GTCAA-GTTT TGAATACAAC CAAAGATTTT GAT------A AAAATAAAAA

Arabidopsis_thaliana_Ycf1 CAAAACT-TG ACCACCGTTT TGCATAAAAA GAAAGATCTT GATCTTGATA AGGATAAAAA

....|....| ....|....| ....|....| ....|....| ....|....| ....|....|

5525 5535 5545 5555 5565 5575

Carica_papaya_Ycf1 TACCCTAATT AAATCAAAGT TCTTTCTTTG GCCCAATTTT CGATTAGAAG ATTTAGCTTG

Vasconcellea_Monoica_Ycf1 TAACCTAATT AAATCAAAGT TCTTTCTTTG GCCCAATTTT CGATTAGAAG ATTTAGCTTG

Jacaratia_spinosa_Ycf1 TAACCTAATT AAATCAAAGT TCTTTCTTTG GCCCAATTTT CGATTAGAAG ATTTAGCTTG

Vasconcellea_pubescens_Ycf1 TAACCTAATT AAATCAAAGT TCTTTCTTTG GCCCAATTTT CGATTAGAAG ATTTAGCTTG

Jarilla_caudata_Ycf1 TAACCTAATT AAATCAAAGC TCTTTCTTTG GCCCAATTTT CGATTAGAAG ATTTAGCTTG

Jarilla_chocola_Ycf1 TAACCTAATT AAATCAAAGC TCTTTCTTTG GCCCAATTTT CGATTAGAAG ATTTAGCTTG

Jarilla_heterophella_Ycf1 TAACCTAATT AAATCAAAGC TCTTTCTTTG GCCCAATTTT CGATTAGAAG ATTTAGCTTG

Arabidopsis_thaliana_Ycf1 TAACTTAATT AATTTAAAAT CCTTTCTTTG GCCCAATTTT AAATTAGAAG ATTTAGCTTG

....|....| ....|....| ....|....| ....|....| ....|....| ....|....|

5585 5595 5605 5615 5625 5635

Carica_papaya_Ycf1 TATGAATCGC TATTGGTTTA ATACTAATAA TGGTAGTCGT TTCAGTATGA TAAGGATACA

Vasconcellea_Monoica_Ycf1 TATGAATCGC TATTGGTTTA ATACTAATAA TGGTAGTCGT TTCAGTATGA TAAGGATACA

Jacaratia_spinosa_Ycf1 TATGAATCGC TATTGGTTTA ATACTAATAA TGGTAGTCGT TTCAGTATGA TAAGGATACA

Vasconcellea_pubescens_Ycf1 TATGAATCGC TATTGGTTTA ATACTAATAA TGGTAGTCGT TTCAGTATGA TAAGGATACA

Jarilla_caudata_Ycf1 TATGAATCGC TATTGGTTTA ATACTAATAA TGGTAGTCGT TTCAGTATGA TAAGGATACA

Jarilla_chocola_Ycf1 TATGAATCGC TATTGGTTTA ATACTAATAA TGGTAGTCGT TTCAGTATGA TAAGGATACA

Jarilla_heterophella_Ycf1 TATGAATCGC TATTGGTTTA ATACTAATAA TGGTAGTCGT TTCAGTATGA TAAGGATACA

Arabidopsis_thaliana_Ycf1 TATGAATCGC TATTGGTTTA ATACTACTAA CGGAAATCAT TTCAGTATGA TAAGAATACG

....|....| ....|....| ....|...

5645 5655 5665

Carica_papaya_Ycf1 TATGTATCCG CGATTAAAAA CTCCTTGA

Vasconcellea_Monoica_Ycf1 TATGTATCCG CGATTAAAAA CTCCTTGA

Jacaratia_spinosa_Ycf1 TATGTATCCG CGATTAAAAA CTCCTTGA

Vasconcellea_pubescens_Ycf1 TATGTATCCG CGATTAAAAA CTCCTTGA

Jarilla_caudata_Ycf1 TATGTATCCG CGATTAAAAA CTCCTTGA

Jarilla_chocola_Ycf1 TATGTATCCG CGATTAAAAA CTCCTTGA

Jarilla_heterophella_Ycf1 TATGTATCCG CGATTAAAAA CTCCTTGA

Arabidopsis_thaliana_Ycf1 CATGTATACG CGATTTCCAA TTCCTTAA
