# Supplementary material for: Comparative analysis of chloroplast genomes in Vasconcellea pubescens A.DC. and Carica papaya L
Source: Sci Rep. 2020 Sep 25;10:15799. doi: 10.1038/s41598-020-72769-y (PMC7519098; doi:10.1038/s41598-020-72769-y)
Supplement: Supplementary file 6 — Supplementary Information 6. [file 41598_2020_72769_MOESM6_ESM.pdf]

Comparative analysis of chloroplast genomes in *Vasconcellea pubescens* A.DC. and *Carica papaya* L.

Zhicong Lin[1] , Ping Zhou[3], Xinyi Ma[2], Youjin Deng[2], Zhenyang Liao[2],

Ruoyu Li[2] and Ray Ming[4,1 *]

[1]College of Agriculture, Center for Genomics and Biotechnology, Fujian Provincial Key Laboratory of Haixia Applied Plant Systems Biology, Fujian Agriculture and Forestry University, Fuzhou, Fujian 350002, China.

[2]College of Life Sciences, Fujian Agriculture and Forestry University, Fuzhou 350002, Fujian, China.

[3]Fruit Research Institute，Fujian Academy of Agricultural Sciences，Fuzhou 350013，Fujian, China

[4]Department of Plant Biology, University of Illinois at Urbana-Champaign, Urbana, IL 61801, USA.

[*]rayming@illinois.edu

Indels and SNPs profile of *Ycf1* gene in three genera of family Lauraceae

	.... |.... |	.... |.... |	.... |.... |	.... |.... |	.... |.... |	.... |.... |
	5	15	25	35	45	55
Machilus_pauhoi	ATGATTTTGA	AATCTTTTCT	ACTAGGTAAT	CTATTATCCT	TATGCATGAA	GATAATAAAT
Machilus_balansae	ATGATTTTGA	AATCTTTTCT	ACTAGGTAAT	CTATTATCCT	TATGCATGAA	GATAATAAAT
Machilus_yunnanensis	ATGATTTTGA	AATCTTTTCT	ACTAGGTAAT	CTATTATCCT	TATGCATGAA	GATAATAAAT
Machilus_thunbergii	ATGATTTTGA	AATCTTTTCT	ACTAGGTAAT	CTATTATCCT	TATGCATGAA	GATAATAAAT
Persea_americana	ATGATTTTGA	AATCTTTTCT	ACTAGGTAAT	CTATTATCCT	TATGCATGAA	GATAATAAAT
Lindera_glauca	ATGATTTTGA	AATCTTTTCT	ACTAGGTAAT	CTATTATCCT	TATGCATGAA	GATAATAAAT
Lindera_sericea	ATGATTTTGA	AATCTTTTCT	ACTAGGTAAT	CTATTATCCT	TATGCATGAA	GATAATAAAT
Lindera_megaphylla	ATGATTTTGA	AATCTTTTCT	ACTAGGTAAT	CTATTATCCT	TATGCATGAA	GATAATAAAT

	.... |.... |	.... |.... |	.... |.... |	.... |.... |	.... |.... |	.... |.... |
	65	75	85	95	105	115
Machilus_pauhoi	TCGGTCGTTG	TGGTCGGACT	CTATTATGGA	TTTCTGACCA	CATTCTCCAT	AGGGCCCTCT
Machilus_balansae	TCGGTCGTTG	TGGTCGGACT	CTATTATGGA	TTTCTGACCA	CATTCTCCAT	AGGGCCCTCT
Machilus_yunnanensis	TCGGTCGTTG	TGGTCGGACT	CTATTATGGA	TTTCTGACCA	CATTCTCCAT	AGGGCCCTCT
Machilus_thunbergii	TCGGTCGTTG	TGGTCGGACT	CTATTATGGA	TTTCTGACCA	CATTCTCCAT	AGGGCCCTCT
Persea_americana	TCGGTCGTTG	TGGTCGGACT	CTATTATGGA	TTTCTGACCA	CATTCTCCAT	AGGGCCCTCT
Lindera_glauca	TCGGTCGTTG	TGGTCGGACT	CTATTATGGA	TTTCTGACCA	CATTCTCCAT	AGGGCCCTCT
Lindera_sericea	TCGGTCGTTG	TGGTCGGACT	CTATTATGGA	TTTCTGACCA	CATTCTCCAT	AGGGCCCTCT
Lindera_megaphylla	TCGGTCGTTG	TGGTCGGACT	CTATTATGGA	TTTCTGACCA	CATTCTCCAT	AGGGCCCTCT

	.... |.... |	.... |.... |	.... |.... |	.... |.... |	.... |.... |	.... |.... |
	125	135	145	155	165	175
Machilus_pauhoi	TATCTTTTCC	TTCTCCGAGC	TAGGATTATG	GAAGAAGGAA	CCGAGAAGGA	GGTATCAGCA
Machilus_balansae	TATCTTTTCC	TTCTCCGAGC	TAGGATTATG	GAAGAAGGAA	CCGAGAAGGA	GGTATCAGCA
Machilus_yunnanensis	TATCTTTTCC	TTCTCCGAGC	TAGGATTATG	GAAGAAGGAA	CCGAGAAGGA	GGTATCAGCA
Machilus_thunbergii	TATCTTTTCC	TTCTCCGAGC	TAGGATTATG	GAAGAAGGAA	CCGAGAAGGA	GGTATCAGCA
Persea_americana	TATCTTTTCC	TTCTCCGAGC	TAGGATTATG	GAAGAAGGAA	CCGAGAAGGA	GGTATCAGCA
Lindera_glauca	TATCTTTTCC	TTCTCCGAGC	TAGGATTATG	GAAGAAGGAA	CCGAGAAGGA	GGTATCAGCA
Lindera_sericea	TATCTTTTCC	TTCTCCGAGC	TAGGATTATG	GAAGAAGGAA	CCGAGAAGGA	GGTATCAGCA
Lindera_megaphylla	TATCTTTTCC	TTCTCCGAGC	TAGGATTATG	GAAGAAGGAA	CCGAGAAGGA	GGTATCAGCA

	.... |.... |	.... |.... |	.... |.... |	.... |.... |	.... |.... |	.... |.... |
	185	195	205	215	225	235
Machilus_pauhoi	ACAACTGGTT	TTATTACGGG	ACAGCTCATT	ATGTTCATAT	CGATCTATTA	TGCGCCTCTG
Machilus_balansae	ACAACTGGTT	TTATTACGGG	ACAGCTCATG	ATGTTCATAT	CGATCTATTA	TGCGCCTCTG
Machilus_yunnanensis	ACAACTGGTT	TTATTACGGG	ACAGCTCATT	ATGTTCATAT	CGATCTATTA	TGCGCCTCTG
Machilus_thunbergii	ACAACTGGTT	TTATTACGGG	ACAGCTCATT	ATGTTCATAT	CGATCTATTA	TGCGCCTCTG

Persea_americana ACAACTGGTT TTATTACGGG ACAGCTCATG ATGTTCATAT CGATCTATTA TGCGCCTCTG

Lindera_glauca ACAACTGGTT TTATTACGGG ACAGCTCATG ATGTTCATAT CGATCTATTA TGCGCCTCTG

Lindera_sericea ACAACTGGTT TTATTACGGG ACAGCTCATG ATGTTCATAT CGATCTATTA TGCGCCTCTG

Lindera_megaphylla ACAACTGGTT TTATTACGGG ACAGCTCATG ATGTTCATAT CGATCTATTA TGCGCCTCTG

....|....| ....|....| ....|....| ....|....| ....|....| ....|....|

245 255 265 275 285 295

Machilus_pauhoi CATCTAGCAT TGGGTAGACC TCATACAATA ACTGTCCTAG TTCTACCGTA TCTTTTGTTT

Machilus_balansae CATCTAGCAT TGGGTAGACC TCATACAATA ACTGTCCTAG TTCTACCGTA TCTTTTGTTT

Machilus_yunnanensis CATCTAGCAT TGGGTAGACC TCATACAATA ACTGTCCTAG TTCTACCGTA TCTTTTGTTT

Machilus_thunbergii CATCTAGCAT TGGGTAGACC TCATACAATA ACTGTCCTAG TTCTACCGTA TCTTTTGTTT

Persea_americana CATCTAGCAT TGGGTAGACC TCATACAATA ACTGTCCTAG TTCTACCGTA TCTTTTGTTT

Lindera_glauca CATCTAGCAT TGGGTAGACC TCATACAATA ACTGTCCTAG TTCTACCGTA TCTTTTGTTT

Lindera_sericea CATCTAGCAT TGGGTAGACC TCATACAATA ACTGTCCTAG TTCTACCGTA TCTTTTGTTT

Lindera_megaphylla CATCTAGCAT TGGGTAGACC TCATACAATA ACTGTCCTAG TTCTACCGTA TCTTTTGTTT

....|....| ....|....| ....|....| ....|....| ....|....| ....|....|

305 315 325 335 345 355

Machilus_pauhoi CATTTCTTCT GGAACAATCA CAAACACTTT TTGGATTATG GATCTACTAC CAGAAATTCA

Machilus_balansae CATTTCTTCT GGAACAATCA CAAACACTTT TTGGATTATG GATCTACTAC CAGAAATTCA

Machilus_yunnanensis CATTTCTTCT GGAACAATCA CAAACACTTT TTGGATTATG GATCTACTAC CAGAAATTCA

Machilus_thunbergii CATTTCTTCT GGAACAATCA CAAACACTTT TTGGATTATG GATCTACTAC CAGAAATTCA

Persea_americana CATTTCTTCT GGAACAATCA CAAACACTTT TTGGATTATG GATCTACTAC CAGAAATTCA

Lindera_glauca CATTTCTTCT GGAACAATCA CAAACACTTT TTGGATTATG GATCTACTAC CAGAAATTCA

Lindera_sericea CATTTCTTCT GGAACAATCA CAAACACTTT TTGGATTATG GATCTACTAC CAGAAATTCA

Lindera_megaphylla CATTTCTTCT GGAACAATCA CAAACACTTT TTGGATTATG GATCTACTAC CAGAAATTCA

....|....| ....|....| ....|....| ....|....| ....|....| ....|....|

365 375 385 395 405 415

Machilus_pauhoi ATGCGTAATC TCAGCATTCA ATGTGTATTC CTGAATAATC TCATTTTTCA ATTATTCAAC

Machilus_balansae ATGCGTAATC TCAGCATTCA ATGTGTATTC CTGAATAATC TCATTTTTCA ATTATTCAAC

Machilus_yunnanensis ATGCGTAATC TCAGCATTCA ATGTGTATTC CTGAATAATC TCATTTTTCA ATTATTCAAC

Machilus_thunbergii ATGCGTAATC TCAGCATTCA ATGTGTATTC CTGAATAATC TCATTTTTCA ATTATTCAAC

Persea_americana ATGCGTAATC TCAGCATTCA ATGTGTATTC CTGAATAATC TCATTTTTCA ATTATTCAAC

Lindera_glauca ATGCGTAATC TCAGCATTCA ATGTGTATTC CTGAATAATC TCATTTTTCA ATTATTCAAC

Lindera_sericea ATGCGTAATC TCAGCATTCA ATGTGTATTC CTGAATAATC TCATTTTTCA ATTATTCAAC

Lindera_megaphylla ATGCGTAATC TCAGCATTCA ATGTGTATTC CTGAATAATC TCATTTTTCA ATTATTCAAC

....|....| ....|....| ....|....| ....|....| ....|....| ....|....|

425 435 445 455 465 475

Machilus_pauhoi CATTTCATTT TACCAAGTTC AACGTTAGTC AGATTAGTCA ACATTTATAT GTTTCGATGC

Machilus_balansae CATTTCATTT TACCAAGTTC AACGTTAGTC AGATTAGTCA ACATTTATAT GTTTCGATGC

Machilus_yunnanensis CATTTCATTT TACCAAGTTC AACGTTAGTC AGATTAGTCA ACATTTATAT GTTTCGATGC

Machilus_thunbergii CATTTCATTT TACCAAGTTC AACGTTAGTC AGATTAGTCA ACATTTATAT GTTTCGATGC

```
Persea_americana        CATTTCATTT TACCAAGTTC AACGTTAGTC AGATTAGTCA ACATTTATAT GTTTCGATGC
Lindera_glauca          CATTTCATTT TACCAAGTTC AACGTTAGTC AGATTAGTCA ACATTTATAT GTTTCGATGC
Lindera_sericea         CATTTCATTT TACCAAGTTC AACGTTAGTC AGATTAGTCA ACATTTATAT GTTTCGATGC
Lindera_megaphylla      CATTTCATTT TACCAAGTTC AACGTTAGTC AGATTAGTCA ACATTTATAT GTTTCGATGC

                        ....|....| ....|....| ....|....| ....|....| ....|....| ....|....|
                            485        495        505        515        525        535
Machilus_pauhoi         AACAACAAGA TGTTATTTGT AACAAGTAGT TTTGTTGGTT GGTTAATTGG TCACATTTTC
Machilus_balansae       AACAACAAGA TGTTATTTGT AACAAGTAGT TTTGTTGGTT GGTTAATTGG TCACATTTTC
Machilus_yunnanensis    AACAACAAGA TGTTATTTGT AACAAGTAGT TTTGTTGGTT GGTTAATTGG TCACATTTTC
Machilus_thunbergii     AACAACAAGA TGTTATTTGT AACAAGTAGT TTTGTTGGTT GGTTAATTGG TCACATTTTC
Persea_americana        AACAACAAGA TGTTATTTGT AACAAGTAGT TTTGTTGGTT GGTTAATTGG TCACATTTTC
Lindera_glauca          AACAACAAGA TGTTATTTGT AACAAGTAGT TTTGTTGGTT GGTTAATTGG TCACATTTTC
Lindera_sericea         AACAACAAGA TGTTATTTGT AACAAGTAGT TTTGTTGGTT GGTTAATTGG TCACATTTTC
Lindera_megaphylla      AACAACAAGA TGTTATTTGT AACAAGTAGT TTTGTTGGTT GGTTAATTGG TCACATTTTC

                        ....|....| ....|....| ....|....| ....|....| ....|....| ....|....|
                            545        555        565        575        585        595
Machilus_pauhoi         TTCATGAAAT GGGTTGGATT GGTATTATTC TGGATACGGC AAAATCATTC TATTAGATCG
Machilus_balansae       TTCATGAAAT GGGTTGGATT GGTATTATTC TGGATACGGC AAAATCATTC TATTAGATCG
Machilus_yunnanensis    TTCATGAAAT GGGTTGGATT GGTATTATTC TGGATACGGC AAAATCATTC TATTAGATCG
Machilus_thunbergii     TTCATGAAAT GGGTTGGATT GGTATTATTC TGGATACGGC AAAATCATTC TATTAGATCG
Persea_americana        TTCATGAAAT GGGTTGGATT GGTATTATTC TGGATACGGC AAAATCATTC TATTAGATCG
Lindera_glauca          TTCATGAAAT GGGTTGGATT GGTATTATTC TGGATACGGC AAAATCATTC TATTAGATCG
Lindera_sericea         TTCATGAAAT GGGTTGGATT GGTATTATTC TGGATACGGC AAAATCATTC TATTAGATCG
Lindera_megaphylla      TTCATGAAAT GGGTTGGATT GGTATTATTC TGGATACGGC AAAATCATTC TATTAGATCG

                        ....|....| ....|....| ....|....| ....|....| ....|....| ....|....|
                            605        615        625        635        645        655
Machilus_pauhoi         AATGTACTTA TTCGATCTAA TAAGTACCTT GTGTCAGAAT TGAGAAATTC TATGGCTCGA
Machilus_balansae       AATGTACTTA TTCGATCTAA TAAGTACCTT GTGTCAGAAT TGAGAAATTC TATGGCTCGA
Machilus_yunnanensis    AATGTACTTA TTCGATCTAA TAAGTACCTT GTGTCAGAAT TGAGAAATTC TATGGCTCGA
Machilus_thunbergii     AATGTACTTA TTCGATCTAA TAAGTACCTT GTGTCAGAAT TGAGAAATTC TATGGCTCGA
Persea_americana        AATGTACTTA TTCGATCTAA TAAGTACCTT GTGTCAGAAT TGAGAAATTC TATGGCTCGA
Lindera_glauca          AATGTACTTA TTCGATCTAA TAAGTACCTT GTGTCAGAAT TGAGAAATTC TATGGCTCGA
Lindera_sericea         AATGTACTTA TTCGATCTAA TAAGTACCTT GTGTCAGAAT TGAGAAATTC TATGGCTCGA
Lindera_megaphylla      AATGTACTTA TTCGATCTAA TAAGTACCTT GTGTCAGAAT TGAGAAATTC TATGGCTCGA

                        ....|....| ....|....| ....|....| ....|....| ....|....| ....|....|
                            665        675        685        695        705        715
Machilus_pauhoi         ATCTTTACTA TTCTCTTATT TATCACCTGT GTCTACTATT TAGGCAGAAT ACCGTCGCCT
Machilus_balansae       ATCTTTACTA TTCTCTTATT TATCACCTGT GTCTACTATT TAGGCAGAAT ACCGTCGCCT
Machilus_yunnanensis    ATCTTTACTA TTCTCTTATT TATCACCTGT GTCTACTATT TAGGCAGAAT ACCGTCGCCT
Machilus_thunbergii     ATCTTTACTA TTCTCTTATT TATCACCTGT GTCTACTATT TAGGCAGAAT ACCGTCGCCT
```

Persea_americana ATCTTTACTA TTCTCTTATT TATCACCTGT GTCTACTATT TAGGCAGAAT ACCGTCGCCT

Lindera_glauca ATCTTTACTA TTCTCTTATT TATCACCTGT GTCTACTATT TAGGCAGAAT ACCGTCGCCT

Lindera_sericea ATCTTTACTA TTCTCTTATT TATCACCTGT GTCTACTATT TAGGCAGAAT ACCGTCGCCT

Lindera_megaphylla ATCTTTACTA TTCTCTTATT TATCACCTGT GTCTACTATT TAGGCAGAAT ACCGTCGCCT

....|....| ....|....| ....|....| ....|....| ....|....| ....|....|

725 735 745 755 765 775

Machilus_pauhoi ATTGTCACTA AGAAACTGAA AGAAACCTCA AAAACGGAAG AAAGGGGGGA AAGTGAGGAA

Machilus_balansae ATTGTCACTA AGAAACTGAA AGAAACCTCA AAAACGGAAG AAAGGGGGGA AAGTGAGGAA

Machilus_yunnanensis ATTGTCACTA AGAAACTGAA AGAAACCTCA AAAACGGAAG AAAGGGGGGA AAGTGAGGAA

Machilus_thunbergii ATTGTCACTA AGAAACTGAA AGAAACCTCA AAAACGGAAG AAAGGGGGGA AAGTGAGGAA

Persea_americana ATTGTCACTA AGAAACTGAA AGAAACCTCA AAAACGGAAG AAAGGGGGGA AAGTGAGGAA

Lindera_glauca ATTGTCACTA AGAAACTGAA AGAAACCTCA AAAACGGAAG AAAGGGGGGA AAGTGAGGAA

Lindera_sericea ATTGTCACTA AGAAACTGAA AGAAACCTCA AAAACGGAAG AAAGGGGGGA AAGTGAGGAA

Lindera_megaphylla ATTGTCACTA AGAAACTGAA AGAAACCTCA AAAACGGAAG AAAGGGGGGA AAGTGAGGAA

....|....| ....|....| ....|....| ....|....| ....|....| ....|....|

785 795 805 815 825 835

Machilus_pauhoi GAAACAGATG TAGAAATAGA AAAAACTTCC GAAACGAAGG GGACTAAACA GGAACAAGAG

Machilus_balansae GAAACAGATG TAGAAATAGA AAAAACTTCC GAAACGAAGG GGACTAAACA GGAACAAGAG

Machilus_yunnanensis GAAACAGATG TAGAAATAGA AAAAACTTCC GAAACGAAGG GGACTAAACA GGAACAAGAG

Machilus_thunbergii GAAACAGATG TAGAAATAGA AAAAACTTCC GAAACGAAGG GGACTAAACA GGAACAAGAG

Persea_americana GAAACAGATG TAGAAATAGA AAAAACTTCC GAAACGAAGG GGACTAAACA GGAACAAGAG

Lindera_glauca GAAACAGATG TAGAAATAGA AAAAACTTCC GAAACGAAGG GGACTAAACA GGAACAAGAG

Lindera_sericea GAAACAGATG TAGAAATAGA AAAAACTTCC GAAACGAAGG GGACTAAACA GGAACAAGAG

Lindera_megaphylla GAAACAGATG TAGAAATAGA AAAAACTTCC GAAACGAAGG GGACTAAACA GGAACAAGAG

....|....| ....|....| ....|....| ....|....| ....|....| ....|....|

845 855 865 875 885 895

Machilus_pauhoi GGATCCACCG AAGAAGACCC TTCCCTTTGT TCGGAAGAAA GGGAGGATCC AAAAAAACTA

Machilus_balansae GGATCCACCG AAGAAGACCC TTCCCTTTGT TCGGAAGAAA GGGAGGATCC AAAAAAACTA

Machilus_yunnanensis GGATCCACCG AAGAAGACCC TTCCCTTTGT TCGGAAGAAA GGGAGGATCC AAAAAAACTA

Machilus_thunbergii GGATCCACCG AAGAAGACCC TTCCCTTTGT TCGGAAGAAA GGGAGGATCC AAAAAAACTA

Persea_americana GGATCCACCG AAGAAGACCC TTCCCTTTGT TCGGAAGAAA GGGAGGATCC AAAAAAACTA

Lindera_glauca GGATCCACCG AAGAAGACCC TTCCCTTTGT TCGGAAGAAA GGGAGGATCC AAAAAAACTA

Lindera_sericea GGATCCACCG AAGAAGACCC TTCCCTTTGT TCGGAAGAAA GGGAGGATCC AAAAAAACTA

Lindera_megaphylla GGATCCACCG AAGAAGACCC TTCCCTTTGT TCGGAAGAAA GGGAGGATCC AAAAAAACTA

....|....| ....|....| ....|....| ....|....| ....|....| ....|....|

905 915 925 935 945 955

Machilus_pauhoi CATGAAAAAA AAAAGAGGCA AGAAATTTTG AAGTTAGAAA TACTTAAAGA GAAAGAAGAT

Machilus_balansae CATGAAAAAA AAAAGAGGCA AGAAATTTTG AAGTTAGAAA TACTTAAAGA GAAAGAAGAT

Machilus_yunnanensis CATGAAAAAA AAAAGAGGCA AGAAATTTTG AAGTTAGAAA TACTTAAAGA GAAAGAAGAT

Machilus_thunbergii CATGAAAAAA AAAAGAGGCA AGAAATTTTG AAGTTAGAAA TACTTAAAGA GAAAGAAGAT

Persea_americana CATGAAAAAA AAAAGAGGCA AGAAATTTTG AAGTTAGAAA TACTTAAAGA GAAAGAAGAT
Lindera_glauca CATGAAAAAA AAAAGAGGCA AGAAATTTTG AAGTTAGAAA TACTTAAAGA GAAAGAAGAT
Lindera_sericea CATGAAAAAA AAAAGAGGCA AGAAATTTTG AAGTTAGAAA TACTTAAAGA GAAAGAAGAT
Lindera_megaphylla CATGAAAAAA AAAAGAGGCA AGAAATTTTG AAGTTAGAAA TACTTAAAGA GAAAGAAGAT

....|....| ....|....| ....|....| ....|....| ....|....| ....|....|
965 975 985 995 1005 1015
Machilus_pauhoi AAAGACCTCT TCTGGTTTGA AAAACCTCTT GTGAATCTTC TTTTCGACTA TAAACGATGT
Machilus_balansae AAAGACCTCT TCTGGTTTGA AAAACCTCTT GTGAATCTTC TTTTCGACTA TAAACGATGT
Machilus_yunnanensis AAAGACCTCT TCTGGTTTGA AAAACCTCTT GTGAATCTTC TTTTCGACTA TAAACGATGT
Machilus_thunbergii AAAGACCTCT TCTGGTTTGA AAAACCTCTT GTGAATCTTC TTTTCGACTA TAAACGATGT
Persea_americana AAAGACCTCT TCTGGTTTGA AAAACCTCTT GTGAATCTTC TTTTCGACTA TAAACGATGT
Lindera_glauca AAAGACCTCT TCTGGTTTGA AAAACCTCTT GTGAATCTTC TTTTCGACTA TAAACGATGT
Lindera_sericea AAAGACCTCT TCTGGTTTGA AAAACCTCTT GTGAATCTTC TTTTCGACTA TAAACGATGT
Lindera_megaphylla AAAGACCTCT TCTGGTTTGA AAAACCTCTT GTGAATCTTC TTTTCGACTA TAAACGATGT

....|....| ....|....| ....|....| ....|....| ....|....| ....|....|
1025 1035 1045 1055 1065 1075
Machilus_pauhoi AATCGTCCAT TGAGATATAT AAAAAAAAAT TTCTTTCAAA ATGCTGTAAG AAATGAAATG
Machilus_balansae AATCGTCCAT TGAGATATAT AAAAAAAAAT TTCTTTCAAA ATGCTGTAAG AAATGAAATG
Machilus_yunnanensis AATCGTCCAT TGAGATATAT AAAAAAAAAT TTCTTTCAAA ATGCTGTAAG AAATGAAATG
Machilus_thunbergii AATCGTCCAT TGAGATATAT AAAAAAAAAT TTCTTTCAAA ATGCTGTAAG AAATGAAATG
Persea_americana AATCGTCCAT TGAGATATAT AAAAAAAAAT TTATTTAAAA ATGCTGTAAG AAATGAAATG
Lindera_glauca AATCGTCCAT TGAGATATAT AAAAAAAAAT TTCTTTCAAA ATGCTGTAAG AAATGAAATG
Lindera_sericea AATCGTCCAT TGAGATATAT AAAAAAAAAT GTCTTTCAAA ATGCTGTAAG AAATGAAATG
Lindera_megaphylla AATCGTCCAT TGAGATATAT AAAAAAAAAT GTCTTTCAAA ATGCTGTAAG AAATGAAATG

....|....| ....|....| ....|....| ....|....| ....|....| ....|....|
1085 1095 1105 1115 1125 1135
Machilus_pauhoi TCACAATATT TTTTTCACGT ATGTCCAGTT GATGGAAAAC AAATAATATC TTTTACATAT
Machilus_balansae TCACAATATT TTTTTCACGT ATGTCCAGTT GATGGAAAAC AAATAATATC TTTTACATAT
Machilus_yunnanensis TCACAATATT TTTTTCACGT ATGTCCAGTT GATGGAAAAC AAATAATATC TTTTACATAT
Machilus_thunbergii TCACAATATT TTTTTCACGT ATGTCCAGTT GATGGAAAAC AAATAATATC TTTTACATAT
Persea_americana TCACAATATT TTTTTCACGT ATGTCCAGTT GATGGAAAAC AAATAATATC TTTTACATAT
Lindera_glauca TCACAATATT TTTTTCACGT ATGTCCAGTT GATGGAAAAC AAATAATATC TTTTACATAT
Lindera_sericea TCACAATATT TTTTTCACGT ATGTCCAGTT GATGGAAAAC AAATAATATC TTTTACATAT
Lindera_megaphylla TCACAATATT TTTTTCACGT ATGTCCAGTT GATGGAAAAC AAATAATATC TTTTACATAT

....|....| ....|....| ....|....| ....|....| ....|....| ....|....|
1145 1155 1165 1175 1185 1195
Machilus_pauhoi CCACCCAGTT TATCGATTTT TTTGGAAATG ATGCAAAGAA AGATGTCTTT GTGTACGACC
Machilus_balansae CCACCCAGTT TATCGATTTT TTTGGAAATG ATGCAAAGAA AGATGTCTTT GTGTACGACC
Machilus_yunnanensis CCACCCAGTT TATCGATTTT TTTGGAAATG ATGCAAAGAA AGATGTCTTT GTGTACGACC
Machilus_thunbergii CCACCCAGTT TATCGATTTT TTTGGAAATG ATGCAAAGAA AGATGTCTTT GTGTACGACC

Persea_americana CCACCCAGTT TATCGATTTT TTTGGAAATG ATGCAAAGAA AGATGTCTTT GTGTACGACC

Lindera_glauca CCACCCAGTT TATCGATTTT TTTGGAAATG ATGCAAAGAA AGATGTCTTT GTGTACGACC

Lindera_sericea CCACCCAGTT TATCGATTTT TTTGGAAATG ATGCAAAGAA AGATGTCTTT GTGTACGACC

Lindera_megaphylla CCACCCAGTT TATCGATTTT TTTGGAAATG ATGCAAAGAA AGATGTCTTT GTGTACGACC

....|....| ....|....| ....|....| ....|....| ....|....| ....|....|

1205 1215 1225 1235 1245 1255

Machilus_pauhoi GAAAAACTAT CCCCCGAAGA TCTGTATAAT CATTGGGTTT ATACCAATGA ACAAAAAAGG

Machilus_balansae GAAAAACTAT CCCCCGAAGA TCTGTATAAT CATTGGGTTT ATACCAATGA ACAAAAAAGG

Machilus_yunnanensis GAAAAACTAT CCCCCGAAGA TCTGTATAAT CATTGGGTTT ATACCAATGA ACAAAAAAGG

Machilus_thunbergii GAAAAACTAT CCCCCGAAGA TCTGTATAAT CATTGGGTTT ATACCAATGA ACAAAAAAGG

Persea_americana GAAAAACTAT CCCCCGAAGA TCTGTATAAT CATTGGGTTT ATACCAATGA ACAAAAAAGG

Lindera_glauca GAAAAACTAT CCCCCGAAGA TCTGTATAAT CATTGGGTTT ATACCAATGA ACAAAAAAGG

Lindera_sericea GAAAAACTAT CCCCCGAAGA TCTGTATAAT CATTGGGTTT ATACCAATGA ACAAAAAAGG

Lindera_megaphylla GAAAAACTAT CCCCCGAAGA TCTGTATAAT CATTGGGTTT ATACCAATGA ACAAAAAAGG

....|....| ....|....| ....|....| ....|....| ....|....| ....|....|

1265 1275 1285 1295 1305 1315

Machilus_pauhoi TACAGCTTGA GCAATGAATT CATAAACCGA ATAGAAGTTC TAAACAAGGG ATCTCTTACT

Machilus_balansae TACAGCTTGA GCAATGAATT CATAAACCGA ATAGAAGTTC TAAACAAGGG ATCTCTTACT

Machilus_yunnanensis TACAGCTTGA GCAATGAATT CATAAACCGA ATAGAAGTTC TAAACAAGGG ATCTCTTACT

Machilus_thunbergii TACAGCTTGA GCAATGAATT CATAAACCGA ATAGAAGTTC TAAACAAGGG ATCTCTTACT

Persea_americana TACAGCTTGA GCAATGAATT AATAAACCGA ATAGAAGTTC TAAACAAGGG ATCTCTTACT

Lindera_glauca TACAGCTTGA GCAATGAATT CATAAACCGA ATAGAAGTTC TAAACAAGGG ATCTCTTACT

Lindera_sericea TACAGCTTGA GCAATGAATT CATAAACCGA ATAGAAGTTC TAAACAAGGG ATCTCTTACT

Lindera_megaphylla TACAGCTTGA GCAATGAATT CATAAACCGA ATAGAAGTTC TAAACAAGGG ATCTCTTACT

....|....| ....|....| ....|....| ....|....| ....|....| ....|....|

1325 1335 1345 1355 1365 1375

Machilus_pauhoi ATGGATGTGC TTGAAAAAAG GACCAGATTG TATAATGATA AAAATAACCA AGAAGATAA-

Machilus_balansae ATGGATGTGC TTGAAAAAAG GACCAGATTG TATAATGATA AAAATAACCA AGAAGATAA-

Machilus_yunnanensis ATGGATGTGC TTGAAAAAAG GACCAGATTG TATAATGATA AAAATAACCA AGAAGATAA-

Machilus_thunbergii ATGGATGTGC TTGAAAAAAG GACCAGATTG TATAATGATA AAAATAACCA AGAAGATAA-

Persea_americana ATGGATGTGC TTGAAAAAAG GACCAGATTT TATAATGATA AAAATAACCA AGAAGATAA-

Lindera_glauca ATGGATGTGC TTGAAAAAAG GACCAGATTG TATAATGATA AAAATAACCA AGAAGATAAA

Lindera_sericea ATGGATGTGC TTGAAAAAAG GACCAGATTG TATAATGATA AAAATAACCA AGAAGATAAA

Lindera_megaphylla ATGGATGTGC TTGAAAAAAG GACCAGATTG TATAATGATA AAAATAACCA AGAAGATAA-

....|....| ....|....| ....|....| ....|....| ....|....| ....|....|

1385 1395 1405 1415 1425 1435

Machilus_pauhoi -----GAATA ACCAAGAAGA TAAGAATAAC CAAGAATGCT TGCCTAGAGT GTATGATCCT

Machilus_balansae -----GAATA ACCAAGAAGA TAAGAATAAC CAAGAATGCT TGCCTAGAGT GTATGATCCT

Machilus_yunnanensis -----GAATA ACCAAGAAGA TAAGAATAAC CAAGAATGCT TGCCTAGAGT GTATGATCCT

Machilus_thunbergii -----GAATA ACCAAGAAGA TAAGAATAAC CAAGAATGCT TGCCTAGAGT GTATGATCCT

Persea_americana -----GAATA ACCAAGAAGA TAAGAATAAC CAAGAATGCT TGCCTAGAGT GTATGATCCT

Lindera_glauca GATAAGAATA ACCAAGAAGA TAAGAATAAC CAAGAATGCT TGCCTAGAGT GTATGATCCT

Lindera_sericea ATTAAGAATA ACCAAGAAGA TAAGAATAAC CAAGAATGCT TGCCTAGAGT GTATGATCCT

Lindera_megaphylla -----GAATA ACCAAGAAGA TAAGAATAAC CAAGAATGCT TGCCTAGAGT GTATGATCCT

....|....| ....|....| ....|....| ....|....| ....|....| ....|....|

1445 1455 1465 1475 1485 1495

Machilus_pauhoi TTTTTAAACG GACCCTATCG TGGAACAATC AAAAAAGCGT ATTCACGTTC AATGGTGGAT

Machilus_balansae TTTTTAAACG GACCCTATCG TGGAACAATC AAAAAAGTGT ATTCACGTTC AATGGTGGAT

Machilus_yunnanensis TTTTTAAACG GACCCTATCG TGGAACAATC AAAAAAGCGT ATTCACGTTC AATGGTGGAT

Machilus_thunbergii TTTTTAAACG GACCCTATCG TGGAACAATC AAAAAAGCGT ATTCACGTTC AATGGTGGAT

Persea_americana TTTTTAAACG GACCATACCG TGGAACAATC AAAAAAGTGT ATTCACGTTC AATGGTGGAT

Lindera_glauca TTTTTAAACG GACCATATCG TGGAACAATC AAAAAAGTGT ATTCACGTTC AATGGTGGAT

Lindera_sericea TTTTTAAACG GACCATATCG TGGAACAATC AAAAAAGTGT ATTCACGTTC AATGGTGGAT

Lindera_megaphylla TTTTTAAACG GACCATATCG TGGAACAATC AAAAAAGTGT ATTCACGTTC AATGGTGGAT

....|....| ....|....| ....|....| ....|....| ....|....| ....|....|

1505 1515 1525 1535 1545 1555

Machilus_pauhoi GACTCAATCA CGTCGACAGA AGATTCCATA GGAATGGTTT GGATAAATAA GATTCATGAT

Machilus_balansae GACTCAATCA CGTCGACAGA AGATTCCATA GGAATGGTTT GGATAAATAA GATTCATGAT

Machilus_yunnanensis GACTCAATCA CGTCGACAGA AGATTCCATA GGAATGGTTT GGATAAATAA GATTCATGAT

Machilus_thunbergii GACTCAATCA CGTCGACAGA AGATTCCATA GGAATGGTTT GGATAAATAA GATTCATGAT

Persea_americana GACTCAATCA CTTCGACAGA AGATTCTATA GGAATGGTTT GGATAAATAA GATTCATGAT

Lindera_glauca GACTCAATCA CTTCGACAGA AGATTCTATA GGAATGGTTT GGATAAATAA GATTCATGAT

Lindera_sericea AACTCAATCA CTTCGACAGA AGATTCTATA GGAATGGTTT GGATAAATAA GATTCATGAT

Lindera_megaphylla GACTCAATCA CTTCGACAGA AGATTCTATA GGAATGGTTT GGATAAATAA GATTCATGAT

....|....| ....|....| ....|....| ....|....| ....|....| ....|....|

1565 1575 1585 1595 1605 1615

Machilus_pauhoi ATGCTTCCTA CTGATTACCA AAAACTTGAA CATAAAATGG ATATATTTAA TGGAGAATCA

Machilus_balansae ATGCTTCCTA CTGATTACCA AAAACTTGAA CATAAAATGG ATATATTTAA TGGAGAATCA

Machilus_yunnanensis ATGCTTCCTA CTGATTACCA AAAACTTGAA CATAAAATGG ATATATTTAA TGGAGAATCA

Machilus_thunbergii ATGCTTCCTA CTGATTACCA AAAACTTGAA CATAAAATGG ATATATTTAA TGGAGAATCA

Persea_americana ATGCTTCCTA CTGATTACCA AAAACTTGAA CATAAAATGG ATATATTTAA TGGAGAATCA

Lindera_glauca AGGCTTCCTA CTGATTACCA AAAACTTGAA CATAAAATGG ATACATTTAA TGGAGAATCA

Lindera_sericea AGGCTTCCTA CTGATTACCA AAAACTTGAA CATAAAATGG ATACATTTAA TGGAGAATCA

Lindera_megaphylla AGGCTTCCTA CTGATTACCA AAAACTTGAA CATAAAATGG ATACATTTAA TGGAGAATCA

....|....| ....|....| ....|....| ....|....| ....|....| ....|....|

1625 1635 1645 1655 1665 1675

Machilus_pauhoi TTATCGACAG ACATTGGTCC TTTCTTGACC TCTATCAGTG AATTAGCTAG GAAATCAACA

Machilus_balansae TTATCGACAG ACATTGGTCC TTTCTTGACC TCTATCAGTG AATTAGCTAG GAAATCAACA

Machilus_yunnanensis TTATCGACAG ACATTGGTCC TTTCTTGACC TCTATCAGTG AATTAGCTAG GAAATCAACA

Machilus_thunbergii TTATCGACAG ACATTGGTCC TTTCTTGACC TCTATCAGTG AATTAGCTAG GAAATCAACA

Persea_americana TTATCGACAG ACATTGGTCC TTTCTTGACC TCTATCAGTG AATTAGCTAG GAAATCAACA

Lindera_glauca TTATCGACAG ACATTGGTCC TTTCTTGACC TCTATCAGTG AATTAGCTAG GAAATCAACA

Lindera_sericea TTATCGACAG ACATTGGTCC TTTCTTGACC TCTATCAGTG AATTAGCTAG GAAATCAACA

Lindera_megaphylla TTATCGACAG ACATTGGTCC TTTCTTGACC TCTATCAGTG AATTAGCTAG GAAATCAACA

....|....| ....|....| ....|....| ....|....| ....|....| ....|....|

1685 1695 1705 1715 1725 1735

Machilus_pauhoi ACTGGTTTTA ATCTTAATTT GAAAAAGCTT GTTTTAATAT CCGAACAAAG AAGATTTGAT

Machilus_balansae ACTGGTTTTA ATCTTAATTT GAAAAAGCTT GTTTTAATAT CCGAACAAAG AAGATTTGAT

Machilus_yunnanensis ACTGGTTTTA ATCTTAATTT GAAAAAGCTT GTTTTAATAT CCGAACAAAG AAGATTTGAT

Machilus_thunbergii ACTGGTTTTA ATCTTAATTT GAAAAAGCTT GTTTTAATAT CCGAACAAAG AAGATTTGAT

Persea_americana ACTGGTTTTA GTCTGAATTT TAAAAAGCTT GTTTTAATAT CCGAACAAAG AAGATTTGAT

Lindera_glauca ACCGGTTTTA GTCTGAATTT TAAAAAGCTT GTTTTAATAT CCGAACAAAG AAGATTTGAT

Lindera_sericea ACTGGTTTTA GTCTTAATTT TAAAAAGCTT GTTTTAATAT CCGAACAAAG AAGATTTGAT

Lindera_megaphylla ACTGGTTTTA GTCTGAATTT TAAAAAGCTT GTTTTAATAT CCGAACAAAG AAGATTTGAT

....|....| ....|....| ....|....| ....|....| ....|....| ....|....|

1745 1755 1765 1775 1785 1795

Machilus_pauhoi TCAGAAAATA AAACAAAATG TTTGAAATTT CTATTCGATG TAATTACAAC TGATCCAAAT

Machilus_balansae TCAGAAAATA AAACAAAATG TTTGAAATTT CTATTCGATG TAATTACAAC TGATCCAAAT

Machilus_yunnanensis TCAGAAAATA AAACAAAATG TTTGAAATTT CTATTCGATG TAATTACAAC TGATCCAAAT

Machilus_thunbergii TCAGAAAATA AAAAAAAATG TTTGAAATTT CTATTCGATG TAATTACAAC TGATCCAAAT

Persea_americana TCAGAAAATA AAACAAAATG TTTGAAATTT CTATTCGATG TAATTACAAC TGATCCAAAT

Lindera_glauca TCAGAAAATA AAACAAAATG TTTGAAATTT CTATTCGATG TAATTACAAC TGATCCAAAT

Lindera_sericea TCAGAAAATA AAACAAAATG TTTGAATTTT CTATTCGATG TAATTACAAC TGATCCAAAT

Lindera_megaphylla TCAGAAAATA AAACAAAATG TTTGAAATTT CTATTCGATG TAATTACAAC TGATCCAAAT

....|....| ....|....| ....|....| ....|....| ....|....| ....|....|

1805 1815 1825 1835 1845 1855

Machilus_pauhoi AATCAAACAA TTCAAAAAAA ATCTATTGGA ATAGAAGAAA TCGGTAAAAA GATTCCTCGA

Machilus_balansae AATCAAACAA TTCAAAAAAA ATCTATTGGA ATAGAAGAAA TCGGTAAAAA GATTCCTCGA

Machilus_yunnanensis AATCAAACAA TTCAAAAAAA ATCTATTGGA ATAGAAGAAA TCGGTAAAAA GATTCCTCGA

Machilus_thunbergii AATCAAACAA TTCAAAAAAA ATCTATTGGA ATAGAAGAAA TCGGTAAAAA GATTCCTCGA

Persea_americana AATCAAACAA TTCAAAATAA ATCTATTGGA ATAGAAGAAA TCGGTAAAAA GATTCCTCGA

Lindera_glauca AATCAAACAA TTCAAAATAA ATCTATTGGA ATAGAAGAAA TCGGTAAAAA GATTCCCCGA

Lindera_sericea AATCAAACAA TTCAAAATAA ATCTATTGGA ATAGAAGAAA TCGGTAAAAA GATTCCTCGA

Lindera_megaphylla AATCAAACAA TTCAAAATAA ATCTATTGGA ATAGAAGAAA TCGGTAAAAA GATTCCTCGA

....|....| ....|....| ....|....| ....|....| ....|....| ....|....|

1865 1875 1885 1895 1905 1915

Machilus_pauhoi CGATCATACA AATTGATCGA TTCTTTTGAA GAGCGGGAGG AGGAAAATGA GGAAGAATCA

Machilus_balansae CGATCATACA AATTGATCGA TTCTTTTGAA GAGCGGGAGG AGGAAAATGA GGAAGAATCA

Machilus_yunnanensis CGATCATACA AATTGATCGA TTCTTTTGAA GAGCGGGAGG AGGAAAATGA GGAAGAATCA

Machilus_thunbergii CGATCATACA AATTGATCGA TTCTTTTGAA GAGCGGGAGG AGGAAAATGA GGAAGAATCA

Persea_americana CGATCATACA AATTGATCGA TTCTTTTGAA GAGCGGGAGG AGGAAAATGA GGAAGAATCA

Lindera_glauca CGATCATACA AATTGATCAA TTCTTTTGAA GAGCGGGAGG AGGAAAATGA GGAAGAATCA

Lindera_sericea CGATCATACA AATTGATCAA TTCTTTTGAA GAGCGGGAGG AGGAAAATGA GGAAGAATCA

Lindera_megaphylla CGATCATACA AATTGATCAA TTCTTTTGAA GAGCGGGAGG AGGAAAATGA GGAAGAATCA

....|....| ....|....| ....|....| ....|....| ....|....| ....|....|

1925 1935 1945 1955 1965 1975

Machilus_pauhoi ACAGAAAATC ATGGGATTCG TTCAAGAAAA GCCAAACGTG TGGTAATTTA TACTGATAAG

Machilus_balansae ACAGAAAATC ATGGGATTCG TTCAAGAAAA GCCAAACGTG TGGTAATTTA TACTGATAAG

Machilus_yunnanensis ACAGAAAATC ATGGGATTCG TTCAAGAAAA GCCAAACGTG TGGTAATTTA TACTGATAAG

Machilus_thunbergii ACAGAAAATC ATGGGATTCG TTCAAGAAAA GCCAAACGTG TGGTAATTTA TACTGATAAG

Persea_americana ACAGAAAATC ATGGGATTCG TTCAAGAAAA GCCAAACGTG TGGTAATTTA TACTGATAAG

Lindera_glauca ACAGAAAATC ATGGGATTCG TTCAAGAAAA GCCAAACGTG TGGTAATTTA TACTGATAAG

Lindera_sericea ACAGAAAATC ATGGGATTCG TTCAAGAAAA GCCAAACGTG TGGTAATTTA TACTGATAAG

Lindera_megaphylla ACAGAAAATC ATGGGATTCG TTCAAGAAAA GCCAAACGTG TGGTAATTTA TACTGATAAG

....|....| ....|....| ....|....| ....|....| ....|....| ....|....|

1985 1995 2005 2015 2025 2035

Machilus_pauhoi GCGGATCCGG ATCAGAATAC CAATACTCAT ACTAGTACCA GTACTAATAG TGATCAAGCA

Machilus_balansae GCGGATCCGG ATCAGAATAC CAATACTCAT ACTAGTACCA GTACTAATAG TGATCAAGCA

Machilus_yunnanensis GCGGATCCGG ATCAGAATAC CAATACTCAT ACTAGTACCA GTACTAATAG TGATCAAGCA

Machilus_thunbergii GCGGATCCGG ATCAGAATAC CAATACTCAT ACTAGTACCA GTACTAATAG TGATCAAGCA

Persea_americana GCGGATCCGG ATCAGAATAC CAATACTCAT ACTAGTACCA GTACTAATAG TGATCAAGCA

Lindera_glauca GCGGATCCGG ATCAGAATAC CAATACTGAT ACTAGTACCA GTACTAATAG TGATCAAGCA

Lindera_sericea GCGGATCCGG ATCAGAATAC CAATACTGAT ACTAGTACCA GTACTAATAG TGATCAAGCA

Lindera_megaphylla GCGGATCCGG ATCAGAATAC CAATACTGAT ACTAGTACCA GTACTAATAG TGATCAAGCA

....|....| ....|....| ....|....| ....|....| ....|....| ....|....|

2045 2055 2065 2075 2085 2095

Machilus_pauhoi GAAGAGTTGG CTTTGATACG TTACTCGCAA CAATCAGATT TTCGTCGGGA TATAGTAAAA

Machilus_balansae GAAGAGTTGG CTTTGATACG TTACTCGCAA CAATCAGATT TTCGTCGGGA TATAGTAAAA

Machilus_yunnanensis GAAGAGTTGG CTTTGATACG TTACTCGCAA CAATCAGATT TTCGTCGGGA TATAGTAAAA

Machilus_thunbergii GAAGAGTTGG CTTTGATACG TTACTCGCAA CAATCAGATT TTCGTCGGGA TATAGTAAAA

Persea_americana GAAGAGTTGG CTTTGATACG TTACTCGCAA CAATCAGATT TTCGTCGGGA TATAGTAAAA

Lindera_glauca GAAGAGTTGG CTTTGGTACG TTACTCGCAA CAATCTGATT TTCGTCGGGA TATAGTAAAA

Lindera_sericea GAAGAGTTGG CTTTGGTACG TTACTCGCAA CAATCAGATT TTCGTCGGGA TATAGTAAAA

Lindera_megaphylla GAAGAGTTGG CTTTGGTACG TTACTCGCAA CAATCAGATT TTCGTCGGGA TATAGTAAAA

....|....| ....|....| ....|....| ....|....| ....|....| ....|....|

2105 2115 2125 2135 2145 2155

Machilus_pauhoi GGATCCATGC GCGCTCAAAG ACGTAAAATA GTTACTTGGG AAATGTTTCA AGCGAATGTG

Machilus_balansae GGATCCATGC GCGCTCAAAG ACGTAAAATA GTTACTTGGG AAATGTTTCA AGCGAATGTG

Machilus_yunnanensis GGATCCATGC GCGCTCAAAG ACGTAAAATA GTTACTTGGG AAATGTTTCA AGCGAATGTG

Machilus_thunbergii GGATCCATGC GCGCTCAAAG ACGTAAAATA GTTACTTGGG AAATGTTTCA AGCGAATGTG

Persea_americana GGATCCATGC GCGCTCAAAG ACGTAAAATA GTTACTTGGG AAATGTTTCA AGCGAATGTG

Lindera_glauca GGATCCATGC GCGCTCAAAG ACGTAAAATA GTTATTTGGG AAATGTTTCA AGCGAATGTG

Lindera_sericea GGATCCATGC GCGCTCAAAG ACGTAAAATA GTTACTTGGG AAATGTTTCA AGCGAATGTG

Lindera_megaphylla GGATCCATGC GCGCTCAAAG ACGTAAAATA GTTATTTGGG AAATGTTTCA AGCGAATGTG

....|....| ....|....| ....|....| ....|....| ....|....| ....|....|

2165 2175 2185 2195 2205 2215

Machilus_pauhoi CATTCCCTGC TTTTTTTGGA CAGAATAGAC AAAACTTTTT TTTTTTCTTT TGATATCTCC

Machilus_balansae CATTCCCTGC TTTTTTTGGA CAGAATAGAC AAAACTTTTT TTTTTTCTTT TGATATCTCC

Machilus_yunnanensis CATTCCCTGC TTTTTTTGGA CAGAATAGAC AAAACTTTTT TTTTTTCTTT TGATATCTCC

Machilus_thunbergii CATTCCCTGC TTTTTTTGGA CAGAATAGAC AAAACTTTTT TTTTTTCTTT TGATATCTCC

Persea_americana CATTCCCTGC TTTTTTTGGA CAGAATAGAC AAAACTTTTT TTTTTTCTTT TGATATCTCC

Lindera_glauca CATTCCCTGC TTTTTTTGGA CAGAATAGAC AAAACTTTTT TTTTTTCTTT TGATATCTCC

Lindera_sericea CATTCCCTGC TTTTTTTGGA CAGAATAGAC AAAACTTTTT TTTTTTCTTT TGATATCTCC

Lindera_megaphylla CATTCCCTGC TTTTTTTGGA CAGAATAGAC AAAACTTTTT TTTTTTCTTT TGATATCTCC

....|....| ....|....| ....|....| ....|....| ....|....| ....|....|

2225 2235 2245 2255 2265 2275

Machilus_pauhoi CGAACAATGA ATCTCATTTT TAGAAATTGG ATAGATACAG GACCGAAATT CAAAACTTCG

Machilus_balansae CGAACAATGA ATCTCATTTT TAGAAATTGG ATAGATACAG GACCGAAATT CAAAACTTCG

Machilus_yunnanensis CGAACAATGA ATCTCATTTT TAGAAATTGG ATAGATACAG GACCGAAATT CAAAACTTCG

Machilus_thunbergii CGAACAATGA ATCTCATTTT TAGAAATTGG ATAGATACAG GACCGAAATT CAAAACTTCG

Persea_americana CGAACAATGA ATCTAATTTT TAGAAATTGG ATAGATACAG GACCGAAATT AAAAACTTCG

Lindera_glauca CGAACAATGA ATCTAATTTT TAGAAATTGG ATAGATACAG GACCGAAATT CAAAACTTCG

Lindera_sericea CGAACAATGA ATCTCATTTT TAGAAATTGG ATAGATACAG GACCGAAATT CAAAACTTCG

Lindera_megaphylla CGAACAATGA ATCTAATTTT TAGAAATTGG ATAGATACAG GACCGAAATT CAAAACTTCG

....|....| ....|....| ....|....| ....|....| ....|....| ....|....|

2285 2295 2305 2315 2325 2335

Machilus_pauhoi GATTCTGAGG AGGAAGAGGC AAAAGAAAAG GCAAAAAAAA TTGCAGATAA AAAAAACGAG

Machilus_balansae GATTCTGAGG AGGAAGAGGC AAAAGAAAAG GCAAAAAAAA TTGCAGATAA AAAAAACGAG

Machilus_yunnanensis GATTCTGAGG AGGAAGAGGC AAAAGAAAAG GCAAAAAAAA TTGCAGATAA AAAAAACGAG

Machilus_thunbergii GATTCTGAGG AGGAAGAGGC AAAAGAAAAG GCAAAAAAAA TTGCAGATAA AAAAAACGAG

Persea_americana GATTCTGAGG AGCAAGAGGC AAAAGAAAAG GCAAAAAAAA TTGCAGATAA AAAAAACGAG

Lindera_glauca GATTCTGAGG AGGAAGAGGC AAAAGAAAAG ACAAAAAAAA TTGCAGATAA AAAAAACGAG

Lindera_sericea GATTCTGAGG AGGAAGAGGC AAAAGAAAAG GCAAAAAAAA TTGCGGATAA AAAAAACGAG

Lindera_megaphylla GATTCTGAGG AGGAAGAGGC AAAAGAAAAG GCAAAAAAAA TTGCAGATAA AAAAAACGAG

....|....| ....|....| ....|....| ....|....| ....|....| ....|....|

2345 2355 2365 2375 2385 2395

Machilus_pauhoi AATGAAAGGA TAGCAATAGC AGAAACTTGG GATACTATTA TATTTGCTCA AGCAATAAGA

Machilus_balansae AATGAAGGGA TAGCATTAGC AGAAACTTGG GATACTATTA TATTTGCTCA AGCAATAAGA

Machilus_yunnanensis AATGAAAGGA TAGCAATAGC AGAAACTTGG GATACTATTA TATTTGCTCA AGCAATAAGA

Machilus_thunbergii AATGAAAGGA TAGCAATAGC AGAAACTTGG GATACTATTA TATTTGCTCA AGCAATAAGA

Persea_americana	AATGAAAGGA	TAGCAATAGC	AGAAACTTGG	GATACTATTA	TATTTGCTCA	AGCAATAAGA
Lindera_glauca	AATGAAAGGA	TAGCAATAGC	AGAAACTTGG	GATACTATTA	TATTTGCTCA	AGCAATAAGA
Lindera_sericea	AATGAAAGGA	TAGCAATAGC	AGAAACTTGG	GATACTATTA	TATTTGCTCA	AGCAATAAGA
Lindera_megaphylla	AATGAGAGGA	TAGCAATAGC	AGAAACTTGG	GATACTATTA	TATTTGCTCA	AGCAATAAGA

	....|....|	....|....|	....|....|	....|....|	....|....|	....|....|
	2405	2415	2425	2435	2445	2455
Machilus_pauhoi	GGGACTATGT	TAGTAACCCA	ATCAATTCTT	AGAAAATACA	TCATATTGCC	TTCATTGATA
Machilus_balansae	GGTACTATGT	TAGTAACCCA	ATCAATTCTT	AGAAAATACA	TCATATTGCC	TTCATTGATA
Machilus_yunnanensis	GGGACTATGT	TAGTAACCCA	ATCAATTCTT	AGAAAATACA	TCATATTGCC	TTCATTGATA
Machilus_thunbergii	GGGACTATGT	TAGTAACCCA	ATCAATTCTT	AGAAAATACA	TCATATTGCC	TTCATTGATA
Persea_americana	GGTACTATGT	TAGTAACCCA	ATCGATTCTT	AGAAAATACA	TCATATTGCC	TTCATTGATA
Lindera_glauca	GGTACTATGT	TAGTAACCCA	ATCGATTCTT	AGAAAATACA	TCATATTGCC	TTCATTGATA
Lindera_sericea	GGTACTATGT	TAGTAACCCA	ATCGATTCTT	AGAAAATACG	TCATATTGCC	TTCATTGATA
Lindera_megaphylla	GGTACTATGT	TAGTAACCCA	ATCGATTCTT	AGAAAATACA	TCATATTGCC	TTCATTGATA

	....|....|	....|....|	....|....|	....|....|	....|....|	....|....|
	2465	2475	2485	2495	2505	2515
Machilus_pauhoi	ATAGCTAAAA	ACCTCGGCCG	TATGCTCTTA	TTTCAATTCC	CCGAGTGGTA	CGAGGATTTG
Machilus_balansae	ATAGCTAAAA	ACCTCGGCCG	TATGCTCTTA	TTTCAATTCC	CCGAGTGGTA	CGAGGATTTG
Machilus_yunnanensis	ATAGCTAAAA	ACCTCGGCCG	TATGCTCTTA	TTTCAATTCC	CCGAGTGGTA	CGAGGATTTG
Machilus_thunbergii	ATAGCTAAAA	ACCTCGGCCG	TATGCTCTTA	TTTCAATTCC	CCGAGTGGTA	CGAGGATTTG
Persea_americana	ATAGCTAAAA	ACCTCGGCCG	TATGTTGTTA	TTTCAATTCC	CCGAGTGGTA	CGAGGATTTG
Lindera_glauca	ATAGCTAAAA	ACCTAGGCCG	TATGTTTTTA	TTTCAATTCC	CCGAGTGGTA	CGAGGATTTT
Lindera_sericea	ATAGCTAAAA	ACCTCGGCCG	TATGTTCTTA	TTTCAATTCC	CCGAGTGGTA	CGAGGATTTG
Lindera_megaphylla	ATAGCTAAAA	ACCTCGGCCG	TATGTTCTTA	TTTCAATTCC	CCGAGTGGTA	CGAGGATTTG

	....|....|	....|....|	....|....|	....|....|	....|....|	....|....|
	2525	2535	2545	2555	2565	2575
Machilus_pauhoi	AAGGAGTGGA	ATAGAGAAAT	GCATGTTAAA	TGCACCTATA	ATGGTGTTCA	ATTATCAGAA
Machilus_balansae	AAGGAGTGGA	ATAGAGAAAT	GCATGTTAAA	TGCACCTATA	ATGGTGTTCA	ATTATCAGAA
Machilus_yunnanensis	AAGGAGTGGA	ATAGAGAAAT	GCATGTTAAA	TGCACCTATA	ATGGTGTTCA	ATTATCAGAA
Machilus_thunbergii	AAGGAGTGGA	ATAGAGAAAT	GCATGTTAAA	TGCACCTATA	ATGGTGTTCA	ATTATCAGAA
Persea_americana	AAGGAGTGGA	ATAGAGAAAT	GCATGTTAAA	TGCACCTATA	ATGGTGTTCA	ATTATCAGAA
Lindera_glauca	AAGGAGTGGA	ATAGAGAAAT	GCATGTTAAA	TGCACCTATA	ATGGTGTTCA	ATTATCAGAA
Lindera_sericea	AAGGAGTGGA	ATAGAGAAAT	GCATGTTAAA	TGCACCTATA	ATGGTGTTCA	ATTATCAGAA
Lindera_megaphylla	AAGGAGTGGA	ATAGAGAAAT	GCATGTTAAA	TGCACCTATA	ATGGTGTTCA	ATTATCAGAA

	....|....|	....|....|	....|....|	....|....|	....|....|	....|....|
	2585	2595	2605	2615	2625	2635
Machilus_pauhoi	ACAGAATTTC	CGAAAAACTG	GTTAACAGAT	GGTATTCAGA	TAAAAATCCT	ATTTCCTTTC
Machilus_balansae	ACAGAATTTC	CGAAAAACTG	GTTAACAGAT	GGTATTCAGA	TAAAAATCCT	ATTTCCTTTC
Machilus_yunnanensis	ACAGAATTTC	CGAAAAACTG	GTTAACAGAT	GGTATTCAGA	TAAAAATCCT	ATTTCCTTTC
Machilus_thunbergii	ACAGAATTTC	CGAAAAACTG	GTTAACAGAT	GGTATTCAGA	TAAAAATCCT	ATTTCCTTTC

Persea_americana ACAGAATTTC CGAAAAACTG GTTAACAGAT GGTATTCAGA TAAAAATCCT ATTTCCTTTC

Lindera_glauca ACAGAATTTC CGAAAAACTG GTTAACAGAT GGTATTCAGA TAAAAATCCT ATTTCCTTTC

Lindera_sericea ACAGAATTTC CGAAAAACTG GTTAACAGAT GGTATTCAGA TAAAAATCCT ATTTCCTTTC

Lindera_megaphylla ACAGAATTTC CGAAAAACTG GTTAACAGAT GGTATTCAGA TAAAAATCCT ATTTCCTTTC

....|....| ....|....| ....|....| ....|....| ....|....| ....|....|

2645 2655 2665 2675 2685 2695

Machilus_pauhoi TGTCTGAAAC CCTGGCGCAA ATCCAAACTA CGATCCCATC ATAGAGATCC AATCCAAAAG

Machilus_balansae TGTCTGAAAC CCTGGCGCAA ATCCAAACTA CGATCCCATC ATAGAGATCC AATCCAAAAG

Machilus_yunnanensis TGTCTGAAAC CCTGGCGCAA ATCCAAACTA CGATCCCATC ATAGAGATCC AATCCAAAAG

Machilus_thunbergii TGTCTGAAAC CCTGGCGCAA ATCCAAACTA CGATCCCATC ATAGAGATCC AATCCAAAAG

Persea_americana TGTCTGAAAC CCTGGCGCAA ATCCAAACTA CGATCCCATC ATAGAGATCC AATCCAAAAG

Lindera_glauca TGTCTGAAAC CCTGGCGCAA ATCCAAACTA CGATCCCATC ATAGAGATCT AATCCAAAAG

Lindera_sericea TGTCTGAAAC CCTGGCGCAA ATCCAAACTA CGATCCCATC ATAGAGATCC AATCCAAAAG

Lindera_megaphylla TGTCTGAAAC CCTGGCGCAA ATCCAAACTA CGATCCCATC ATAGAGATCC AATCCAAAAG

....|....| ....|....| ....|....| ....|....| ....|....| ....|....|

2705 2715 2725 2735 2745 2755

Machilus_pauhoi AAGGGGAAAA CGGAAAATTT TTGTTTTTTA ACAATCTGGG GAAGGGAAAC CGAACTACCT

Machilus_balansae AAGGGGAAAA CGGAAAATTT TTGTTTTTTA ACAATCTGGG GAAGGGAAAC CGAACTACCT

Machilus_yunnanensis AAGGGGAAAA CGGAAAATTT TTGTTTTTTA ACAATCTGGG GAAGGGAAAC CGAACTACCT

Machilus_thunbergii AAGGGGAAAA CGGAAAATTT TTGTTTTTTA ACAATCTGGG GAAGGGAAAC CGAACTACCT

Persea_americana AAAGGGAAAA CGGAAAATTT TTGTTTTTTA ACAATCTGGG GAAGGGAAAC CGAACTACCT

Lindera_glauca AAAGGGAAAA CAGAAAATTT TTGTTTTTTA ACAATCTGGG GAAGGGAAAC CGAACTACCT

Lindera_sericea AAAGGGAAAA CAGAAAATTT TTGTTTTTTA ACAATCTGGG GAAGGGAAAC CGAACTACCT

Lindera_megaphylla AAAGGGAAAA CAGAAAATTT TTGTTTTTTA ACAATCTGGG GAAGGGAAAC CGAACTACCT

....|....| ....|....| ....|....| ....|....| ....|....| ....|....|

2765 2775 2785 2795 2805 2815

Machilus_pauhoi TTTGGTTCTG CCCGACAACA ACCTTCCTTT TTTGAACCTA TTTATAATGA ATTCGAAAAA

Machilus_balansae TTTGGTTCTG CCCGACAACA ACCTTCCTTT TTTGAACCTA TTTATAATGA ATTCGAAAAA

Machilus_yunnanensis TTTGGTTCTG CCCGACAACA ACCTTCCTTT TTTGAACCTA TTTATAATGA ATTCGAAAAA

Machilus_thunbergii TTTGGTTCTG CCCGACAACA ACCTTCCTTT TTTGAACCTA TTTATAATGA ATTCGAAAAA

Persea_americana TTTGGTTCTG CCCGACAACA ACCTTCCTTT TTTGAACCTA TTTATAATGA ATTCGAAAAA

Lindera_glauca TTTGGTTCTG CCCGACAACA ACCTTCCTTT TTTGAACCTA TTTATAATGA ATTCAAAAAA

Lindera_sericea TTTGGTTCTG CCCGACAACA ACCTTCCTTT TTTGAACCTA TTTATAATGA ATTCGAAAAA

Lindera_megaphylla TTTGGTTCTG CCCGACAACA ACCTTCCTTT TTTGAACCTA TTTATAATGA ATTCGAAAAA

....|....| ....|....| ....|....| ....|....| ....|....| ....|....|

2825 2835 2845 2855 2865 2875

Machilus_pauhoi AAAAAGAGAA AAGTGAAAAA AAAATGTTTT CTAGTTCTAA GAGTTTTCAA AAAAAAAACA

Machilus_balansae AAAAAAAGAA AAGTGAAAAA AAAATGTTTT CTAGTTCTAA GAGTTTTCAA AAAAAAAACA

Machilus_yunnanensis AAAAAGAGAA AAGTGAAAAA AAAATGTTTT CTAGTTCTAA GAGTTTTCAA AAAAAAAACA

Machilus_thunbergii AAAAAGAGAA AAGTGAAAAA AAAATGTTTT CTAGTTCTAA GAGTTTTCAA AAAAAAAACA

```
Persea_americana        AAAAAGAGAA AAGTGAAAAA AAAATGTTTT CTAGTTCTAA GAGTTTTCAA AAAAAAAACA
Lindera_glauca          AAAAAGAGAA AAGTGAAAAA AAAATGTTTT CTAGTTCTAA GAGTTTTCAA AGAAAAAACA
Lindera_sericea         AAAAAGAGAA AAGTGAAAAA AAAAAGTTTT CTAGTTCTAA GAGTTTTCAA AGAAAAAACA
Lindera_megaphylla      AAAAAGAGAA AAGTGAAAAA AAAATGTTTT CTAGTTCTAA GAGTTTTCAA AGAAAAAACA

                        ....|....| ....|....| ....|....| ....|....| ....|....| ....|....|
                            2885       2895       2905       2915       2925       2935
Machilus_pauhoi         AAACAGTTTA TAAAAGTCTC AAAAGAAAAA ACAAGATGGA TTATCAAAAC GGTTCTATTT
Machilus_balansae       AAACAGTTTA TAAAAGTCTC AAAAGAAAAA ACAAGATGGA TTATCAAAAC GCTTCTATTT
Machilus_yunnanensis    AAACAGTTTA TAAAAGTCTC AAAAGAAAAA ACAAGATGGA TTATCAAAAC GGTTCTATTT
Machilus_thunbergii     AAACAGTTTA TAAAAGTCTC AAAAGAAAAA ACAAGATGGA TTATCAAAAC GGTTCTATTT
Persea_americana        AAACAGTTTA TAAAGGTCTC AAAAGAAAAA ACAAGATGGA TTATCAAAAC GGTTCTATTT
Lindera_glauca          AAACAGTTTA TAAAAGTCTC AAAAGAAAAA ACAAGATGGA TTATCAAAAT GGTTCTATTT
Lindera_sericea         AAACAGTTTA TAAAGGTCTC AAAAGAAAAA ACAAGATGGA TTATCAAAAC GGTTCTATTT
Lindera_megaphylla      AAACGGTTTA TAAAGGTCTC AAAAGAAAAA ACAAGATGGA TTATCAAAAC GGTTCTATTT

                        ....|....| ....|....| ....|....| ....|....| ....|....| ....|....|
                            2945       2955       2965       2975       2985       2995
Machilus_pauhoi         TTAAAAAGAA TAATAAAAGA GTTTGTAAAC GTAAATACAA TTTTCTTATT TGTATTGAAG
Machilus_balansae       TTAAAAAGAA TAATAAAAGA GTTTGTAAAC GTAAATACAA TTTTCTTATT TGTATTGAAG
Machilus_yunnanensis    TTAAAAAGAA TAATAAAAGA GTTTGTAAAC GTAAATACAA TTTTCTTATT TGTATTGAAG
Machilus_thunbergii     TTAAAAAGAA TAATAAAAGA GTTTGTAAAC GTAAATACAA TTTTCTTATT TGTATTGAAG
Persea_americana        TTAAAAAGAA TAATAAAAGA GTTTGTAAAC GTAAATACAA TTTTCTTATT TGTATTGAAG
Lindera_glauca          TTGAAAAGAA TAATAAAAAA GTTTGCAAAC GTAAATCCAA TTTTCTTATT TGTATTGAAG
Lindera_sericea         TTAAAAAGAA TAATAAAAGA GTTTGCAAAC GTAAATCCAA TTTTCTTATT TGTATTGAAG
Lindera_megaphylla      TTAAAAAGAA TAATAAAAGA GTTTGCAAAC GTAAATCCAA TTTTCTTATT TGTATTGAAG

                        ....|....| ....|....| ....|....| ....|....| ....|....| ....|....|
                            3005       3015       3025       3035       3045       3055
Machilus_pauhoi         AAAGTATATG AACCGAATGG AAATGGAAAA GATTCCATAA TCAGAAGCAG TAATAAAATT
Machilus_balansae       AAAGTATATG AACCGAATGG AAATGGAAAA GATTCCATAA TCAGAAGCAG TAATAAAATT
Machilus_yunnanensis    AAAGTATATG AACCGAATGG AAATGGAAAA GATTCCATAA TCAGAAGCAG TAATAAAATT
Machilus_thunbergii     AAAGTATATG AACCGAATGG AAATGGAAAA GATTCCATAA TCAGAAGCAG TAATAAAATT
Persea_americana        AAAGTATATG AACCGAATGA AAATGGAAAA GATTCCATAA TCATAAGCAG TAATAAAATT
Lindera_glauca          AAAGTATATG AACCAAATGA AAATGGAAAA GATTCCATAA TCATAAGCAG TAATAAAATT
Lindera_sericea         AAAGTATATG AACCGAATGA AAATGGAAAA GATTCCATAA TCATAAGCAG TAATAAAACT
Lindera_megaphylla      AAAGTATATG AACCGAATGA AAATGGAAAA GATTCCATAA TTATAAGCAG TACTAAAACT

                        ....|....| ....|....| ....|....| ....|....| ....|....| ....|....|
                            3065       3075       3085       3095       3105       3115
Machilus_pauhoi         GTTCCTGAAT CGACCATTCG AATTAGATTC ATGGATTGGG CAAATTATTC ACTGACAGAA
Machilus_balansae       GTTCCTGAAT CGACCATTCG AATTAGATTC ATGGATTGGG CAAATTATTC ACTGACAGAA
Machilus_yunnanensis    GTTCCTGAAT CGACCATTCG AATTAGATTC ATGGATTGGG CAAATTATTC ACTGACAGAA
Machilus_thunbergii     GTTCCTGAAT CGACCATTCG AATTAGATTC ATGGATTGGG CAAATTATTC ACTGACAGAA
```

Persea_americana GTTCCTGAAT CGACCATTCG AATTAGATTC ATGGATTGGT CAAATTATTC ACTGACAGAA

Lindera_glauca GTTCCTGAAT CGACCATTCG AATTAGATTC ATGGATTGGG CAAATTATTC ACTGACAGAA

Lindera_sericea GTTCCTGAAT CGACCATTCG AATTAGATTC ATGGATTGGG CAAATTATTC ACTGACAGAA

Lindera_megaphylla GTTCCTGAAT CGACCATTCG AATTAGATTC ATGGATTGGG CAAATTATTC ACTGACAGAA

....|....| ....|....| ....|....| ....|....| ....|....| ....|....|

3125 3135 3145 3155 3165 3175

Machilus_pauhoi AAAAAAAGGA AAGATCTGTC CGATAGAACA ACCCTAATCA GAAATCAAAT AGAAAGGGGT

Machilus_balansae AAAAAAAGGA AAGATCTGTC CGATAGAACA ACCCTAATCA GAAATCAAAT AGAAAGGGGT

Machilus_yunnanensis AAAAAAAGGA AAGATCTGTC CGATAGAACA ACCCTAATCA GAAATCAAAT AGAAAGGGGT

Machilus_thunbergii AAAAAAAGGA AAGATCTGTC CGATAGAACA ACCCTAATCA GAAATCAAAT AGAAAGGGGT

Persea_americana AAAAAAAGGA AAGATCTGTC CGATAGAACA ACCCTAATCA GAAATCAAAT AGAAAGGGGT

Lindera_glauca AAAAAAAGGA AAGATCTGTC CGATAGAACA ACCCTAATCA GAAATCAAAT AGAAAGGGGT

Lindera_sericea AAAAAAAGGA AAGATCTGTC TGATAGAACA ACCCTAATCA GAAATCAAAT AGAAAGGGGT

Lindera_megaphylla AAAAAAAGGA AAGATCTGTC CGATAGAACA ACCCTAATCA GAAATCAAAT AGAAAGGGGT

....|....| ....|....| ....|....| ....|....| ....|....| ....|....|

3185 3195 3205 3215 3225 3235

Machilus_pauhoi GCAAAAGACA AAAGAAAAAT ATTTCTAACT CCGGATATAA ATATTAGTCC TAACGATACA

Machilus_balansae GCAAAAGACA AAAGAAAAAT ATTTCTAACT CCGGATATAA ATATGAGTCC TAACGATACA

Machilus_yunnanensis GCAAAAGACA AAAGAAAAAT ATTTCTAACT CCGGATATAA ATATTAGTCC TAACGATACA

Machilus_thunbergii GCAAAAGACA AAAGAAAAAT ATTTCTAACT CCGGATATAA ATATTAGTCC TAACGATACA

Persea_americana GCGAAAGACA AAAGAAAAAT ATTTCTAACT CCGGATATAA ATATTAGTCC TAACGATACA

Lindera_glauca GCAAAAGACA AAAGAAAAAT ATTTCTAACT CCGGATATAA ATATTAGTCC TAACGATACA

Lindera_sericea GCAAAAGACA AAAGAAAAAT ATTTCTAACT CCGGATATAA ATATTAGTCC TAACGATACA

Lindera_megaphylla GCAAAAGACA AAAGAAAAAT ATTTCTAACT CCGGATATAA ATATTAGTCC TAACGATACA

....|....| ....|....| ....|....| ....|....| ....|....| ....|....|

3245 3255 3265 3275 3285 3295

Machilus_pauhoi AGTTGTGGTG ATAAAAGATC GGAATCGCAG AAACCTATTT GGCAGATATC AAAAAGAAGA

Machilus_balansae AGTTGTGGTG ATAAAAGATC GGAATCGCAG AAACCTATTT GGCAGATATC AAAAAGAAGA

Machilus_yunnanensis AGTTGTGGTG ATAAAAGATC GGAATCGCAG AAACCTATTT GGCAGATATC AAAAAGAAGA

Machilus_thunbergii AGTTGTGGTG ATAAAAGATC GGAATCGCAG AAACCTATTT GGCAGATATC AAAAAGAAGA

Persea_americana AGTTGTGGTG ATAAAAGATC GGAATCGCAG AAACATATTT GGCAGATATC AAAAAGAAGA

Lindera_glauca AGTTGTGGTG ATAAAAGATC GGAATCGCAG AAACATATTT GGCAGATATC AAAAAGAAGA

Lindera_sericea AGTTGTGGTG ATAAAAGATC GGAATCGCAG AAACATATTT GGCAGATATC AAAACGAAGA

Lindera_megaphylla AGTTGTGGTG ATAAAAGATC GGAATCGCAG AAACATATTT GGCAGATATC AAAAAGAAGA

....|....| ....|....| ....|....| ....|....| ....|....| ....|....|

3305 3315 3325 3335 3345 3355

Machilus_pauhoi AGTAACAGAT TCATATTCAT ACGCAAATGG CACTATTTTT TGACATTTTT CAACGAAAGA

Machilus_balansae AGTAACAGAT TCATATTCAT ACGCAAATGG CACTATTTTT TGACATTTTT CAACGAAAGA

Machilus_yunnanensis AGTAACAGAT TCATATTCAT ACGCAAATGG CACTATTTTT TGACATTTTT CAACGAAAGA

Machilus_thunbergii AGTAACAGAT TCATATTCAT ACGCAAATGG CACTATTTTT TGACATTTTT CAACGAAAGA

```
Persea_americana        AGTAACAGAT TCATATTCAT ACACAAATGG CACTATTTTT TGACATTTTT CAACGAAAGA
Lindera_glauca          AGTAACAGAT TCATATTCAT ACGCAAATGG CACTATTTTT TGACATTTTT CAACGAAAGA
Lindera_sericea         AGTAACAGAT TCATATTCAT ACGCAAATGG CACTATTTTT TGACATTTTT CAACGAAAGA
Lindera_megaphylla      AGTAACAGAT TCATATTCAT ACGCAAATGG CACTATTTTT TGACATTTTT CAACGAAAGA

                        ....|....| ....|....| ....|....| ....|....| ....|....| ....|....|
                             3365       3375       3385       3395       3405       3415
Machilus_pauhoi         ATATACATAC ATATCTTTCT ATGTACGGTT AATATTTCTA GAGTCAATGT ACAACTTTTC
Machilus_balansae       ATATACATAC ATATCTTTCT ATGTACGGTT AATATTTCTA GAGTCAATGT ACAACTTTTC
Machilus_yunnanensis    ATATACATAC ATATCTTTCT ATGTACGGTT AATATTTCTA GAGTCAATGT ACAACTTTTC
Machilus_thunbergii     ATATACATAC ATATCTTTCT ATGTACGGTT AATATTTCTA GAGTCAATGT ACAACTTTTC
Persea_americana        ATATACATAC ATATCTTTCT ATGTACTGTT AATATTTCTA GAGTCAATGT ACAACTTTTC
Lindera_glauca          ATATACATAC ATATCTTTCT ATGTACTGTT AATGTTTCTA GAGTCAATGT ACAACTTTTC
Lindera_sericea         ATATACATAC ATATCTTTCT ATGTACTGTT AATGTTTCTA GAGTCAATGT ACAACTTTTC
Lindera_megaphylla      ATATACATAC ATATCTTTCT ATGTACTGTT AATGTTTCTA GAGTCAATGT ACAACTTTTT

                        ....|....| ....|....| ....|....| ....|....| ....|....| ....|....|
                             3425       3435       3445       3455       3465       3475
Machilus_pauhoi         CTTGAATCAA CAAAAAAGAT TATCGATAAA TACATTCACA ATGATGAAAA AAATAAAGAA
Machilus_balansae       CTTGAATCAA CAAAAAAGAT TATCGATAAA TACATTCACA ATGATGAAAA AAATAAAGAA
Machilus_yunnanensis    CTTGAATCAA CAAAAAAGAT TATCGATAAA TACATTCACA ATGATGAAAA AAATAAAGAA
Machilus_thunbergii     CTTGAATCAA CAAAAAAGAT TATCGATAAA TACATTCACA ATGATGAAAA AAATAAAGAA
Persea_americana        CTTGAATCAA CAAAAAAGAT TATCGATAAA TACATTCACA ATGATGAAAA AAATAAAGAA
Lindera_glauca          CTTGAATCAA CAAAAAAGAT TATCGATAAA TACATTCACA ATGATGAAAA AAATAAAGAA
Lindera_sericea         CTTGAATCAA CAAAAAAGAT TATCGATAAA TACATTCACA ATGATGAAAA AAATAAAGAA
Lindera_megaphylla      CTTGAATCAA CAAAAAAGAT TATCGATAAA TACATTCACA ATGATGAAAA AAATAAAGAA

                        ....|....| ....|....| ....|....| ....|....| ....|....| ....|....|
                             3485       3495       3505       3515       3525       3535
Machilus_pauhoi         GGGATTGATG AAACAAATCA AAAAAAAATT CAATTTATTT CGACTATAAA AAAGTCTCTT
Machilus_balansae       GGGATTGATG AAACAAATAA AAAAAAAATT CACTTTATTT CGACTATAAA AAAGTCTCTT
Machilus_yunnanensis    GGGATTGATG AAACAAATCA AAAAAAAATT CAATTTATTT CGACTATAAA AAAGTCTCTT
Machilus_thunbergii     GGGATTGATG AAACAAATCA AAAAAAAATT CAATTTATTT CGACTATAAA AAAGTCTCTT
Persea_americana        GGGATTGATG AAACAAATCA AAAAAAAATT CACTTTATTT CGACTATAAA AAAGTCTCTT
Lindera_glauca          GGGATTGATG AAACAAATCA AAAAAAAATT CACTTTATTT CGACTATAAA AAAGCCTCTT
Lindera_sericea         GGGATTGATG AAACAAATCA AAAAAAAGTG CACTTTATTT CGACTATAAA AAAGTCTCTT
Lindera_megaphylla      GGGATTGATG AAACAAATCA AAAAAAAATT CACTTTATTT CGACTATAAA AAAGTCTCTT

                        ....|....| ....|....| ....|....| ....|....| ....|....| ....|....|
                             3545       3555       3565       3575       3585       3595
Machilus_pauhoi         TCTAATATTA GTAATAATAA ATCAAAAATT TCTGGTGACC CATATTCCTT TTCACAAGCA
Machilus_balansae       TCTAATATTA GTAATAATAA ATCAAAGATT TCTGGTGACC CATATTCCTT TTCACAAGCA
Machilus_yunnanensis    TCTAATATTA GTAATAATAA ATCAAAAATT TCTGGTGACC CATATTCCTT TTCACAAGCA
Machilus_thunbergii     TCTAATATTA GTAATAATAA ATCAAAAATT TCTGGTGACC CATATTCCTT TTCACAAGCA
```

Persea_americana TCTAATATTA GTAATAATAA ATCAAAGATT TCTGGTGACC TATATTCCTT TTCACAAGCA

Lindera_glauca TTTAATATTA GTAATAATAA ATCAAAGATT TCTGGTGACC TATATCCCTT TTCACAAGCA

Lindera_sericea TCTGATATTC GTAATAATAA ATCAAAGATT TCTGGTGACC TATATTCCTT TTCACAAGCA

Lindera_megaphylla TCTAATATTA GTAATAATAA ATCAAAGATT TCTGGTGACC TATATTCCTT TTCACAAGCA

....|....| ....|....| ....|....| ....|....| ....|....| ....|....|

3605 3615 3625 3635 3645 3655

Machilus_pauhoi TCTGTATTTT ACAAATTATC ACAAATCGAA GCTATCAA-- -GAAGTATCA TTTGAGATCT

Machilus_balansae TCTGTATTTT ACAAATTATC ACAAATCCAA GCTATCAA-- -GAAGTATCA TTTGAGATCT

Machilus_yunnanensis TCTGTATTTT ACAAATTATC ACAAATCGAA GCTATCAA-- -GAAGTATCA TTTGAGATCT

Machilus_thunbergii TCTGTATTTT ACAAATTATC ACAAATCGAA GCTATCAA-- -GAAGTATCA TTTGAGATCT

Persea_americana TCTGTATTTT ACAAATTATC ACAAATCCAA GCTATTAA-- -GAAGTATCA TTTGAGATCT

Lindera_glauca TCTGTATTTT ACAAATTATC ACAAATCCAA GCTATTAATA AGAAGTATCA TTTGAGATCT

Lindera_sericea TCTGTATTTT ACAAATTATC ACAAATCCAA GCTATTAATA AGAAGTATCA TTTGAGATCT

Lindera_megaphylla TCTGTATTTT ACAAATTATC ACAAATCCAA GCTATTAATA AGAAGTATCA TTTGAGATCT

....|....| ....|....| ....|....| ....|....| ....|....| ....|....|

3665 3675 3685 3695 3705 3715

Machilus_pauhoi CTACTTCAAT ATCGCGAAGC ATATCTTATT CTTAAGGATA GAATCAGGAA TTTTTTTGGA

Machilus_balansae CTACTTCAAT ATCGCGAAGC ATATCTTATT CTTAAGGATA GAATCAGGAA TTTTTTTGGA

Machilus_yunnanensis CTACTTCAAT ATCGCGAAGC ATATCTTATT CTTAAGGATA GAATCAGGAA TTTTTTTGGA

Machilus_thunbergii CTACTTCAAT ATCGCGAAGC ATATCTTATT CTTAAGGATA GAATCAGGAA TTTTTTTGGA

Persea_americana CTACTTCAAT ATCGCGAAGC ATATCTTATT CTTAAGGATA GAATCAGGAA TTTTTTTGGA

Lindera_glauca CTACTTCAAT ATCGCGAAGC ATATCTTATT CTTAAGGATA GAATCAGGAA TTTTTTTGGA

Lindera_sericea CTACTTCAAT ATCGCGAAGC ATATCTTATT CTTAAGGATA GAATCAGGAA TTTTTTTGGA

Lindera_megaphylla CTACTTCAAT ATCGCGAAGC ATATCTTATT CTTAAGGATA GAATCAGGAA TTTTTTTGGA

....|....| ....|....| ....|....| ....|....| ....|....| ....|....|

3725 3735 3745 3755 3765 3775

Machilus_pauhoi ACACGAAGAA TATTTGATTC CAAATCAAGG CATAAAAAAC TTCCGAATTC TGGAATGAAT

Machilus_balansae ACACGAAGAA TATTTGATTC CAAATCAAGG CATAAAAAAC TTCCGAATTC TGGAATGAAT

Machilus_yunnanensis ACACGAAGAA TATTTGATTC CAAATCAAGG CATAAAAAAC TTCCGAATTC TGGAATGAAT

Machilus_thunbergii ACACGAAGAA TATTTGATTC CAAATCAAGG CATAAAAAAC TTCCGAATTC TGGAATGAAT

Persea_americana ACACGAAGAA TATTTGATTC CAAATCAAGG CATAAAAAAC TTCCGAATTC TGGAATGAAT

Lindera_glauca ACACGAAGAA TATTAGATTC CAAATCAAGG CATAAAAAAC TTCCAAATTC CGGAATGAAT

Lindera_sericea ACACGAAGAA TATTAGATTC CAAATCAAGG CATAAAAAAT TTCCGAATTC TGGAATGAAT

Lindera_megaphylla ACACGAAGAA TATTAGATTC CAAATCAAGG CATAAAAAAC TTCCGAATTC TGGAATGAAT

....|....| ....|....| ....|....| ....|....| ....|....| ....|....|

3785 3795 3805 3815 3825 3835

Machilus_pauhoi GAATGGAAAA ACTGGTTAAG GGGTCATTAT CAATACAATT TATCTCAGGC TAGGTGGTCT

Machilus_balansae GAATGGAAAA ACTGGTTAAG GGGTCATTAT CAATACAATT TATCTCAGGC TAGGTGGTCT

Machilus_yunnanensis GAATGGAAAA ACTGGTTAAG GGGTCATTAT CAATACAATT TATCTCAGGC TAGGTGGTCT

Machilus_thunbergii GAATGGAAAA ACTGGTTAAG GGGTCATTAT CAATACAATT TATCTCAGGC TAGGTGGTCT

Persea_americana	GAATGGAAAA ACTGGTTAAG GGGTCATTAT CAATACAATT TATCTCAGGC TAGGTGGTCT
Lindera_glauca	GAATGGAAAA ACTGGTTAAG GGGTCATTAT CAATACAATT TATCTCAGGC TAGGTGGTCT
Lindera_sericea	GAATGGAAAA ACTGGTTAAG GGGTCATTAT CAATACAATT TATCTCAGGC TAGGTGGTCT
Lindera_megaphylla	GAATGGAAAA ACTGGTTAAG GGGTCATTAT CAATACAATT TATCTCAGGC TAGGTGGTCT

	....|....| ....|....| ....|....| ....|....| ....|....| ....|....|
	3845 3855 3865 3875 3885 3895
Machilus_pauhoi	AAATTAGTAC CGCAAAAATG GCGAACTAGG GTCAATTGGC GTCGTACGAT TCAAAATAAA
Machilus_balansae	AAATTAGTAC CGCAAAAATG GCGAACTAGG GTCAATTGGC GTCGTACGAT TCAAAATAAA
Machilus_yunnanensis	AAATTAGTAC CTCAAAAATG GCGAACTAGG GTCAATTGGC GTCGTACGAT TCAAAATAAA
Machilus_thunbergii	AAATTAGTAC CGCAAAAATG GCGAACTAGG GTCAATTGGC GTCGTACGAT TCAAAATAAA
Persea_americana	AAATTAGTAC CGCAAAAATG GCGAACTAGG GTCAATCGGC GTCGTACGAT TCAAAATAAA
Lindera_glauca	AAATTAGTAC CGCAAAAATG GCGAACTAGG GTCAATCGGC GTCGTACGAT TCAAAATAAA
Lindera_sericea	AAATTAGTAC CGCAAAAATG GCGAACTAGG GTCAATCGGC GTCGTACGAT TCAAAATAAA
Lindera_megaphylla	AAATTAGTAC CGCAAAAATG GCGAACTAGG GTCAATCGGC GTCGTACGAT TCAAAATAAA

	....|....| ....|....| ....|....| ....|....| ....|....| ....|....|
	3905 3915 3925 3935 3945 3955
Machilus_pauhoi	GACTCAAAAA AGAATTCATA TGAAAAAGCC CAATTCATTC ATTACGAGAA AAAAAATGAT
Machilus_balansae	GACTCAAAAA AGAATTCATA TGAAAAAGCC CAATTCATTC ATTACGAGAA AAAAAATGAT
Machilus_yunnanensis	GACTCAAAAA AGAATTCATA TGAAAAAGCC CAATTCATTC ATTACGAGAA AAAAAATGAT
Machilus_thunbergii	GACTCAAAAA AGAATTCATA TGAAAAAGCC CAATTCATTC ATTACGAGAA AAAAAATGAT
Persea_americana	GACTCAAAAA AGAATTCATA TGAAAAAGCC CAATTCATTC ATTACGAGAA AAAAAATGAT
Lindera_glauca	GACTCAAAAA AGAATTCATA TGAAAAAGCC CAATTCATTC ATTACGAGAA AAAAAATGAT
Lindera_sericea	GACTCAAAAA AGAATTCATA TGAAAAAGCC CAATTCATTC ATTACGAGAA AAAAAATGAT
Lindera_megaphylla	GACTCAAAAA AGAATTCATA TGAAAAAGCC CAATTCATTC ATTACGAGAA AAAAAATGAT

	....|....| ....|....| ....|....| ....|....| ....|....| ....|....|
	3965 3975 3985 3995 4005 4015
Machilus_pauhoi	TATGAAGTGA ATTCATTGAC GAGCAAAAAA GCAAAATTAA AAAAAAACTA CAGATATGAT
Machilus_balansae	TATGAAGTGA ATTCATTGAC GAGCAAAAAA GCAAAATTAA AAAAAAACTA CAGATATGAT
Machilus_yunnanensis	TATGAAGTGA ATTCATTGAC GAGCAAAAAA GCAAAATTAA AAAAAAACTA CAGATATGAT
Machilus_thunbergii	TATGAAGTGA ATTCATTGAC GAGCAAAAAA GCAAAATTAA AAAAAAACTA CAGATATGAT
Persea_americana	TATGAAGTGA ATTCATTGAC GAGCAAAAAA GAAAAATTAA AAAAAAACTA CAGATATGAT
Lindera_glauca	TATGAAGTGA ATTCATTGAC GAACAAAAAA GCAAAATTAA AAAAAAACTA CAGATATGAT
Lindera_sericea	TATGAAGTGA ATTCATTGAC GAGCAAAAAA GAAAAATTAA AAAAAAACTA CAGATATGAT
Lindera_megaphylla	TATGAAGTGA ATTCATTGAC GAGCAAAAAA GAAAAATTAA AAAAAAACTA CAGATATGAT

	....|....| ....|....| ....|....| ....|....| ....|....| ....|....|
	4025 4035 4045 4055 4065 4075
Machilus_pauhoi	CTTTTTTCAT ATAAATATAT TAATTATGGG GATAGGAAAG ACTCATATAT TTATCCATCC
Machilus_balansae	CTTTTTTCAT ATAAATATAT TAATTATGGG GATAGGAAAG ACTCATATAT TTATCCATCC
Machilus_yunnanensis	CTTTTTTCAT ATAAATATAT TAATTATGGG GATAGGAAAG ACTCATATAT TTATCCATCC
Machilus_thunbergii	CTTTTTTCAT ATAAATATAT TAATTATGGG GATAGGAAAG ACTCATATAT TTATCCATCC

Persea_americana CTTTTTTCAT ATAAATATAT TAATTATGGG GATAGGAAAG ACTCCTATAT TTATTCATCC
Lindera_glauca CTTTTTTCAT ATAAATATAT TAATTATGGG GATAGGAAAG ACTCATATAT TTATCCATCC
Lindera_sericea CTTTTTTCAT ATAAATATAT TCATTATGGG GATAGGAAAG ACTCATATAT TTATCCATCC
Lindera_megaphylla CTTTTTTCAT ATAAATATAT TAATTATGGG GATAGGAAAG ACTCATATAT TTATCCATCC

....|....| ....|....| ....|....| ....|....| ....|....| ....|....|
4085 4095 4105 4115 4125 4135

Machilus_pauhoi TCATTACAAG TAAACGAGGA CCGAGAGATT CCATATAACT ACAACACACC TAAAATTGAA
Machilus_balansae TCATTACAAG TAAACGAGGA CCGAGAGATT CCATATAATT ACAACACACC TAAAATTGAA
Machilus_yunnanensis TCATTACAAG TAAACGAGGA CCGAGAGATT CCATATAACT ACAACACACC TAAAATTGAA
Machilus_thunbergii TCATTACAAG TAAACGAGGA CCGAGAGATT CCATATAACT ACAACACACC TAAAATTGAA
Persea_americana TCATTACAAG TAAACGAGGA CCGAGAGATT CCATATAATT ACAACACACC TAAAATTGAA
Lindera_glauca TCATTACAAG TAAACGAGGA CCGAGAGATT CCATATAATT ACAACACACC TAAAATTGAA
Lindera_sericea TCATTACAAG TAAACGAGGA CCGAGAGATT CCATATAATT ACAACACACC TAAAATTGAA
Lindera_megaphylla TCATTACAAG TAAACGAGGA CCGAGAGATT CCATATAATT ACAACACACC TAAAATTGAA

....|....| ....|....| ....|....| ....|....| ....|....| ....|....|
4145 4155 4165 4175 4185 4195

Machilus_pauhoi CCATTTTATG TACTGGGGGA TATATCTATT AGTGATTATC TAGGAGAAGA GTATATTATT
Machilus_balansae CCATTTTATG TACTGGGGGA TATATCTATT CGTGATTATC TAGGAGAAGA GTATATTATT
Machilus_yunnanensis CCATTTTATG TACTGGGGGA TATATCTATT AGTGATTATC TAGGAGAAGA GTATATTATT
Machilus_thunbergii CCATTTTATG TACTGGGGGA TATATCTATT AGTGATTATC TAGGAGAAGA GTATATTATT
Persea_americana CCATTTTATG TACTGGGGGA TATATCTATT AGTGATTATC TAGGAGAAGA GTATATTATT
Lindera_glauca CCATTTTATG TAGTGGGGGA TATATCTATT AGTGATTATC TAGGAGAAGA GTATATTATT
Lindera_sericea CCATTTTATG TACTGGGGGA TATATCTATT AGTGATTATC TAGGAGAAGA GTATATTATT
Lindera_megaphylla CCATTTTATG TACTGGGGGA TATATCTATT GGTGATTATC TAGGAGAAGA GTATATTATT

....|....| ....|....| ....|....| ....|....| ....|....| ....|....|
4205 4215 4225 4235 4245 4255

Machilus_pauhoi GGTACGGGTA AAAGTACGGA TAGAAAATAT TTGGAGTGGA AAATTTTCGA TTTATTTCTT
Machilus_balansae GGTACGGGTA AAAGTACGGA TAGAAAATAT TTGGAGTGGA AAATTTTCGA TTTATTTCTT
Machilus_yunnanensis GGTACGGGTA AAAGTACGGA TAGAAAATAT TTGGAGTGGA AAATTTTCGA TTTATTTCTT
Machilus_thunbergii GGTACGGGTA AAAGTACGGA TAGAAAATAT TTGGAGTGGA AAATTTTCGA TTTATTTCTT
Persea_americana GGTACGGGTA AAAGTACGGA TAGAAAATAT TTGGAGTGGA AAATTTTCGA TTTATTTCTT
Lindera_glauca GGTACGGGTA AAAGTACGGA TAGAAAATAT TTGGAGTGGA AAATTTTTGA TTTATTTCTT
Lindera_sericea GGTACGGGTA AAAGTACGGA TAGAAAATAT TTGGAGTGGA AAATTTTCGA TTTATTTCTT
Lindera_megaphylla GGTACGGGTA AAAGTACGGA TAGAAAATAT TTGGAGTGGA AAATTTTCGA TTTATTTCTT

....|....| ....|....| ....|....| ....|....| ....|....| ....|....|
4265 4275 4285 4295 4305 4315

Machilus_pauhoi AGAAAGAATA TCGATATTGA GTCCTGGACC GATACGGATA CCGGGACCAA CATTAATAAA
Machilus_balansae AGAAAGAATA TCGATATTGA GTCCTGGACC GATACGGATA CCGGGACCAA CATTAATAAA
Machilus_yunnanensis AGAAAGAATA TCGATATTGA GTCCTGGACC GATACGGATA CCGGGACCAA CATTAATAAA
Machilus_thunbergii AGAAAGAATA TCGATATTGA GTCCTGGACC GATACGGATA CCGGGACCAA CATTAATAAA

Persea_americana AGAAAGAATA TCGATATTGA GTCCTGGACC GATACGGATA CCGGGACCAA CATTAATAAA

Lindera_glauca AGAAAGAATA TCGATATTGA GTCCTGTACC GATACGGATA CCGGGACCAA CATTAATAAA

Lindera_sericea AGAAAGAATA TCGATATTGA GTCCTGGCCC GATACGGATA CCGGGACCAA CATTAATAAA

Lindera_megaphylla AGAAAGAATA TCGATATTGA GTCCTGGACC GATACGGATA CCGGGACCAA CATTAATAAA

....|....| ....|....| ....|....| ....|....| ....|....| ....|....|

4325 4335 4345 4355 4365 4375

Machilus_pauhoi ATGACTAAGA CCGAGACTGA TTATTATCAA ATGATTGATA AGAAAGATCT TTTCTATCTC

Machilus_balansae ATGACTAAGA CCGAGACTTA TTATTATCAA ATGATTGATA AGAAAGATCT TTTCTATCTC

Machilus_yunnanensis ATGACTAAGA CCGAGACTGA TTATTATCAA ATGATTGATA AGAAAGATCT TTTCTATCTC

Machilus_thunbergii ATGACTAAGA CCGAGACTGA TTATTATCAA ATGATTGATA AGAAAGATCT TTTCTATCTC

Persea_americana ATGACTAAGA CCGAGACTGA TTATTATCAA ATGATTGATA AGAAAGATCT TTTCTATCTC

Lindera_glauca ATGACTAAGA CCGAGACTTA TTATTATCAA ATGATTGATA AGAAAGATCT TTTCTATCTC

Lindera_sericea ATGACTAAGA CCGAGACTGA TTATTATCAA ATGATTGATA AGAAAGATCT TTTCTATCTC

Lindera_megaphylla ATGACTAAGA CCGAGACTGA TTATTATCAA ATGATTGATA AGAAAGATCT TTTCTATCTC

....|....| ....|....| ....|....| ....|....| ....|....| ....|....|

4385 4395 4405 4415 4425 4435

Machilus_pauhoi ACGATTCATC AAGAAATCAA CCCACCCAAT CAAAAAAAAA AGTTTTTTTT GATGGGAATG

Machilus_balansae ACGATTCATC AAGAAATCAA CCCACCCAAT CAAAAAAAAA AGTTTTTTTT GATGGGAATG

Machilus_yunnanensis ACGATTCATC AAGAAATCAA CCCACCCAAT CAAAAAAAAA AGTTTTTTTT GATGGGAATG

Machilus_thunbergii ACGATTCATC AAGAAATCAA CCCACCCAAT CAAAAAAAAA AGTTTTTTTT GATGGGAATG

Persea_americana ACGATTCATC AAGAAATCAA CCCACCCAAT CAAAAAAAAA AGTTTTTTTT GATGGGAATG

Lindera_glauca ACGATTCATC AAGAAATCAA CCCACCCAAT CAAAAAAAAA ACTTTTTTTT GATGGGAATG

Lindera_sericea ACGATTCATC AAGAAATCAA CCCACCCAAT CAAAAAAAAA ACTTTTTTTT GATGGGAATG

Lindera_megaphylla ACGATTCATC AAGAAATCAA CCCACCCAAT CAAAAAAAAA ACTTTTTTTT GATGGGAATG

....|....| ....|....| ....|....| ....|....| ....|....| ....|....|

4445 4455 4465 4475 4485 4495

Machilus_pauhoi AATAAAGAAA TGCTATATCG TCCCATATTA AATCCGAAAT CTTGGTTCTT CTCAGAATTT

Machilus_balansae AATAAAGAAA TGCTATATCG TCCCATATTA AATCCGAAAT CTTGGTTCTT CTCAGAATTT

Machilus_yunnanensis AATAAAGAAA TGCTATATCG TCCCATATTA AATCCGAAAT CTTGGTTCTT CTCAGAATTT

Machilus_thunbergii AATAAAGAAA TGCTATATCG TCCCATATTA AATCCGAAAT CTTGGTTCTT CTCAGAATTT

Persea_americana AATAAAGAAA TGCTATATCG TCCCATATTA AATCCGAAAT CTTGGTTCTT CTCAGAATTT

Lindera_glauca AATAAAGAAA TGCTATATCG TCCCATATTA AATACGAAAT CTTGGTTCTT CTCAGAATTT

Lindera_sericea AATAAAGAAA TGCTATATCG TCCCATATTA AATACGAAAT CTTGGTTCTT CTCAGAATTT

Lindera_megaphylla AATAAAGAAA TGCTATATCG TCCCATATTA AATACGAAAT CTTGGTTCTT CTCAGAATTT

....|....| ....|....| ....|....| ....|....| ....|....| ....|....|

4505 4515 4525 4535 4545 4555

Machilus_pauhoi GTGCCACTTT ATGATGCATA TAAGATCAAA CCGTGGATTA TACCAATCAA ATTACTTCTT

Machilus_balansae GTGCCACTTT ATGATGCATA TAAGATCAAA CCGTGGATTA TACCAATCAA ATTACTTCTT

Machilus_yunnanensis GTGCCACTTT ATGATGCATA TAAGATCAAA CCGTGGATTA TACCAATCAA ATTACTTCTT

Machilus_thunbergii GTGCCACTTT ATGATGCATA TAAGATCAAA CCGTGGATTA TACCAATCAA ATTACTTCTT

```
Persea_americana        GTGCCACTTT ATGATGCATA TAAGATCAAA CCGTGGATTA TACCAATCAA ATTACTTCTT
Lindera_glauca          GTGCCACTTT ATGATGCATA TAAGATCAAA CCGTGGATTA TACCAATCAA ATTACTTCTT
Lindera_sericea         GTGCCACTTT ATGATGCATA TAAGATCAAA CCGTGGATTA TACCAATCAA ATTACTTCTT
Lindera_megaphylla      GTGCCACTTT ATGATGCATA TAAGATCAAA CCGTGGATTA TACCAATCAA ATTACTTCTT

                        ....|....| ....|....| ....|....| ....|....| ....|....| ....|....|
                             4565       4575       4585       4595       4605       4615
Machilus_pauhoi         TTCATTTTTA ATGGAAATGA AAACATTAGT GAAAACAAAA ACATTAATGG AAATCAAAAA
Machilus_balansae       TTCATTTTTA ATGGAAATGA AAACATTAGT GAAAACAAAA ACATTAATGG AAATCAAAAA
Machilus_yunnanensis    TTCATTTTTA ATGGAAATGA AAACATTAGT GAAAACAAAA ACATTAATGG AAATCAAAAA
Machilus_thunbergii     TTCATTTTTA ATGGAAATGA AAACATTAGT GAAAACAAAA ACATTAATGG AAATCAAAAA
Persea_americana        TTCATTTTTA ATGGAAATGA AAACATTAGT GAAAACAAAA ACATTAATGG AAATAAAAAA
Lindera_glauca          TTCATTTTTA ATGGAAATGA AAACATTA-- ---------- ------ATGG AAATCAAAAA
Lindera_sericea         TTCATTTTTA ATGGAAATGA AAACATTAGT GAAAACAAAA ACATTAATGG AAATCAAAAA
Lindera_megaphylla      TTCATTTTTA ATGGAAATGA AAACATTAGT GAAAACAAAA ACATTAATGG AAATCAAGAA

                        ....|....| ....|....| ....|....| ....|....| ....|....| ....|....|
                             4625       4635       4645       4655       4665       4675
Machilus_pauhoi         AAGGATCTTC GTATATCATC TAATCAAAAA GAATATCTTG AATTAAAGAA TCGAAATCAA
Machilus_balansae       AAGGATCTTC GTATATCATC TAATCAAAAA GAATATCTTG AATTAAAGAA TCGAAATCAA
Machilus_yunnanensis    AAGGATCTTC GTATATCATC TAATCAAAAA GAATATCTTG AATTAAAGAA TCGAAATCAA
Machilus_thunbergii     AAGGATCTTC GTATATCATC TAATCAAAAA GAATATCTTG AATTAAAGAA TCGAAATCAA
Persea_americana        AAGGATCTTC GTATATCATC TAATCAAAAA GAATATCTTG AATTAAAGAA TCGAAATCAA
Lindera_glauca          AAGGATCTTC GTATATCATC TAATCAAAAA GAATATCTTG AATTAAAGAA TCGAAATCAA
Lindera_sericea         AAGGATCTTC GTATATCATC TAATCAAAAA GAATATCTTG AATTAAAGAA TCGAAATCAA
Lindera_megaphylla      AAGGATCTTC GTATATCATC TAATCAAAAA GAATATCTTG AATTAAAGAA TCGAAATCAA

                        ....|....| ....|....| ....|....| ....|....| ....|....| ....|....|
                             4685       4695       4705       4715       4725       4735
Machilus_pauhoi         GAAGAAAAAG AACAGCTCGG CCACGGAAAT ATTGGCTCAG ACGCACGAAA ACGACAAAAA
Machilus_balansae       GAAGAAAAAG AACAGCTCGG CCACGGAAAT ATTGGCTCAG ACGCACGAAA ACGACAAAAA
Machilus_yunnanensis    GAAGAAAAAG AACAGCTCGG CCACGGAAAT ATTGGCTCAG ACGCACGAAA ACGACAAAAA
Machilus_thunbergii     GAAGAAAAAG AACAGCTCGG CCACGGAAAT ATTGGCTCAG ACGCACGAAA ACGACAAAAA
Persea_americana        GAAGAAAAAG AACAGCTCGG ACACGGAAAT ATTGGCTCAG ACGCACGAAA ACGACAAAAA
Lindera_glauca          GAAGAAAAAG AACAGCTCGG CCACGGAAAT ATTGGTTCAG ACGCACGAAA ACGACAAAAA
Lindera_sericea         GAAGAAAAAG AACAGCTCGG CCACGGAAAT ATTGGCTCAG ACGCACGAAA ACGACAAAAA
Lindera_megaphylla      GAAGAAAAAG AACAGCTCGG CCACGGAAAT ATTGGCTCAG ACGCACGAAA ACGACAAAAA

                        ....|....| ....|....| ....|....| ....|....| ....|....| ....|....|
                             4745       4755       4765       4775       4785       4795
Machilus_pauhoi         GATTTTGAAA AGGATTACAC GGAATCAGAC ATTCAAAAAC GTGAAAAGAA AGGACAACCC
Machilus_balansae       GATTTTGAAA AGGATTACAC GGAATCAGAC ATTCAAAAAC GTGAAAAGAA AGGACAACCC
Machilus_yunnanensis    GATTTTGAAA AGGATTACAC GGAATCAGAC ATTCAAAAAC GTGAAAAGAA AGGACAACCC
Machilus_thunbergii     GATTTTGAAA AGGATTACAC GGAATCAGAC ATTCAAAAAC GTGAAAAGAA AGGACAACCC
```

Persea_americana GATTTTGAAA AGGATTACAC GGAATCAGAC ATTCAAAAAC GTGAAAAGAA AGGACAACCC
Lindera_glauca TATTTGGAAA AGGATTACAT GGAATCAGAC ATTCAAAAAC GTGAAAAGAA AGGACAACCC
Lindera_sericea GATTTTGAAA AGGATTACAC GGAATCAGAC ATTCAAAAAC GTGAAAAGAA AGGACAACCC
Lindera_megaphylla GATTTTGAAA AGGATTACAC GGAATCAGAC ATTCAAAAAC GTGAAAAGAA AGGACAACCC

....|....| ....|....| ....|....| ....|....| ....|....| ....|....|
4805 4815 4825 4835 4845 4855
Machilus_pauhoi GAGAGTAACA AGAAAGCAAA ACTAGAGTTA TTCCTGAAAA AATATTTGCT TTTTCAATTG
Machilus_balansae GAGAGTAACA AGAAAGCAAA ACTAGAGTTA TTCCTGAAAA AATATTTGCT TTTTCAATTG
Machilus_yunnanensis GAGAGTAACA AGAAAGCAAA ACTAGAGTTA TTCCTGAAAA AATATTTGCT TTTTCAATTG
Machilus_thunbergii GAGAGTAACA AGAAAGCAAA ACTAGAGTTA TTCCTGAAAA AATATTTGCT TTTTCAATTG
Persea_americana GAGAGTAACA AGAAAGCAAA ACTAGAGTTA TTCCTGAAAA AATATTTGCT TTTTCAATTG
Lindera_glauca GAGAGTAACA AGAAAGCAAA ACTAGAGTTA TTCCTGAAAA AATATTTGCT TTTTCAATTG
Lindera_sericea GAGAGTAACA AGAAAGCAAA ACTAGAGTTA TTCCTGAAAA AATATTTGCT TTTTCAATTG
Lindera_megaphylla GAGAGTAACA AGAAAGCAAA ACTAGAGTTA TTCCTGAAAA AATATTTGCT TTTTCAATTG

....|....| ....|....| ....|....| ....|....| ....|....| ....|....|
4865 4875 4885 4895 4905 4915
Machilus_pauhoi AGATGGGATG ATCCTTTGAA TCACAGAATT TTCAATAATG TTAAGGTATA TTGTTTCCTG
Machilus_balansae AGATGGGATG ATCCTTTGAA TCACAGAATT TTCAATAATG TTAAGGTATA TTGTTTCCTG
Machilus_yunnanensis AGATGGGATG ATCCTTTGAA TCACAGAATT TTCAATAATG TTAAGGTATA TTGTTTCCTG
Machilus_thunbergii AGATGGGATG ATCCTTTGAA TCACAGAATT TTCAATAATG TTAAGGTATA TTGTTTCCTG
Persea_americana AGATGGGATG ATCCTTTGAA TCACAGAATT TTCAATAATG TTAAGGTATA TTGTTTCCTG
Lindera_glauca AGATGGGATG ATCCTTTGAA TCACAGAATT TTCAATAATG TTAAGGTATA TTGTTTCCTG
Lindera_sericea AGATGGGATG ATCCTTTGAA TCACAGAATT TTCAATAATG TTAAGGTATA TTGTTTCCTG
Lindera_megaphylla AGATGGGATG ATCCTTTGAA TCACAGAATT TTCAATAATG TTAAGGTATA TTGTTTCCTG

....|....| ....|....| ....|....| ....|....| ....|....| ....|....|
4925 4935 4945 4955 4965 4975
Machilus_pauhoi CTTAGACTAA TAAATGCAAA GGAAATTGCT ATATCCTCTA TTCAAGGAGG AGAAATGCAC
Machilus_balansae CTTAGACTAA TAAATGCAAA GGAAATTGCT ATATCCTCTA TTCAAGGAGG AGAAATGCAC
Machilus_yunnanensis CTTAGACTAA TAAATGCAAA GGAAATTGCT ATATCCTCTA TTCAAGGAGG AGAAATGCAC
Machilus_thunbergii CTTAGACTAA TAAATGCAAA GGAAATTGCT ATATCCTCTA TTCAAGGAGG AGAAATGCAC
Persea_americana CTTAGACTAA TAAATGCAAA GGAAATTGCT ATATCCTCTA TTCAAGGAGG AGAAATGCAC
Lindera_glauca CTTAGACTAA TAAATGCAAA GGAAATTGCT ATATCCTCTA TTCAAGGAGG AGAAATGCAC
Lindera_sericea CTTAGACTAA TAAATGCAAA GGAAATTGCT ATATCCTCTA TTCAAGGAGG AGAAATGCAC
Lindera_megaphylla CTTAGACTAA TAAATGCAAA GGAAATTGCT ATATCCTCTA TTCAAGGAGG AGAAATGCAC

....|....| ....|....| ....|....| ....|....| ....|....| ....|....|
4985 4995 5005 5015 5025 5035
Machilus_pauhoi CTGGATGTAA TGTTAATTCA GACGAATCTA ACTCTTCCAG AATTGATAAA AAAGGGAATA
Machilus_balansae CTGGATGTAA TGTTAATTCA GACGAATCTA ACTCTTCCAG AATTGATAAA AAAGGGAATA
Machilus_yunnanensis CTGGATGTAA TGTTAATTCA GACGAATCTA ACTCTTCCAG AATTGATAAA AAAGGGAATA
Machilus_thunbergii CTGGATGTAA TGTTAATTCA GACGAATCTA ACTCTTCCAG AATTGATAAA AAAGGGAATA

Persea_americana CTGGATGTAA TGTTAATTCA GACGAATCTA ACTCTTCCAG AATTGATAAA AAAGGGAATA
Lindera_glauca CTGGATGTAA TGTTAATTCA GACGAATCTA ACTCTTCCAG AATTGATAAA AAAGGGAATA
Lindera_sericea CTGGATGTAA TGTTAATTCA GACGAATCTA ACTCTTCCAG AATTGATAAA AAAGGGAATA
Lindera_megaphylla CTGGATGTAA TGTTAATTCA GACGAATCTA ACTCTTCCAG AATTGATAAA AAAGGGAATA

....|....| ....|....| ....|....| ....|....| ....|....| ....|....|
5045 5055 5065 5075 5085 5095

Machilus_pauhoi TTGATTCTCG AACCAGTACG TCTGTCTATA AAATGGGATA GACAATTTAT TATGTATCAA
Machilus_balansae TTGATTCTCG AACCAGTACG TCTGTCTATA AAATGGGATA GACAATTTAT TATGTATCAA
Machilus_yunnanensis TTGATTCTCG AACCAGTACG TCTGTCTATA AAATGGGATA GACAATTTAT TATGTATCAA
Machilus_thunbergii TTGATTCTCG AACCAGTACG TCTGTCTATA AAATGGGATA GACAATTTAT TATGTATCAA
Persea_americana TTGATTCTCG AACCAGTACG TCTGTCTATA AAATGGGATA GACAATTTAT TATGTATCAA
Lindera_glauca TTGATTCTCG AACCAGTACG TCTGTCTATA AAATGGGATA GACAATTTAT TATGTATCAA
Lindera_sericea TTGATTCTCG AACCAGTACG TCTGTCTATA AAATGGGATA GACAATTTAT TATGTATCAA
Lindera_megaphylla TTGATTCTCG AACCAGTACG TCTGTCTATA AAATGGGATA GACAATTTAT TATGTATCAA

....|....| ....|....| ....|....| ....|....| ....|....| ....|....|
5105 5115 5125 5135 5145 5155

Machilus_pauhoi ACCATAGGTA TCTCATTGGT CCATAATAAT AAATGCCAAA CTAATGAAAG ATATCGAGAA
Machilus_balansae ACCATAGGTA TCTCATTGGT CCATAATAAT AAATGCCAAA CTAATGAAAG ATATCGAGAA
Machilus_yunnanensis ACCATAGGTA TCTCATTGGT CCATAATAAT AAATGCCAAA CTAATGAAAG ATATCGAGAA
Machilus_thunbergii ACCATAGGTA TCTCATTGGT CCATAATAAT AAATGCCAAA CTAATGAAAG ATATCGAGAA
Persea_americana ACCATGGGTA TCCCATTGGT CCATAATAAT AAATGCCAAA CTAATGGAAG ATATCGAGAA
Lindera_glauca ACCATAGGTA TCTCATTGGT CCATAATAAT AAATACCAAA CTAATGGAAG ATATCGAGAA
Lindera_sericea ACCATAGGTA TCTCATTGGT CCATAATAAA AAATGCCAAA CTAATGGAAG ATATCGAGAA
Lindera_megaphylla ACCATAGGTA TCTCATTGGT CCATAATAAT AAATGCCAAA CTAATGGAAG ATATCGAGAA

....|....| ....|....| ....|....| ....|....| ....|....| ....|....|
5165 5175 5185 5195 5205 5215

Machilus_pauhoi AAAAGATATG TTGATGAGAA TTATTTCAAT GGATCCATTG TACAACAAAA AAAGATGCTT
Machilus_balansae AAAAGATATG TTGACGAGAA TTATTTCAAT GGATCCATTG TACAACAAAA AAAGATGCTT
Machilus_yunnanensis AAAAGATATG TTGATGAGAA TTATTTCAAT GGATCCATTG TACAACAAAA AAAGATGCTT
Machilus_thunbergii AAAAGATATG TTGATGAGAA TTATTTCAAT GGATCCATTG TACAACAAAA AAAGATGCTT
Persea_americana AAAAGATATG TTGATGAGAA TTATTTCAAT GGATCCATTG TACAACATAA AAAGATGCTT
Lindera_glauca AAAAGATATG TTGATGAGAA TTATTTCAAT GGATCCGTTG TACAACATAA AAAGATGCTT
Lindera_sericea AAAAGATATG TTGATGAGAA TTATTTCAAT GGATCCATTG TACAACATAA AAAGATGCTT
Lindera_megaphylla AAAAGATATG TTGATGAGAA TTATTTCAAT GGATCCATTG TACAACATAA AAAGATGCTT

....|....| ....|....| ....|....| ....|....| ....|....| ....|....|
5225 5235 5245 5255 5265 5275

Machilus_pauhoi GTGAATAGAG ACGAAAATCA TTATGATTTG CTTGTTCCTG AAAATATTCT ATCCCCTAGG
Machilus_balansae GTGAATAGAG ACGAAAATCA TTATGATTTG CTTGTTCCTG AAAATATTCT ATCCCCTAGG
Machilus_yunnanensis GTGAATAGAG ACGAAAATCA TTATGATTTG CTTGTTCCTG AAAATATTCT ATCCCCTAGG
Machilus_thunbergii GTGAATAGAG ACGAAAATCA TTATGATTTG CTTGTTCCTG AAAATATTCT ATCCCCTAGG

```
Persea_americana        GTGAATAGAG ACGAAAATCA TTATGATTTG CTTGTTCCTG AAAATATTCT ATCCCCTAGG
Lindera_glauca          GTGAATAGAG ACGAAAATCA TTATGATTTG CTTGTTCCTG AAAATATTCT ATCCCCTAGG
Lindera_sericea         GTGAATAGAG ACGAAAATCA TTATGATTTG CTTGTTCCTG AAAATATTCT ATCCCCTAGG
Lindera_megaphylla      GTGAATAGAG ACGAAAATCA TTATGATTTG CTTGTTCCTG AAAATATTCT ATCCCCTAGG

                        ....|....| ....|....| ....|....| ....|....| ....|....| ....|....|
                            5285       5295       5305       5315       5325       5335
Machilus_pauhoi         CGTCGTAGAG AATTGAGAAT TCTAATTTGT TTCAATTCCG GAAATAGGAA TGTTATGGAT
Machilus_balansae       CGTCGTAGAG AATTGAGAAT TCTAATTTGT TTCAATTCCG GAAATAGGAA TGTTATGGAT
Machilus_yunnanensis    CGTCGTAGAG AATTGAGAAT TCTAATTTGT TTCAATTCCG GAAATAGGAA TGTTATGGAT
Machilus_thunbergii     CGTCGTAGAG AATTGAGAAT TCTAATTTGT TTCAATTCCG GAAATAGGAA TGTTATGGAT
Persea_americana        CGTCGTAGAG AATTGAGAAT TCTAATTTGT TTCAATTCCG GAAATAGGAA TGTTATGGAT
Lindera_glauca          CGTCGTAGAG AATTGAGAAT TCTAATTTGT TTCAATTCCG GAAATAGGAA TGTTGTGGAT
Lindera_sericea         CGTCGTAGAG AATTGAGAAT TCTAATTTGT TTCAATTCCG GAAATAGGAA TGTTATGGAT
Lindera_megaphylla      CGTCGTAGAG AATTGAGAAT TCTAATTTGT TTCAATTCCG GAAATAGGAA TGTTATGGAT

                        ....|....| ....|....| ....|....| ....|....| ....|....| ....|....|
                            5345       5355       5365       5375       5385       5395
Machilus_pauhoi         AGAAATCCGG TATTTTTCAA TGACAACAAT GTAAGGAACT GGGGGCAATT TTTGGATGAG
Machilus_balansae       AGAAATCCGG TATTTTTCAA TGACAACAAT GTAAGGAACT GGGGGCAATT TTTGGATGAG
Machilus_yunnanensis    AGAAATCCGG TATTTTTCAA TGACAACAAT GTAAGGAACT GGGGGCAATT TTTGGATGAG
Machilus_thunbergii     AGAAATCCGG TATTTTTCAA TGACAACAAT GTAAGGAACT GGGGGCAATT TTTGGATGAG
Persea_americana        AGAAATCCGG TATTTTTCAA TGACAACAAT GTAAGGAACT GGGGACAATT TTTGGATGAG
Lindera_glauca          AGAAATCCGA TATTTTTCAA TGACAACAAT GTAAGGAACT GGGGGCAATT TTTGGATGAG
Lindera_sericea         AGAAATCCGG TATTTTTCAA TGACAACAAT GTAAGGAACT GGGGGCAATT TTTGGATGAG
Lindera_megaphylla      AGAAATCCGG TATTTTTCAA TGACAACAAT GTAAGGAACT GGGGGCAATT TTTGGATGAG

                        ....|....| ....|....| ....|....| ....|....| ....|....| ....|....|
                            5405       5415       5425       5435       5445       5455
Machilus_pauhoi         GACAAGCATA TTGATACAGA TATAAATAAA TTCATTCAAT TCAAATTGTT TCTTTGGCCC
Machilus_balansae       GACAAGCATA TTGATACAGA TATAAATAAA TTCATTCAAT TCAAATTGTT TCTTTGGCCC
Machilus_yunnanensis    GACAAGCATA TTGATACAGA TATAAATAAA TTCATTCAAT TCAAATTGTT TCTTTGGCCC
Machilus_thunbergii     GACAAGCATA TTGATACAGA TATAAATAAA TTCATTCAAT TCAAATTGTT TCTTTGGCCC
Persea_americana        GACAAGCATA TTGATACAGA TATAAATAAA TTCATTCAAT TCAAATTGTT TCTTTGGCCA
Lindera_glauca          GACAAGCATA TTGATACAGA TATAAATAAA TTCATTCAAT TCAAATTGTT TCTTTGGCCC
Lindera_sericea         GACAAGCATA TTGATACAGA TATAAATAAA TTCATTCAAT TCAAATTGTT TCTTTGGCCC
Lindera_megaphylla      GACAAGCATA TTGATACAGA TATAAATAAA TTCATTCAAT TCAAATTGTT TCTTTGGCCC

                        ....|....| ....|....| ....|....| ....|....| ....|....| ....|....|
                            5465       5475       5485       5495       5505       5515
Machilus_pauhoi         AATTATCGAT TAGAGGATTT AGCTTGTATG AATCGCTACT GGTTTGATAC CAATAATGGC
Machilus_balansae       AATTATCGAT TAGAGGATTT AGCTTGTATG AATCGCTACT GGTTTGATAC CAATAATGGC
Machilus_yunnanensis    AATTATCGAT TAGAGGATTT AGCTTGTATG AATCGCTACT GGTTTGATAC CAATAATGGC
Machilus_thunbergii     AATTATCGAT TAGAGGATTT AGCTTGTATG AATCGCTACT GGTTTGATAC CAATAATGGC
```

Persea_americana	AATTATCGAT TAGAGGATTT AGCTTGTATG AATCGCTACT GGTTTGATAC CAATAATGGC
Lindera_glauca	AATTATCGAT TAGAGGATTT AGCTTGTATG AATCGCTACT GGTTTGATAC CAATAATGGC
Lindera_sericea	AATTATCGAT TAGAGGATTT AGCTTGTATG AATCGCTACT GGTTTGATAC CAATAATGGC
Lindera_megaphylla	AATTATCGAT TAGAGGATTT AGCTTGTATG AATCGCTACT GGTTTGATAC CAATAATGGC

	....|....| ....|....| ....|....| ....|....| ....|....| ....
	5525 5535 5545 5555 5565
Machilus_pauhoi	AGCCGTTTCA GTATGTCAAG GATACATATG TATCCACGAT TCGGAATTAG TTGA
Machilus_balansae	AGCCGTTTCA GTATGTCAAG GATACATATG TATCCACGAT TCGGAATTAG TTGA
Machilus_yunnanensis	AGCCGTTTCA GTATGTCAAG GATACATATG TATCCACGAT TCGGAATTAG TTGA
Machilus_thunbergii	AGCCGTTTCA GTATGTCAAG GATACATATG TATCCACGAT TCGGAATTAG TTGA
Persea_americana	AGCCGTTTCA GTATGTCAAG GATACATATG TATCCACGAT TCGGAATTAG TTGA
Lindera_glauca	AGCCGTTTCA GTATGTCAAG GATACATATG TATCCACGAT TCGGAATTAG TTGA
Lindera_sericea	AGCCGTTTCA GTATGTCAAG GATACATATG TATCCACGAT TCGGAATTAG TTGA
Lindera_megaphylla	GGCCGTTTCA GTATGTCAAG GATACATATG TATCCACGAT TCGGAATTAG TTGA
